# Fe(II)/Et_3_N-Relay-catalyzed domino reaction of isoxazoles with imidazolium salts in the synthesis of methyl 4-imidazolylpyrrole-2-carboxylates, its ylide and betaine derivatives

**DOI:** 10.3762/bjoc.11.189

**Published:** 2015-09-24

**Authors:** Ekaterina E Galenko, Olesya A Tomashenko, Alexander F Khlebnikov, Mikhail S Novikov, Taras L Panikorovskii

**Affiliations:** 1 Institute of Chemistry, Saint Petersburg State University, Universitetskii pr. 26, 198504, St. Petersburg, Russia; 2 Institute of Earth Sciences, Saint Petersburg State University, University Emb. 7/9, 199034, St. Petersburg, Russia

**Keywords:** imidazole, isoxazole, NHC carbene, pyrrole-2-carboxylate, relay catalysis

## Abstract

A simple approach was developed for the synthesis of methyl 4-imidazolylpyrrole-2-carboxylates from easily available compounds, 5-methoxyisoxazoles and phenacylimidazolium salts under hybrid Fe(II)/Et_3_N relay catalysis. The products were easily transformed into the corresponding 3-(5-methoxycarbonyl-1*H*-imidazol-3-ium-3-yl)pyrrol-1-ides, which in turn can be hydrolyzed under basic conditions into the corresponding betaines. A carbene tautomeric form of the 4-methoxycarbonyl-substituted imidazolylpyrrolides was trapped by reaction with sulfur affording the corresponding imidazolethiones under very mild conditions.

## Introduction

**Scheme 1:**
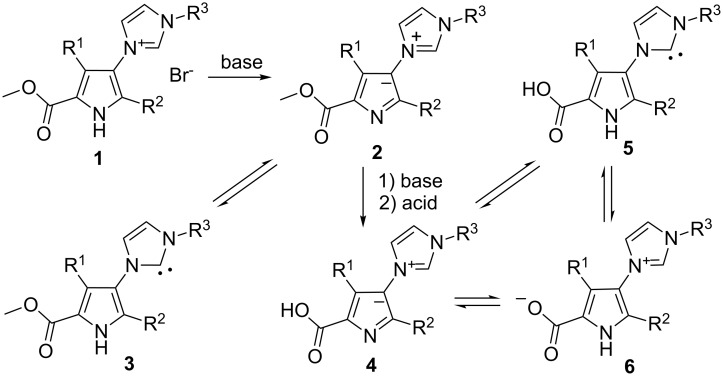
The formation and possible tautomeric equilibria of 2-methoxycarbonyl- and 2-carboxy-3-(1*H*-imidazol-3-ium-3-yl)pyrrol-1-ides **2** and **4**.

Pyrrole-2-carboxyate and imidazole units are present in bioactive pyrrole-imidazole alkaloids and pyrrole-imidazole polyamides [1–5]. Derivatives of 4-imidazolylpyrrole-2-carboxylic acid are much less known, though some of these compounds showed various bioactivities [6–9] and were patented as inhibitors of c-Met protein kinase [8] and as anti-inflammatory agents [9]. Additionally, 5-alkoxycarbonylpyrrol-3-ylimidazolium salts **1** attracted our attention as the potential precursors of ylides **2**, which in principle could be in equilibrium with N-heterocyclic carbenes (NHC) **3**. Furthermore, hydrolysis of **2** could provide an easy access to unknown carboxy-substituted ylides **4,** and then could potentially be in the equilibrium with N-heterocyclic carbenes **5** and betaine **6** (Scheme 1). Interplay between N-heterocyclic carbenes, heterocyclic betaines and ylides is currently intensively investigated as a promising route for tuning NHC for specific use. This topic was extensively reviewed [10–14] and many papers were published recently [15–22]. The **4–6** triads could particularly be interesting as new ligands for the preparation of mixed complexes: a chelate complex with the carboxypyrrole part and a monodentate NHC complex. Not so much is known about metal chelate complexes of pyrrole-2-carboxylic acid [23–25]. In some cases these complexes were prepared via dehydration of the corresponding proline complexes [25]. Substituted pyrrole-2-carboxylic acids as ligands of complexes are seldom used and are only exemplified by complexes of indole-2-carboxylic acid [26–27] and coenzyme pyrroloquinoline quinone [28].

**Scheme 2:**
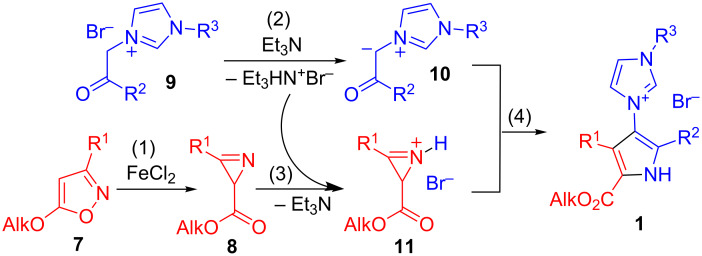
FeCl_2/_Et_3_N-catalyzed domino sequence leading to 5-alkoxycarbonylpyrrol-3-ylimidazolium salts **1**.

The synthesis of 4-imidazolylpyrrole-2-carboxylic acid derivatives usually involves the corresponding pyrrole with a functional group allowing the formation of a imidazole ring [8–9]. Recently we developed a new approach to 3-(1*H*-pyrrol-3-yl)-1*H*-imidazoles based on the formation of a pyrrole ring via the reaction of 2*H*-azirines with 1-alkyl-3-phenacyl-1*H*-imidazolium bromides [29], in which one example of the synthesis of ethyl 4-imidazolylpyrrole-2-carboxylate from ethyl 3-methyl-2*H*-azirine-2-carboxylate was described. Earlier it was found that alkyl 2*H*-azirine-2-carboxylates can be prepared by isomerization of 5-alkoxyisoxazoles under Fe(II)-salt catalysis [30]. Quite recently this isomerization has been used for the preparation of substituted pyrrole-2-carboxylic acid derivatives by the domino reaction of 3-aryl-5-methoxyisoxazoles with 1,3-dicarbonyl compounds under relay catalysis [31]. Taking into account the facts discussed above, we envisioned that the synthesis of 5-alkoxycarbonylpyrrol-3-ylimidazolium salts **1** could be carried out starting from easily available 5-alkoxyisoxazoles **7** [32–33] and 1-alkyl-3-phenacyl-1*H*-imidazolium bromides **9** according to Scheme 2, whereby excluding the isolation of often unstable 2*H*-azirines [34].

## Results and Discussion

The synthetic scheme (Scheme 2) implies an implementation of all stages (1: generation of azirine **8** from isoxazole **7** under FeCl_2_ catalysis; 2: formation of phenacylimidazolium ylide **10** induced by Et_3_N; 3: activation of azirine **8** with Et_3_HN^+^Br^−^; 4: reaction of the activated azirine **11** with the imidazolium ylide **10**) as a domino reaction under relay catalysis [35–36]. A simple procedure, consisting of stirring a mixture of isoxazole **7**, phenacylimidazolium salt **9**, FeCl_2_·4H_2_O and Et_3_N in MeCN for 6–7 h at 45 °C, gave 5-alkoxycarbonylpyrrol-3-ylimidazolium bromides **1** in reasonable yields (Table 1). All new compounds were characterized by ^1^H and ^13^C NMR, IR spectroscopy, and mass spectrometry.

**Table 1:** The synthesis of 5-alkoxycarbonylpyrrol-3-ylimidazolium salts **1** by the domino reaction of 5-methoxyisoxazoles **7** and phenacylimidazolium bromides **9** under FeCl_2_·4H_2_O/Et_3_N catalysis.

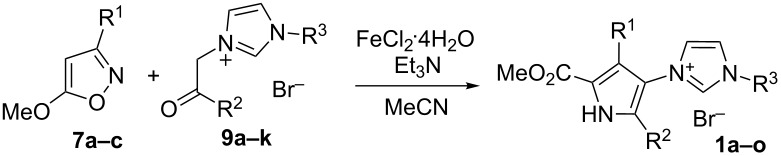						

entry	R^1^	R^2^	R^3^	**7 + 9**	**1**	yield, %

1	Ph	Ph	Me	**7a + 9a**	**1a**	54
2	Ph	4-ClC_6_H_4_	Me	**7a + 9b**	**1b**	54
3	Ph	4-NO_2_C_6_H_4_	Me	**7a + 9c**	**1c**	63
4	4-BrC_6_H_4_	3-BrC_6_H_4_	Me	**7b + 9d**	**1d**	66
5	Ph	Ph	Ph	**7a + 9e**	**1e**	68
6	Ph	4-MeOC_6_H_4_	Ph	**7a + 9f**	**1f**	71
7	Ph	4-BrC_6_H_4_	Ph	**7a + 9g**	**1g**	57
8	Me	Ph	Ph	**7c + 9e**	**1h**	54
9	Me	4-MeOC_6_H_4_	Ph	**7c + 9f**	**1i**	59
10	Ph	Ph	Bn	**7a + 9h**	**1j**	51
11	Ph	4-MeOC_6_H_4_	Bn	**7a + 9i**	**1k**	51
12	Ph	4-FC_6_H_4_	Bn	**7a + 9j**	**1l**	55
13	Ph	4-ClC_6_H_4_	Bn	**7a + 9k**	**1m**	72
14	Me	Ph	Bn	**7c + 9h**	**1n**,	47
15	Me	4-ClC_6_H_4_	Bn	**7c + 9k**	**1o**,	69

**Scheme 3:**
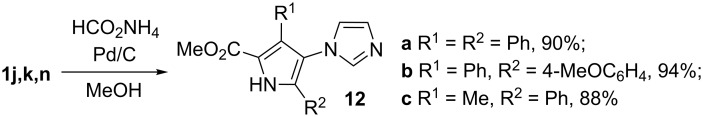
The synthesis of methyl 4-(1*H*-imidazol-1-yl)-1*H*-pyrrole-2-carboxylates **12**.

1-Benzyl-3-(1*H*-pyrrol-3-yl)-1*H*-imidazol-3-ium bromides **1** can be easily debenzylated on Pd/C, with ammonium formate as a source of hydrogen, to give the corresponding methyl 4-(1*H*-imidazol-1-yl)-1*H*-pyrrole-2-carboxylates **12** in high yields (Scheme 3).

The reaction of aq KOH with imidazolium bromides **1** at room temperature afforded the corresponding stable ylides **2** (Table 2) in high yields without hydrolyzing the ester group. Ylides **2** can also be debenzylated, affording the corresponding pyrrolyl imidazoles **12**. Thus, ylide **2h** was debenzylated on Pd/C with hydrogen to produce methyl 5-(4-fluorophenyl)-4-(1*H*-imidazol-1-yl)-3-phenyl-1*H*-pyrrole-2-carboxylate (**12d**) in quantitive yield.

**Table 2:** The preparation of ylides **2** and imidazolethiones **13**.

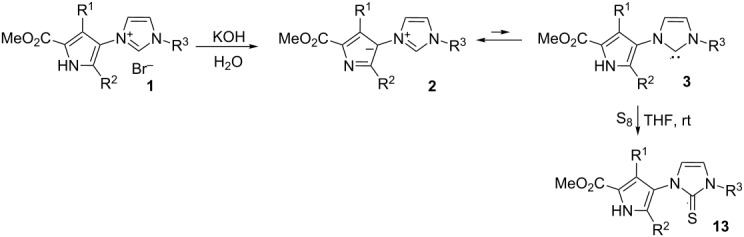						

entry	R^1^	R^2^	R^3^	**1**	**2**, yield, %	**13**, yield, %

1	Ph	Ph	Me	**1a**	**2a**, 71	**13a**, 80
2	Ph	4-ClC_6_H_4_	Me	**1b**	**2b**, 86	**13b**, 81
3	Ph	4-NO_2_C_6_H_4_	Me	**1c**	**2c**, 98	**13c**, 93
4	4-BrC_6_H_4_	3-BrC_6_H_4_	Me	**1d**	**2d**, 80	**13d**, 92
5	Ph	Ph	Ph	**1e**	**2e**, 91	**13e**, 91
6	Ph	4-BrC_6_H_4_	Ph	**1g**	**2f**, 94	**13f,** 80
7	Me	4-MeOC_6_H_4_	Ph	**1i**	**2g**, 82	**13g**, 90
8	Ph	4-FC_6_H_4_	Bn	**1l**	**2h**, 88	**–**
9	Ph	4-ClC_6_H_4_	Bn	**1m**	**2i**, 95	**13h**, 80

As mentioned above, ylides **2** can potentially be in tautomeric equilibrium with N-heterocyclic carbenes **3**. No signals characteristic for carbenes **3a–i**, however, were found in the NMR spectra of compound **2a–i**. According to the DFT calculations mesomeric electron-donating substituents R^2^ in the pyrrole ring (Table 3, cf. entries 3 and 5) stabilize carbene tautomer **3** slightly. Changing *N*-alkyl for *N*-aryl substituents in the imidazolium ring has a relatively small effect on the tautomeric ratio (Table 3, entries 1 and 3). At the same time, the solvent has a dramatic effect on the equilibrium position. In the gas phase carbenes **3** are more thermodynamically stable than the corresponding ylides **2**. However, as one can expect, the solvent stabilizes the zwitterion species much better than the uncharged ones. According to the DFT calculations in solution the equilibrium is shifted to the ylide side and the higher the polarity of the solvent the stronger the shift. Nevertheless, carbene tautomers **3** were trapped in THF by reaction with sulfur, leading to imidazolethiones **13**, under unusually mild conditions (Table 2) [10,15–16].

**Table 3:** Relative free energies (carbene **3/**ylide **2**, ylide **4/**betaine **6** and carbene **5/**betaine **6**) computed at the DFT B3LYP/6-31+G(d,p) level in the gas phase or with the PCM model for the corresponding solvent at 298 K. Calculated data are based on the most stable conformer of **2−6**.

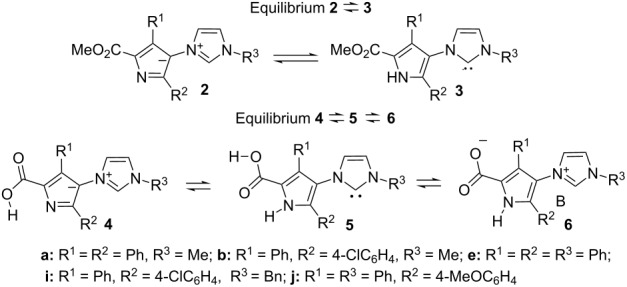					

entry	equilibrium system	Δ*G***_3-2_**, kcal·mol^−1^			
		gas phase	DCM	THF	DMSO

equilibrium **2** 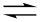 **3** (Δ*G***_3-2_**, kcal·mol^−1^)					

1	**a**	–1.5	9.6	–	12.3
2	**b**	–0.5	10.2	9.5	12.8
3	**e**	–1.3	7.9	–	10.1
4	**i**	–0.8	9.6	9.3	12.4
5	**j**	–2.7	6.8	–	9.5

equilibrium **4** 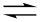 **5** 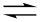 **6** (Δ*G***_4-6_/**Δ*G***_5-6_**, kcal·mol^−1^)					

6	**a**	–11.4/–8.6	1.5/16.0	–	4.0/20.0
7	**b**	–12.4/–9.0	1.2/15.7	0.5/14.6	4.0/20.5
8	**e**	–11.6/–8.8	1.9/14.4	–	4.0/17.9
9	**i**	–10.9/–7.0	1.8/16.3	–	3.8/20.1
10	**j**	–11.3/–9.1	2.1/14.1	–	4.3/18.0

**Scheme 4:**
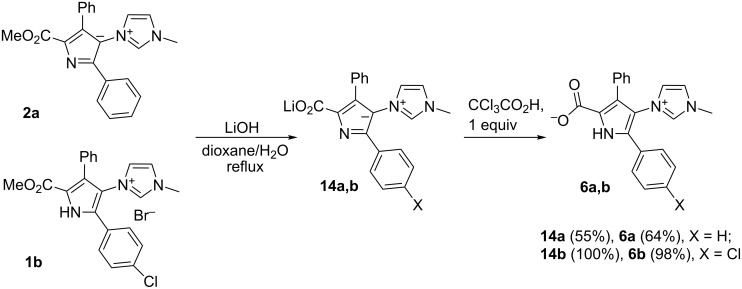
The hydrolysis of ylide **2a** and bromide **1b**.

Hydrolysis of the ester group in compounds **1** or **2** needs much harsher conditions. Reflux of **1b** in a NaOH solution in methanol/water 2:1 or in a LiOH solution in THF/water 9:1 leaved the ester group unchanged and only by refluxing **1b** in a LiOH solution in dioxane/water 9:1 the Li salt **14b** was obtained. Betaine **6b** was isolated in quantitative yield after treatment of **14b** with 1 equiv of CCl_3_CO_2_H (Scheme 4). Ylide **2a** was hydrolyzed with a lower yield due to the high solubility of the salt **14a** in water.

Betaines **6** can, in principle, exist in tautomeric equilibrium with the corresponding carboxy-substituted ylides **4** and N-heterocyclic carbenes **5**. The results of the DFT study of the relative thermodynamic stability of tautomers **4–6** revealed (Table 3, entries 6–10) that solvent has a crucial impact on the equilibrium position. Betaines **6** are the most unstable species in the gas phase, whereas in solvents they become the most stable species and therefore dominate in solution. It is worth noting that the concentration of carbene **5** in solution is negligible and much less than carbene **3** in equilibrium **2/3**. It is therefore not surprising that the corresponding imidazolethiones were not formed from **6a** with sulfur either in THF (rt or reflux) or even in refluxing dioxane.

**Figure 1:**
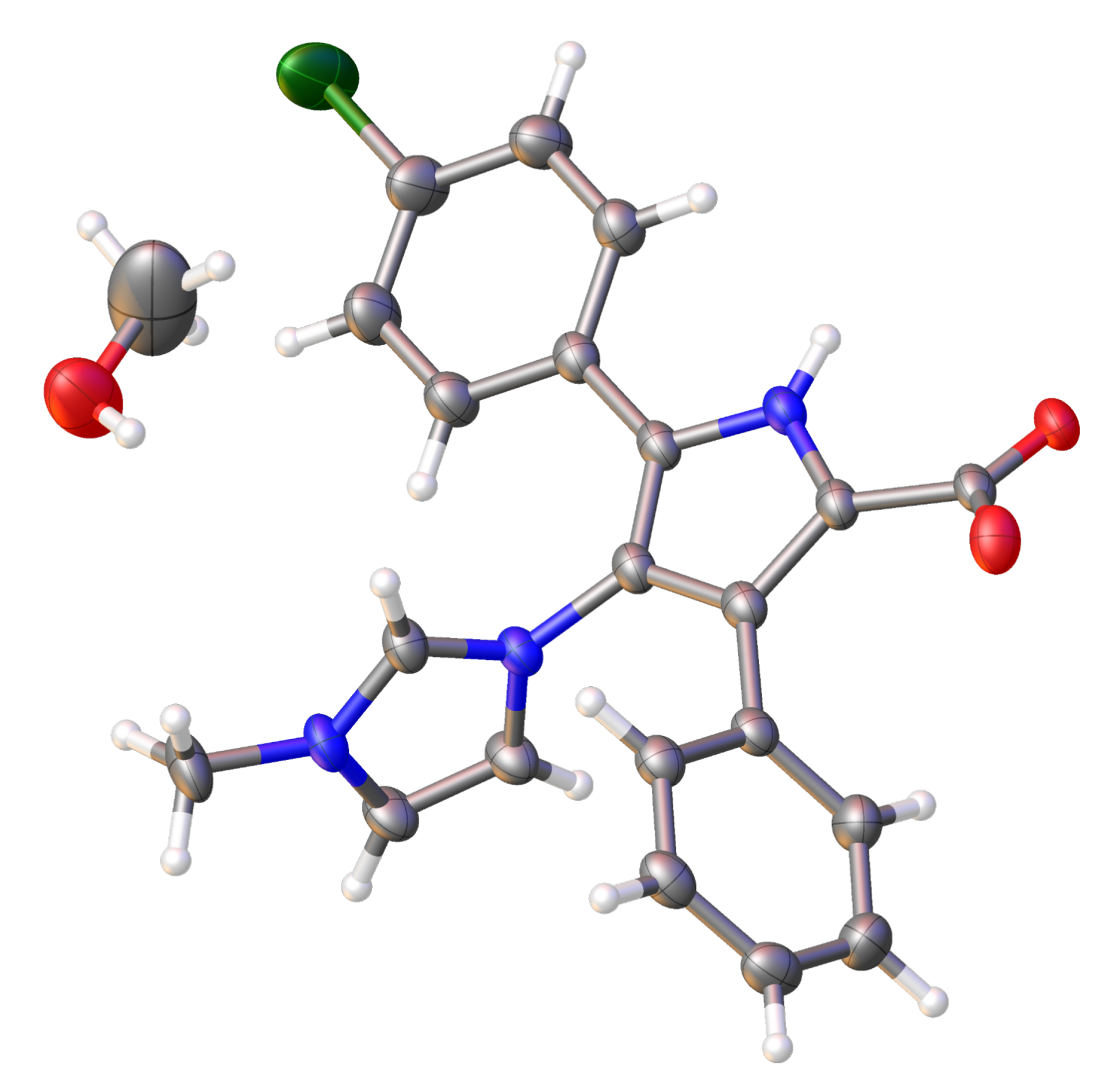
Molecular structure of compound **6b** (CCDC 1406417). Carbon, nitrogen and oxygen atoms are grey, blue and red, respectively. Chlorine is dark-green. Thermal ellipsoids are drawn at the 50% probability level.

The structure of the crystalline compound **6b** was also analyzed by single X-ray diffraction (Figure 1). X-ray analysis cannot give preference to one of the three possible structures with the same positions of heavy atoms: ylide **4b**, carbene **5b** and betaine **6b**. A comparison, however, of the carbon–oxygen bond lengths of the carboxy group obtained from X-ray analysis with bond lengths calculated at the B3LYP/6-31+G(d,p) level of theory for structures mentioned above shows that betaine **6b** is the correct structure (for calculated geometries of ylide **4b**, carbene **5b** and betaine **6b** see Supporting Information File 1). It can therefore be concluded that betaine **6b** is thermodynamically much more stable than the corresponding ylide **4b** and carbene **5b** both in solution and in the solid state.

## Conclusion

A convenient approach was developed for the synthesis of derivatives of methyl 4-imidazolylpyrrole-2-carboxylates from easily available compounds, 5-methoxyisoxazoles and phenacylimidazolium salts, under hybrid Fe(II)/Et_3_N relay catalysis. 3-(5-Methoxycarbonyl-1*H*-pyrrol-3-yl)-1*H*-imidazol-3-ium bromides were easily transformed into the corresponding 3-(5-methoxycarbonyl-1*H*-imidazol-3-ium-3-yl)pyrrol-1-ides. The carbene form of the latter were trapped by reaction with sulfur with formation of the corresponding imidazolethiones under very mild conditions. Hydrolysis of 3-(5-methoxycarbonyl-1*H*-pyrrol-3-yl)-1*H*-imidazol-3-ium bromides under harsh conditions leads to (1*H*-imidazol-3-ium-3-yl)-1*H*-pyrrole-2-carboxylates which are potential ligands for hybrid chelate/NHC complexes.

## Experimental

### General methods

Melting points were determined on a capillary melting point apparatus Stuart^®^ SMP30. ^1^H (400 MHz) and ^13^C (100 MHz) NMR spectra were determined in CDCl_3_ and DMSO-*d*_6_ with a Bruker AVANCE III 400 spectrometer. Chemical shifts (δ) are reported in parts per million downfield from tetramethylsilane (TMS δ = 0.00). ^1^H NMR spectra were calibrated according to the residual peak of CDCl_3_ (7.26 ppm) or DMSO-*d*_6_ (2.50 ppm). For all new compounds ^13^C{^1^H} and ^13^C DEPT135 were recorded and calibrated according to the peak of CDCl_3_ (77.00 ppm) or DMSO-*d*_6_ (39.51 ppm). Mass spectra were recorded on a Bruker maXis HRMS–ESI–QTOF, with electrospray ionization in positive mode. IR spectra were recorded on a Bruker FTIR spectrometer Tensor 27 for tablets in KBr, only characteristic absorption is indicated. The single crystal X-ray diffraction experiment was performed on Agilent Technologies SuperNova diffractometer at 100 K using monochromated Cu Kα radiation. Thin-layer chromatography (TLC) was conducted on aluminium sheets with 0.2 mm silica gel (fluorescent indicator, Macherey-Nagel). The isoxazoles **7** [37–38] and imidazolium salts **9** [29] were synthesized by known literature procedures.

**General procedure for the synthesis of 5-methoxycarbonylpyrrol-3-ylimidazolium bromides 1a–o from isoxazoles 7a–c and imidazolium bromides 9a–k.** Isoxazole **7** (1.2–1.5 mmol) and imidazolium bromide **9** (1.0 mmol) were suspended in MeCN (4 mL), FeCl_2_·4H_2_O (0.06–0.08 mmol, 5 mol % calcd on isoxazole) and Et_3_N (3.0 mmol, 3 equiv) were added and the mixture was stirred at 45 °C for 6–7 h (monitored by TLC). Reaction mixture was evaporated to dryness, ethyl acetate was added and the precipitate formed was filtered off and washed with ethyl acetate or an ethyl acetate/CH_2_Cl_2_ mixture. The residue was purified by column chromatography on silica gel (CH_2_Cl_2_/MeOH 12:1), additionally washed with ethyl acetate or an ethyl acetate/CH_2_Cl_2_ mixture and dried to give the analytically pure compound.

**3-(2-(4-Chlorophenyl)-5-methoxycarbonyl-4-phenyl-1*H*-pyrrol-3-yl)-1-methyl-1*H*-imidazol-3-ium bromide (1b):** colorless solid, mp 242–243 °C (dec, ethyl acetate), yield 204 mg, 54%, obtained from 5-methoxy-3-phenylisoxazole (**7a**, 175 mg, 1 mmol), 3-(2-(4-chlorophenyl)-2-oxoethyl)-1-methyl-1*H*-imidazol-3-ium bromide (**9b**, 253 mg, 0.8 mmol), FeCl_2_·4H_2_O (10 mg, 0.05 mmol, 5 mol %) and Et_3_N (242 mg, 2.4 mmol) according to the general procedure. ^1^Н NMR (DMSO-*d*_6_) δ 3.68 (s, 3H), 3.83 (s, 3H), 7.25–7.28 (m, 2H), 7.31–7.32 (m, 3H), 7.37–7.40 (m, 2H), 7.48–7.50 (m, 2H), 7.81–7.82 (m, 1H), 7.83–7.84 (m, 1H), 9.35-9.36 (m, 1H), 13.03 (br s, 1H); ^13^С NMR (DMSO-*d*_6_) δ 36.2 (CH_3_), 51.5 (CH_3_), 116.5 (C), 118.1(C), 124.2 (CH), 125.6 (CH), 126.6 (C), 127.8 (CH), 127.9 (CH), 128.0 (C), 128.9 (CH), 129.5 (CH), 129.7 (CH), 130.4 (C), 130.6 (C), 133.8 (C), 138.8 (CH), 160.2 (C); HRMS–ESI (*m*/*z*): [M – Br]^+^ calcd for C_22_Н_19_ClN_3_O_2_, 392.1160; found, 392.1168; IR (KBr, cm^−1^) ν: 3389, 3042, 1706.

**General procedure for debenzylation of 1-benzyl-3-pyrrol-3-yl-1*H*-imidazol-3-ium bromides 1j,k,n.** 1-Benzyl-1*H*-imidazol-3-ium bromide **1** (100 mg) was dissolved in MeOH (10 mL), Pd/C (10 mg, 10 wt %) and ammonium formate (10 equiv) were added. The suspension was stirred under reflux for 1 h (monitored by TLC). The reaction mixture was filtered to remove Pd/C, MeOH was evaporated under reduced pressure, water was added to the residue and the product was filtered, washed with water and dried to give the analytically pure compound.

**Methyl 4-(1*H*-imidazol-1-yl)-3,5-diphenyl-1*H*-pyrrole-2-carboxylate (12a)**: colorless solid, mp 239–241 °C (dec., water), yield 128 mg, 90%, obtained from 1-benzyl-3-(5-(methoxycarbonyl)-2,4-diphenyl-1*H*-pyrrol-3-yl)-1*H*-imidazol-3-ium bromide (**1j**, 220 mg, 0.43 mmol), Pd/C (22 mg, 10 wt %) and ammonium formate (270 mg, 4.3 mmol) according to the general procedure. ^1^Н NMR (DMSO-*d*_6_) δ 3.66 (s, 3H), 6.89–6.90 (m, 1H), 7.10–7.11 (m, 1H), 7.21–7.26 (m, 7H), 7.30–7.33 (m, 3H), 7.51–7.52 (m, 1H), 12.49 (br s, 1H); ^13^С NMR (DMSO-*d*_6_) δ 51.2 (CH_3_), 117.3 (C), 119.1 (C), 122.5 (CH), 126.9 (CH), 127.1 (CH), 127.4 (CH), 128.2 (CH), 128.5 (CH), 128.69 (CH), 128.70 (C), 128.9 (C), 129.7 (CH), 131.1 (C), 131.8 (C), 139.1 (CH), 160.5 (C); HRMS–ESI (*m*/*z*): [M + H]^+^ calcd for C_21_Н_18_N_3_O_2_, 344.1394; found, 344.1401; IR (KBr, cm^–1^) ν: 3124, 2951, 1688.

**General procedure for the synthesis of pyrrolydes 2 from 5-methoxycarbonylpyrrol-3-ylimidazolium bromides 1.** A suspension of 3-(1*H*-pyrrol)-1*H*-imidazol-3-ium bromide **1** (1 mmol) in aqueous solution of KOH (2 mmol, 2 equiv, 5 mL H_2_O) was sonicated for 5 min and then vigorously stirred for 12 h. The precipitate was filtered, washed with water (2–3 mL) and dried to give analytically pure compound.

**2-Methoxycarbonyl-4-(1-methyl-1*H*-imidazol-3-ium-3-yl)-3,5-diphenylpyrrol-1-ide (2a):** colorless solid, mp 237–238 °C (dec.), yield 188 mg, 71%, obtained from 3-(5-(methoxycarbonyl)-2,4-diphenyl-1*H*-pyrrol-3-yl)-1-methyl-1*H*-imidazol-3-ium bromide (**1a**, 323 mg, 0.737 mmol) and a aqueous solution of KOH (83 mg, 1.482 mmol, 4 mL H_2_O) according to the general procedure. ^1^Н NMR (DMSO-*d*_6_) δ 3.51 (s, 3H), 3.81 (s, 3H), 7.03–7.21 (m, 10H), 7.69–7.72 (m. 2H), 9.11 (s, 1H); ^13^С NMR (DMSO-*d*_6_) δ 35.7 (CH_3_), 49.4 (CH_3_), 115.2 (C), 123.3 (CH), 124.6 (CH), 124.9 (CH), 125.2 (CH), 125.8 (C), 126.3 (CH), 127.0 (CH), 128.0 (CH), 128.0 (C), 129.6 (CH), 135.5 (C), 135.8 (C), 137.1 (C), 137.9 (CH), 165.0 (C); HRMS–ESI (*m*/*z*): [M + H]^+^ calcd for C_22_H_20_N_3_O_2_, 358.1550; found, 358.1566; IR (KBr, cm^–1^) ν: 3528, 3144, 3059, 1676.

**General procedure for the synthesis of 4-(2-thioxo-2,3-dihydro-1*H*-imidazol-1-yl)-1*H*-pyrrol-2-carboxylates 13 from pyrrolides 2.** A suspension of 3-(1*H*-imidazol-3-ium-3-yl)-5-(methoxycarbonyl)-pyrrol-1-ide (**2**, 1 mmol) and sulfur (2 mmol, 2 equiv) in dry THF was stirred at rt for 1–2 hours (monitored by TLC). Then the reaction mixture was evaporated to dryness and the residue was purified by column chromatography on silica gel (hexane/ethyl acetate from 1:1 to 0:1) to give the analytically pure compound.

**Methyl 4-(3-methyl-2-thioxo-2,3-dihydro-1*H*-imidazol-1-yl)-3,5-diphenyl-1*H*-pyrrole-2-carboxylate (13a)**: colorless solid, mp 261–262 °C, yield 43 mg, 80%, obtained from 2-(methoxycarbonyl)-4-(1-methyl-1*H*-imidazol-3-ium-3-yl)-3,5-diphenylpyrrol-1-ide (**2a**, 50 mg, 0.140 mmol) and sulfur (9 mg, 0.280 mmol) according to the general procedure. ^1^Н NMR (DMSO-*d*_6_) δ 3.46 (s, 3H), 3.67 (s, 3H), 6.84 (d, *J* = 2.3 Hz, 1H), 7.03 (d, *J* = 2.3 Hz, 1H), 7.17–7.42 (m, 8H), 7.52 (d, *J* = 7.0 Hz, 2H), 12.41 (s, 1H); ^13^С NMR (DMSO-*d*_6_) δ 34.9 (CH_3_), 51.1 (CH_3_), 117.1 (C), 118.9 (CH), 119.6 (CH), 119.7 (C), 127.0 (CH), 127.3 (CH), 127.5 (CH), 128.1 (CH), 128.3 (CH), 129.4 (C), 129.7 (C), 130.1 (CH), 132.1 (C), 132.4 (C), 160.6 (C), 165.4 (C); HRMS–ESI (*m*/*z*): [M + Na]^+^ calcd for C_22_H_19_N_3_O_2_SNa, 412.1090; found 412.1112; IR (KBr, cm^−1^) ν: 3304, 1662, 1454, 1375.

**5-(4-Chlorophenyl)-4-(1-methyl-1*H*-imidazol-3-ium-3-yl)-3-phenyl-1*H*-pyrrole-2-carboxylate (6b)**. A suspension of 3-(2-(4-chlorophenyl)-5-(methoxycarbonyl)-4-phenyl-1*H*-pyrrol-3-yl)-1-methyl-1*H*-imidazol-3-ium bromide **1b** (100 mg, 0.212 mmol) and LiOH (253 mg, 10.6 mmol, 50 equiv) in dioxane (30 mL) and water (3 mL) was stirred at 110 °C for 24 h. Then reaction mixture was evaporated to dryness and water (5 mL) was added. The suspension was filtered, the solid was washed with water (2 × 5 mL) and thoroughly dried to obtain lithium salt **14b** in quantitive yield. To convert the lithium salt **14b** into **6b** trichloroacetic acid (35 mg, 0.212 mmol, 1 equiv) was added to a suspension of the lithium salt in water (5 mL). The suspension was sonicated for 5 min, stirred for 1 h and filtered. The solid was washed with water and dried to obtain **6b** as a colorless solid, mp 205–207 °C, yield 79 mg, 98%. ^1^Н NMR (DMSO-*d*_6_) δ 3.82 (s, 3H), 7.11–7.26 (m, 5H), 7.29 (d, *J* = 8.5 Hz, 2H), 7.36 (d, *J* = 8.5 Hz, 2H), 7.77 (s, 1H), 7.80 (s, 1H), 9.26 (s, 1H); ^13^С NMR (DMSO-*d*_6_) δ 36.0 (CH_3_), 115.1 (C), 120.8 (C), 123.9 (CH), 124.0 (C), 124.2 (C), 125.9 (CH), 126.0 (CH), 127.1 (CH), 128.4 (CH), 128.42 (C), 128.6 (CH), 130.1 (CH), 131.8 (C), 133.1 (C), 138.6 (CH), 162.2 (C); HRMS–ESI (*m*/*z*): [M + H]^+^ calcd for C_21_H_17_ClN_3_O_2_, 378.1004; found, 378.1009; IR (KBr, cm^−1^) ν: 3498, 3033, 1694. Carboxylate **6a** (CCDC 1406417) was analyzed by single X-ray diffraction. It is triclinic *P−*1, *a* = 9.2999(5) Å, *b* = 9.5698(4) Å, *c* = 15.3875(5) Å, *α* = 72.782(3)°, *β* = 78.712(4)°, *γ* = 66.401(4)°, *V* = 1194.02(9) Å^3^, *Z* = 2, 4960 unique reflections were measured, which were used in all calculations. The final *R*_1_ was 0.0561 and *wR*_2_ was 0.1681 (all data) (>2sigma(I)) (see Supporting Information File 1 for details).

## Supporting Information

File 1 Detailed experimental procedures including characterization data for all synthesized compounds, ^1^H and ^13^C NMR spectra for all new compounds. Computational details: energies of molecules, transition states and their Cartesian coordinates of atoms. X-ray details.
