# Supplementary material for: Fe(II)/Et3N-Relay-catalyzed domino reaction of isoxazoles with imidazolium salts in the synthesis of methyl 4-imidazolylpyrrole-2-carboxylates, its ylide and betaine derivatives
Source: Beilstein J Org Chem. 2015 Sep 24;11:1732–40. doi: 10.3762/bjoc.11.189 (PMC4660897; doi:10.3762/bjoc.11.189)
Supplement: File 1 — Detailed experimental procedures including characterization data for all synthesized compounds, 1H and 13C NMR spectra for all new compounds. Computational details: energies of molecules, transition states and their Cartesian coordinates of atoms. X-ray details. [file Beilstein_J_Org_Chem-11-1732-s001.pdf]

Supporting Information

for

Fe(II)/Et_3N-Relay-catalyzed domino reaction of isoxazoles with imidazolium salts in the synthesis of methyl 4-imidazolylpyrrole-2-carboxylates, its ylide and betaine derivatives

Ekaterina E. Galenko[1], Olesya A. Tomashenko[1], Alexander F. Khlebnikov*[,§,1], Mikhail S. Novikov[1], Taras L. Panikorovskii[2]

Address: [1]Institute of Chemistry, Saint Petersburg State University, Universitetskii pr. 26, 198504, St. Petersburg, Russia and [2]Institute of Earth Sciences, Saint Petersburg State University, University Emb. 7/9, 199034, St. Petersburg, Russia

Email: Alexander F. Khlebnikov* - a.khlebnikov@spbu.ru

*Corresponding author

[§]Tel: +7 812 4289344, Fax: +7 812 4286939

Detailed experimental procedures including characterization data for all synthesized compounds, ^{1}H and ^{13}C NMR spectra for all new compounds. Computational details: energies of molecules, transition states and their Cartesian coordinates of atoms. X-ray details

List of Contents

General methods S2

Synthetic details S2

Single crystal X-ray diffraction experiment S12

References S16

NMR spectra S17

Computational details S55

General methods

Melting points were determined on a capillary melting point apparatus Stuart® SMP30. ^{1}H (400 MHz) and ^{13}C (100 MHz) NMR spectra were determined in $CDCl_3$ and DMSO-d_6 with a Bruker AVANCE III 400 spectrometer. Chemical shifts (δ) are reported in parts per million downfield from tetramethylsilane (TMS δ = 0.00). ^{1}H NMR spectra were calibrated according to the residual peak of $CDCl_3$ (7.26 ppm) or DMSO-d_6 (2.50 ppm). For all new compounds ^{13}C{^{1}H} and ^{13}C DEPT135 were recorded and calibrated according to the peak of $CDCl_3$ (77.00 ppm) or DMSO-d_6 (39.51 ppm). Mass spectra were recorded on a Bruker maXis HRMS–ESI–QTOF, with electrospray ionization in positive mode. IR-spectra were recorded on a Bruker FTIR spectrometer Tensor 27 for tablets in KBr, only characteristic absorption is indicated. The single crystal X-ray diffraction experiment was performed on an Agilent Technologies SuperNova diffractometer at 100 K using monochromated Cu*Kα* radiation. Thin-layer chromatography (TLC) was conducted on aluminium sheets with 0.2 mm silica gel (fluorescent indicator, Macherey-Nagel). The isoxazoles **7** [1,2] and imidazolium salts **9** [3] were synthesized by known literature procedures.

Synthetic details

General procedure for the synthesis of 5-methoxycarbonylpyrrol-3-ylimidazolium bromides 1 from isoxazoles 7 and imidazolium bromides 9. Isoxazole **7** (1.2–1.5 mmol) and imidazolium bromide **9** (1.0 mmol) were suspended in MeCN (4 mL), $FeCl_2{\cdot}4H_2O$ (0.06–0.08 mmol, 5 mol % calcd on isoxazole) and Et_3N (3.0 mmol, 3 equiv) were added and the mixture was stirred at 45 °C for 6–7 h (monitored by TLC). The reaction mixture was evaporated to dryness, ethyl acetate was added and the precipitate formed was filtered off and washed with ethyl acetate or ethyl acetate/CH_2Cl_2 mixture. The residue was purified by column chromatography on silica gel (CH_2Cl_2/MeOH 12:1), additionally washed with ethyl acetate or ethyl acetate/CH_2Cl_2 mixture and dried to give the analytically pure compound.

3-(5-Methoxycarbonyl-2,4-diphenyl-1*H*-pyrrol-3-yl)-1-methyl-1*H*-imidazol-3-ium bromide (1a): colorless solid, mp 245–246 °C (dec.) (ethyl acetate), yield 135 mg, 54%, obtained from 5-methoxy-3-phenylisoxazole (**7a**, 150 mg, 0.86 mmol), 1-methyl-3-(2-oxo-2-phenylethyl)-1*H*-imidazol-3-ium bromide (**9a**, 161 mg, 0.57 mmol), $FeCl_2{\cdot}4H_2O$ (8 mg, 0.04 mmol, 5 mol %) and Et_3N (173 mg, 1.71 mmol) according to the general procedure. ^{1}H NMR (DMSO-d$_6$): δ 3.68 (s, 3H), 3.82 (s, 3H), 7.26-7.28 (m, 2H), 7.32-7.33 (m, 3H), 7.36-7.37 (m, 2H), 7.41-7.43 (m, 3H), 7.77-7.79 (m, 1H), 7.85-7.86 (m, 1H), 9.33 (m, 1H), 12.93 (br s, 1H). ^{13}C NMR (DMSO-d$_6$): δ 36.2 (CH_3), 51.4 (CH_3), 116.3 (C), 117.8 (C), 124.1 (CH), 125.8 (CH), 127.6 (CH), 127.78 (CH), 127.84 (CH), 128.1 (C), 128.9 (CH), 129.0 (CH), 129.7 (CH), 130.5 (C), 131.8 (C), 138.8 (CH), 138.8 (C), 160.2 (C). ESI/HRMS (m/z): found 358.1545 calcd for $C_{22}H_{20}N_3O_2$ [M – Br]$^+$, found 358.1550. IR (KBr, cm^{-1}): ν 3402, 3049, 1702.

3-(2-(4-Chlorophenyl)-5-methoxycarbonyl-4-phenyl-1*H*-pyrrol-3-yl)-1-methyl-1*H*-imidazol-3-ium bromide (1b): colorless solid, mp 242–243 °C (dec.) (ethyl acetate), yield 204 mg, 54%, obtained from 5-methoxy-3-phenylisoxazole (**7a**, 175 mg, 1 mmol), 3-(2-(4-chlorophenyl)-2-oxoethyl)-1-methyl-1*H*-imidazol-3-ium bromide (**9b**, 253 mg, 0.8 mmol), $FeCl_2{\cdot}4H_2O$ (10 mg, 0.05 mmol, 5 mol %) and Et_3N (242 mg, 2.4 mmol) according to the general procedure. ^{1}H NMR (DMSO-d$_6$): δ 3.68 (s, 3H), 3.83 (s, 3H), 7.25-7.28 (m, 2H), 7.31-7.32 (m, 3H), 7.37-7.40 (m, 2H), 7.48-7.50 (m, 2H), 7.81-7.82 (m, 1H), 7.83-7.84 (m, 1H), 9.35-9.36 (m, 1H), 13.03 (br s, 1H). ^{13}C NMR (DMSO-d$_6$): δ 36.2 (CH_3), 51.5 (CH_3), 116.5 (C), 118.1(C), 124.2 (CH), 125.6 (CH), 126.6 (C), 127.8 (CH), 127.9 (CH), 128.0 (C), 128.9 (CH), 129.5 (CH), 129.7 (CH), 130.4 (C), 130.6 (C), 133.8 (C), 138.8 (CH), 160.2 (C). ESI/HRMS

(m/z): 392.1160 calcd for $C_{22}H_{19}ClN_3O_2$ [M – Br]$^+$, found 392.1168. IR (KBr, cm^{-1}): ν 3389, 3042, 1706.

3-(5-Methoxycarbonyl-2-(4-nitrophenyl)-4-phenyl-1*H*-pyrrol-3-yl)-1-methyl-1*H*-imidazol-3-ium bromide (1c): light yellow solid, mp 248–250 °C (dec.) (ethyl acetate), yield 244 mg, 63%, obtained from 5-methoxy-3-phenylisoxazole (**7a**, 175 mg, 1 mmol), 1-methyl-3-(2-(4-nitrophenyl)-2-oxoethyl)-1*H*-imidazol-3-ium bromide (**9c**, 261 mg, 0.8 mmol), $FeCl_2·4H_2O$ (10 mg, 0.05 mmol, 5 mol %) and Et_3N (242 mg, 2.4 mmol) according to the general procedure. ^{1}H NMR (DMSO-d$_6$): δ 3.71 (s, 3H), 3.85 (s, 3H), 7.27-7.29 (m, 2H), 7.33-7.35 (m, 3H), 7.62 (d, *J* = 8.9 Hz, 2H), 7.83-7.85 (m, 2H), 8.25 (d, *J* = 8.9 Hz, 2H), 9.39-9.40 (m, 1H), 13.30 (br s, 1H). ^{13}C NMR (DMSO-d$_6$): δ 36.3 (CH_3), 51.7 (CH_3), 117.6 (C), 119.3 (C), 124.0 (CH), 124.5 (CH), 125.4 (CH), 127.9 (CH), 128.0(CH), 128.3 (C), 128.7 (CH), 129.3 (C), 129.7 (CH), 130.2 (C), 134.1 (C), 138.8 (CH), 147.1 (C), 160.2 (C). ESI/HRMS (m/z): 403.1401 calcd for $C_{22}H_{19}N_4O_4$ [M – Br]$^+$, found 403.1410. IR (KBr, cm^{-1}): ν 3448, 3007, 1718.

3-(2-(3-bromophenyl)-4-(4-bromophenyl)-5-methoxycarbonyl-1*H*-pyrrol-3-yl)-1-methyl-1*H*-imidazol-3-ium bromide (1d): colorless solid, mp 240–242 °C (dec.) (ethyl acetate), yield 314 mg, 66%, obtained from 3-(4-bromophenyl)-5-methoxyisoxazole (**7b**, 254 mg, 1 mmol), 3-(2-(3-bromophenyl)-2-oxoethyl)-1-methyl-1*H*-imidazol-1-ium bromide (**9d**, 288 mg, 0.8 mmol), $FeCl_2·4H_2O$ (10 mg, 0.05 mmol, 5 mol %) and Et_3N (242 mg, 2.4 mmol) according to the general procedure. ^{1}H NMR (DMSO-d$_6$): δ 3.71 (s, 3H), 3.85 (s, 3H), 7.15-7.17 (m, 1H), 7.21-7.25 (m, 2H), 7.34 (t, *J* = 7.9 Hz, 1H), 7.51-7.54 (m, 2H), 7.60-7.63 (m, 1H), 7.75 (t, *J* = 1.7 Hz, 1H), 7.84-7.85 (m, 1H), 7.86-7.87 (m, 1H), 9.35-9.36 (m, 1H), 13.12 (br s, 1H). ^{13}C NMR (DMSO-d$_6$): δ 36.3 (CH_3), 51.7 (CH_3), 116.5 (C), 118.4 (C), 121.5 (C), 122.1 (C), 124.4 (CH), 125.5 (CH), 126.2 (CH), 126.7 (C), 129.6 (C), 129.8 (C), 130.2 (C), 130.6 (CH), 130.9 (CH), 131.0 (CH), 131.8 (CH), 131.9 (CH), 138.9 (CH), 160.9 (C). ESI/HRMS (m/z): 513.9760 calcd for $C_{22}H_{18}Br_2N_3O_2$ [M – Br]$^+$, found 513.9770. IR (KBr, cm^{-1}): ν 3345, 3033, 1718, 1705.

3-(5-Methoxycarbonyl-2,4-diphenyl-1*H*-pyrrol-3-yl)-1-phenyl-1*H*-imidazol-3-ium bromide (1e): colorless solid, mp 254–255°C (dec.) (ethyl acetate), yield 339 mg, 68%, obtained from 5-methoxy-3-phenylisoxazole (**7a**, 210 mg, 1.2 mmol), 3-(2-oxo-2-phenylethyl)-1-phenyl-1*H*-imidazol-3-ium bromide (**9e**, 344 mg, 1.0 mmol), $FeCl_2·4H_2O$ (12 mg, 0.06 mmol, 5 mol %) and Et_3N (305 mg, 3.0 mmol) according to the general procedure. ^{1}H NMR (DMSO-d$_6$): δ 3.71 (s, 3H), 7.30-7.34 (m, 5H), 7.42-7.50 (m, 5H), 7.57-7.71 (m, 5H), 8.13-8.14 (m, 1H), 8.48-8.49 (m, 1H), 10.15-10.16 (m, 1H), 13.04 (br s, 1H). ^{13}C NMR (DMSO-d$_6$): δ 51.5 (CH_3), 116.2 (C), 118.0 (C), 121.8 (CH), 121.9 (CH), 126.7 (CH), 127.7 (C), 127.8 (CH), 127.9 (CH), 127.9 (CH), 128.1 (C), 128.9 (CH), 129.2 (CH), 129.7 (CH), 130.2 (CH), 130.2 (CH), 130.4 (C), 131.8 (C), 134.1 (C), 137.2 (CH), 160.3 (C). ESI/HRMS (m/z): 420.1707 calcd for $C_{27}H_{22}N_3O_2$ [M – Br]$^+$, found 420.1720. IR (KBr, cm^{-1}): ν 3452, 3031, 1708.

3-(5-Methoxycarbonyl-2-(4-methoxyphenyl)-4-phenyl-1*H*-pyrrol-3-yl)-1-phenyl-1*H*-imidazol-3-ium bromide (1f): colorless solid, mp 245–258°C (dec.) (ethyl acetate), yield 302 mg, 71%, obtained from 5-methoxy-3-phenylisoxazole (**7a**, 175 mg, 1.0 mmol), 3-(2-(4-methoxyphenyl)-2-oxoethyl)-1-phenyl-1*H*-imidazol-3-ium bromide (**9f**, 299 mg, 0.8 mmol), $FeCl_2·4H_2O$ (10 mg, 0.05 mmol, 5 mol %) and Et_3N (242 mg, 2.4 mmol) according to the general procedure. ^{1}H NMR (DMSO-d$_6$): δ 3.70 (s, 1H), 3.77 (s, 3H), 7.00 (d, *J* = 8.2 Hz, 2H), 7.31-7.34 (m, 5H), 7.42 (d, *J* = 8.2 Hz, 2H), 7.59-7.71 (m. 5H), 8.12 (s, 1H), 8.48 (s, 1H), 10.13 (s, 1H), 12.90 (br s, 1H). ^{13}C NMR (DMSO-d$_6$): δ 51.5 (CH_3), 55.3 (CH_3), 114.4 (CH), 115.6 (C), 117.4 (C), 120.0 (C), 121.8 (CH), 121.8 (CH), 126.8 (CH), 127.85 (CH), 127.90 (CH), 128.1 (C), 129.2 (CH), 129.7 (CH), 130.2 (CH), 130.6 (C), 130.7 (CH), 131.9 (C), 134.1 (C), 137.2 (CH), 159.8 (C), 160.3 (C). ESI/HRMS (m/z): 450.1812 calcd for $C_{28}H_{24}N_3O_3$ [M – Br]$^+$, found 450.1825. IR (KBr, cm^{-1}): ν 3422, 3048, 2952, 1719.

3-(2-(4-Bromophenyl)-5-methoxycarbonyl-4-phenyl-1*H*-pyrrol-3-yl)1-phenyl-1*H*-imidazol-3-ium bromide (1g): colorless solid, mp 201–202 °C (water), yield 379 mg, 57%, obtained from 5-methoxy-3-phenylisoxazole (**7a**, 241 mg, 1.4 mmol), 3-(2-(4-bromophenyl)-2-oxoethyl)-1-phenyl-1*H*-imidazol-3-ium bromide (**9g**, 485 mg, 1.2 mmol), $FeCl_2 \cdot 4H_2O$ (14 mg, 0.75 mmol, 5 mol %) and NEt_3 (348 mg, 3.5 mmol) according to the general procedure. ^{1}H NMR (DMSO-d$_6$): δ 3.71 (s, 3H), 7.31-7.35 (m, 5H), 7.42-7.44 (m, 2H), 7.57-7.66 (m, 5H), 7.69-7.72 (m, 2H), 8.10-8.11 (m, 1H), 8.48-8.49 (m, 1H), 10.14 (s, 1H), 13.14 (s, 1H). ^{13}C NMR (DMSO-d$_6$): δ 51.6 (CH_3), 116.4 (C), 118.3 (C), 121.8 (CH), 121.9 (CH), 122.6 (C), 126.5 (CH), 126.9 (C), 127.9 (CH), 127.9 (CH), 128.1 (C), 129.7 (CH), 129.8 (CH), 130.2 (CH), 130.2 (CH), 130.3 (C), 130.5 (C), 131.9 (CH), 134.1 (C), 137.2 (CH), 160.2 (C). ESI/HRMS (m/z): 500.0793 calcd for $C_{27}H_{21}BrN_3O_2$ $[M – Br]^+$, found 500.0822. IR (KBr, cm^{-1}): ν 3399, 3066, 1699, 1553.

3-(5-Methoxycarbonyl-4-methyl-2-phenyl-1*H*-pyrrol-3-yl)-1-phenyl-1*H*-imidazol-3-ium bromide (1h): colorless solid, mp 240–242°C (dec.) (ethyl acetate), yield 235 mg, 54%, obtained from 5-methoxy-3-methylisoxazole (**7c**, 170 mg, 1.5 mmol), 3-(2-oxo-2-phenylethyl)-1-phenyl-1*H*-imidazol-3-ium bromide (**9e**, 344 mg, 1.0 mmol), $FeCl_2 \cdot 4H_2O$ (12 mg, 0.06 mmol, 5 mol %) and Et_3N (305 mg, 3.0 mmol) according to the general procedure. ^{1}H NMR (DMSO-d$_6$): δ 2.28 (s, 3H), 3.87 (s, 3H), 7.36-7.42 (m, 5H), 7.62-7.64 (m. 1H), 7.67-7.71 (m, 2H), 7.86-7.90 (m, 2H), 8.14-8.15 (m, 1H), 8.62-8.63 (m, 1H), 10.17-10.18 (m, 1H), 12.65 (br s, 1H). ^{13}C NMR (DMSO-d$_6$): δ 9.0 (CH_3), 51.5 (CH_3), 117.1 (C), 118.1 (C), 122.0 (CH), 122.0 (CH), 123.8 (C), 125.8 (CH), 127.6 (CH), 127.9 (C), 128.8 (CH), 128.9 (CH), 130.0 (CH), 130.1 (CH), 131.3 (C), 134.5 (C), 136.9 (CH), 160.9 (C). ESI/HRMS (m/z): 358.1550 calcd for $C_{22}H_{20}N_3O_2$ $[M – Br]^+$, found 358.1566. IR (KBr, cm^{-1}): ν 3011, 2914, 1709.

3-(5-Methoxycarbonyl-2-(4-methoxyphenyl)-4-methyl-1*H*-pyrrol-3-yl)-1-phenyl-1*H*-imidazol-3-ium bromide (1i): colorless solid, mp 237-238°C (dec.) (ethyl acetate), yield 276 mg, 59%, obtained from 5-methoxy-3-methylisoxazole (**7c**, 170 mg, 1.5 mmol), 3-(2-(4-methoxyphenyl)-2-oxoethyl)-1-phenyl-1*H*-imidazol-3-ium bromide (**9f**, 344 mg, 1.0 mmol), $FeCl_2 \cdot 4H_2O$ (12 mg, 0.06 mmol, 5 mol %) and Et_3N (305 mg, 3.0 mmol) according to the general procedure. ^{1}H NMR (DMSO-d$_6$): δ 2.26 (s, 3H), 3.76 (s, 3H), 3.86 (s, 3H), 6.94-6.98 (m, 2H), 7.31-7.35 (m, 2H), 7.60-7.64 (m, 1H), 7.67-7.71 (m, 2H), 7.89-7.92 (m, 2H), 8.12-8.13 (m, 1H), 8.64-8.65 (m, 1H), 10.19-10.20 (m, 1H), 12.51 (br s, 1H). ^{13}C NMR (DMSO-d$_6$): δ 9.1 (CH_3), 51.4 (CH_3), 55.2 (CH_3), 114.3 (CH), 116.5 (C), 117.5 (C), 120.2 (C), 122.0 (CH), 122.0 (CH), 123.8 (C), 125.9 (CH), 129.0 (CH), 130.0 (CH), 130.1 (CH), 131.4 (C), 134.5 (C), 136.9 (CH), 159.6 (C), 160.9 (C). ESI/HRMS (m/z): 388.1656 calcd for $C_{23}H_{22}N_3O_3$ $[M – Br]^+$, found 388.1672. IR (KBr, cm^{-1}): ν 3412, 3348, 3042, 1711.

1-Benzyl-3-(5-methoxycarbonyl-2,4-diphenyl-1*H*-pyrrol-3-yl)-1*H*-imidazol-3-ium bromide (1j): colorless solid, mp 230–232°C (dec.) (ethyl acetate), yield 279 mg, 51%, obtained from 5-methoxy-3-phenylisoxazole (**7a**, 220 mg, 1.3 mmol), 1-benzyl-3-(2-oxo-2-phenylethyl)-1*H*-imidazol-3-ium bromide (**9h**, 381 mg, 1.1 mmol), $FeCl_2 \cdot 4H_2O$ (13 mg, 0.06 mmol, 5 mol %) and Et_3N (321 mg, 3.2 mmol) according to the general procedure. ^{1}H NMR (DMSO-d$_6$): δ 3.69 (s, 3H), 5.42 (s, 2H), 7.03-7.05 (m, 2H), 7.24-43 (m, 13H), 7.86-7.88 (m, 2H), 9.59-9.60 (m, 1H), 12.98 (br s, 1H). ^{13}C NMR (DMSO-d$_6$): δ 51.5 (CH_3), 52.1 (CH_2), 116.4 (C), 117.8 (C), 123.2 (CH), 126.3 (CH), 127.3 (CH), 127.6 (CH), 127.7 (C), 127.8 (CH), 127.9 (CH), 128.1 (C), 128.5 (CH), 128.8 (CH), 128.9 (CH), 129.1 (CH), 129.6 (CH), 130.5 (C), 131.7 (C), 134.7 (C), 138.8 (CH), 160.2 (C). ESI/HRMS (m/z): 434.1863 calcd for $C_{28}H_{24}N_3O_2$ $[M – Br]^+$, found 434.1854. IR (KBr, cm^{-1}): ν 3066, 1697.

1-Benzyl-3-(5-methoxycarbonyl-2-(4-methoxyphenyl)-4-phenyl-1*H*-pyrrol-3-yl)-1*H*-imidazol-3-ium bromide (1k): colorless solid, mp 244–246°C (dec.) (ethyl acetate), yield 279 mg, 51%, obtained from 5-methoxy-3-phenylisoxazole (**7a**, 175 mg, 1.0 mmol), 1-benzyl-3-(2-

(4-methoxyphenyl)-2-oxoethyl)-1*H*-imidazol-3-ium bromide (**9i**, 299 mg, 0.8 mmol), $FeCl_2 \cdot 4H_2O$ (10 mg, 0.05 mmol, 5 mol %) and Et_3N (242 mg, 2.4 mmol) according to the general procedure. ^1H NMR (DMSO-d_6): δ 3.68 (s, 3H), 3.79 (s, 3H), 5.43 (s, 2H), 6.92-6.95 (m, 2H), 7.05-7.09 (m, 2H), 7.23-7.34 (m, 7H), 7.37-7.40 (m, 3H), 7.85-7.86 (m, 1H), 7.88-7.89 (m, 1H), 9.61-9.62 (m, 1H), 12.83 (br s, 1H). ^{13}C NMR (DMSO-d_6): δ 51.4 (CH_3), 52.1 (CH_2), 55.3 (CH_3), 114.3 (CH), 115.9 (C), 117.2 (C), 120.0 (C), 123.2 (CH), 126.3 (CH), 127.3 (CH), 127.7 (CH), 127.8 (CH), 128.1 (C), 128.5 (CH), 128.9 (CH), 129.1 (CH), 129.6 (CH), 130.7 (C), 131.8 (C), 134.8 (C), 138.8 (CH), 159.8 (C), 160.3 (C). ESI/HRMS (m/z): 464.1969 calcd for $C_{29}H_{26}N_3O_3$ $[M – Br]^+$, found 464.1974. IR (KBr, cm^{-1}): ν 3407, 3068, 1696.

1-Benzyl-3-(2-(4-fluorophenyl)-5-methoxycarbonyl-4-phenyl-1*H*-pyrrol-3-yl)-1*H*-imidazol-3-ium bromide (1l): colorless solid, mp 250–251 °C (dec., ethyl acetate), yield 305 mg, 55%, obtained from 5-methoxy-3-phenylisoxazole (**7a**, 230 mg, 1.3 mmol), 1-benzyl-3-(2-(4-fluorophenyl)-2-oxoethyl-1*H*-imidazol-3-ium bromide (**9j**, 411 mg, 1.1 mmol), $FeCl_2 \cdot 4H_2O$ (14 mg, 0.75 mmol, 5 mol %) and NEt_3 (333 mg, 3.3 mmol) according to the general procedure. ^1H NMR (DMSO-d_6): δ 3.69 (s, 3H), 5.42 (s, 2H), 7.05-7.06 (m, 2H), 7.24-7.38 (m, 10H), 7.42-7.46 (m, 2H), 7.86-7.89 (m, 2H), 9.59 (s, 1H), 13.01 (s, 1H). ^{13}C NMR (DMSO-d_6): δ 51.5 (CH_3), 52.1 (CH_2), 115.9 (d, J = 21.8 Hz, CH), 116.4 (C), 117.7 (C), 123.2 (CH), 124.3 (d, J = 2.9 Hz, C), 126.1 (CH), 127.3 (CH), 127.8 (CH), 127.9 (CH), 128.0 (C), 128.6 (CH), 128.9 (CH), 129.6 (CH), 130.1 (d, J = 8.1 Hz, CH), 130.5 (C), 130.8 (C), 134.7 (C), 138.8 (CH), 160.2 (C), 162.3 (d, J = 246.5 Hz, C). ^{19}F NMR (DMSO-d_6): -111.8. ESI/HRMS (m/z): 452.1769 calcd for $C_{22}H_{19}ClN_3O_2$ $[M – Br]^+$, found 452.1788. IR (KBr, cm^{-1}): ν 3377, 3065, 1694.

1-Benzyl-3-(2-(4-chlorophenyl)-5-methoxycarbonyl-4-phenyl-1*H*-pyrrol-3-yl)-1*H*-imidazol-3-ium bromide (1m): colorless solid, mp 249–251°C (dec.) (ethyl acetate), yield 317 mg, 72%, obtained from 5-methoxy-3-phenylisoxazole (**7a**, 175 mg, 1.0 mmol), 1-benzyl-3-(2-(4-chlorophenyl)-2-oxoethyl)-1*H*-imidazol-3-ium bromide (**9k**, 313 mg, 0.8 mmol), $FeCl_2 \cdot 4H_2O$ (10 mg, 0.05 mmol, 5 mol %) and Et_3N (245 mg, 2.4 mmol) according to the general procedure. ^1H NMR (DMSO-d_6): δ 3.69 (s, 3H), 5.43 (s, 2H), 7.05-7.07 (m, 2H), 7.23-7.40 (m, 10H), 7.45-7.48 (m, 2H), 7.85-7.86 (m, 1H), 7.89-7.90 (m, 1H), 9.59-9.60 (m, 1H), 13.06 (br s, 1H). ^{13}C NMR (DMSO-d_6): δ 51.5 (CH_3), 52.1 (CH_2), 116.6 (C), 118.0 (C), 123.3 (CH), 126.0 (CH), 126.6 (C), 127.3 (CH), 127.8 (CH), 127.9 (CH), 128.0 (C), 128.5 (CH), 128.9 (CH), 128.9 (CH), 129.5 (CH), 129.6 (CH), 130.4 (C), 130.5 (C), 133.8 (C), 134.7 (C), 138.8 (CH), 160.2 (C). ESI/HRMS (m/z): 468.1473 calcd for $C_{28}H_{23}ClN_3O_2$ $[M – Br]^+$, found 468.1470. IR (KBr, cm^{-1}): ν 3402, 3081, 1700.

1-Benzyl-3-(5-methoxycarbonyl-4-methyl-2-phenyl-1*H*-pyrrol-3-yl)-1*H*-imidazol-3-ium bromide (1n): colorless solid, mp 220–223°C (dec.) (ethyl acetate), yield 374 mg, 47%, obtained from 5-methoxy-3-methylisoxazole (**7c**, 300 mg, 2.7 mmol), 1-benzyl-3-(2-oxo-2-phenylethyl)-1*H*-imidazol-3-ium bromide (**9h**, 633 mg, 1.8 mmol), $FeCl_2 \cdot 4H_2O$ (27 mg, 0.14 mmol, 5 mol %) and Et_3N (536 mg, 5.3 mmol) according to the general procedure. ^1H NMR (DMSO-d_6): δ 2.19 (s, 3H), 3.86 (s, 3H), 5.51 (s, 2H), 7.23-7.25 (m, 2H), 7.33-7.46 (m, 8H), 7.97-7.98 (m, 1H), 8.06-8.07 (m, 1H), 9.64-9.65 (m, 1H), 12.62 (br s, 1H). ^{13}C NMR (DMSO-d_6): δ 9.0 (CH_3), 51.5 (CH_3), 52.2 (CH_2), 117.2 (C), 118.0 (C), 123.4 (CH), 123.5 (C), 125.5 (CH), 127.3 (CH), 127.8 (CH), 127.9 (C), 128.7 (CH), 128.8 (CH), 128.9 (CH), 129.0 (CH), 131.3 (C), 134.8 (C), 138.4 (CH), 160.8 (C). ESI/HRMS (m/z): 372.1707 calcd for $C_{23}H_{22}N_3O_2$ $[M – Br]^+$, found 372.1712. IR (KBr, cm^{-1}): ν 3343, 3046, 1703.

1-Benzyl-3-(2-(4-chlorophenyl)-5-methoxycarbonyl-4-methyl-1*H*-pyrrol-3-yl)-1*H*-imidazol-3-ium bromide (1o): colorless solid, mp 229–231°C (dec.) (ethyl-acetate), yield 335 mg, 69%,

obtained from 5-methoxy-3-methylisoxazole (**7c**, (170 mg, 1.5 mmol), 1-benzyl-3-(2-(4-chlorophenyl)-2-oxoethyl)-1*H*-imidazol-3-ium bromide (**9k**, 392 mg, 1.0 mmol), $FeCl_2{\cdot}4H_2O$ (15 mg, 0.08 mmol, 5 mol %) and Et_3N (300 mg, 3.0 mmol) according to the general procedure. ^{1}H NMR (DMSO-d_6): δ 2.19 (s, 3H), 3.86 (s, 3H), 5.52 (s, 2H), 7.22-7.25 (m, 2H), 7.34-7.37 (m, 2H), 7.38-7.46 (m, 5H), 7.97-7.98 (m, 1H), 8.09-8.10 (m, 1H), 9.63-9.64 (m, 1H), 12.70 (br s, 1H). ^{13}C NMR (DMSO-d_6): δ 9.0 (CH_3), 51.5 (CH_3), 52.2 (CH_2), 117.5 (C), 118.3 (C), 123.4 (C), 123.5 (CH), 125.3 (CH), 126.7 (C), 127.9 (CH), 128.7 (CH), 128.8 (CH), 128.9 (CH), 129.2 (CH), 130.1 (C), 133.5 (C), 134.8 (C), 138.3 (CH), 160.8 (C). ESI/HRMS (m/z): 406.1317 calcd for $C_{23}H_{21}ClN_3O_2$ $[M – Br]^+$, found 406.1317. IR (KBr, cm^{-1}): ν 3420, 3103, 1702.

General procedure for debenzylation of 1-benzyl-3-pyrrol-3-yl-1*H*-imidazol-3-ium bromides 1j,k,n. 1-Benzyl-1*H*-imidazol-3-ium bromide **1** (100 mg) was dissolved in MeOH (10 mL), Pd/C (10 mg, 10% wt) and ammonium formate (10 equiv) were added. The suspension was stirred under reflux for 1 h (monitored by TLC). The reaction mixture was filtered to remove Pd/C, MeOH was evaporated under reduced pressure, water was added to the residue and the product was filtered, washed with water and dried to give the analytically pure compound.

Methyl 4-(1*H*-imidazol-1-yl)-3,5-diphenyl-1*H*-pyrrole-2-carboxylate (12a): colorless solid, mp 239-241°C (dec.) (water), yield 128 mg, 90%, obtained from 1-benzyl-3-(5-(methoxycarbonyl)-2,4-diphenyl-1*H*-pyrrol-3-yl)-1*H*-imidazol-3-ium bromide (**1j**, 220 mg, 0.43 mmol), Pd/C (22 mg, 10% wt) and ammonium formate (270 mg, 4.3 mmol) according to the general procedure. ^{1}H NMR (DMSO-d_6): δ 3.66 (s, 3H), 6.89-6.90 (m, 1H), 7.10-7.11 (m, 1H), 7.21-7.26 (m, 7H), 7.30-7.33 (m, 3H), 7.51-7.52 (m, 1H), 12.49 (br s, 1H). ^{13}C NMR (DMSO-d_6): δ 51.2 (CH_3), 117.3 (C), 119.1 (C), 122.5 (CH), 126.9 (CH), 127.1 (CH), 127.4 (CH), 128.2 (CH), 128.5 (CH), 128.69 (CH), 128.70 (C), 128.9 (C), 129.7 (CH), 131.1 (C), 131.8 (C), 139.1 (CH), 160.5 (C). ESI/HRMS (m/z): 344.1394 calcd for $C_{21}H_{18}N_3O_2$ $[M + H]^+$, found 344.1401. IR (KBr, cm^{-1}): ν 3124, 2951, 1688.

Methyl 4-(1*H*-imidazol-1-yl)-5-(4-methoxyphenyl)-3-phenyl-1*H*-pyrrole-2-carboxylate (12b): colorless solid, mp 240-241 °C (dec.) (water), yield 138 mg, 94%, obtained from 1-benzyl-3-(5-(methoxycarbonyl)-2-(4-methoxyphenyl)-4-phenyl-1*H*-pyrrol-3-yl)-1*H*-imidazol-3-ium bromide (**1k**, 220 mg, 0.40 mmol), Pd/C (22 mg, 10% wt) and ammonium formate (253 mg, 4.0 mmol) according to the general procedure. ^{1}H NMR (DMSO-d_6): δ 3.65 (s, 3H), 3.74 (s, 3H), 6.87-6.90 (m, 3H), 7.09-7.10 (m, 1H), 7.17-7.24 (m, 7H), 7.50-7.51 (m, 1H), 12.29 (br s, 1H). ^{13}C NMR (DMSO-d_6): δ 51.1 (CH_3), 55.2 (CH_3), 114.0 (CH), 116.6 (C), 118.5 (C), 121.3 (C), 122.5 (CH), 127.1 (CH), 127.4 (CH), 128.3 (CH), 128.7 (CH), 128.8 (C), 129.7 (CH), 131.3 (C), 131.9 (C), 139.1 (CH), 159.2 (C), 160.6 (C). ESI/HRMS (m/z): 374.1499 calcd for $C_{22}H_{20}N_3O_2$ $[M + H]^+$, found 374.1496. IR (KBr, cm^{-1}): ν 3119, 2950, 2841, 2726, 1723, 1691.

Methyl 4-(1*H*-imidazol-1-yl)-3-methyl-5-phenyl-1*H*-pyrrole-2-carboxylate (12c): colorless solid, mp 265-267 °C (dec.) (water), yield 116 mg, 88%, obtained from 1-benzyl-3-(5-(methoxycarbonyl)-4-methyl-2-phenyl-1*H*-pyrrol-3-yl)-1*H*-imidazol-3-ium bromide (**1n**, 220 mg, 0.49 mmol), Pd/C (22 mg, 10% wt) and ammonium formate (306 mg, 4.9 mmol) according to the general procedure. ^{1}H NMR (DMSO-d_6): δ 2.07 (s, 3H), 3.84 (s, 3H), 7.08-7.09 (m, 1H), 7.17-7.18 (m, 1H), 7.20-7.23 (m, 2H), 7.27-7.31 (m, 3H), 7.57-7.58 (m, 1H), 12.04 (br s, 1H).^{13}C NMR (DMSO-d_6): δ 8.8 (CH_3), 50.9 (CH_3), 117.4 (C), 119.8 (C), 121.5 (CH), 124.1 (C), 126.5 (CH), 127.8 (CH), 128.2 (CH), 128.9 (CH), 129.0 (C), 130.5 (C), 138.5 (CH), 160.9 (C). ESI/HRMS (m/z): 282.1237 calcd for $C_{16}H_{16}N_3O_2$ $[M + H]^+$, found 282.1232. IR (KBr, cm^{-1}): ν 3136, 3008, 2778, 1704.

Methyl 5-(4-fluorophenyl)-4-(1*H*-imidazol-1-yl)-3-phenyl-1*H*-pyrrole-2-carboxylate (12d). Suspension of 3-(1-benzyl-1*H*-imidazol-3-ium-3-yl)-2-(4-fluorophenyl)-5-(methoxycarbonyl)-4-

phenylpyrrol-1-ide (**2h**, 45 mg, 0.0997 mmol) and Pd/C (4.5 mg, 10 wt%) in dry methanol (5 mL) was stirred at rt for 5 h under pressure of balloon with hydrogen. Then reaction mixture was filtered from catalyst and evaporated to dryness. Colorless solid, mp 226–227 °C, yield 35 mg, 99%. ^{1}H NMR (DMSO-d$_6$): δ 3.31 (s, 3H), 6.89 (s, 1H), 7.11 (s, 1H), 7.14-7.32 (m, 9H), 7.52 (s, 1H), 12.53 (s, 1H). ^{13}C NMR (DMSO-d$_6$): δ 51.17 (CH$_3$), 115.5 (d, *J* = 21.7 Hz, CH), 117.3 (C) 119.1 (C), 122.4 (CH), 125.5 (d, *J* = 3 Hz, C), 127.2 (CH), 127.4 (CH), 128.6 (C), 128.8 (CH), 129.2 (d, *J* = 8.2 Hz, CH), 129.7 (CH), 130.3 (C), 131.7 (C), 139.1 (CH), 160.5 (C), 161.9 (d, *J* = 247.5 Hz, C). ESI/HRMS (m/z): 362.1299 calcd for $C_{21}H_{17}FN_3O_2$ [M + H]$^+$, found 362.1311. IR (KBr, cm^{-1}): ν 3121, 1716, 1499.

General procedure for the synthesis of pyrrolydes 2 from 5-methoxycarbonylpyrrol-3-ylimidazolium bromides 1. A suspension of 3-(1*H*-pyrrol)-1*H*-imidazol-3-ium bromide **1** (1 mmol) in aq solution of KOH (2 mmol, 2 equiv, 5 mL H_2O) was sonicated for 5 min and then vigorously stirred for 12 h. The precipitate was filtered, washed with water (2–3 mL) and dried to give the analytically pure compound.

2-(Methoxycarbonyl)-4-(1-methyl-1*H*-imidazol-3-ium-3-yl)-3,5-diphenylpyrrol-1-ide (2a): colorless solid, mp 237–238 °C (dec.), yield 188 mg, 71%, obtained from 3-(5-(methoxycarbonyl)-2,4-dipheny-1*H*-pyrrol-3-yl)-1-methyl-1*H*-imidazol-3-ium bromide (**1a**, 323 mg, 0.737 mmol) and aq solution of KOH (83 mg, 1.482 mmol, 4 mL H_2O) according to the general procedure. ^{1}H NMR (DMSO-d$_6$): δ 3.51 (s, 3H), 3.81 (s, 3H), 7.03-7.21 (m, 10H), 7.69-7.72 (m. 2H), 9.11 (s, 1H). ^{13}C NMR (DMSO-d$_6$): δ 35.7 (CH$_3$), 49.4 (CH$_3$), 115.2 (C), 123.3 (CH), 124.6 (CH), 124.9 (CH), 125.2 (CH), 125.8 (C), 126.3 (CH), 127.0 (CH), 128.0 (CH), 128.0 (C), 129.6 (CH), 135.5 (C), 135.8 (C), 137.1 (C), 137.9 (CH), 165.0 (C). ESI/HRMS (m/z): 358.1550 calcd for $C_{22}H_{20}N_3O_2$ [M + H]$^+$, found 358.1566. IR (KBr, cm^{-1}): ν 3528, 3144, 3059, 1676.

2-(4-Chlorophenyl)-5-methoxycarbonyl-3-(1-methyl-1*H*-imidazol-3-ium-3-yl)-4-phenylpyrrol-1-ide (2b): colorless solid, mp 253 °C, yield 72 mg, 86%, obtained from 3-(2-(4-chlorophenyl)-5-(methoxycarbonyl)-4-phenyl-1*H*-pyrrol-3-yl)-1-methyl-1*H*-imidazol-3-ium bromide (**1b**, 100 mg, 0.212 mmol) and aq solution of KOH (24 mg, 0.423 mmol, 2 equiv, 3 mL H_2O) according to the general procedure. ^{1}H NMR (DMSO-d$_6$): δ 3.51 (s, 3H), 3.81 (s, 3H), 7.07-7.24 (m, 9H), 7.70 (s, 1H), 7.73 (s, 1H), 9.12 (s, 1H). ^{13}C NMR (DMSO-d$_6$): δ 35.8 (CH$_3$), 49.5 (CH$_3$), 115.4 (C), 123.5 (CH), 125.3 (CH), 126.1 (CH), 126.2 (C), 126.4 (CH), 127.1 (CH), 128.0 (CH), 128.2 (C), 128.9 (C), 129.6 (CH), 134.2 (C), 135.6 (C), 135.9 (C), 138.0 (CH), 164.9 (C). ESI/HRMS (m/z): 392.1160 calcd for $C_{22}H_{19}ClN_3O_2$ [M + H]$^+$, found 392.1165. IR (KBr, cm^{-1}): ν 3125, 3068, 2942, 1678.

2-Methoxycarbonyl-4-(1-methyl-1*H*-imidazol-3-ium-3-yl)-5-(4-nitrophenyl)-3-phenylpyrrol-1-ide (2c): orange solid, mp 289–291 °C (dec.), yield 377 mg, 98%, obtained from 3-(5-(methoxycarbonyl)-2-(4-nitrophenyl)-4-phenyl-1*H*-pyrrol-3-yl)-1-methyl-1*H*-imidazol-3-ium bromide (**1c**, 460 mg, 0.953 mmol) and aq solution of KOH (107 mg, 1.911 mmol, 5 mL H_2O) according to the general procedure. ^{1}H NMR (DMSO-d$_6$): 3.55 (s, 3H), 3.85 (s, 3H), 7.12-7.21 (m, 5H), 7.36 (d, *J* = 9.0 Hz, 2H), 7.78-7.80 (m, 2H), 8.03 (d, *J* = 9.0 Hz, 2H), 9.23 (s, 1H). ^{13}C NMR (DMSO-d$_6$): δ 35.9 (CH$_3$), 49.7 (CH$_3$), 117.3 (C), 123.9 (CH), 123.9 (CH), 124.3 (CH), 125.6 (CH), 125.8 (CH), 127.2 (CH), 128.6 (C), 129.1 (C), 129.5 (CH), 133.0 (C), 135.0 (C), 138.0 (CH), 143.5 (C), 143.7 (C), 164.8 (C). ESI/HRMS (m/z): 403.1401 calcd for $C_{22}H_{19}N_4O_4$ [M + H]$^+$, found 403.1419. IR (KBr, cm^{-1}): ν 3431, 3132, 1685, 1589.

2-(3-Bromophenyl)-4-(4-bromophenyl)-5-methoxycarbonyl-3-(1-methyl-1*H*-imidazol-3-ium-3-yl)pyrrol-1-ide (2d): colorless solid, mp 265–266 °C (dec.), yield 254 mg, 80%, obtained from 3-(2-(3-bromophenyl)-4-(4-bromophenyl)-5-(methoxycarbonyl)-1*H*-pyrrol-3-yl-1-methyl-

1*H*-imidazol-3-ium bromide (**1d**, 366 mg, 0.615 mmol) and aq solution of KOH (69 mg, 1.232 mmol, 4 mL H_2O) according to the general procedure. ^1H NMR (DMSO-d_6): δ 3.54 (s, 3H), 3.85 (s, 3H), 6.85-6.87 (m, 1H), 7.06-7.12 (m, 3H), 7.21-7.23 (m, 1H), 7.35-7.37 (m, 2H), 7.61 (s, 1H), 7.76-7.79 (m, 2H), 9.17 (s, 1H). ^{13}C NMR (DMSO-d_6): δ 35.9 (CH_3), 49.6 (CH_3), 115.5 (C), 118.7 (C), 121.8(C), 122.8 (CH), 123.7 (CH), 126.0 (CH), 126.5 (C), 126.8 (C), 127.1 (CH), 127.7 (CH), 130.0 (CH), 130.3 (CH), 131.7 (CH), 134.0 (C), 134.8 (C), 138.1 (CH), 139.3 (C), 164.0 (C). ESI/HRMS (m/z): 517.9720 calcd for $C_{22}H_{18}Br_2N_3O_2$ $[M + H]^+$, found 517.9766. IR (KBr, cm^{-1}): ν 3142, 3073, 1670.

2-Methoxycarbonyl-3,5-diphenyl-4-(1-phenyl-1*H*-imidazol-3-ium-3-yl)pyrrol-1-ide (2e): colorless solid, mp 235–236 °C (dec.), yield 172 mg, 91%, obtained from 3-(5-(methoxycarbonyl)-2,4-dipheny-1*H*-pyrrol-3-yl)-1-phenyl-1*H*-imidazol-3-ium bromide (**1e**, 226 mg, 0.452 mmol) and aq solution of KOH (51 mg, 0.911 mmol, 3 mL H_2O) according to the general procedure. ^1H NMR (DMSO-d_6): δ 3.53 (s, 3H), 7.04-7.14 (m, 2H), 7.17-7.22 (m, 6H), 7.31-7.33 (m, 2H), 7.53-7.58 (m, 1H), 7.59-7.64 (m, 2H), 7.68-7.71 (m, 2H), 7.99-8.00 (m, 1H), 8.40-8.41 (m, 1H), 9.94-9.95 (m, 1H). ^{13}C NMR (DMSO-d_6): δ 49.5 (CH_3), 115.2 (C), 120.9 (CH), 121.5 (CH), 124.8 (CH), 125.1 (CH), 125.3 (CH), 126.0 (C), 127.1 (CH), 127.3 (CH), 128.1 (CH), 128.1 (C), 129.6 (CH), 129.8 (CH), 130.2 (CH), 134.4 (C), 135.4 (C), 135.8 (C), 136.1 (CH), 137.0 (C), 165.0 (C). ESI/HRMS (m/z): 420.1707 calcd for $C_{27}H_{22}N_3O_2$ $[M + H]^+$, found 420.1724. IR (KBr, cm^{-1}): ν 3377, 3129, 1657.

2-(4-Bromophenyl)-5-methoxycarbonyl-4-phenyl-3-(1-phenyl-1*H*-imidazol-3-ium-3-yl)pyrrol-1-ide (2f): colorless solid, mp 228–229 °C (dec.), yield 242 mg, 94%, obtained from 3-(2-(4-bromophenyl)-5-(methoxycarbonyl)-4-phenyl-1*H*-pyrrol-3-yl)1-phenyl-1*H*-imidazol-3-ium bromide (**1g**, 300 mg, 0.519 mmol) and aq solution of KOH (59 mg, 1.054 mmol, 4 mL H_2O) according to the general procedure. ^1H NMR (DMSO-d_6): 3.53 (s, 3H), 7.08-7.14 (m, 1H), 7.17-7.21 (m, 4H), 7.26 (d, J = 8.5 Hz, 2H), 7.37 (d, J = 8.5 Hz, 2H), 7.54-7.58 (m, 1H), 7.60-7.64 (m, 2H), 7.70-7.72 (m, 1H), 8.00 (s, 1H), 8.41 (s, 1H), 9.96 (s, 1H). ^{13}C NMR (DMSO-d_6): δ 49.5 (CH_3), 115.4 (C), 117.5 (C), 121.1 (CH), 121.6 (CH), 125.4 (CH), 126.4 (C), 126.9 (CH), 127.2 (CH), 127.2 (CH), 128.4 (C), 129.6 (CH), 129.8 (CH), 130.2 (CH), 131.0 (CH), 134.1 (C), 134.4 (C), 135.5 (C), 136.1 (CH), 136.2 (C), 164.9 (C). ESI/HRMS (m/z): 500.0793 calcd for $C_{27}H_{21}BrN_3O_2$ $[M + H]^+$, found 500.0819. IR (KBr, cm^{-1}): ν 3447, 3130, 1662, 1597, 1544.

2-Methoxycarbonyl-5-(4-methoxyphenyl)-3-methyl-4-(1-phenyl-1H-imidazol-3-ium-3-yl)pyrrol-1-ide (2g): colorless solid, mp 206–208 °C (dec.), yield 335 mg, 82%, obtained from 3-(5-(methoxycarbonyl)-2-(4-methoxyphenyl)-4-methyl-1*H*-pyrrol-3-yl)-1-phenyl-1*H*-imidazol-3-ium bromide (**1i**, 494 mg, 1.055 mmol) and aq solution of KOH (119 mg, 2.125 mmol, 5 mL H_2O) according to the general procedure. ^1H NMR (DMSO-d_6): δ 2.14 (s, 3H), 3.63 (s, 3H), 3.68 (s, 3H), 6.75 (d, J = 8.9 Hz, 2H), 7.15 (d, J = 8.9 Hz, 2H), 7.57-7.60 (m, 1H), 7.64-7.68 (m, 2H), 7.88-7.90 (m, 2H), 7.96-7.97 (m, 1H), 8.53-8.54 (m, 1H), 10.04 (s, 1H). ^{13}C NMR (DMSO-d_6): δ 10.3 (CH_3), 49.3 (CH_3), 54.9 (CH_3) 113.5 (CH), 115.6 (C), 121.1 (CH), 121.7 (CH), 122.4 (C), 125.1 (C), 126.2 (CH), 126.6 (CH), 129.6 (CH), 129.9 (C), 130.1 (CH), 134.7 (C), 134.9 (C), 135.8 (CH), 156.8 (C), 165.3 (C). ESI/HRMS (m/z): 390.1723 calcd for $C_{23}H_{22}N_3O_3$ $[M + H]^+$, found 390.1756. IR (KBr, cm^{-1}): ν 3377, 2943, 1670.

3-(1-Benzyl-1*H*-imidazol-3-ium-3-yl)-2-(4-fluorophenyl)-5-methoxycarbonyl-4-phenylpyrrol-1-ide (2h): colorless solid, mp 188–190 °C, yield 64 mg, 88%, obtained from 1-benzyl-3-(2-(4-fluorophenyl)-5-(methoxycarbonyl)-4-phenyl-1*H*-pyrrol-3-yl)-1*H*-imidazol-3-ium bromide (**1l**, 86 mg, 0.162 mmol) and aq solution of KOH (18 mg, 0.324 mmol, 2 mL H_2O) according to the general procedure. ^1H NMR (DMSO-d_6): 3.53 (s, 3H), 5.38 (s, 2H), 6.94-7.03 (m, 2H), 7.05-7.18 (m, 7H), 7.18-7.27 (m, 2H), 7.35-7.42 (m, 3H), 7.72 (s, 1H), 7.80 (s 1H), 9.36 (s, 1H). ^{13}C NMR (DMSO-d_6): δ 49.6 (CH_3), 51.8 (CH_2), 114.8 (d, J = 21.0 Hz, CH), 115.2 (C),

122.6 (CH), 125.3 (C), 125.4 (CH), 126.6 (CH), 126.8 (d, J = 7.6 Hz, CH), 127.1 (CH), 127.2 (CH), 128.1 (C), 128.5 (CH), 128.9 (CH), 129.6 (CH), 133.1 (C), 134.4 (C), 135.2 (C), 135.5 (C), 138.0 (CH), 160.2 (d, J = 241.7 Hz, C), 164.7 (C). ESI/HRMS (m/z): 452.1769 calcd for $C_{28}H_{23}FN_3O_2$ $[M + H]^+$, found 452.1785. IR (KBr, cm^{-1}): ν 3424, 3130, 1662, 1521.

3-(1-Benzyl-1*H*-imidazol-3-ium-3-yl)-2-(4-chlorophenyl)-5-methoxycarbonyl-4-phenylpyrrol-1-ide (2i): colorless solid, mp 245 °C, yield 81 mg, 95%, obtained from1-benzyl-3-(2-(4-chlorophenyl)-5-(methoxycarbonyl)-4-phenyl-1*H*-pyrrol-3-yl)-1*H*-imidazol-3-ium bromide (**1m**, 100 mg, 0.182 mmol) and aq solution of KOH (20 mg, 0.364 mmol, 2 equiv, 3 mL H_2O) according to the general procedure. ^1H NMR (DMSO-d_6): δ 3.52 (s, 3H), 5.38 (s, 2H), 7.07-7.22 (m, 11H), 7.33-7.43 (m, 3H), 7.72 (s, 1H), 7.80 (s, 1H), 9.34 (s, 1H). ^{13}C NMR (DMSO-d_6): δ 49.5 (CH_3), 51.8 (CH_2), 115.5 (C), 122.6 (CH), 125.3 (CH), 126.1 (C), 126.4 (CH), 126.5 (CH), 127.1 (CH), 127.2 (CH), 128.0 (CH), 128.3 (C), 128.4 (CH), 128.9 (CH), 129.0 (C), 129.5 (CH), 134.1 (C), 135.2 (C), 135.6 (C), 135.9 (C), 137.9 (CH), 164.9 (C). ESI/HRMS (m/z): 468.1473 calcd for $C_{28}H_{23}ClN_3O_2$ $[M + H]^+$, found 468.1475. IR (KBr, cm^{-1}): ν 3572, 3134, 1679.

General procedure for the synthesis of 4-(2-thioxo-2,3-dihydro-1*H*-imidazol-1-yl)-1*H*-pyrrol-2-carboxylates 13 from pyrrolides 2. A suspension of 3-(1*H*-imidazol-3-ium-3-yl)-5-(methoxycarbonyl)-pyrrol-1-ide **2** (1 mmol) and sulfur (2 mmol, 2 equiv) in dry THF was stirred at rt for 1–2 hours (monitored by TLC). Then the reaction mixture was evaporated to dryness and the residue was purified by column chromatography on silica gel (hexane/ethyl acetate from 1:1 to 0:1) to give the analytically pure compound.

Methyl 4-(3-methyl-2-thioxo-2,3-dihydro-1*H*-imidazol-1-yl)-3,5-diphenyl-1*H*-pyrrole-2-carboxylate (13a): colorless solid, mp 261–262 °C, yield 43 mg, 80%, obtained from 2-(methoxycarbonyl)-4-(1-methyl-1*H*-imidazol-3-ium-3-yl)-3,5-diphenylpyrrol-1-ide (**2a**, 50 mg, 0.140 mmol) and sulfur (9 mg, 0.280 mmol) according to the general procedure. ^1H NMR (DMSO-d_6): δ 3.46 (s, 3H), 3.67 (s, 3H), 6.84 (d, J = 2.3 Hz, 1H), 7.03 (d, J = 2.3 Hz, 1H), 7.17-7.42 (m, 8H), 7.52 (d, J = 7.0 Hz, 2H), 12.41 (s, 1H). ^{13}C NMR (DMSO-d_6): δ 34.9 (CH_3), 51.1 (CH_3), 117.1 (C), 118.9 (CH), 119.6 (CH), 119.7 (C), 127.0 (CH), 127.3 (CH), 127.5 (CH), 128.1 (CH), 128.3 (CH), 129.4 (C), 129.7 (C), 130.1 (CH), 132.1 (C), 132.4 (C), 160.6 (C), 165.4 (C). ESI/HRMS (m/z): 412.1090 calcd for $C_{22}H_{19}N_3O_2SNa$ $[M + Na]^+$, found 412.1112. IR (KBr, cm^{-1}): ν 3304, 1662, 1454, 1375.

Methyl 5-(4-chlorophenyl)-4-(3-methyl-2-thioxo-2,3-dihydro-1*H*-imidazol-1-yl)-3-phenyl-1*H*-pyrrole-2-carboxylate (13b): colorless solid, mp 245–247 °C, yield 26 mg, 81%, obtained from 2-(4-chlorophenyl)-5-(methoxycarbonyl)-3-(1-methyl-1*H*-imidazol-3-ium-3-yl)-4-phenylpyrrol-1-ide (**2b**, 30 mg, 0.0765 mmol) and sulfur (5 mg, 0.153 mmol, 2 equiv) according to the general procedure. ^1H NMR (DMSO-d_6): δ 3.45 (s, 3H), 3.67 (s, 3H), 6.85 (d, J = 2.4 Hz, 1H), 7.05 (d, J = 2.3 Hz, 1H), 7.19-7.28 (m, 3H), 7.36 (dd, J = 7.7 Hz, J = 1.6 Hz, 2H), 7.43 (d, J = 8.6 Hz, 2H), 7.51 (d, J = 8.7 Hz, 2H), 12.53 (s, 1H). ^{13}C NMR (DMSO-d_6): δ 34.9 (CH_3), 51.2 (CH_3), 117.5 (C), 119.1 (CH), 119.5 (C), 120.0 (CH), 127.0 (CH), 127.3 (CH), 128.3 (C), 128.4 (CH), 129.2 (CH), 129.6 (C), 130.1 (CH), 131.2 (C), 131.9 (C), 132.8 (C), 160.5 (C), 165.3 (C). ESI/HRMS (m/z): 446.0700 calcd for $C_{22}H_{18}ClN_3O_2SNa$ $[M + Na]^+$, found 446.0709. IR (KBr, cm^{-1}): ν 3225, 3126, 2950, 1692.

Methyl 4-(3-methyl-2-thioxo-2,3-dihydro-1*H*-imidazol-1-yl)-5-(4-nitrophenyl)-3-phenyl-1*H*-pyrrole-2-carboxylate (13c): yellow solid, mp 258–287 °C, yield 50 mg, 93%, obtained from (methoxycarbonyl)-4-(1-methyl-1*H*-imidazol-3-ium-3-yl)-5-(4-nitrophenyl)-3-phenylpyrrol-1-ide (**2c**, 50 mg, 0.124 mmol) and sulfur (8 mg, 0.248 mmol) in dry THF (10 mL) under reflux for 1h. ^1H NMR (DMSO-d_6): δ 3.47 (s, 3H), 3.70 (s, 3H), 6.90 (d, J = 2.4 Hz, 1H), 7.09 (d, J = 2.4

Hz, 1H), 7.20-7.30 (m, 3H), 7.36 (dd, *J* = 7.7 Hz, *J* = 1.6 Hz, 2H), 7.72 (d, *J* = 8.9 Hz, 2H), 8.21 (d, *J* = 8.9 Hz, 2H), 12.83 (s, 1H). ^{13}C NMR (DMSO-d$_6$): δ 34.9 (CH_3), 51.4 (CH_3), 118.9 (C), 119.3 (CH), 119.4 (CH), 121.3 (C), 123.6 (CH), 127.2 (CH), 127.4 (CH), 128. 1 (CH), 129.8 (C), 129.9 (C), 130.1 (CH), 131.6 (C), 135.7 (C), 146.5 (C), 160.4 (C), 165.1 (C). ESI/HRMS (m/z): 457.0941 calcd for $C_{22}H_{18}N_4O_4SNa$ [M + Na]$^+$, found 457.0953. IR (KBr, cm^{-1}): ν 3275, 1724, 1598, 1517.

Methyl 5-(3-bromophenyl)-3-(4-bromophenyl)-4-(3-methyl-2-thioxo-2,3-dihydro-1*H*-imidazol-1-yl)-1*H*-pyrrole-2-carboxylate (13d): colorless solid, mp 280–281 °C, yield 49 mg, 92%, obtained from 2-(3-bromophenyl)-4-(4-bromophenyl)-5-(methoxycarbonyl)-3-(1-methyl-1*H*-imidazol-3-ium-3-yl)pyrrol-1-ide (**2d**, 50 mg, 0.097 mmol) and sulfur (6 mg, 0.194 mmol) according to the general procedure. ^{1}H NMR (DMSO-d$_6$): δ 3.47 (s, 3H), 3.70 (s, 3H), 6.91 (d, *J* = 2.4 Hz, 1H), 7.10 (d, *J* = 2.4 Hz, 1H), 7.27–7.34 (m, 1H), 6.91 (d, *J* = 2.4 Hz, 2H), 7.40-7.52 (m, 4H), 7.76 (t, *J* = 1.7 Hz, 1H), 12.66 (s, 1H). ^{13}C NMR (DMSO-d$_6$): δ 34.9 (CH_3), 51.3 (CH_3), 117.8 (C), 119.3 (CH), 119.4 (CH), 120.1 (C), 120.7 (C), 121.7 (C), 126.2 (CH), 128.3 (C), 130.1 (CH), 130.3 (CH), 130.5 (CH), 130.8 (CH), 131.2 (C), 131.3 (C), 132.2 (CH), 160.3 (C), 165. 2 (C). ESI/HRMS (m/z): 547.9461 calcd for $C_{22}H_{18}Br_2N_3O_2S$ [M + H]$^+$, found 547.9470. IR (KBr, cm^{-1}): ν 3302, 1667, 1485, 1452.

Methyl 3,5-diphenyl-4-(3-phenyl-2-thioxo-2,3-dihydro-1*H*-imidazol-1-yl)-1*H*-pyrrole-2-carboxylate (13e): colorless solid, mp 266–267 °C, yield 49 mg, 91%, obtained from 2-(methoxycarbonyl)-3,5-diphenyl-4-(1-phenyl-1*H*-imidazol-3-ium-3-yl)pyrrol-1-ide (**2e**, 50 mg, 0.119 mmol) and sulfur (8 mg, 0.238 mmol) according to the general procedure. ^{1}H NMR (DMSO-d$_6$): δ 3.69 (s, 3H), 7.06 (d, *J* = 2.5 Hz, 1H), 7.22-7.49 (m, 10H), 7.49-7.55 (m, 2H), 7.58 (d, *J* = 7.4 Hz, 2H), 7.61 (dd, *J* = 10.6 Hz, *J* = 3.4 Hz, 2H), 12.48 (s, 1H). ^{13}C NMR (DMSO-d$_6$): δ 51.2 (CH_3), 117.2 (C), 118.9 (CH), 119.6 (C), 121.0 (CH), 125.7 (CH), 127.1 (CH), 127.3 (CH), 127.5 (CH), 127.9 (CH), 128.2 (CH), 128.4 (CH), 128.9 (CH), 129.4 (C), 129.6 (C), 130.2 (CH), 132.1 (C), 132.5 (C), 138.2 (C), 160.6 (C), 165.8 (C). ESI/HRMS (m/z): 452.1427 calcd for $C_{27}H_{22}N_3O_2S$ [M + H]$^+$, found 452.1441. IR (KBr, cm^{-1}): ν 3292, 1665, 1500, 1450.

Methyl 5-(4-bromophenyl)-3-phenyl-4-(3-phenyl-2-thioxo-2,3-dihydro-1*H*-imidazol-1-yl)-1*H*-pyrrole-2-carboxylate (13f): colorless solid, mp 260–261 °C, yield 43 mg, 80%, obtained from 2-(4-bromophenyl)-5-(methoxycarbonyl)-4-phenyl-3-(1-phenyl-1*H*-imidazol-3-ium-3-yl)pyrrol-1-ide (**2f**, 50 mg, 0.100 mmol) and sulfur (7 mg, 0.200 mmol) in dry THF (8 mL) according to the general procedure. ^{1}H NMR ($CDCl_3$): δ 3.74 (s, 3H), 6.49 (d, *J* = 2.5 Hz, 1H), 6.72 (d, *J* = 2.5 Hz, 1H), 7.28-7.37 (m, 3H), 738-7.44 (m, 1H), 7.46-7.59 (m, 10H), 9.48 (s, 1H). ^{13}C NMR (DMSO-d$_6$): δ 51.2 (CH_3), 117.6 (C), 119.0 (CH), 119.8 (C), 120.8 (CH), 121.6 (C), 125.8 (CH), 127.2 (CH), 127.4 (CH), 127.9 (CH), 128.6 (C), 128.9 (CH), 129.4 (CH), 129.6 (C), 130.1 (CH), 131.2 (C), 131.4 (CH), 131.9 (C), 138.2 (C), 160.5 (C), 165.6 (C). ESI/HRMS (m/z): 554.0332 calcd for $C_{27}H_{20}BrN_3O_2SNa$ [M + Na]$^+$, found 554.0358. IR (KBr, cm^{-1}): ν 3316, 1664, 1599, 1499.

Methyl 5-(4-methoxyphenyl)-3-methyl-4-(3-phenyl-2-thioxo-2,3-dihydro-1*H*-imidazol-1-yl)-1*H*-pyrrole-2-carboxylate (13g): colorless solid, mp 256–257 °C (dec.), yield 112 mg, 90%, obtained from 2-(methoxycarbonyl)-5-(4-methoxyphenyl)-3-methyl-4-(1-phenyl-1*H*-imidazol-3-ium-3-yl)pyrrol-1-ide (**2g**, 115 mg, 0.297 mmol) and sulfur (19 mg, 0.594 mmol) in dry THF (8 mL) according to the general procedure. ^{1}H NMR ($CDCl_3$): δ 2.09 (s, 3H), 3.76 (s, 3H), 3.83 (s, 3H), 6.93-6.96 (m, 2H), 7.16-7.17 (m, 2H), 7.40-7.41 (m, 2H), 7.45-7.48 (m, 2H), 7.53-7.57 (m, 2H), 12.00 (s, 1H). ^{13}C NMR (DMSO-d$_6$): δ 9.9 (CH_3), 51.1 (CH_3), 55.1 (CH_3), 114.0 (CH), 117.0 (C), 119.1 (CH), 119.9 (C), 120.5 (CH), 121.9 (C), 125.7 (C), 125.8 (CH), 127.9 (CH), 128.5 (CH), 128.9 (CH), 131.9 (C), 138.3 (C), 159.1 (C), 161.2 (C), 164.7 (C). ESI/HRMS

(m/z): 442.1196 calcd for $C_{23}H_{21}N_3O_3SNa$ [M + Na]$^+$, found 442.1217. IR (KBr, cm^{-1}): ν 3337, 1667, 1612, 1458.

Methyl 4-(3-benzyl-2-thioxo-2,3-dihydro-1*H*-imidazol-1-yl)-5-(4-chlorophenyl)-3-phenyl-1*H*-pyrrole-2-carboxylate (13h): colorless solid, mp 249 °C, yield 11 mg, 80%, obtained from 3-(1-benzyl-1*H*-imidazol-3-ium-3-yl)-2-(4-chlorophenyl)-5-(methoxycarbonyl)-4-phenylpyrrol-1-ide (**2i**, 13 mg, 0.0278 mmol) and sulfur (2 mg, 0.0056 mmol, 2 equiv) according to the general procedure.^{1}H NMR (DMSO-d_6): δ 3.69 (s, 3H), 5.16-5.31 (m, 2H), 6.91 (d, *J* = 2.4 Hz, 1H), 7.07 (d, *J* = 2.4 Hz, 1H), 7.12 (d, *J* = 6.5 Hz, 2H), 7.21-7.44 (m, 10H), 7.55 (d, *J* = 8.6 Hz, 2H), 12.58 (s, 1H). ^{13}C NMR (DMSO-d_6): δ 50.0 (CH_2), 51.2 (CH_3), 117.5 (C), 118.3 (CH), 120.0 (C), 120.2 (CH), 127.0 (CH), 127.1 (CH), 127.3 (CH), 127.4 (CH), 128.2 (C), 128.36 (CH), 128.38 (CH), 129.3 (CH), 129.6 (C), 130.1 (CH), 131.3 (C), 131.9 (C), 132.9 (C), 137.0 (C), 160.5 (C), 165.6 (C). ESI/HRMS (m/z): 522.1013 calcd for $C_{28}H_{22}ClN_3O_2SNa$ [M + Na]$^+$, found 522.1023. IR (KBr, cm^{-1}): ν 3243, 3137, 2923, 1693.

4-(1-Methyl-1*H*-imidazol-3-ium-3-yl)-3,5-diphenyl-1*H*-pyrrole-2-carboxylate (6a). A suspension of 2-(methoxycarbonyl)-4-(1-methyl-1*H*-imidazol-3-ium-3-yl)-3,5-diphenylpyrrol-1-ide (**2a**, 118 mg, 0.330 mmol) and LiOH (80 mg, 3.3 mmol, 10 equiv) in a mixture of dioxane (20 mL) and water (2 mL) was stirred at 110 °C for 24 h. Then reaction mixture was evaporated to dryness and water (2 mL) was added. The suspension was filtered, the solid was washed with water (2 × 1 mL) and thoroughly dried to obtain lithium salt **14a**, 63 mg, 55%. To convert lithium salt **14a** (49 mg, 0.139 mmol) into **6a** trichloroacetic acid (23 mg, 0.139 mmol, 1 equiv) was added to a suspension of the lithium salt in water (5 mL). The suspension was sonicated for 10 min, then evaporated to dryness and purified by column chromatography on silica gel (DCM/methanol from 10:1 to 1:1), colorless solid, mp 264–266 °C, yield 31 mg, 64%. ^{1}H NMR (MeOH-d_4): δ 3.86 (s, 3H), 7.17-7.29 (m, 5H), 7.29-7.42 (m, 5H), 7.58 (d, *J* = 1.9 Hz, 1H), 7.61 (d, *J* = 1.9 Hz, 1H). ^{13}C NMR (MeOH-d_4): δ 36.6 (CH_3), 116.6 (C), 125.0 (CH), 125.5 (C), 127.45 (CH), 127.48 (C), 127.9 (CH), 128.1 (CH), 128.9 (CH), 128.91 (C), 129.5 (CH), 130.2 (CH), 130.6 (C), 131.1 (CH), 133.7 (C), 167.9 (C). ESI/HRMS (m/z): 344.1394 calcd for $C_{21}H_{18}N_3O_2$ [M + H]$^+$, found 344.1414. IR (KBr, cm^{-1}): ν 3400, 1584.

5-(4-Chlorophenyl)-4-(1-methyl-1*H*-imidazol-3-ium-3-yl)-3-phenyl-1*H*-pyrrole-2-carboxylate (6b). A suspension of 3-(2-(4-chlorophenyl)-5-(methoxycarbonyl)-4-phenyl-1*H*-pyrrol-3-yl)-1-methyl-1*H*-imidazol-3-ium bromide (**1b**, 100 mg, 0.212 mmol) and LiOH (253 mg, 10.6 mmol, 50 equiv) in mixture of dioxane (30 mL) and water (3 mL) was stirred at 110 °C for 24 h. Then reaction mixture was evaporated to dryness and water (5 mL) was added. The suspension was filtered, the solid was washed with water (2 × 5mL) and thoroughly dried to obtain lithium salt **14b** in quantitive yield. To convert lithium salt **14b** into **6b** trichloroacetic acid (35 mg, 0.212 mmol, 1 equiv) was added to a suspension of the lithium salt in water (5 mL). The suspension was sonicated for 5 min, stirred for 1 h and filtered. Solid was washed with water and dried to obtain **6b** as a colorless solid, mp 205–207 °C, yield 79 mg, 98%. ^{1}H NMR (DMSO-d_6): δ 3.82 (s, 3H), 7.11-7.26 (m, 5H), 7.29 (d, *J* = 8.5 Hz, 2H), 7.36 (d, *J* = 8.5 Hz, 2H), 7.77 (s, 1H), 7.80 (s, 1H), 9.26 (s, 1H). ^{13}C NMR (DMSO-d_6): δ 36.0 (CH_3), 115.1 (C), 120.8 (C), 123.9 (CH), 124.0 (C), 124.2 (C), 125.9 (CH), 126.0 (CH), 127.1 (CH), 128.4 (CH), 128.42 (C), 128.6 (CH), 130.1 (CH), 131.8 (C), 133.1 (C), 138.6 (CH), 162.2 (C). ESI/HRMS (m/z): 378.1004 calcd for $C_{21}H_{17}ClN_3O_2$ [M + H]$^+$, found 378.1009. IR (KBr, cm^{-1}): ν 3498, 3033, 1694.

Single crystal X-ray diffraction experiment

For a single crystal X-ray diffraction experiment, a crystal of **6b** was fixed on a micro mount and placed on an Agilent Technologies SuperNova diffractometer and measured at the temperature of 100 K using monochromated Cu$K\alpha$ radiation. The unit cell parameters (Table S1) were refined by least square techniques using 4960 reflections in the 2θ range of 6.04–153.96. The structure has been solved by direct methods and refined R_1 = 0.056 for 4960 unique reflections with $|F_o| \geq 4\sigma_F$ by means of the SHELXL–97 program [4] incorporated in the *OLEX2* program package [5]. The carbon-bound H atoms were placed in calculated positions and were included in the refinement in the 'riding' model approximation, with U_{iso}(H) set to 1.2U_{eq}(C) and C–H 0.97 Å for the CH_2 groups, U_{iso}(H) set to 1.5U_{eq}(N) and C–H 0.96 Å for the CH_3 groups and U_{iso}(H) set to 1.2U_{eq}(N) and C–H 0.93 Å for the CH groups and N–H 0.86 Å with U_{iso}(H) set to 1.2U_{eq}(C) for the NH group. Empirical absorption correction was applied in the CrysAlisPro [6] program complex using spherical harmonics, implemented in SCALE3 ABSPACK scaling algorithm. The SQUEEZE routine from *PLATON* [7] was applied to the data to account for the solvent void within the lattice. Two void spaces with a total void volume of 254 $Å^{-3}$ and a sum of 75 $\bar{e}$ were accounted for within the unit cell. This could be accounted for by the inclusion of four molecules of methanol per unit cell. Supplementary crystallographic data for this paper have been deposited at Cambridge Crystallographic Data Centre (CCDC 1406417) and can be obtained free of charge via www.ccdc.cam.ac.uk/data_request/cif.

Table S1 Crystal data and structure refinement for **6b**

Empirical formula	$C_{22}H_{20}N_3O_3Cl$
Formula weight	409.86
Temperature/K	100(2)
Crystal system	triclinic
Space group	P-1
a/Å	9.2999(5)
b/Å	9.5698(4)
c/Å	15.3875(5)
α/°	72.782(3)
β/°	78.712(4)
γ/°	66.401(4)
Volume/Å^3	1194.02(9)
Z	2
ρ_{calc}/mg/mm^3	1.140
m/mm^{-1}	1.618
F(000)	428.0
Crystal size/mm^3	0.2 × 0.17 × 0.01
2Θ range for data collection	6.04 to 153.96°
Index ranges	$-11 \leq h \leq 11$, $-11 \leq k \leq 12$, $0 \leq l \leq 19$
Reflections collected	4960
Independent reflections	4960[R(int) = 0.0000]
Data/restraints/parameters	4960/0/265
Goodness-of-fit on F^2	1.094

Final R indexes [I>=2σ (I)]	R_1 = 0.0561, wR_2 = 0.1626
Final R indexes [all data]	R_1 = 0.0631, wR_2 = 0.1681
Largest diff. peak/hole / e Å^{-3}	0.42/-0.46

Table S2 Fractional atomic coordinates (×10^4) and equivalent isotropic displacement parameters (Å^2×10^3) for **6b**. U_{eq} is defined as 1/3 of the trace of the orthogonalised U_{IJ} tensor.

**Atom**	***x***	***y***	***z***	**U(eq)**
Cl1	8014.3(10)	149.6(9)	4858.2(4)	70.0(3)
O1	9805.2(17)	-3607.9(17)	11700(1)	36.4(3)
O2	9147.8(16)	-4964.1(15)	10976.3(10)	32.8(3)
N18	6643.3(17)	1796.2(17)	9231.6(10)	25.3(3)
N20	6115.4(19)	4295.1(17)	8793.2(11)	30.4(3)
N11	8599.9(17)	-2351.7(17)	9459.3(10)	25.5(3)
C8	7494(2)	136(2)	9472.7(12)	25.5(4)
O1S	9661(3)	4546(3)	6527.4(15)	75.6(7)
C5	7129(2)	869(2)	7369.1(14)	34.6(4)
C9	7901(2)	-708(2)	10368.6(12)	25.6(4)
C12	8180(2)	1119(2)	11135.0(13)	28.5(4)
C10	8599(2)	-2277(2)	10335.9(12)	25.5(4)
C1	8690(2)	-1876(2)	7488.4(14)	32.3(4)
C3	7955(3)	-148(3)	6037.3(15)	42.5(5)
C22	5028(2)	2533(2)	9376.3(14)	31.3(4)
C16	6656(2)	-460(2)	11942.8(14)	35.3(4)
C23	6327(3)	5811(2)	8398.1(18)	42.6(5)
C13	7876(2)	1789(2)	11871.2(14)	32.7(4)
C2	8706(3)	-1640(3)	6554.8(15)	39.5(5)
C15	6346(3)	212(3)	12677.7(15)	40.8(5)
C4	7155(3)	1110(3)	6435.3(15)	40.9(5)
C6	7916(2)	-629(2)	7917.3(13)	26.8(4)
C21	4703(2)	4094(2)	9094.9(15)	34.5(4)
C7	7952(2)	-888.1(19)	8904.6(12)	24.9(3)
C19	7268(2)	2898(2)	8877.6(13)	27.6(4)
C17	7580(2)	-20(2)	11163.6(12)	26.1(4)
C14	6960(3)	1324(2)	12645.4(14)	36.8(4)
C24	9236(2)	-3740.6(19)	11067.2(12)	26.1(4)
C1S	9956(8)	3228(6)	6260(3)	117(2)

Table S3 Anisotropic displacement parameters ($Å^2×10^3$) for **6b**. The anisotropic displacement factor exponent takes the form: $-2\pi^2[h^2a^{*2}U_{11}+...+2hka×b×U_{12}]$

Atom	U_{11}	U_{22}	U_{33}	U_{23}	U_{13}	U_{12}
Cl1	89.6(5)	63.4(4)	31.2(3)	-13.2(3)	-21.1(3)	6.5(4)
O1	35.3(7)	33.3(7)	38.1(8)	2.8(6)	-13.0(6)	-13.6(6)
O2	36.6(7)	18.8(6)	35.7(7)	-3.6(5)	0.7(5)	-6.8(5)
N18	24.3(7)	19.3(7)	30.3(7)	-4.7(5)	-5.1(6)	-5.8(6)
N20	31.8(8)	17.6(7)	36.5(8)	-0.4(6)	-9.7(6)	-5.1(6)
N11	25.9(7)	17.9(7)	29.9(7)	-3.7(5)	-2.9(6)	-6.3(6)
C8	25.4(8)	19.6(8)	30.0(9)	-3.2(6)	-4.1(6)	-7.9(6)
O1S	109.5(19)	57.5(12)	48.3(11)	-12.9(9)	-24.3(12)	-11.0(12)
C5	37(1)	27.8(9)	34.7(10)	-7.2(7)	-7.4(8)	-5.8(8)
C9	23.4(8)	21.4(8)	31.0(9)	-4.9(6)	-2.3(6)	-8.3(6)
C12	25.2(8)	24.7(8)	33.1(9)	-5.2(7)	-5.2(7)	-6.8(7)
C10	23.8(8)	19.9(8)	29.9(9)	-2.0(6)	-2.9(6)	-7.5(6)
C1	34.7(10)	26.8(9)	33.7(10)	-5.1(7)	-8.0(7)	-8.7(8)
C3	46.6(12)	42.5(12)	29.6(10)	-7.0(8)	-11.9(9)	-4.8(10)
C22	23.8(8)	26.2(9)	41.5(10)	-7.7(7)	-2.8(7)	-6.9(7)
C16	40.2(10)	29.7(9)	34.8(10)	-5.0(8)	0.5(8)	-15.2(8)
C23	42.9(11)	19.9(9)	57.6(13)	3.2(8)	-12(1)	-9.3(8)
C13	32.5(9)	25.5(9)	39.8(10)	-8.2(7)	-12.4(8)	-5.8(7)
C2	41.4(11)	36.8(11)	35.1(10)	-12.2(8)	-6.2(8)	-5.1(9)
C15	49.6(12)	37.0(11)	30.8(10)	-5.7(8)	2.6(9)	-15.4(9)
C4	47.1(12)	32.4(10)	34.5(10)	-3.0(8)	-13.7(9)	-4.6(9)
C6	24.7(8)	22.7(8)	31.4(9)	-4.1(7)	-3.5(7)	-8.4(7)
C21	25.9(9)	26.5(9)	43.5(11)	-6.8(8)	-6.0(8)	-1.8(7)
C7	22.5(8)	18.0(8)	32.0(9)	-3.7(6)	-3.7(6)	-6.3(6)
C19	27.4(8)	22.1(8)	31.0(9)	-1.5(6)	-8.8(7)	-7.4(7)
C17	23.0(8)	20.6(8)	30.8(9)	-5.0(6)	-4.2(6)	-4.2(6)
C14	41.1(11)	33.7(10)	31.2(9)	-10.7(8)	-9.4(8)	-4.5(8)
C24	21.1(8)	17.8(8)	31.6(9)	-0.9(6)	-1.8(6)	-3.1(6)
C1S	216(6)	100(3)	71(2)	-22(2)	-32(3)	-85(4)

Table S4 Bond lengths for **6b**

Atom	Atom	Length/Å	Atom	Atom	Length/Å
Cl1	C3	1.745(2)	C9	C10	1.389(2)
O1	C24	1.255(2)	C9	C17	1.482(2)
O2	C24	1.255(2)	C12	C13	1.391(3)
N18	C8	1.431(2)	C12	C17	1.397(2)

N18	C22	1.385(2)	C10	C24	1.497(2)
N18	C19	1.331(2)	C1	C2	1.385(3)
N20	C23	1.474(2)	C1	C6	1.404(3)
N20	C21	1.375(3)	C3	C2	1.381(3)
N20	C19	1.326(2)	C3	C4	1.385(3)
N11	C10	1.372(2)	C22	C21	1.348(3)
N11	C7	1.368(2)	C16	C15	1.390(3)
C8	C9	1.413(3)	C16	C17	1.393(3)
C8	C7	1.390(2)	C13	C14	1.391(3)
O1S	C1S	1.349(5)	C15	C14	1.380(3)
C5	C4	1.383(3)	C6	C7	1.470(3)
C5	C6	1.410(3)			

Table S5 Bond angles for **6b**

**Atom**	**Atom**	**Atom**	**Angle/°**	**Atom**	**Atom**	**Atom**	**Angle/°**
C22	N18	C8	125.44(15)	C21	C22	N18	107.14(16)
C19	N18	C8	126.15(15)	C15	C16	C17	120.71(18)
C19	N18	C22	108.32(15)	C12	C13	C14	119.70(18)
C21	N20	C23	125.95(17)	C3	C2	C1	119.3(2)
C19	N20	C23	124.86(17)	C14	C15	C16	120.2(2)
C19	N20	C21	109.13(16)	C5	C4	C3	119.2(2)
C7	N11	C10	111.45(15)	C5	C6	C7	121.59(16)
C9	C8	N18	123.01(16)	C1	C6	C5	117.86(18)
C7	C8	N18	126.66(16)	C1	C6	C7	120.54(16)
C7	C8	C9	110.23(15)	C22	C21	N20	106.93(16)
C4	C5	C6	121.02(19)	N11	C7	C8	104.95(15)
C8	C9	C17	125.76(16)	N11	C7	C6	122.41(15)
C10	C9	C8	105.44(16)	C8	C7	C6	132.54(16)
C10	C9	C17	128.77(17)	N20	C19	N18	108.48(16)
C13	C12	C17	120.81(18)	C12	C17	C9	120.06(16)
N11	C10	C9	107.91(15)	C16	C17	C9	121.37(16)
N11	C10	C24	120.91(15)	C16	C17	C12	118.56(17)
C9	C10	C24	131.15(17)	C15	C14	C13	120.03(19)
C2	C1	C6	121.17(18)	O1	C24	O2	127.26(17)
C2	C3	Cl1	119.07(18)	O1	C24	C10	116.30(15)
C2	C3	C4	121.4(2)	O2	C24	C10	116.44(16)
C4	C3	Cl1	119.55(17)				

Table S6 Hydrogen atom coordinates (Å×10[4]) and isotropic displacement parameters ($Å^2 \times 10^3$) for **6b**

Atom	*x*	*y*	*z*	U(eq)
H11	8962	-3210	9281	31
H1S	9884	4309	7055	113
H5	6585	1709	7640	42
H12	8790	1434	10617	34
H1	9201	-2879	7837	39
H22	4303	2043	9622	38
H16	6242	-1213	11971	42
H23A	5700	6535	8767	64
H23B	6001	6229	7789	64
H23C	7416	5657	8381	64
H13	8284	2544	11846	39
H2	9218	-2477	6279	47
H15	5724	-88	13193	49
H4	6641	2105	6079	49
H21	3711	4884	9103	41
H19	8333	2717	8717	33
H14	6762	1762	13141	44
H1SA	9005	3017	6350	176
H1SB	10739	2360	6616	176
H1SC	10335	3370	5626	176

References

1. Schmidt, A.; Wiechmann, S.; Freese, T. ARKIVOC, **2013**, i, 424-469.
2. Katritzky, A. R.; Øksne, S.; Boulton, A. J. *Tetrahedron*, **1962**, *18*, 777-790.
3. Micetich, R. G.; Chin, C. G. *Can. J. Chem.*, 1970, *48*, 1371-1376.
4. SHELXL, G.M. Sheldrick, Acta Cryst. (2008). A64, 112-122
5. Dolomanov, O.V.; Bourhis, L. J.; Gildea, R. J.; Howard, J. A. K.; Puschmann, H. *J. Appl. Cryst.* **2009**, *42*, 339-341.
6. Palatinus, L.; Chapuis, G. *J. Appl. Cryst.* **2007**, *40*, 786-790
7. Spek, A.L. Acta Cryst. D, **2009**, *65*, 148-155

NMR spectra

3-(5-Methoxycarbonyl-2,4-diphenyl-1*H*-pyrrol-3-yl)-1-methyl-1*H*-imidazol-3-ium bromide (1a), DMSO-d_6

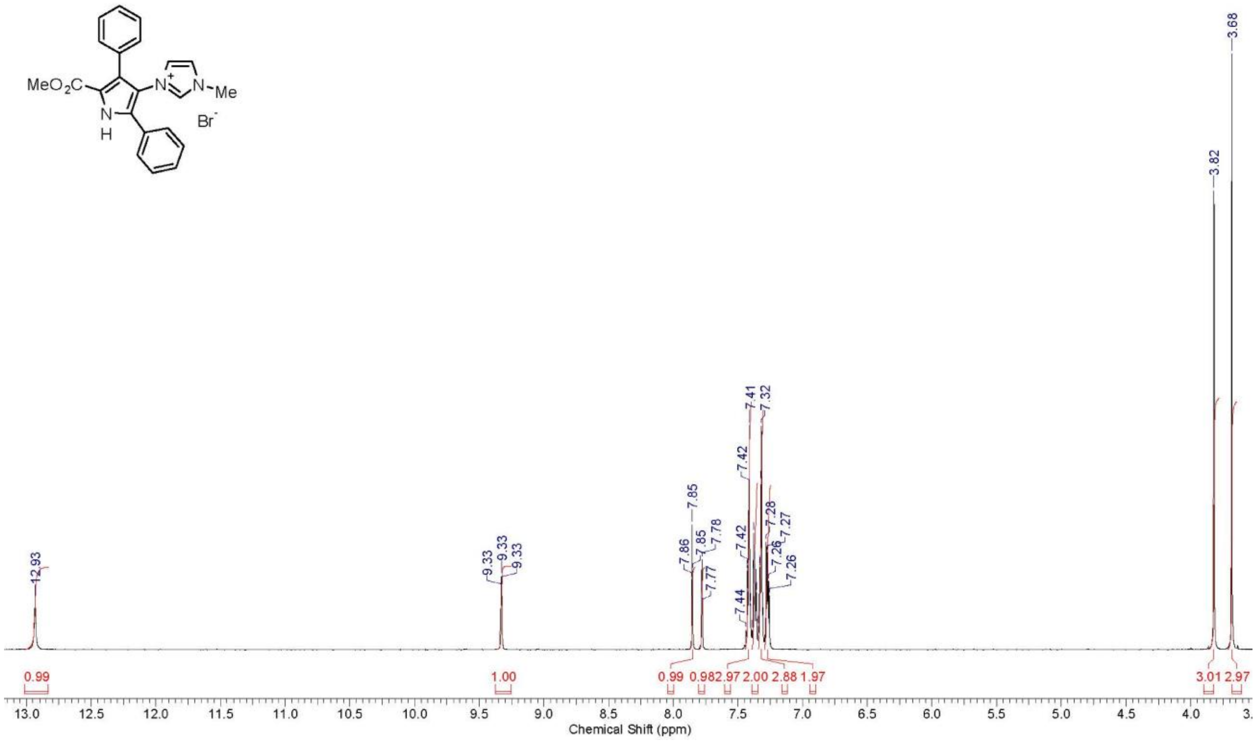

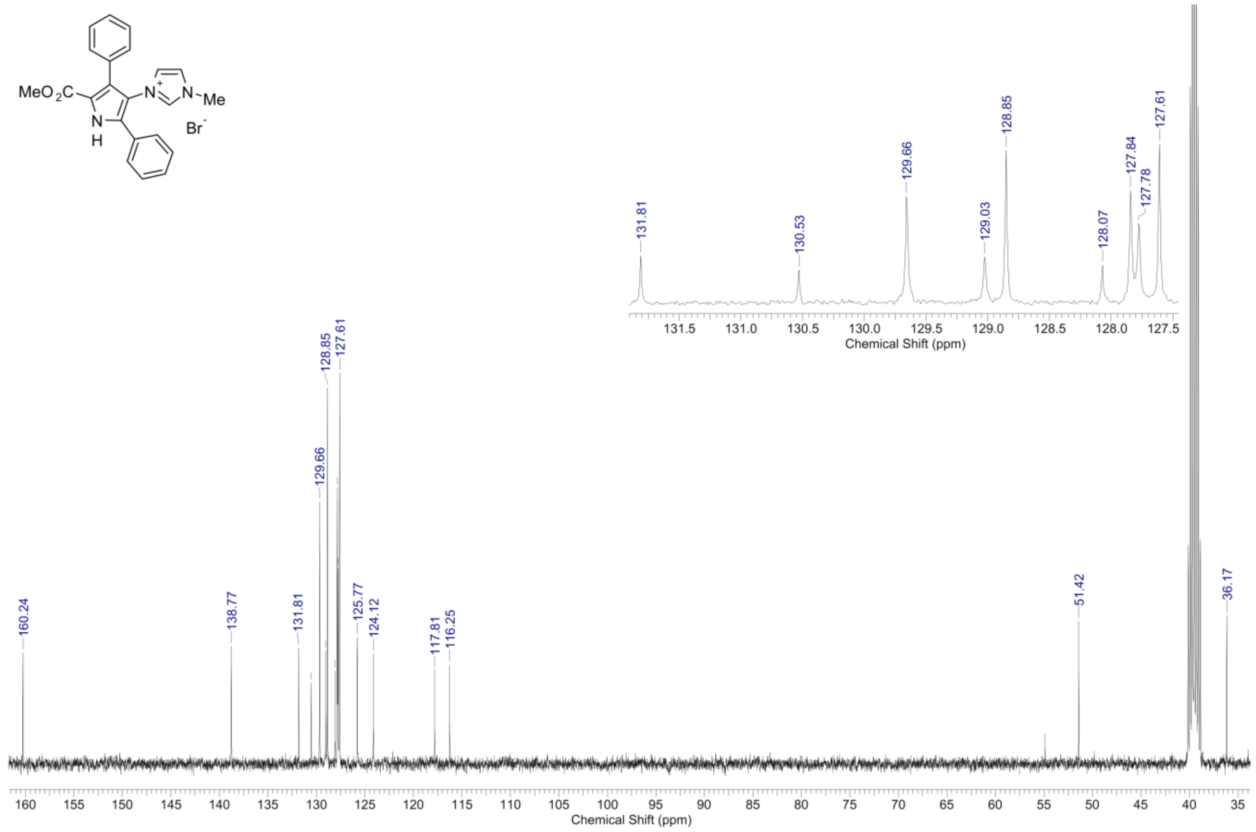

3-(2-(4-Chlorophenyl)-5-methoxycarbonyl-4-phenyl-1*H*-pyrrol-3-yl)-1-methyl-1*H*-imidazol-3-ium bromide (1b), DMSO-d_6

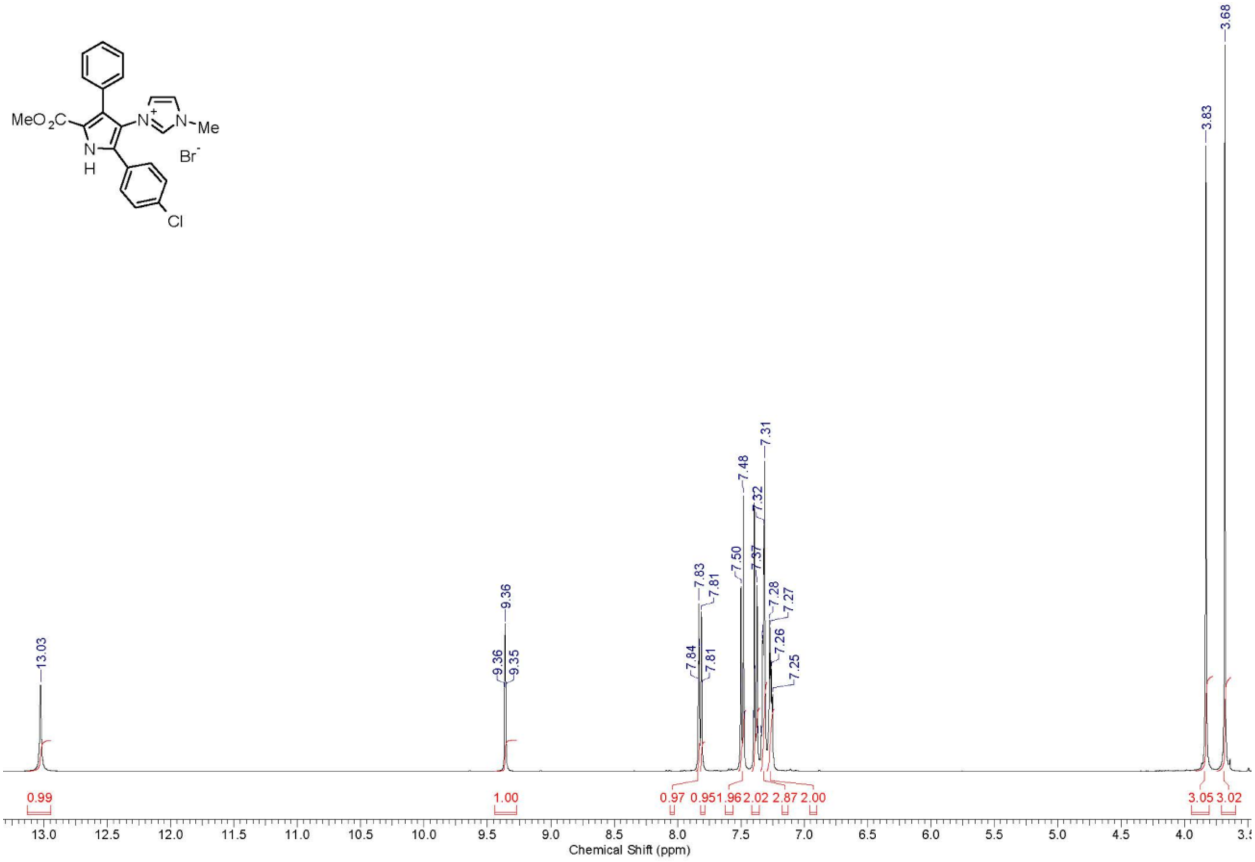

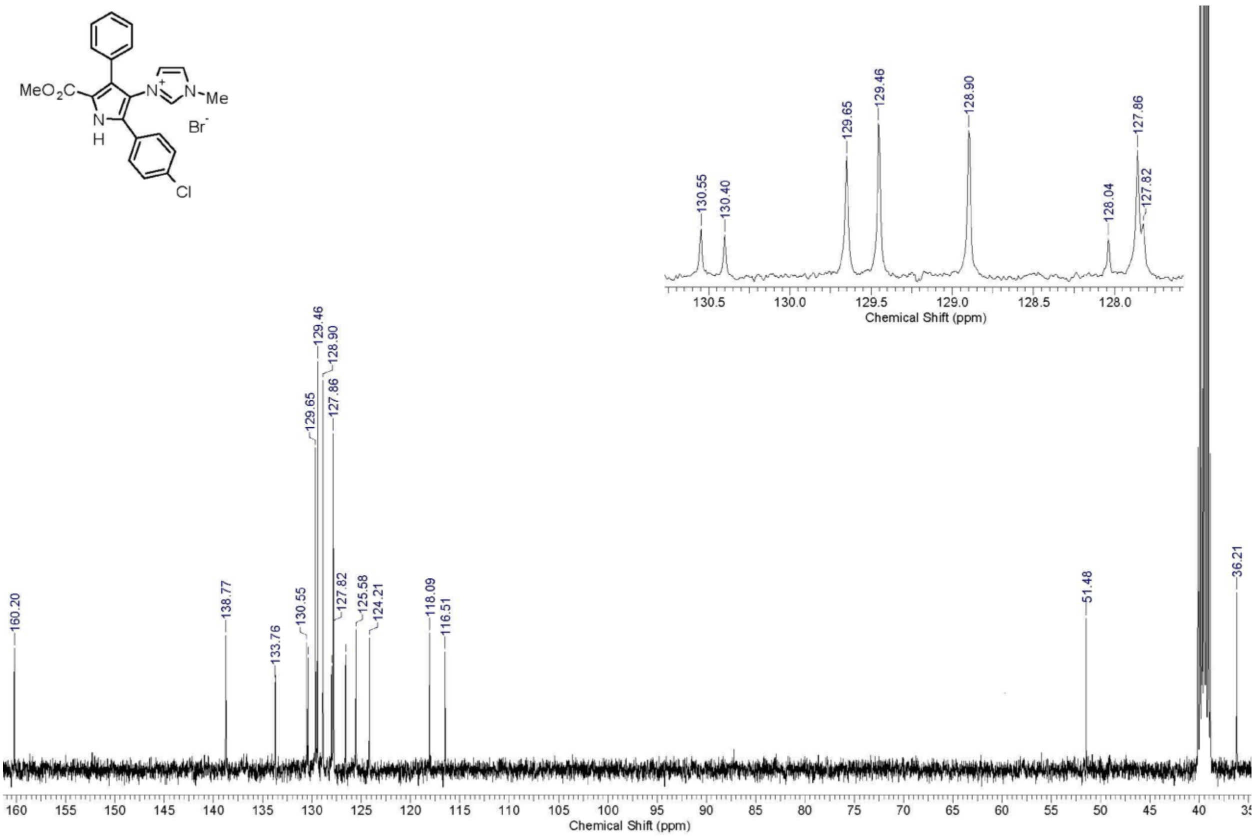

3-(5-Methoxycarbonyl-2-(4-nitrophenyl)-4-phenyl-1*H*-pyrrol-3-yl)-1-methyl-1*H*-imidazol-3-ium bromide (1c), DMSO-d_6

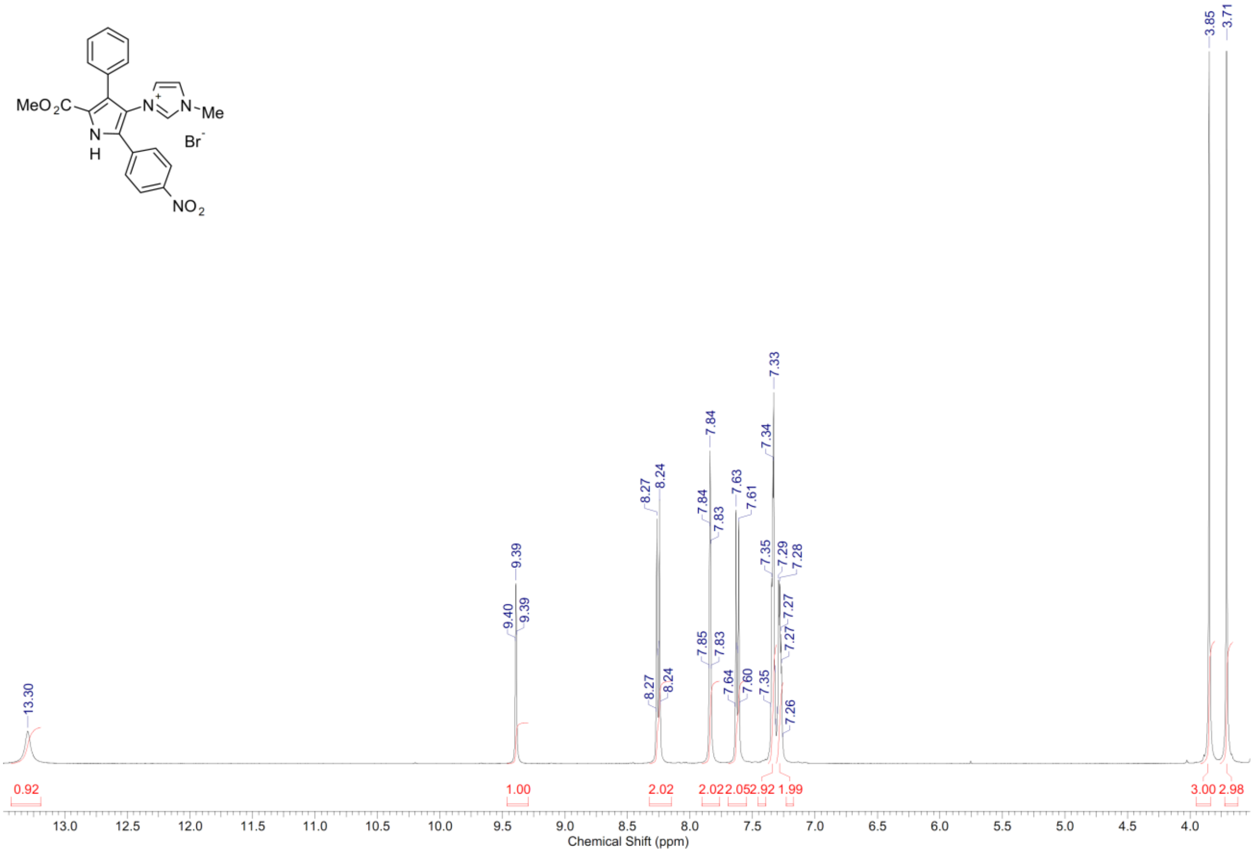

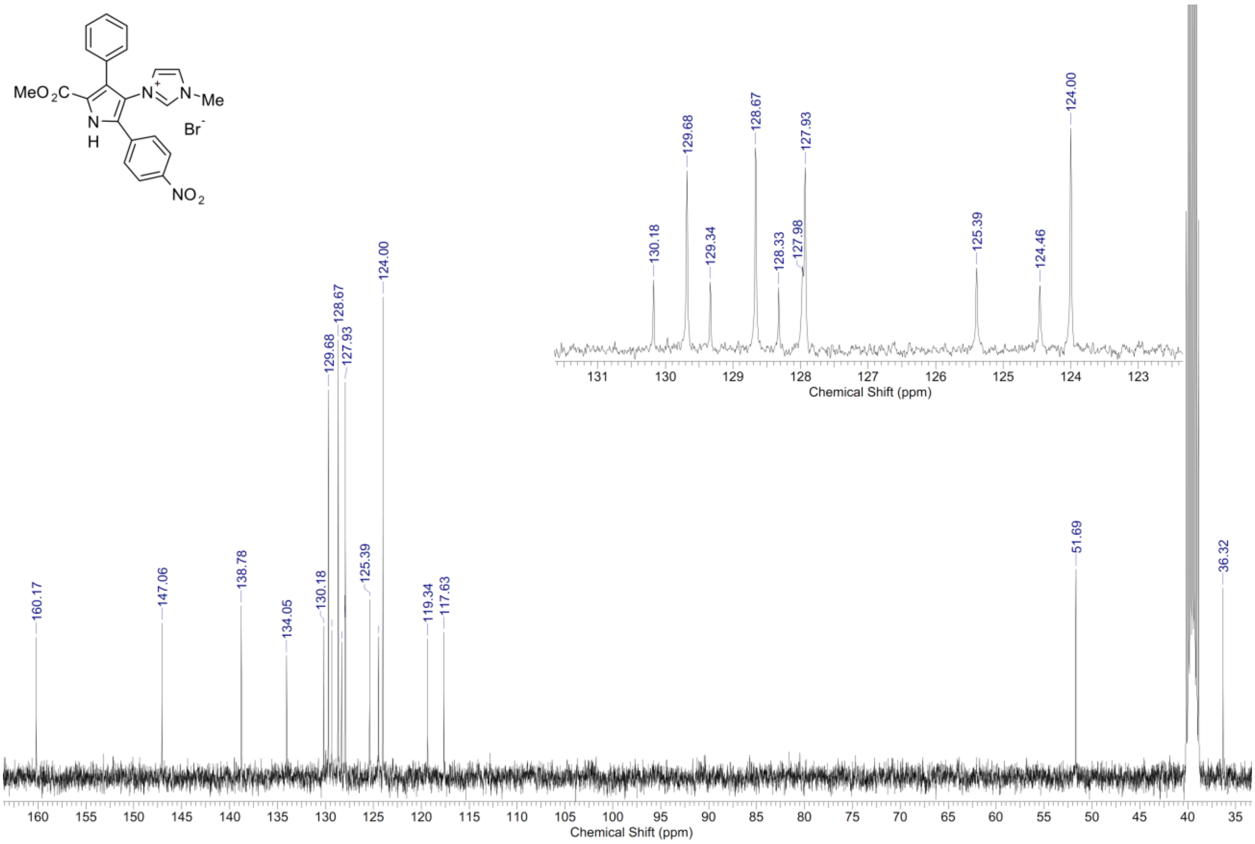

3-(2-(3-bromophenyl)-4-(4-bromophenyl)-5-methoxycarbonyl-1*H*-pyrrol-3-yl)-1-methyl-1*H*-imidazol-3-ium bromide (1d), DMSO-d_6

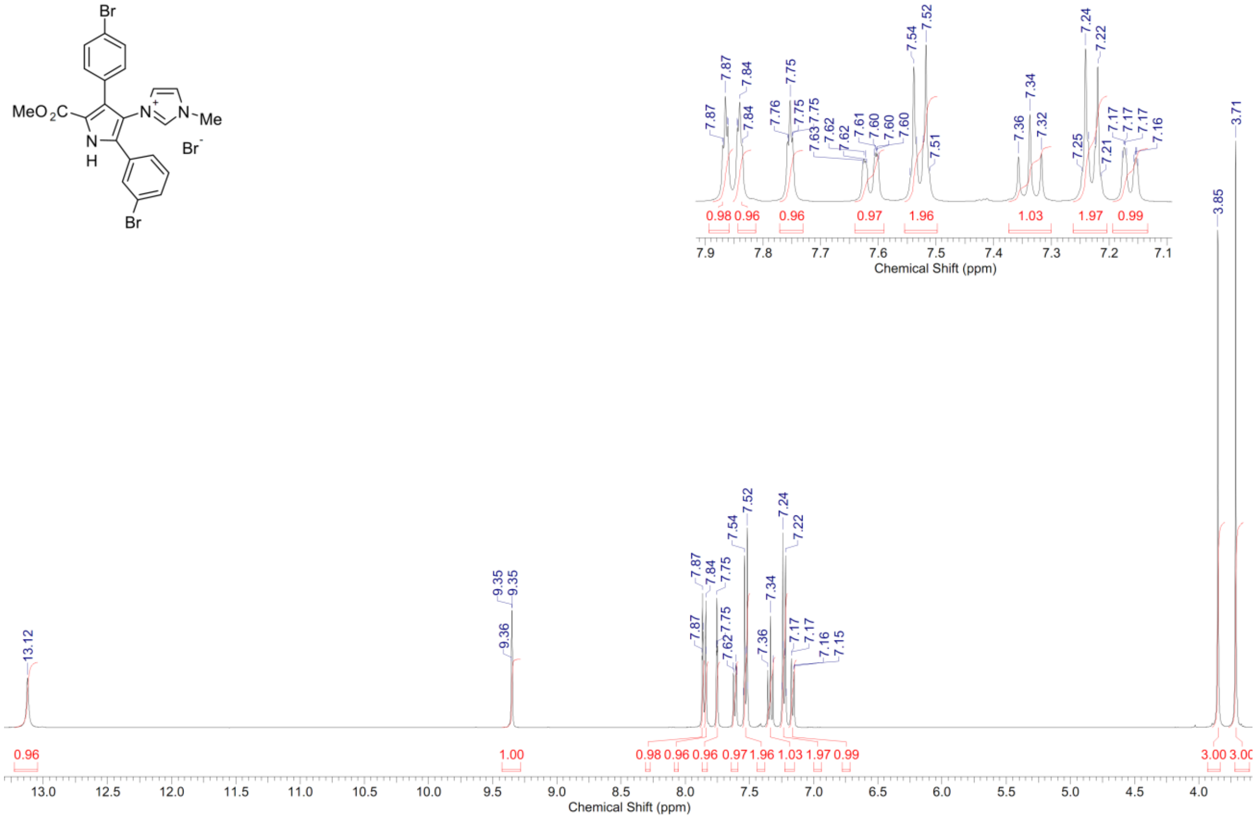

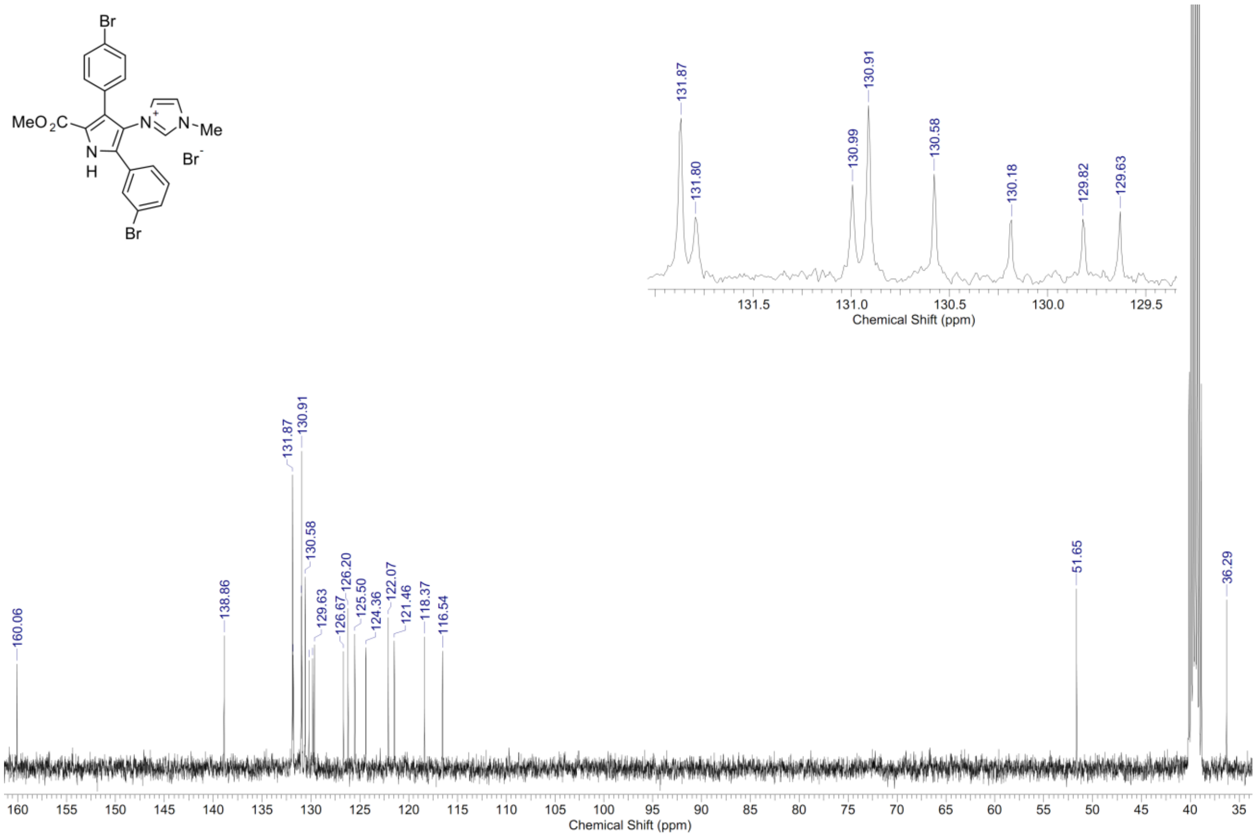

3-(5-Methoxycarbonyl-2,4-diphenyl-1*H*-pyrrol-3-yl)-1-phenyl-1*H*-imidazol-3-ium bromide (1e), DMSO-d_6

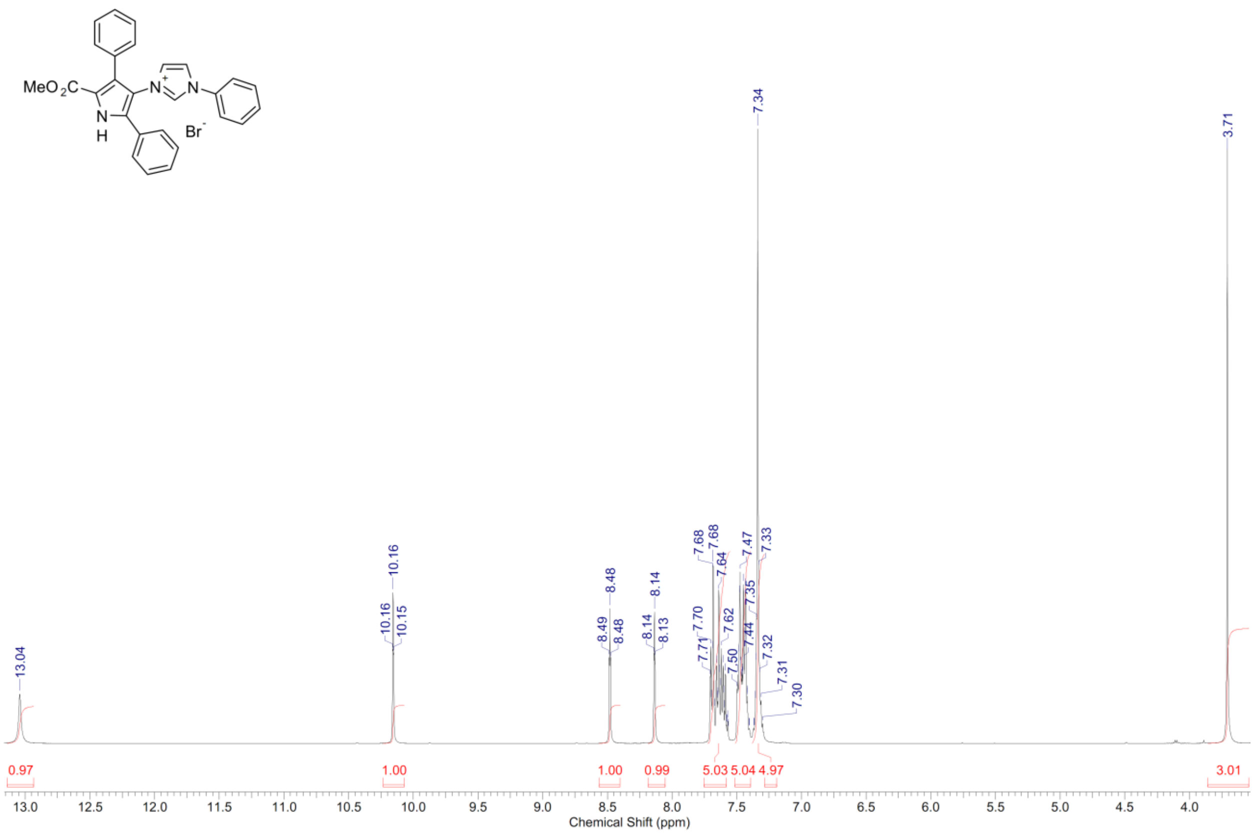

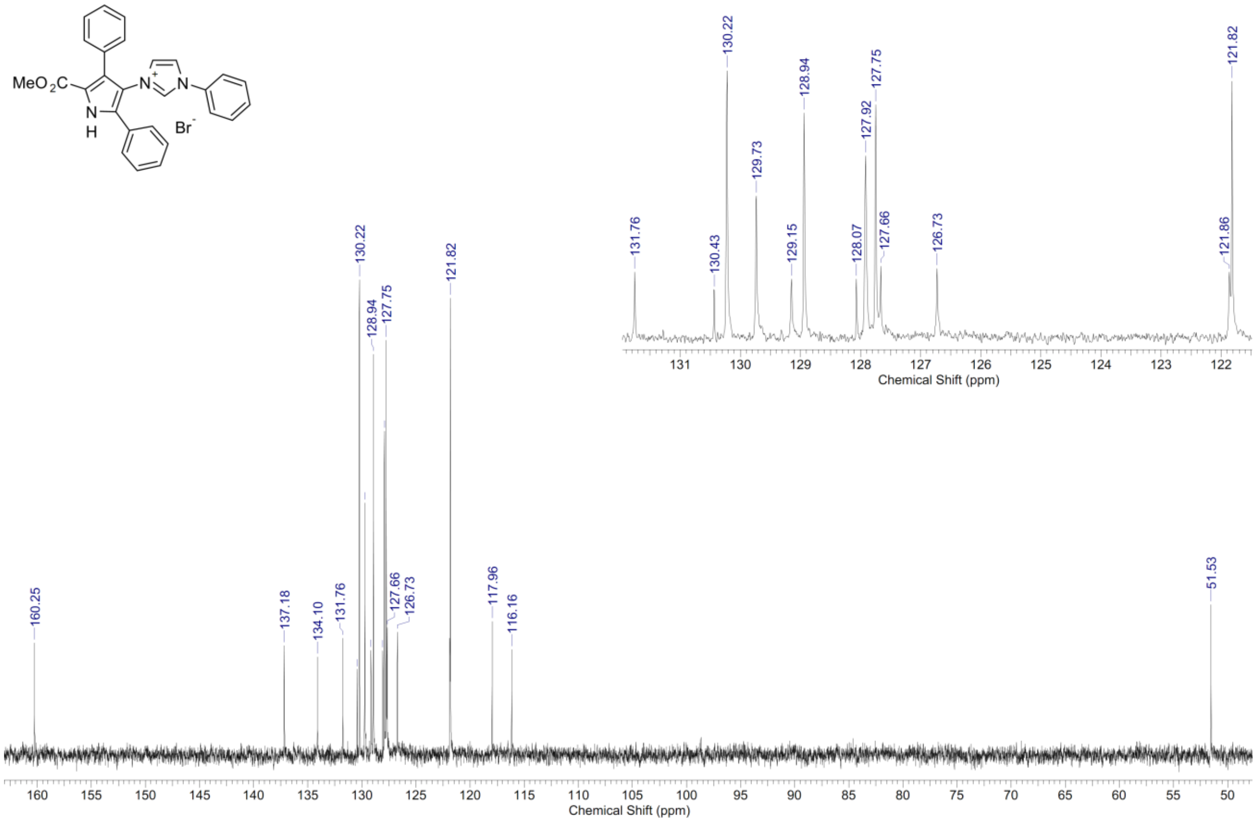

3-(5-Methoxycarbonyl-2-(4-methoxyphenyl)-4-phenyl-1H-pyrrol-3-yl)-1-phenyl-1H-imidazol-3-ium bromide (1f), DMSO-d_6

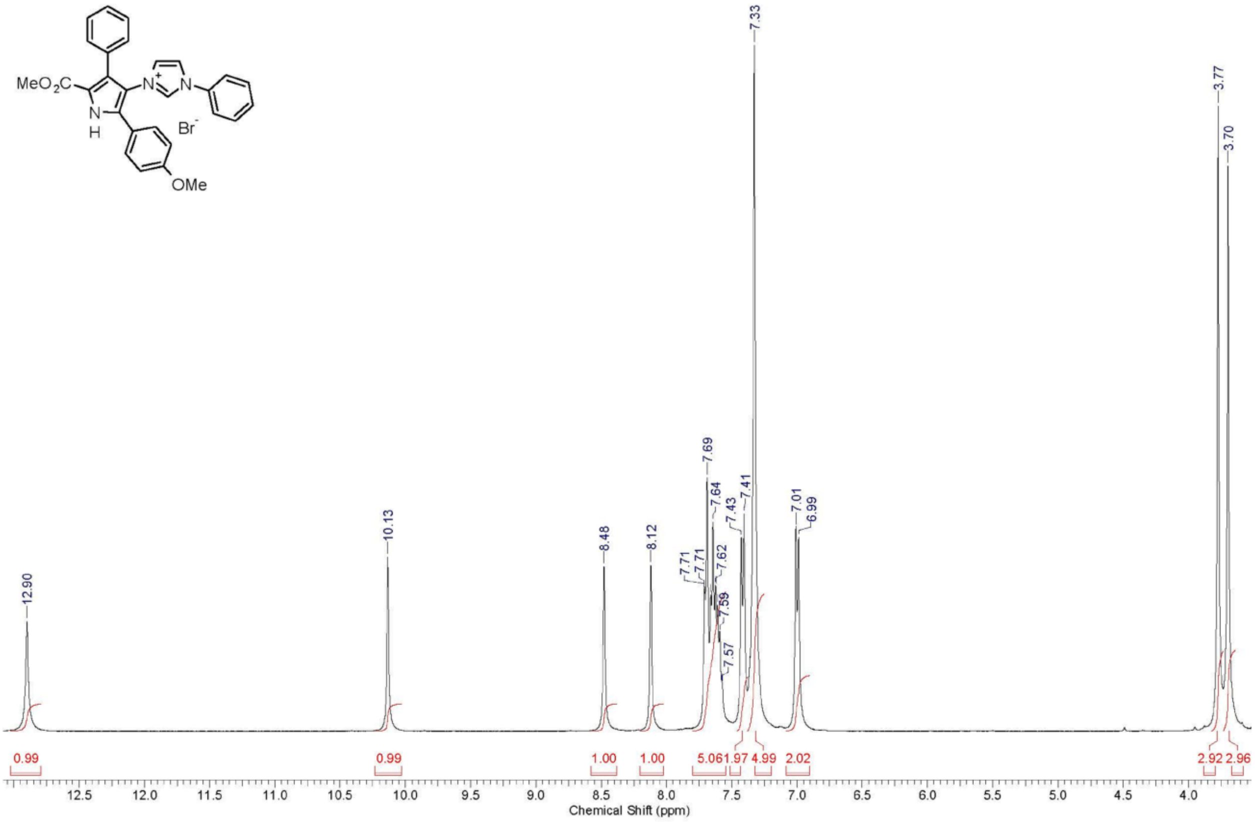

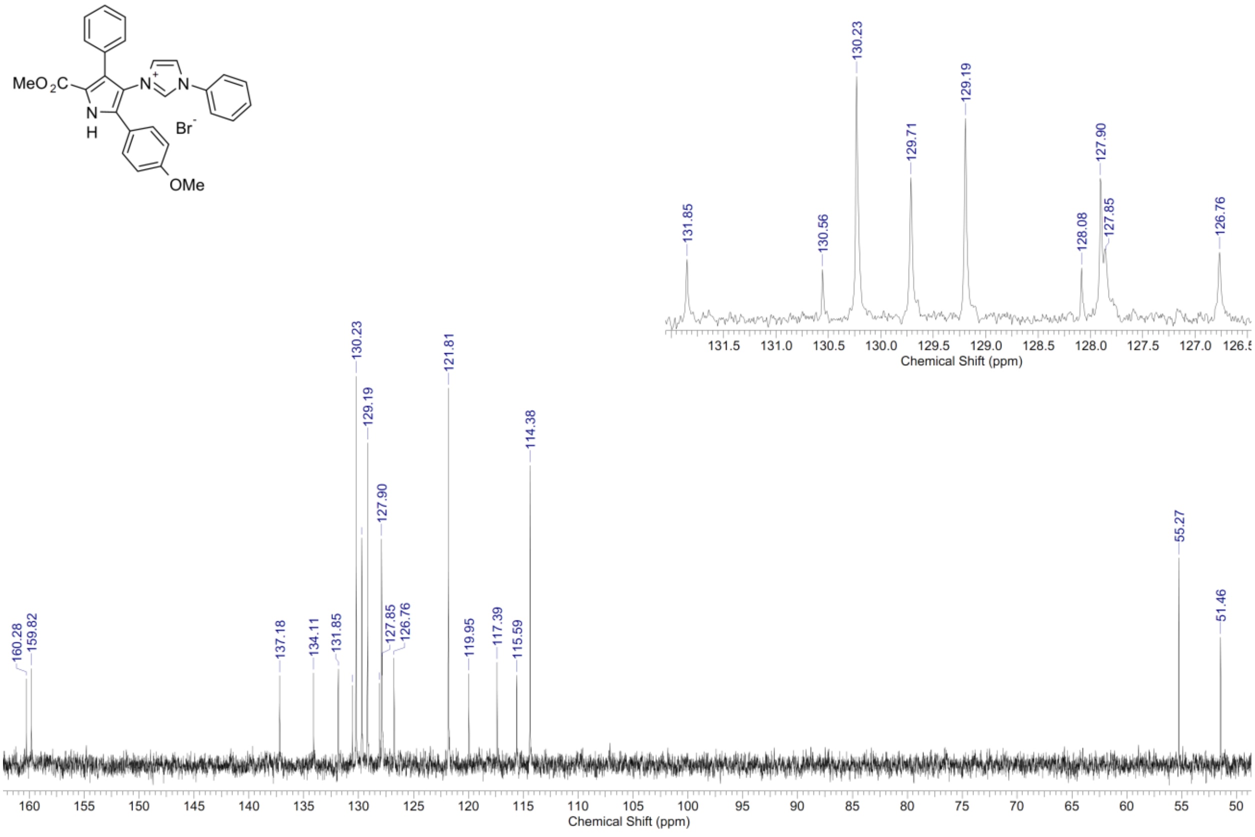

3-(2-(4-Bromophenyl)-5-methoxycarbonyl-4-phenyl-1*H*-pyrrol-3-yl)-1-phenyl-1*H*-imidazol-3-ium bromide (1g), DMSO-d_6

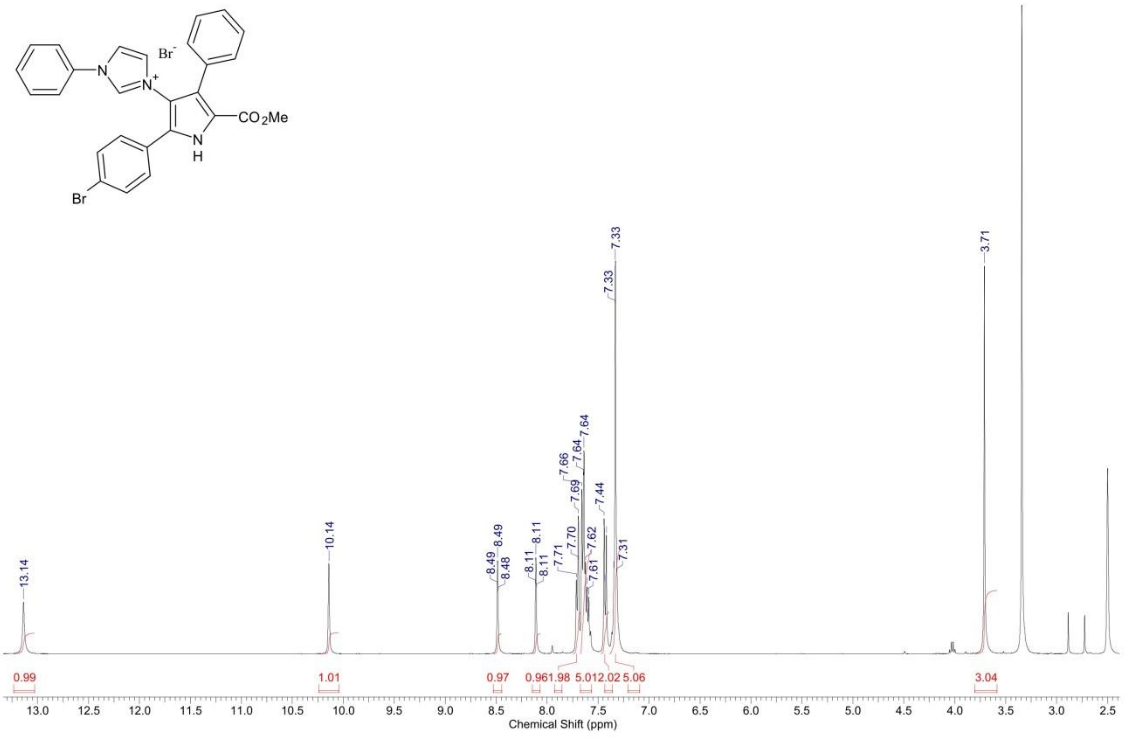

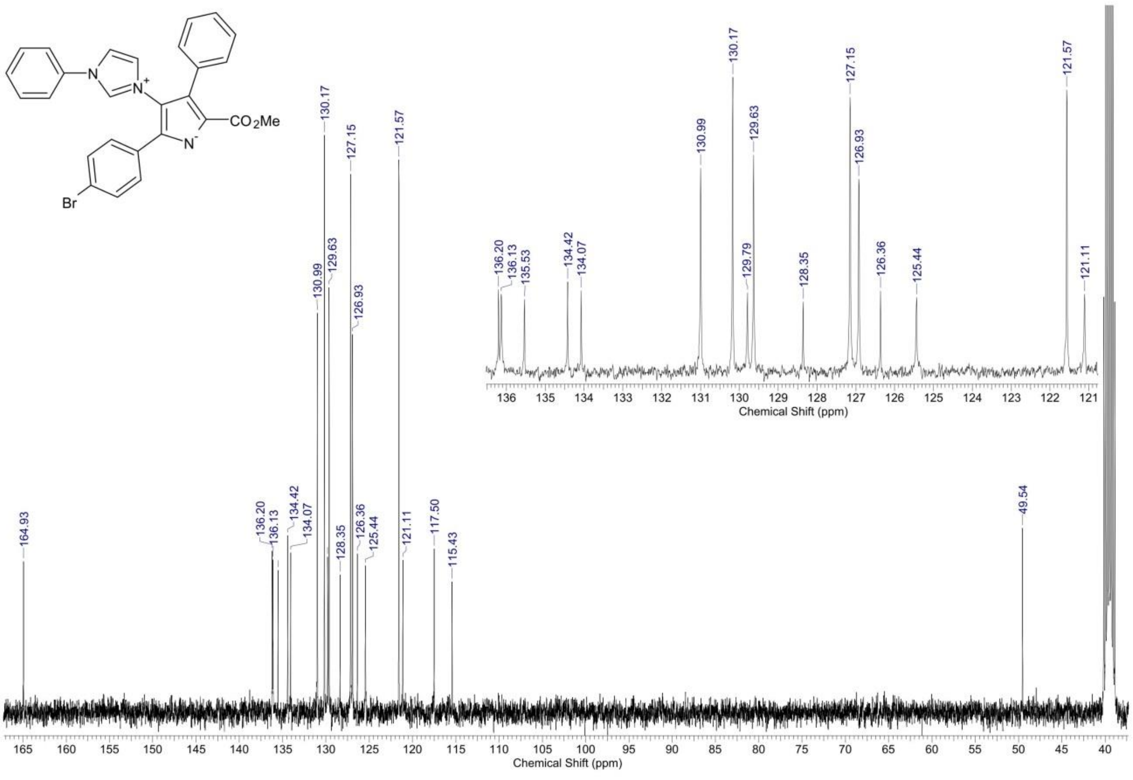

3-(5-Methoxycarbonyl-4-methyl-2-phenyl-1*H*-pyrrol-3-yl)-1-phenyl-1*H*-imidazol-3-ium bromide (1h), DMSO-d_6

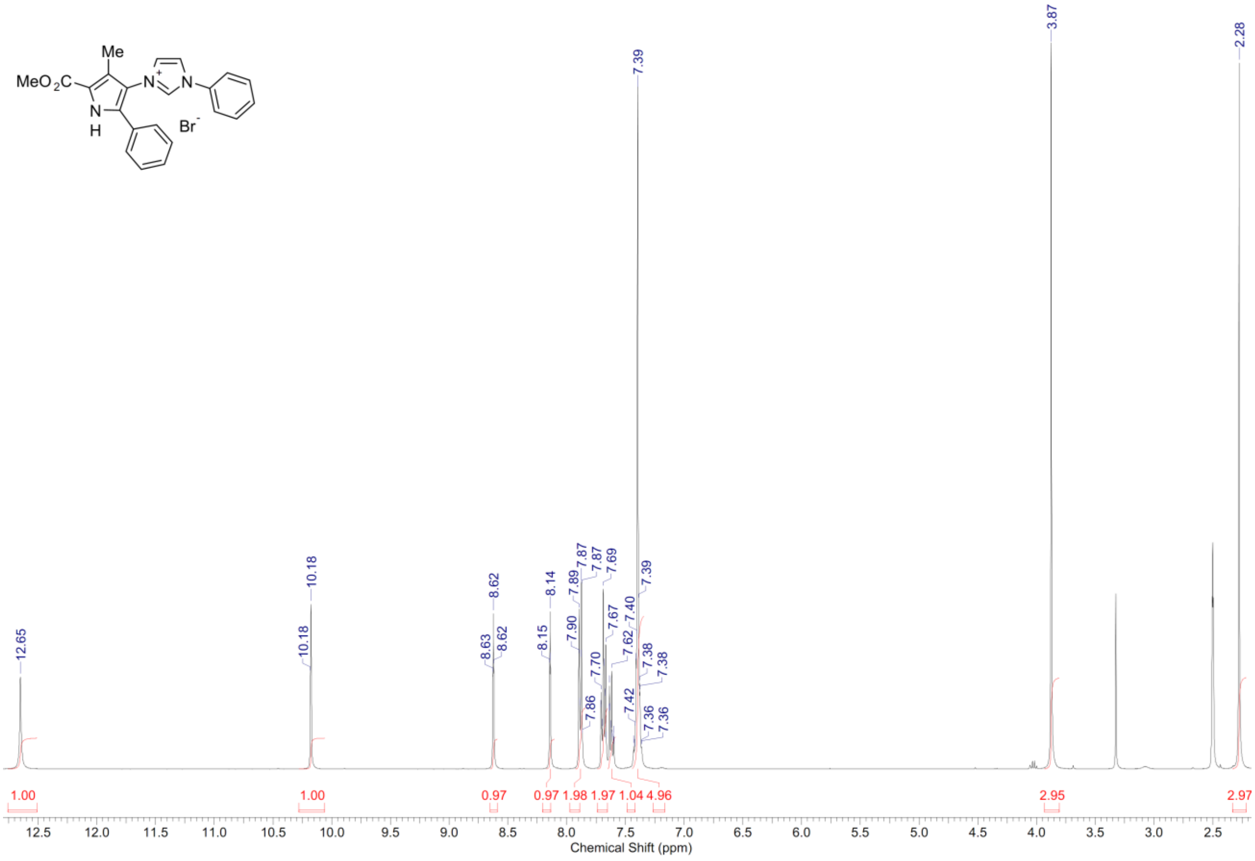

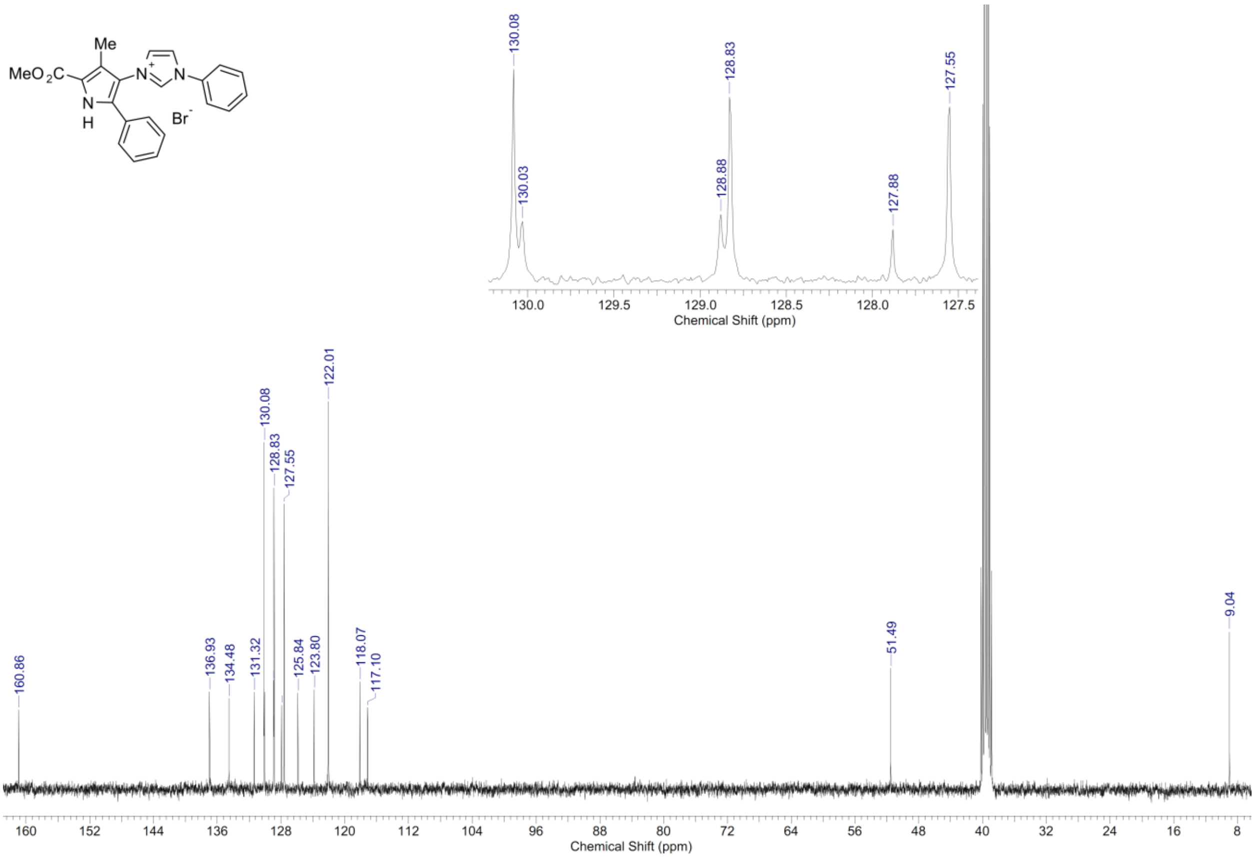

3-(5-Methoxycarbonyl-2-(4-methoxyphenyl)-4-methyl-1*H*-pyrrol-3-yl)-1-phenyl-1*H*-imidazol-3-ium bromide (1i), DMSO-d_6

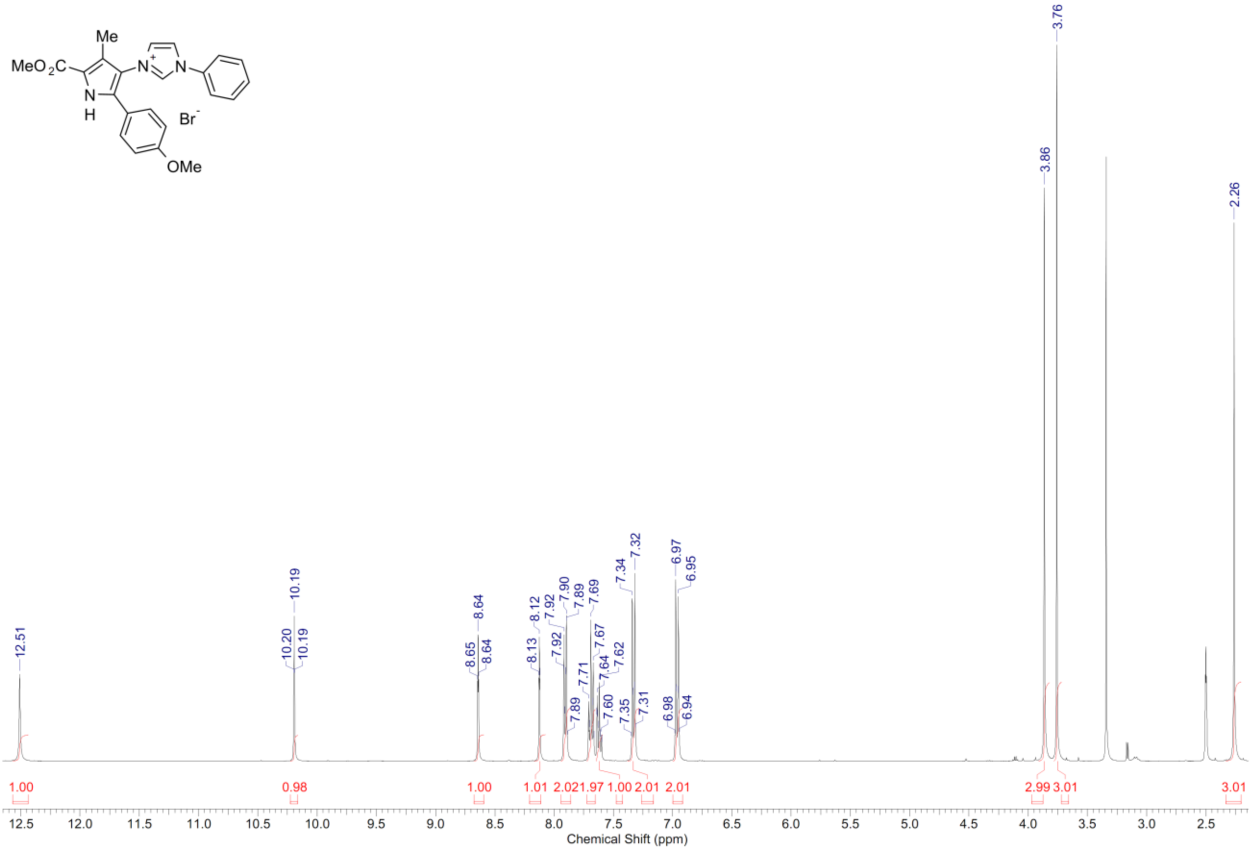

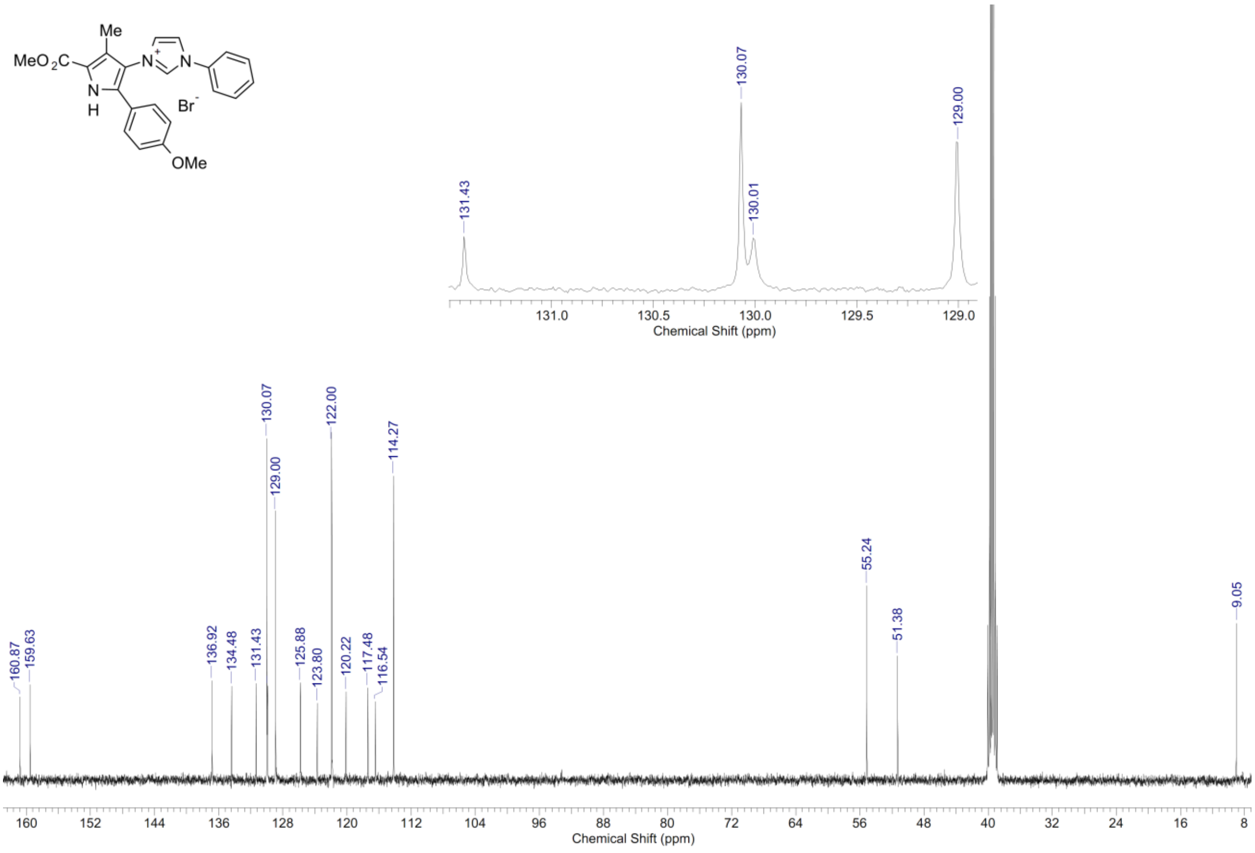

1-Benzyl-3-(5-methoxycarbonyl-2,4-diphenyl-1*H*-pyrrol-3-yl)-1*H*-imidazol-3-ium bromide (1j), DMSO-d_6

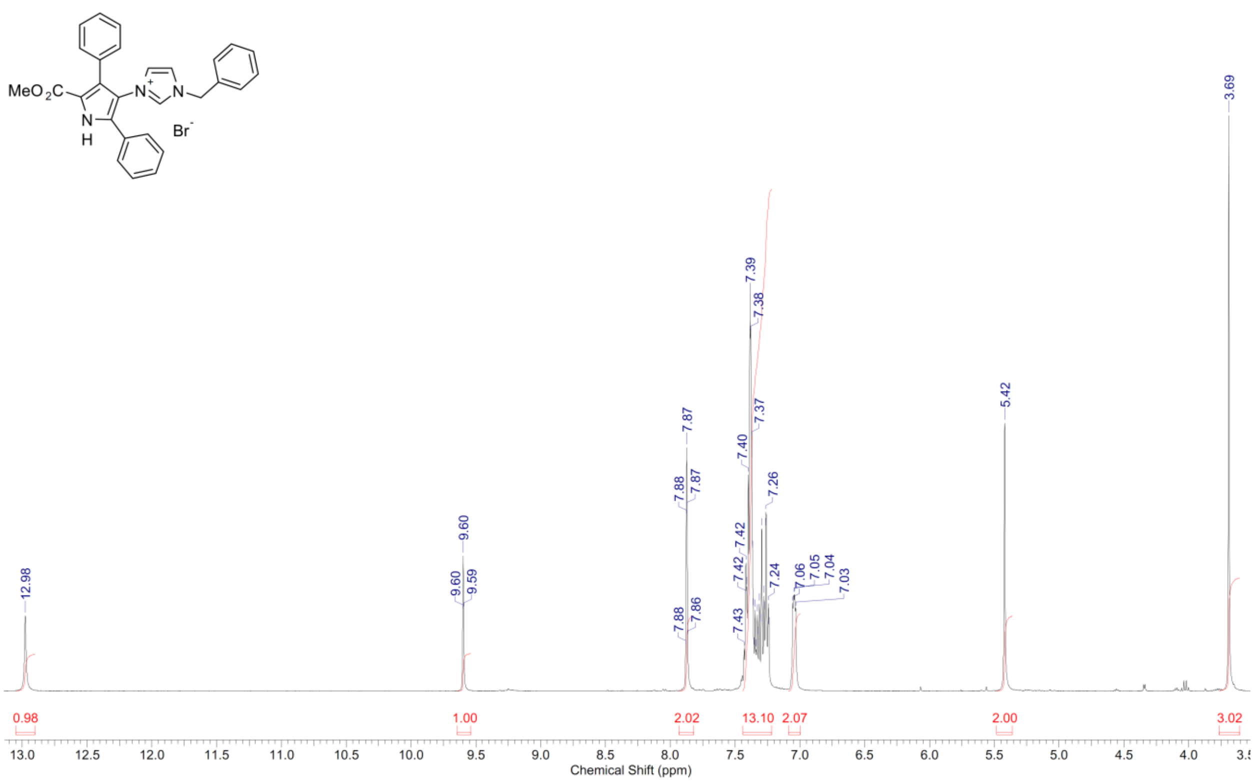

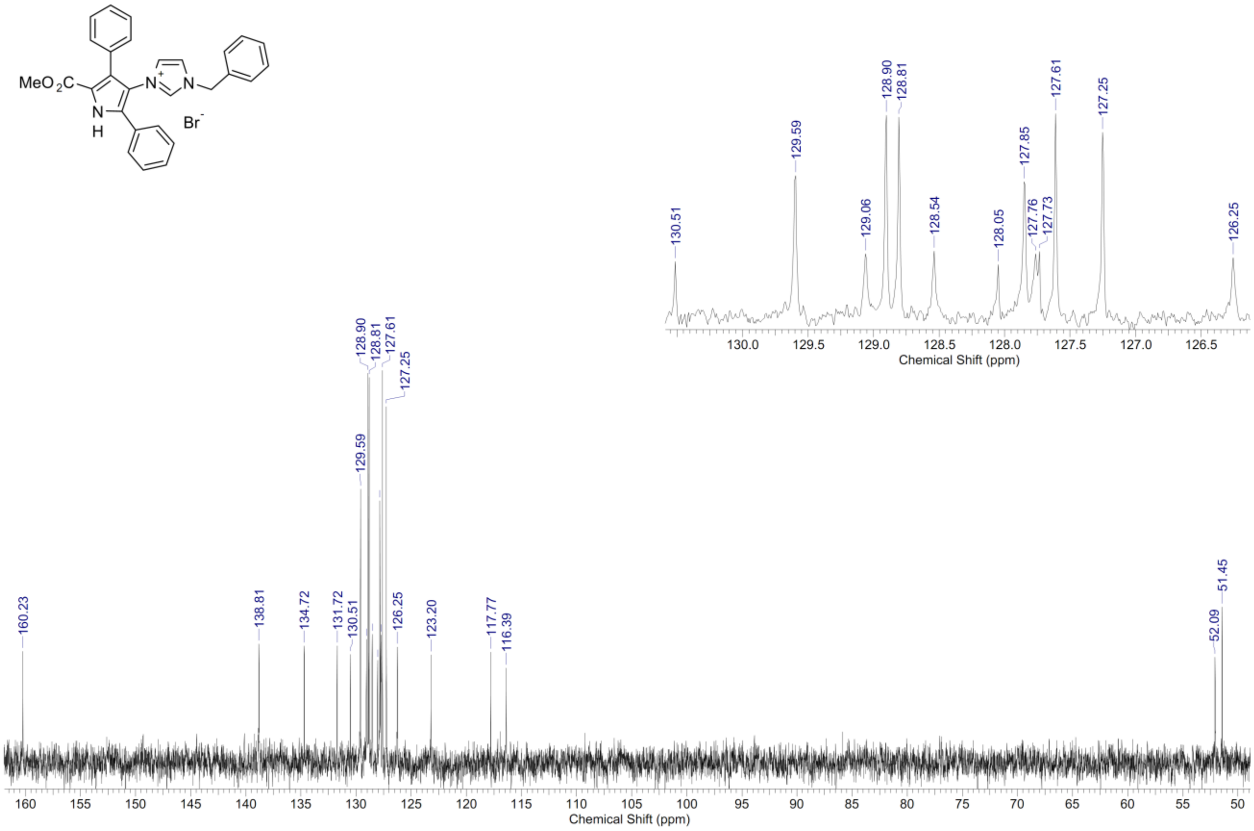

1-Benzyl-3-(5-methoxycarbonyl-2-(4-methoxyphenyl)-4-phenyl-1*H*-pyrrol-3-yl)-1*H*-imidazol-3-ium bromide (1k), DMSO-d_6

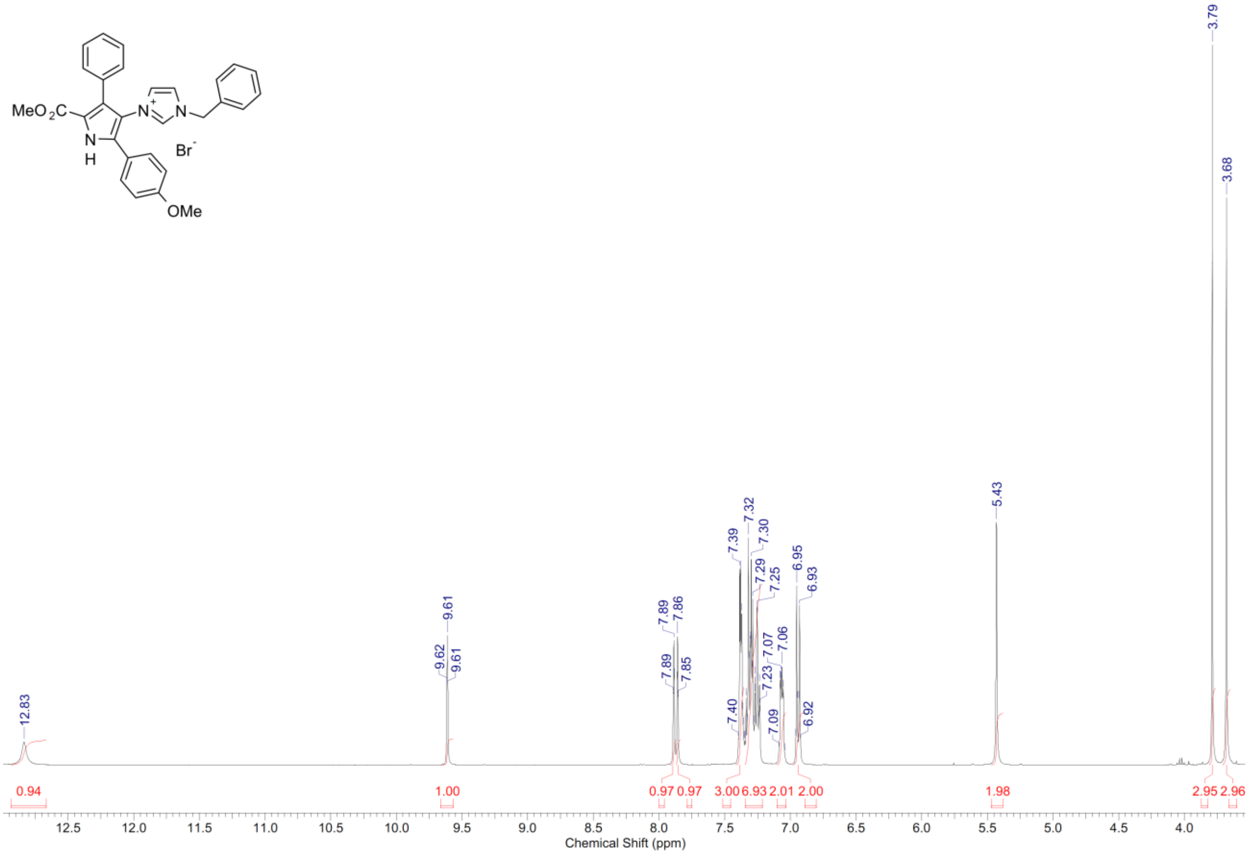

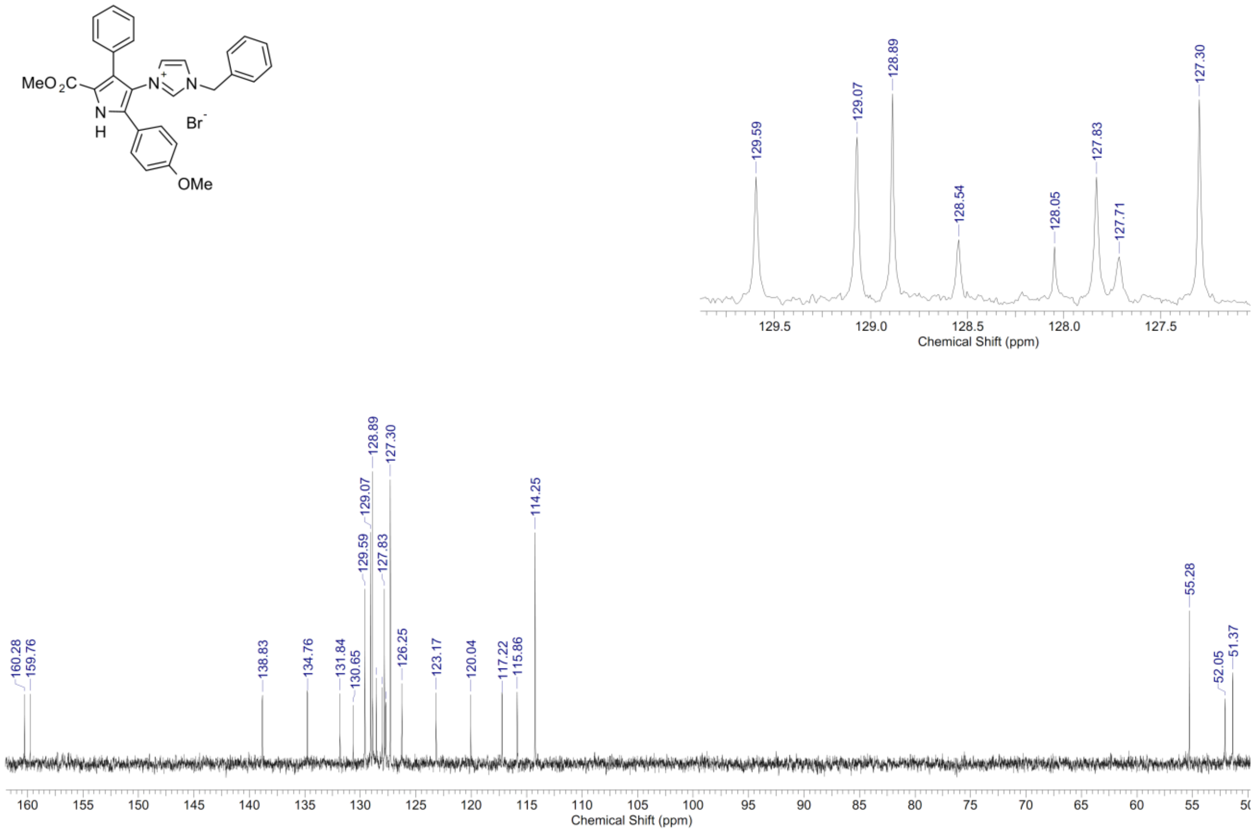

1-Benzyl-3-(2-(4-fluorophenyl)-5-methoxycarbonyl-4-phenyl-1*H*-pyrrol-3-yl)-1*H*-imidazol-3-ium bromide (1l), DMSO-d_6

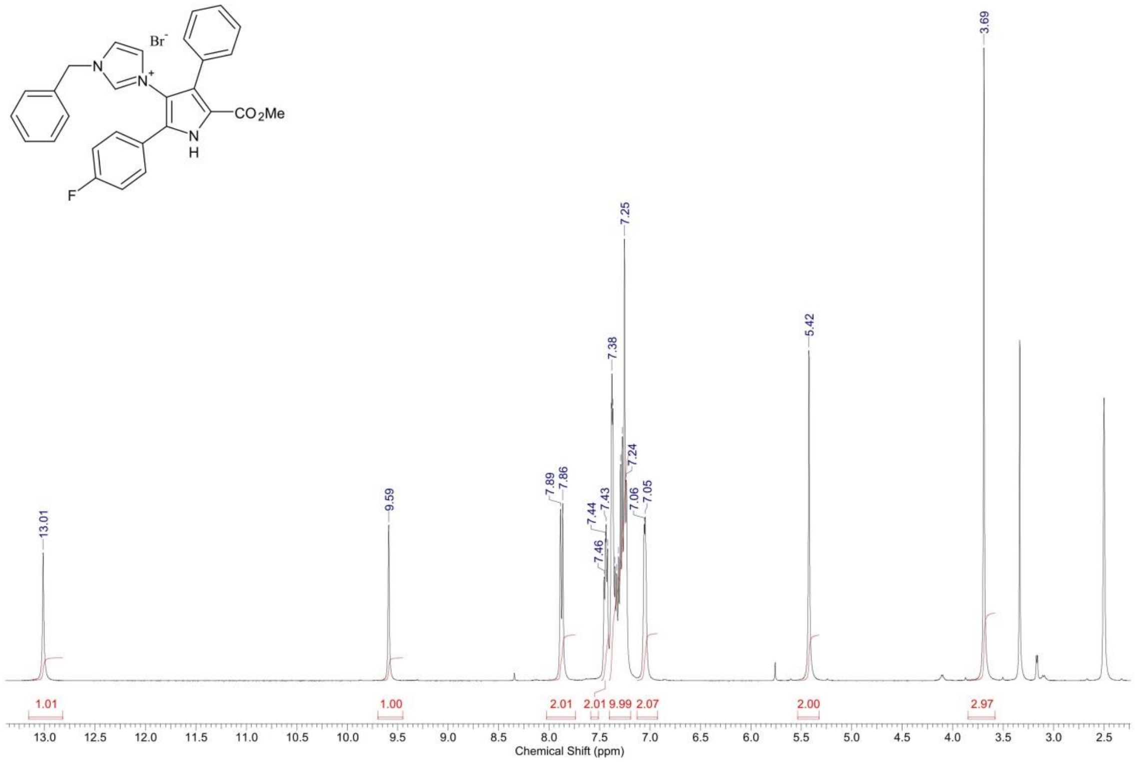

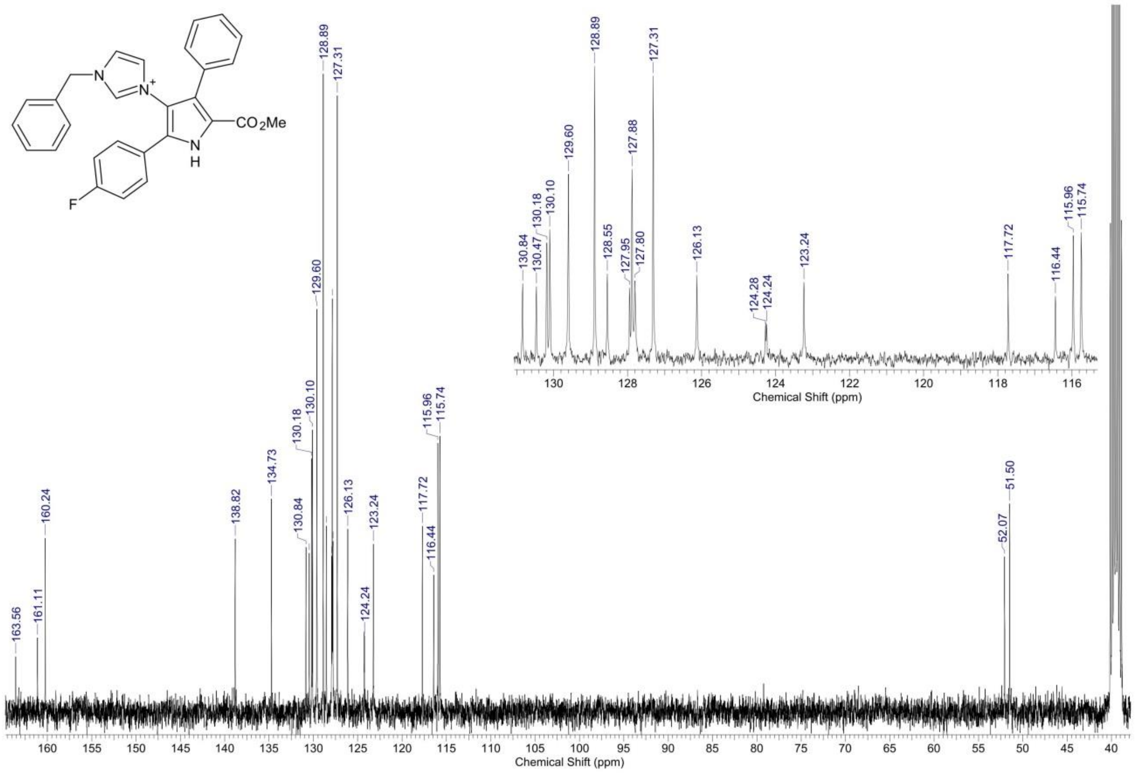

1-Benzyl-3-(2-(4-chlorophenyl)-5-methoxycarbonyl-4-phenyl-1*H*-pyrrol-3-yl)-1*H*-imidazol-3-ium bromide (1m), DMSO-d_6

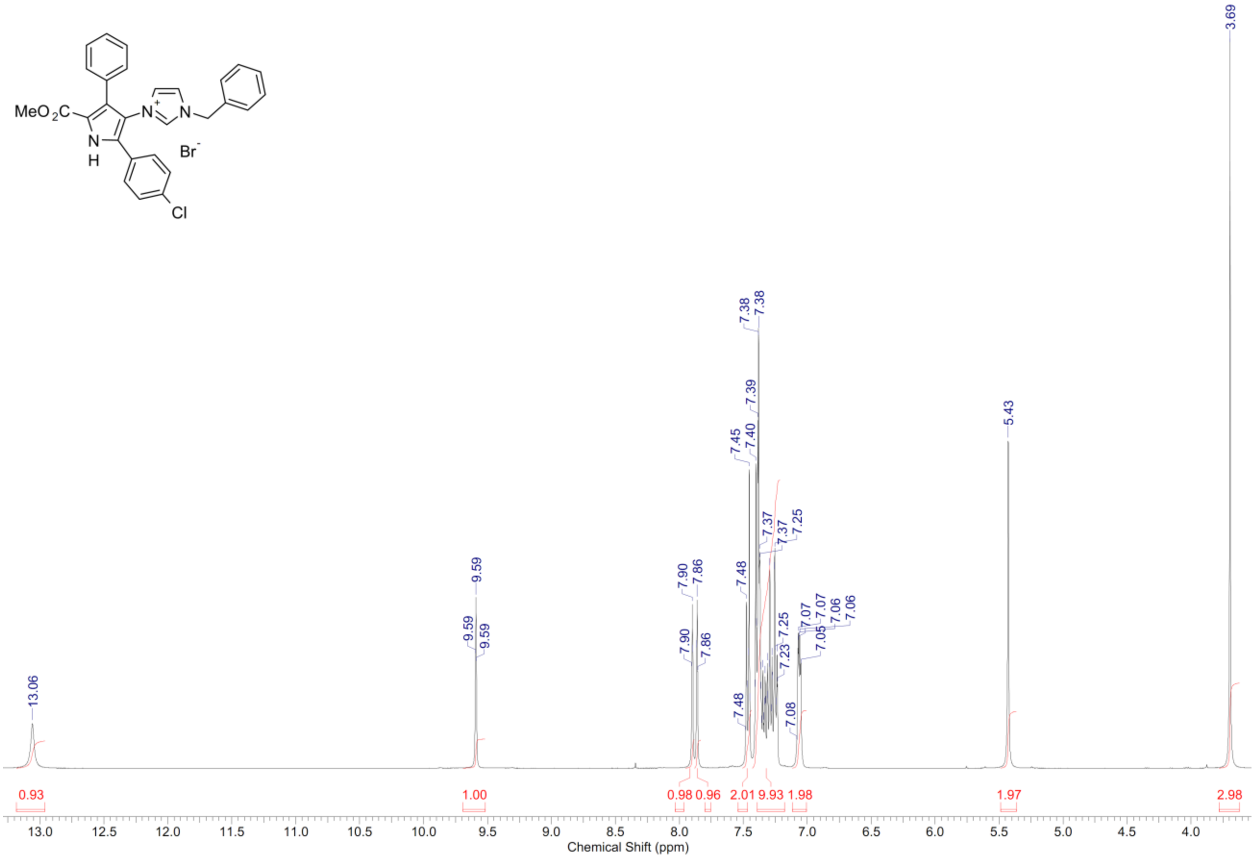

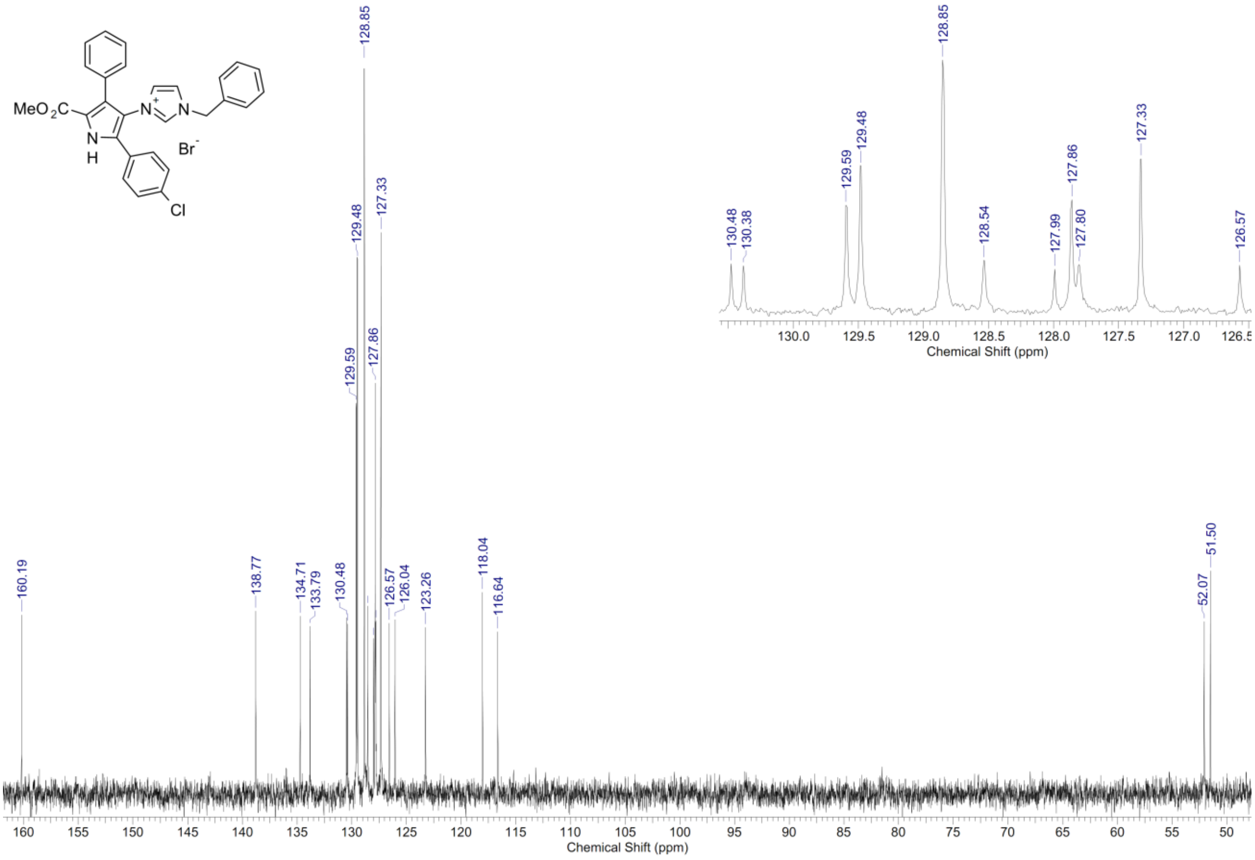

1-Benzyl-3-(5-methoxycarbonyl-4-methyl-2-phenyl-1*H*-pyrrol-3-yl)-1*H*-imidazol-3-ium bromide (1n), DMSO-d_6

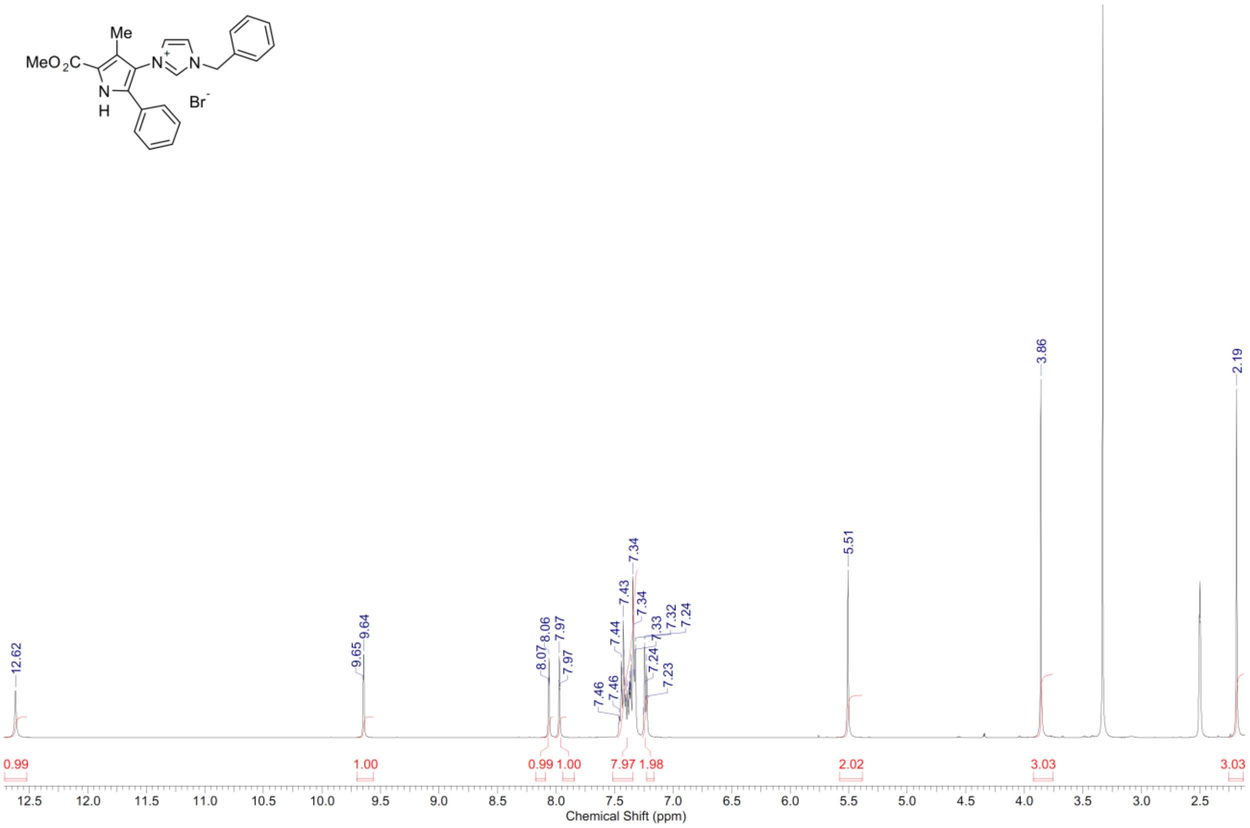

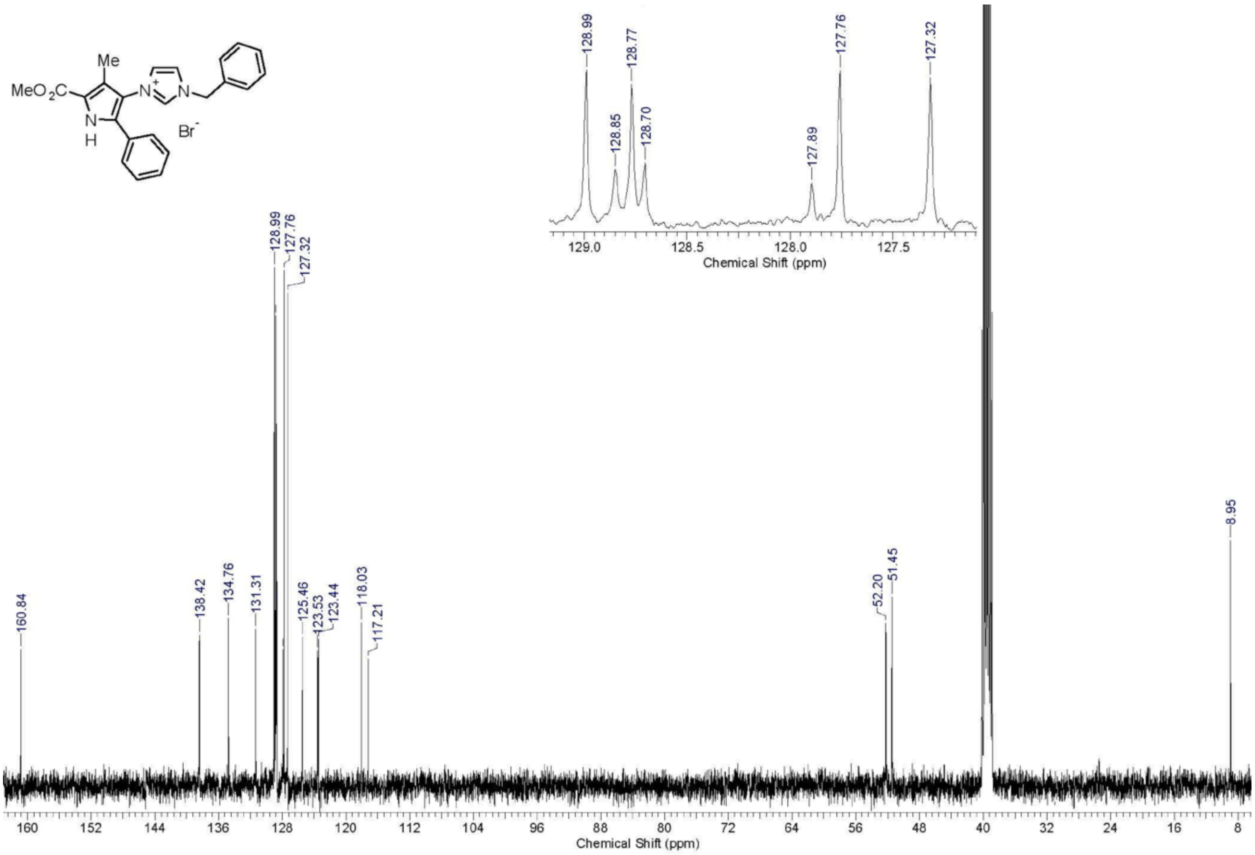

1-Benzyl-3-(2-(4-chlorophenyl)-5-methoxycarbonyl-4-methyl-1*H*-pyrrol-3-yl)-1*H*-imidazol-3-ium bromide (1o), DMSO-d_6

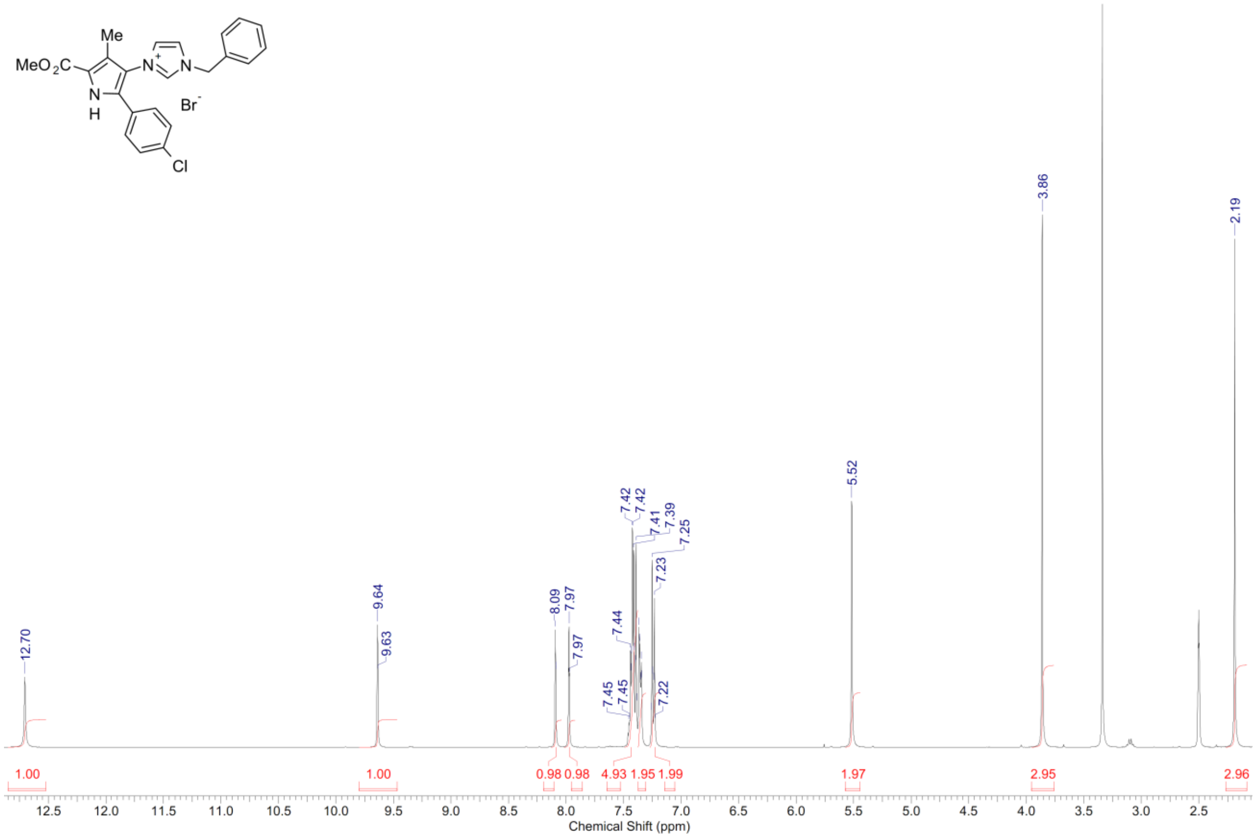

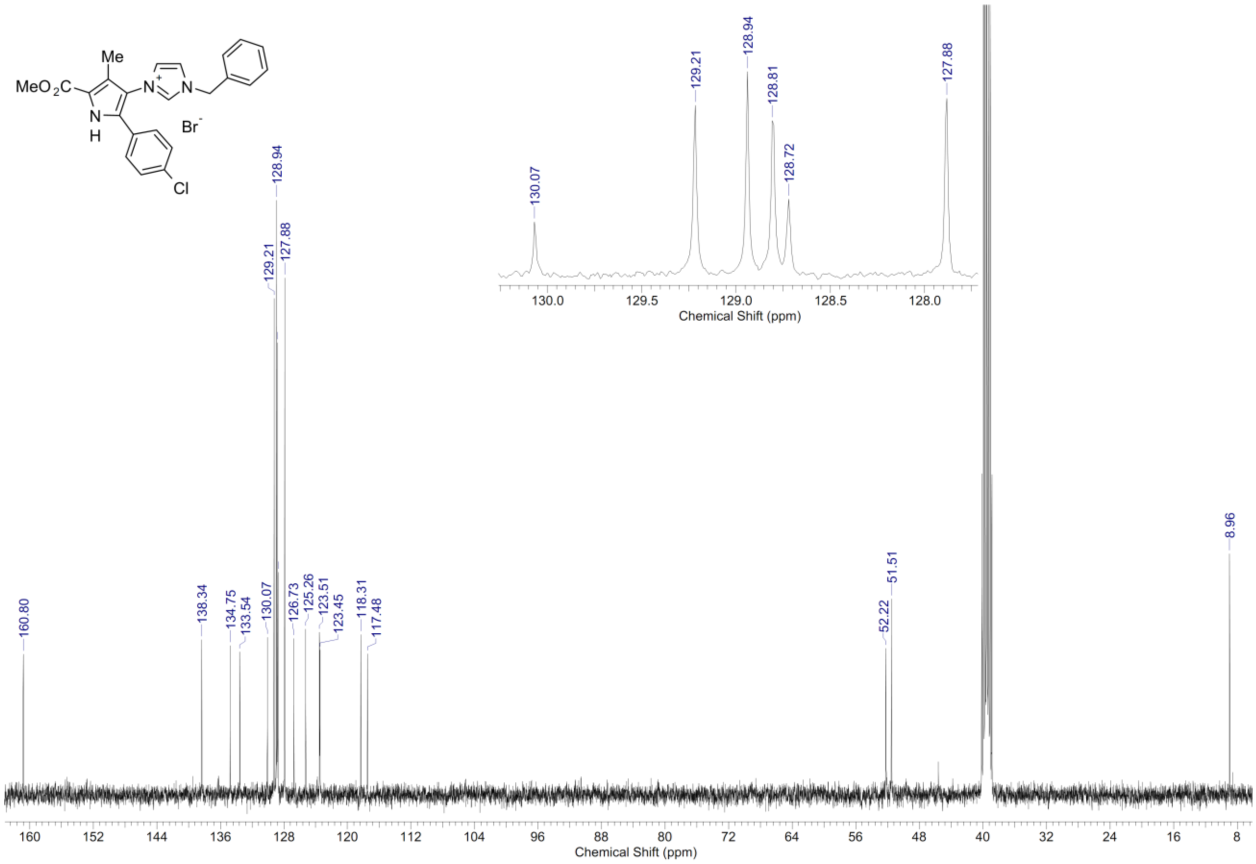

Methyl 4-(1*H*-imidazol-1-yl)-3,5-diphenyl-1*H*-pyrrole-2-carboxylate (12a), DMSO-d_6

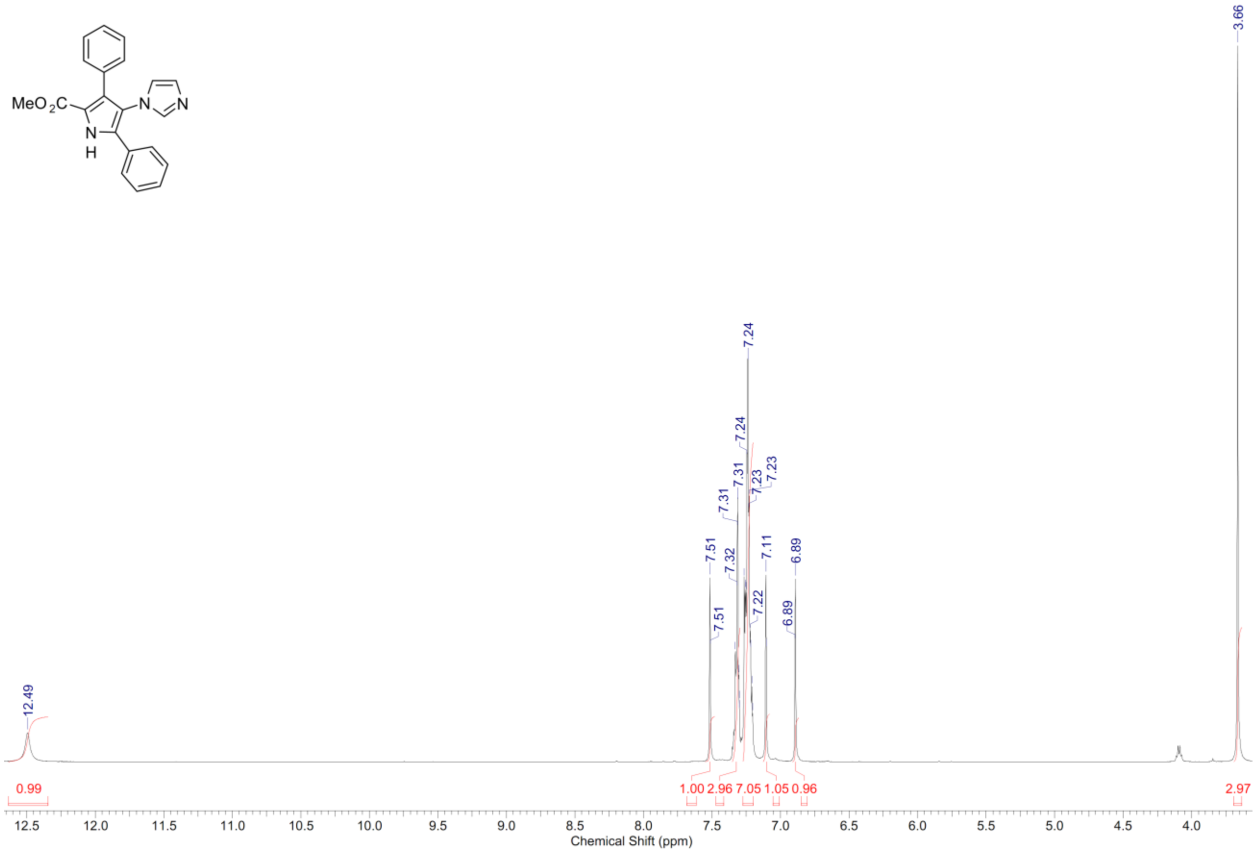

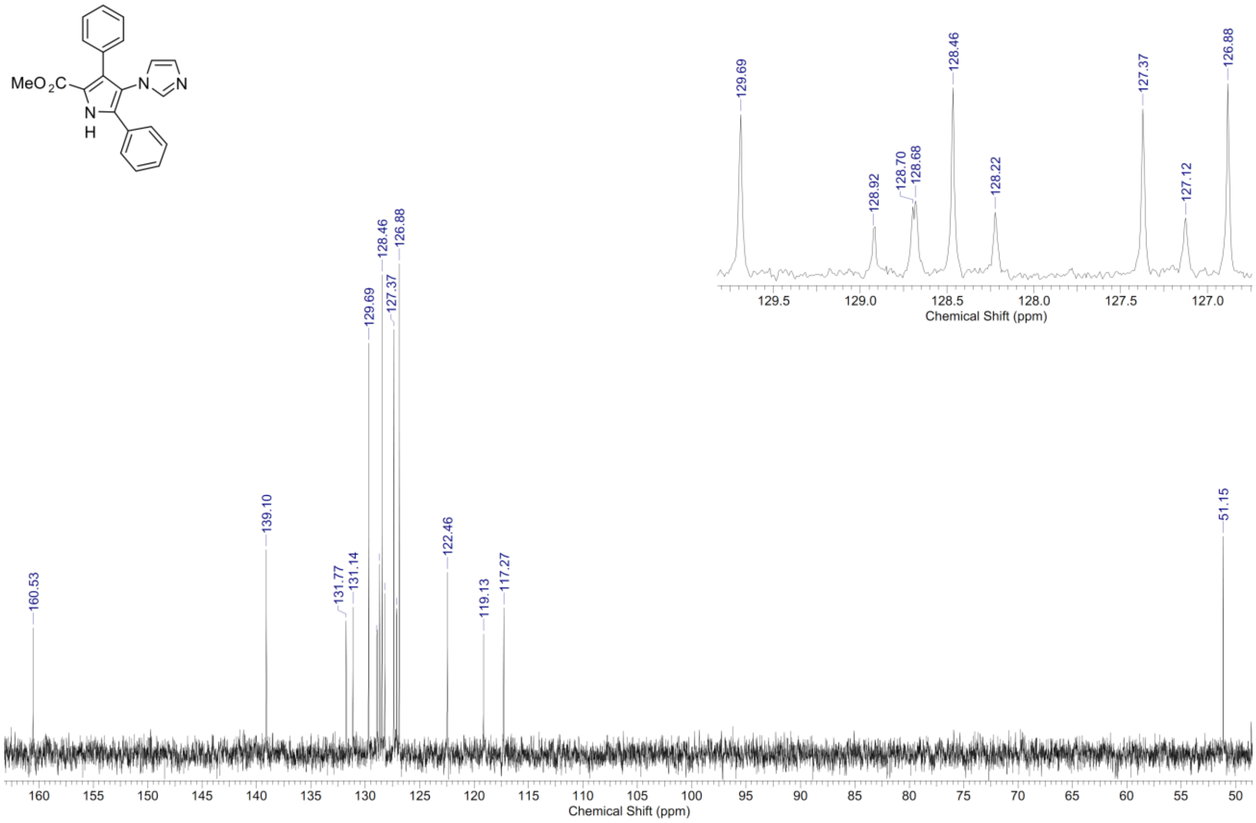

Methyl 4-(1*H*-imidazol-1-yl)-5-(4-methoxyphenyl)-3-phenyl-1*H*-pyrrole-2-carboxylate (12b), DMSO-d_6

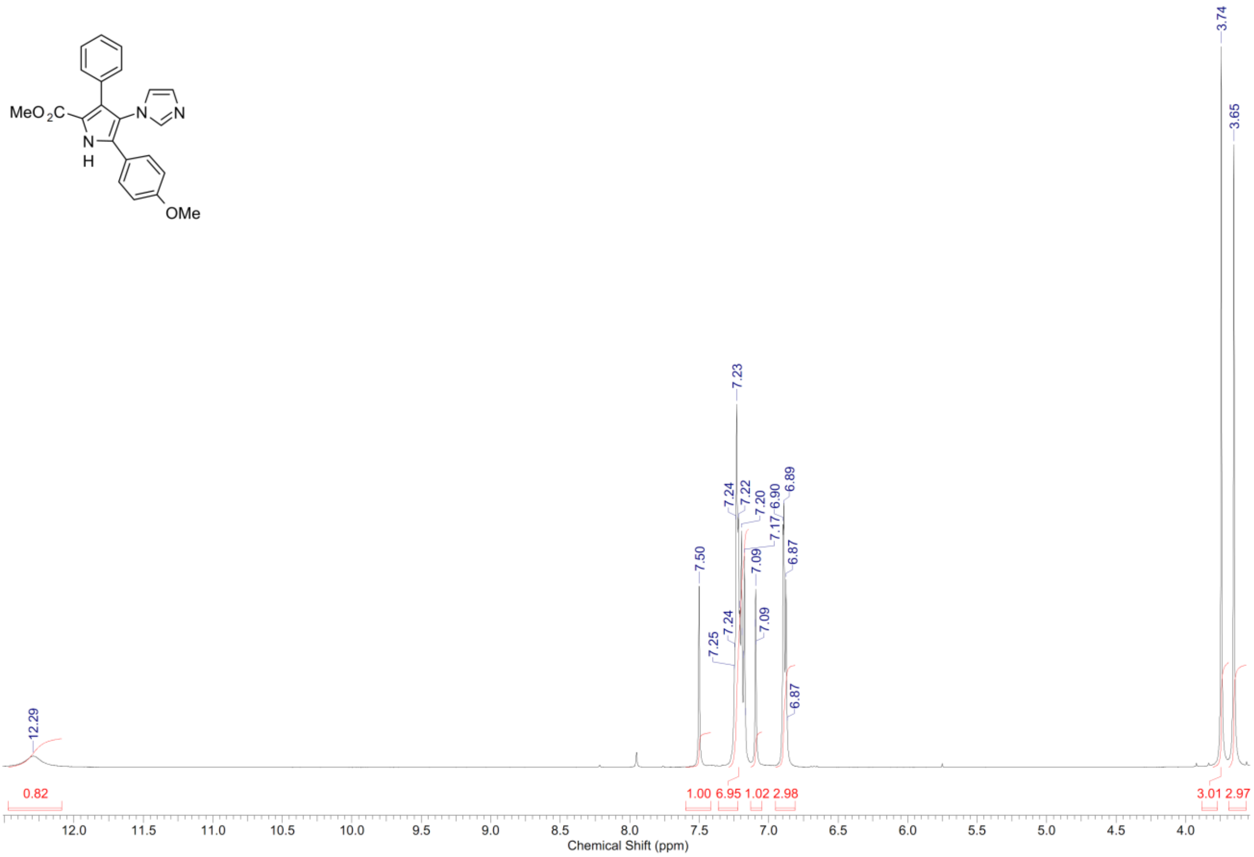

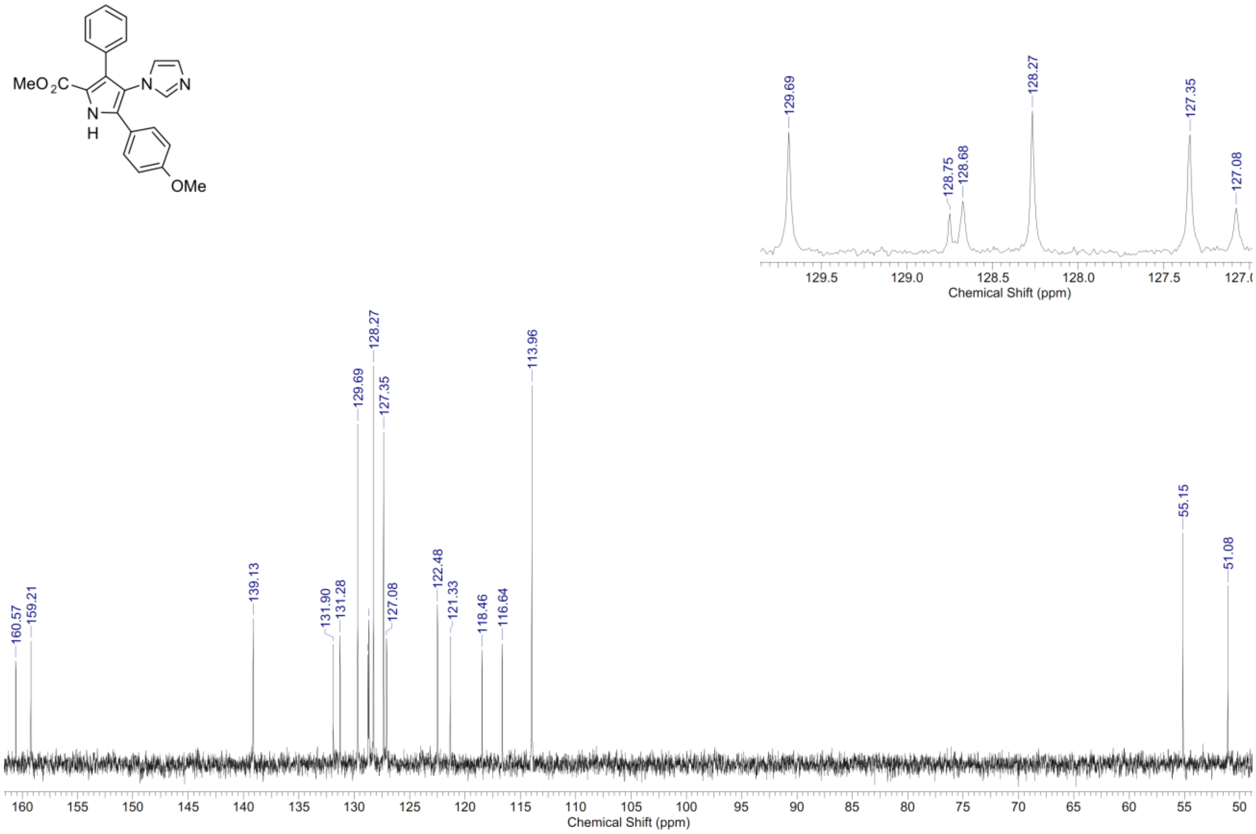

Methyl 4-(1*H*-imidazol-1-yl)-3-methyl-5-phenyl-1*H*-pyrrole-2-carboxylate (12c), DMSO-d_6

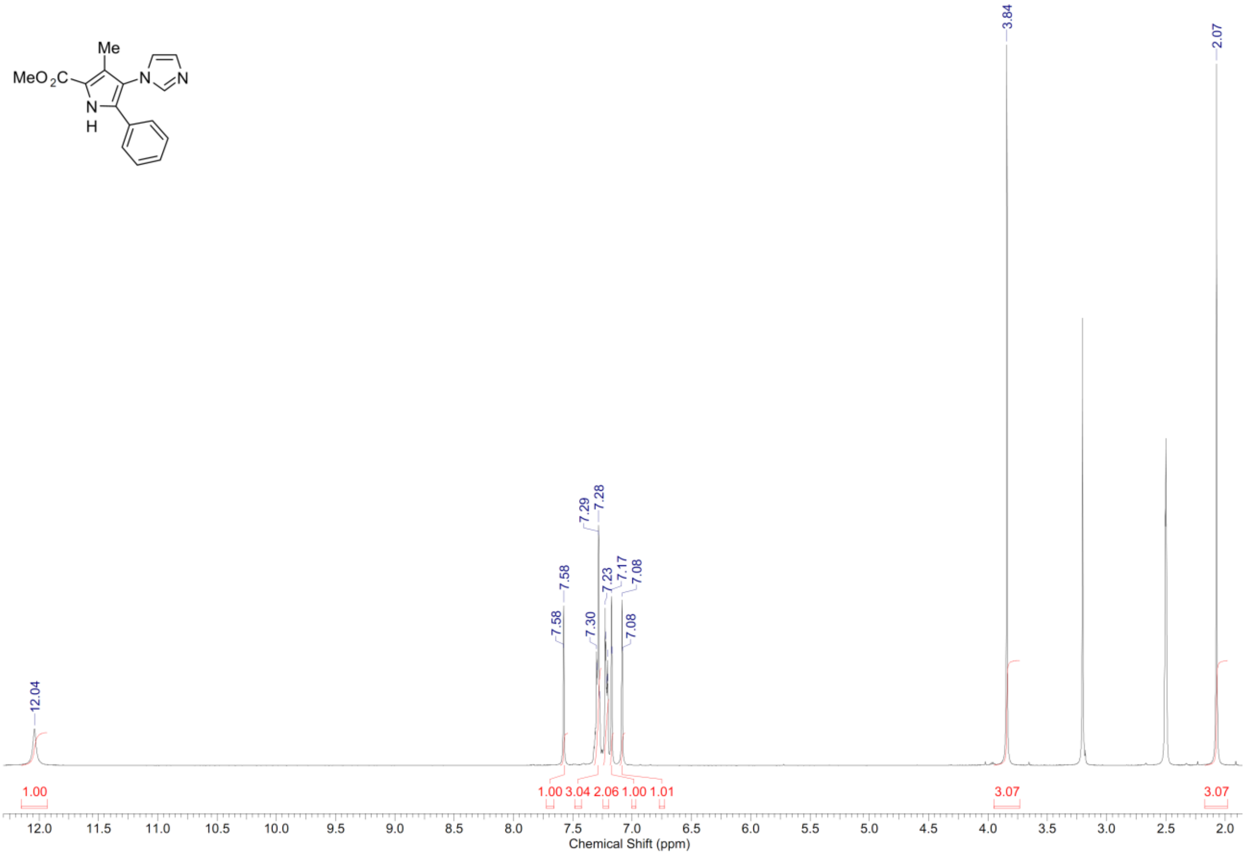

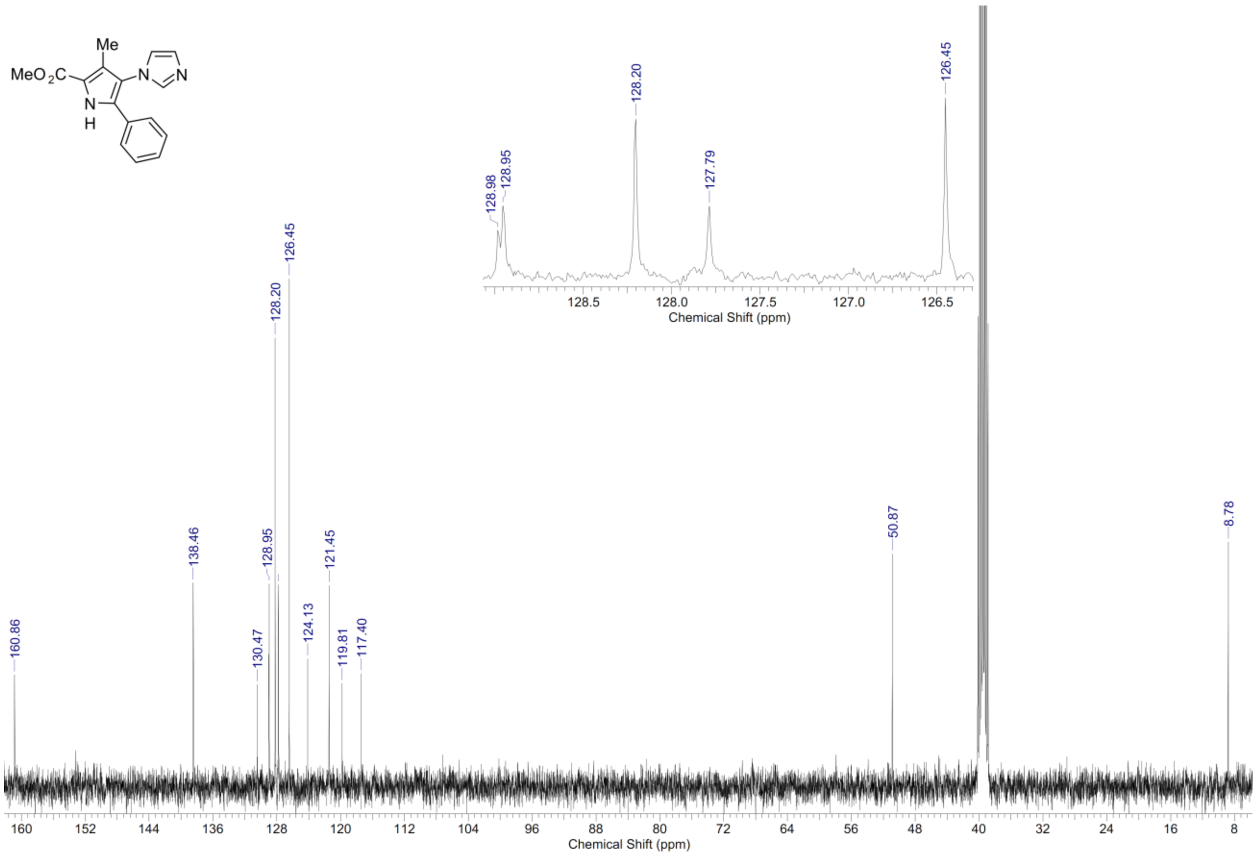

Methyl 5-(4-fluorophenyl)-4-(1*H*-imidazol-1-yl)-3-phenyl-1*H*-pyrrole-2-carboxylate (12d), DMSO-d_6

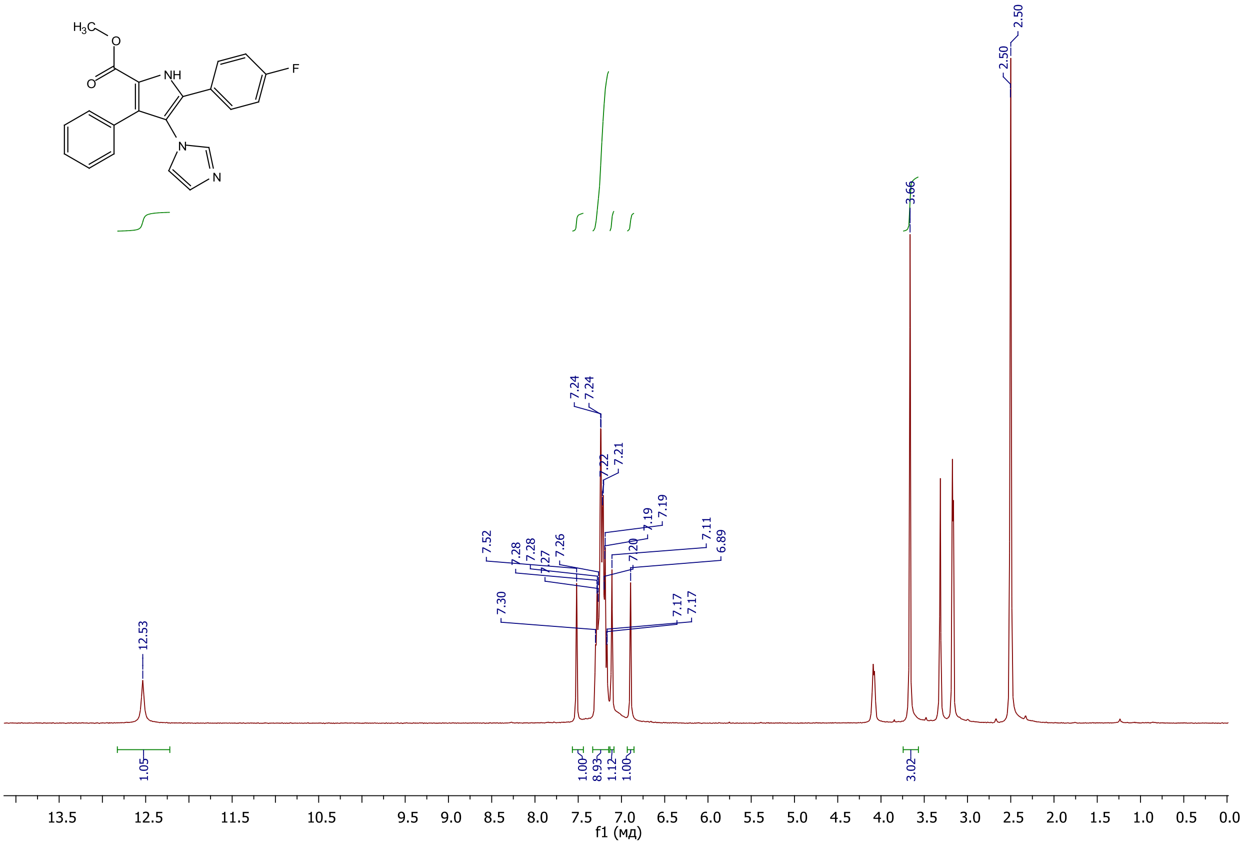

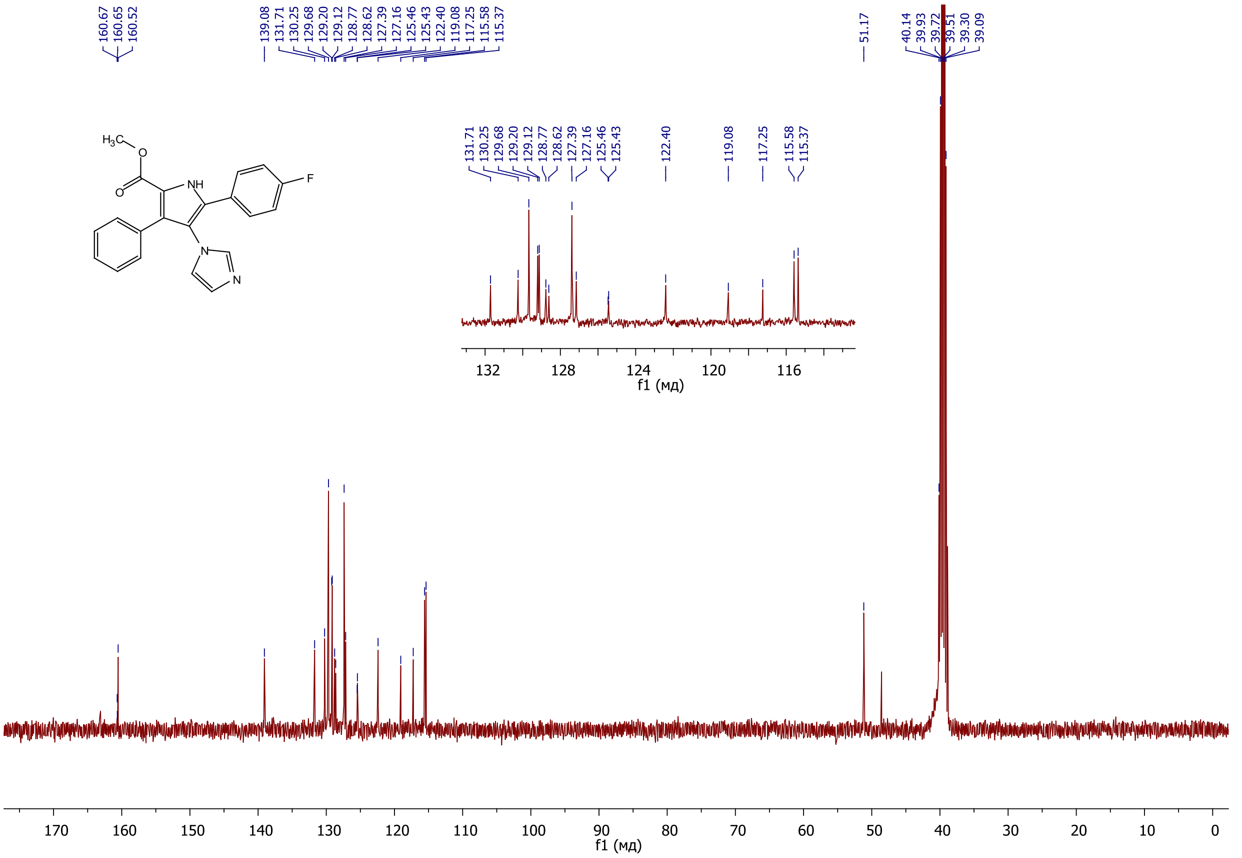

2-Methoxycarbonyl-4-(1-methyl-1*H*-imidazol-3-ium-3-yl)-3,5-diphenylpyrrol-1-ide (2a), DMSO-d_6

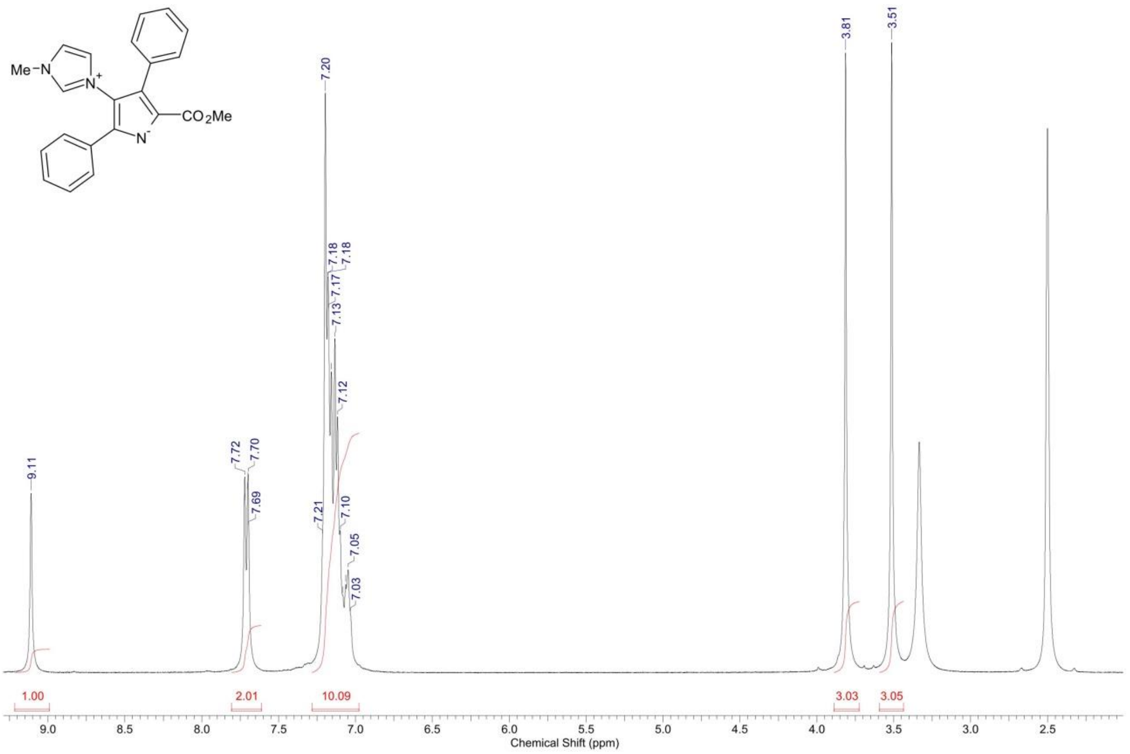

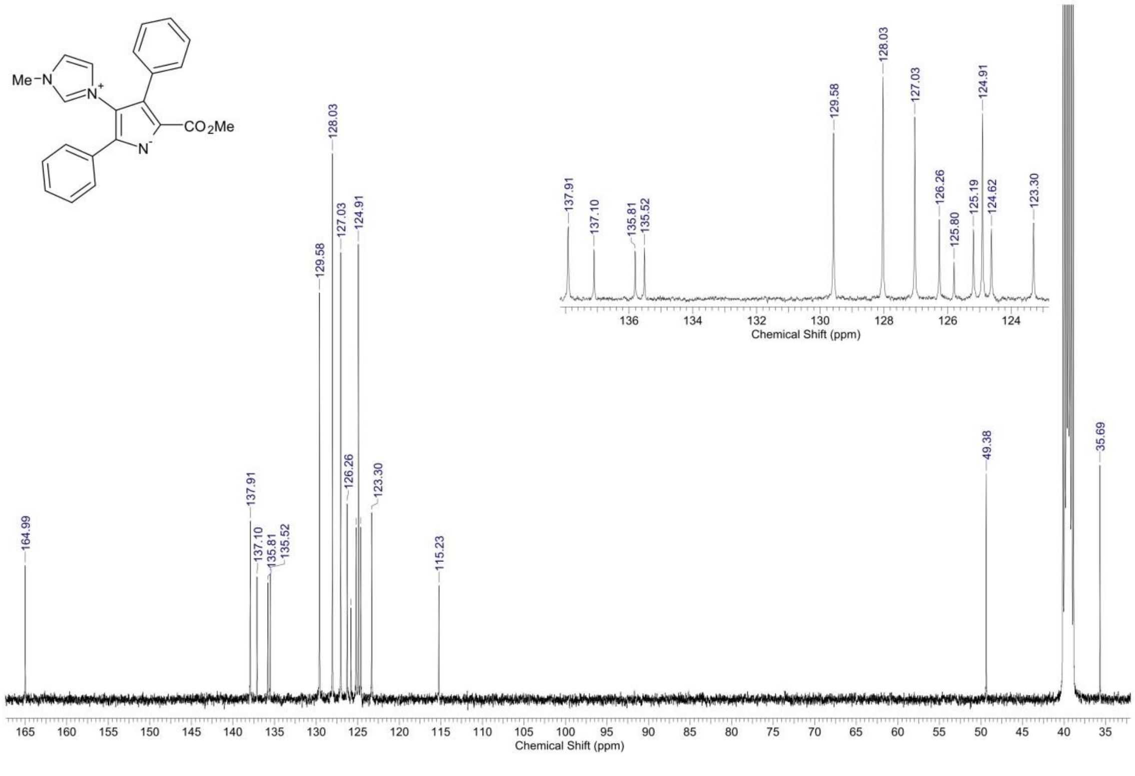

2-(4-Chlorophenyl)-5-methoxycarbonyl-3-(1-methyl-1*H*-imidazol-3-ium-3-yl)-4-phenylpyrrol-1-ide (2b), DMSO-d_6

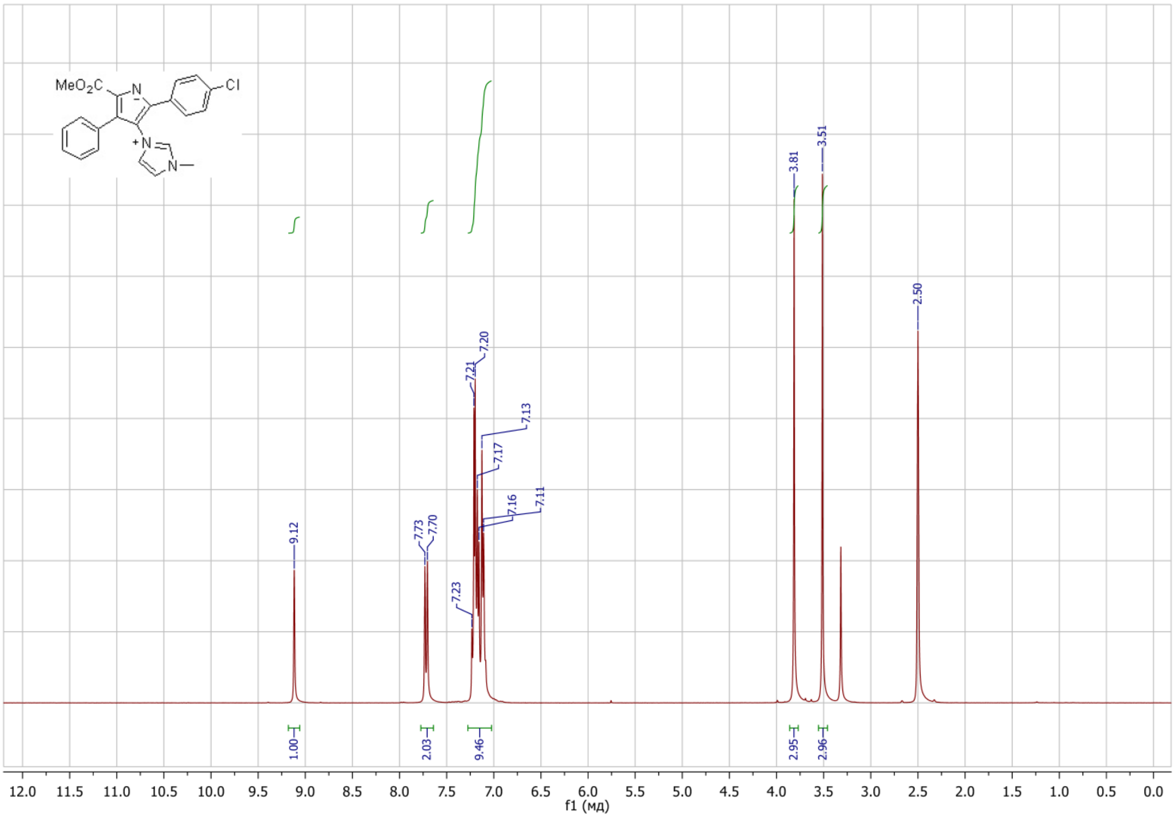

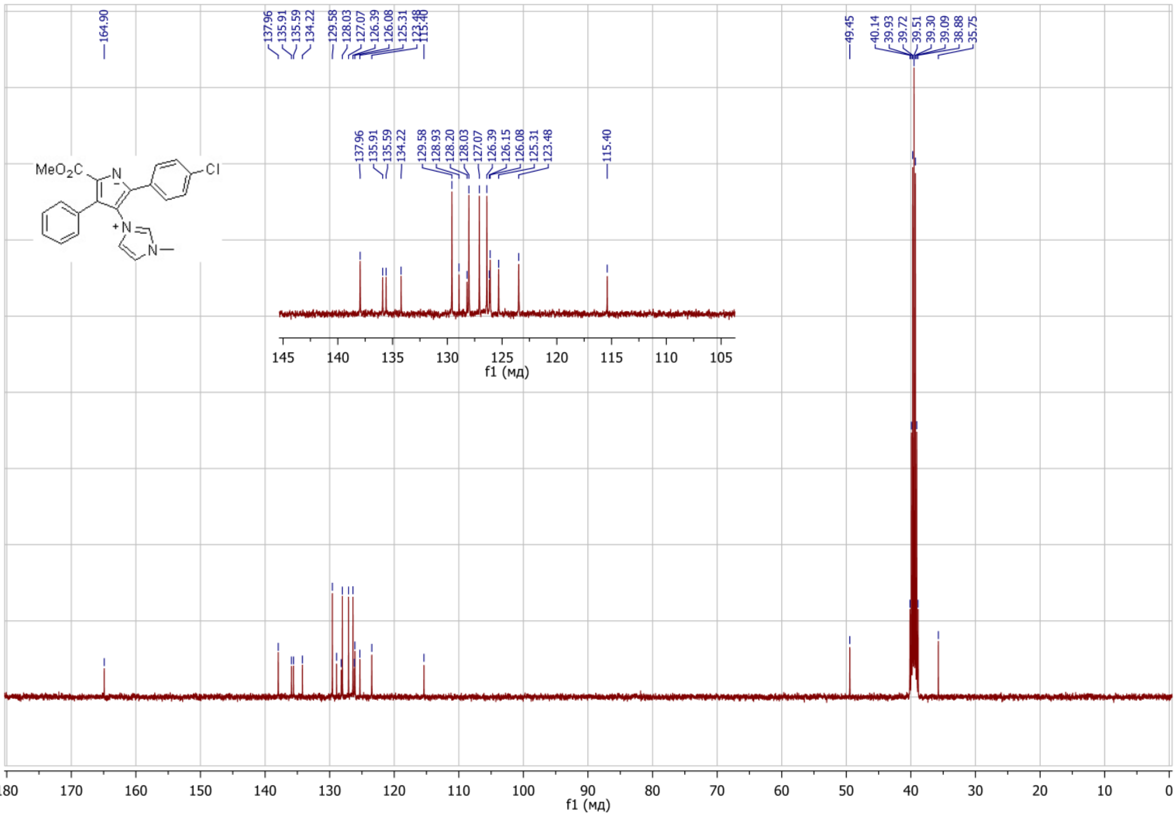

2-Methoxycarbonyl-4-(1-methyl-1*H*-imidazol-3-ium-3-yl)-5-(4-nitrophenyl)-3-phenylpyrrol-1-ide (2c), DMSO-d_6

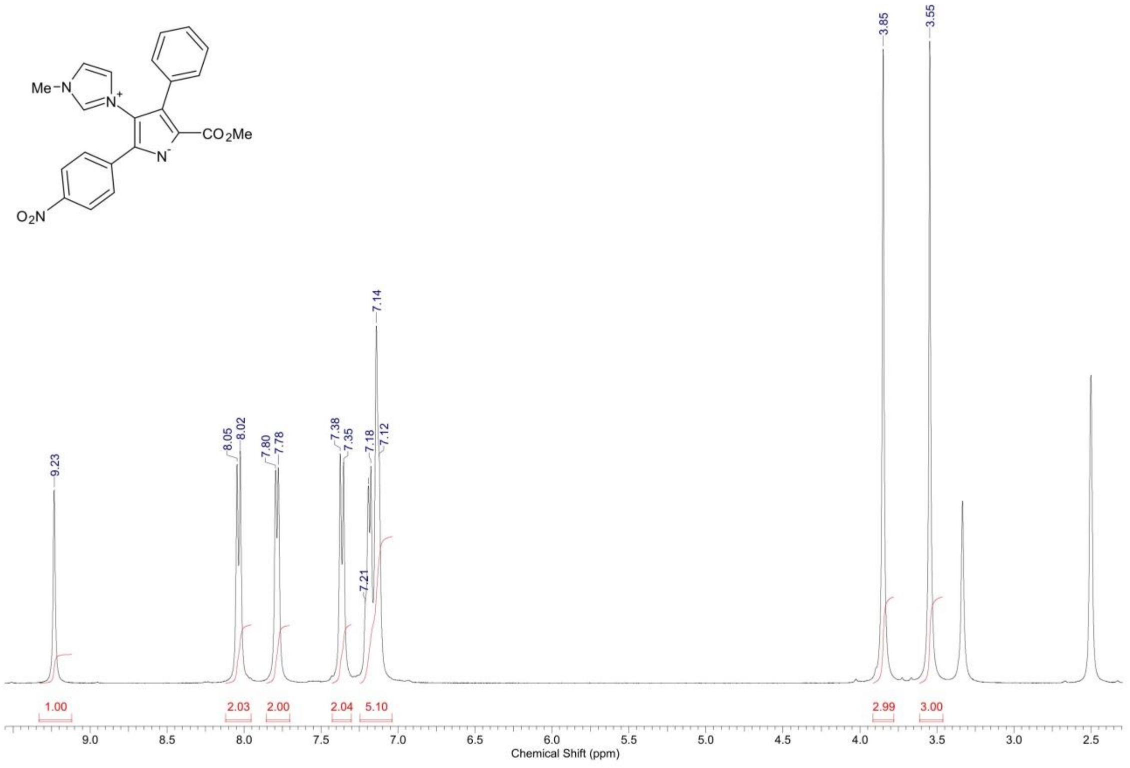

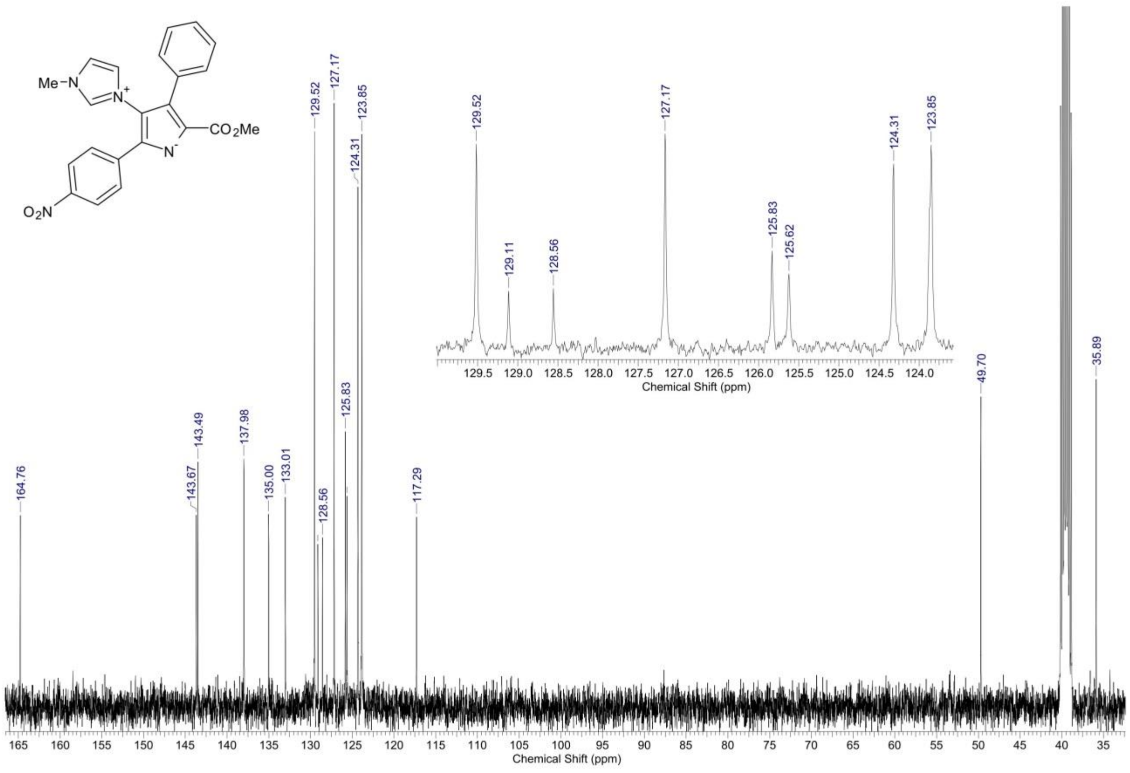

2-(3-Bromophenyl)-4-(4-bromophenyl)-5-methoxycarbonyl-3-(1-methyl-1*H*-imidazol-3-ium-3-yl)pyrrol-1-ide (2d), DMSO-d_6

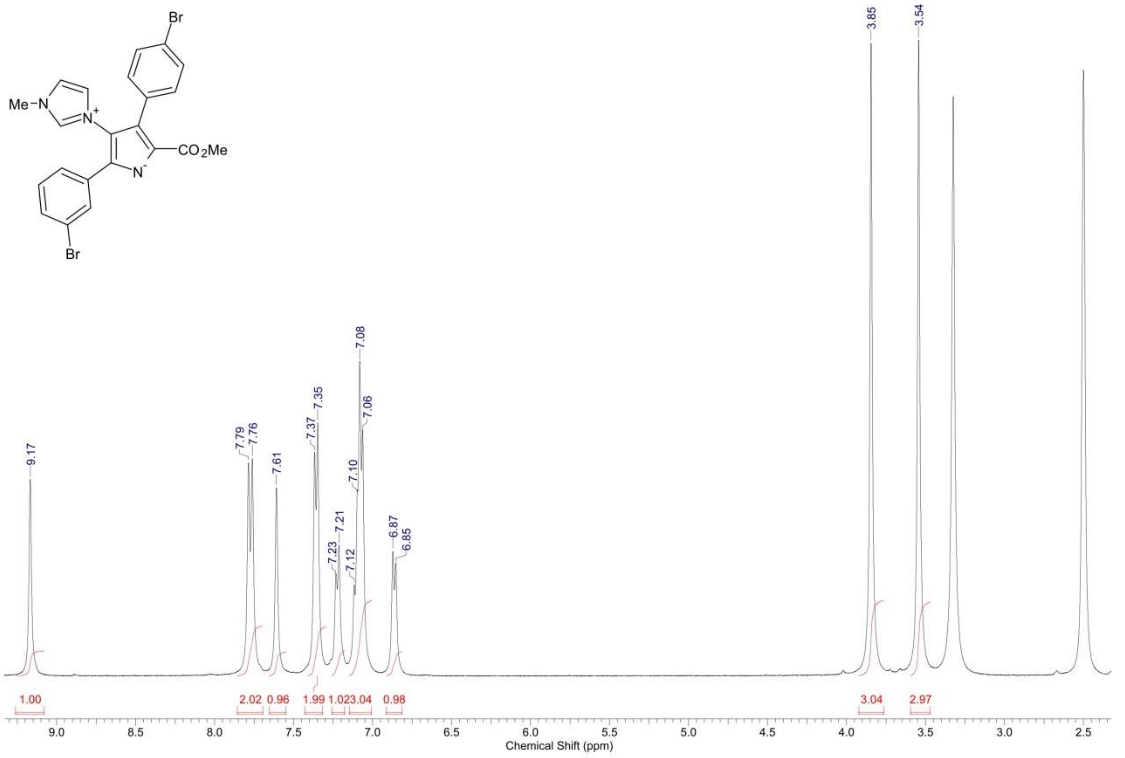

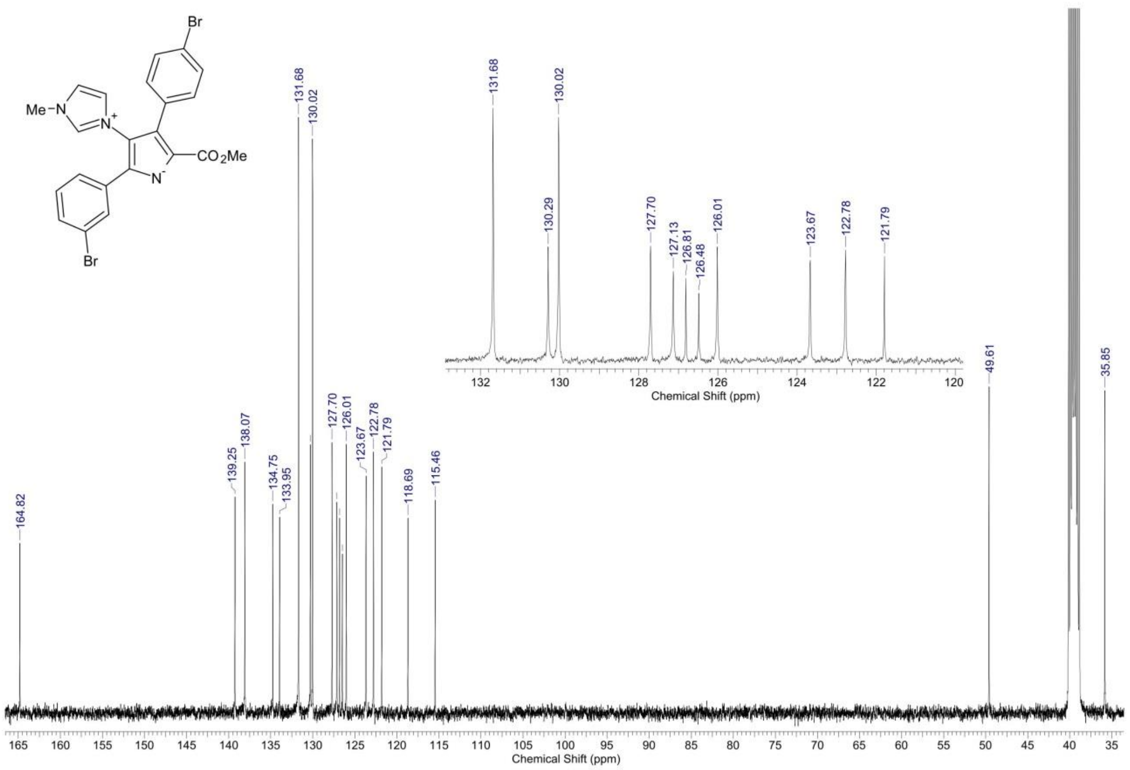

2-Methoxycarbonyl-3,5-diphenyl-4-(1-phenyl-1*H*-imidazol-3-ium-3-yl)pyrrol-1-ide (2e), DMSO-d_6

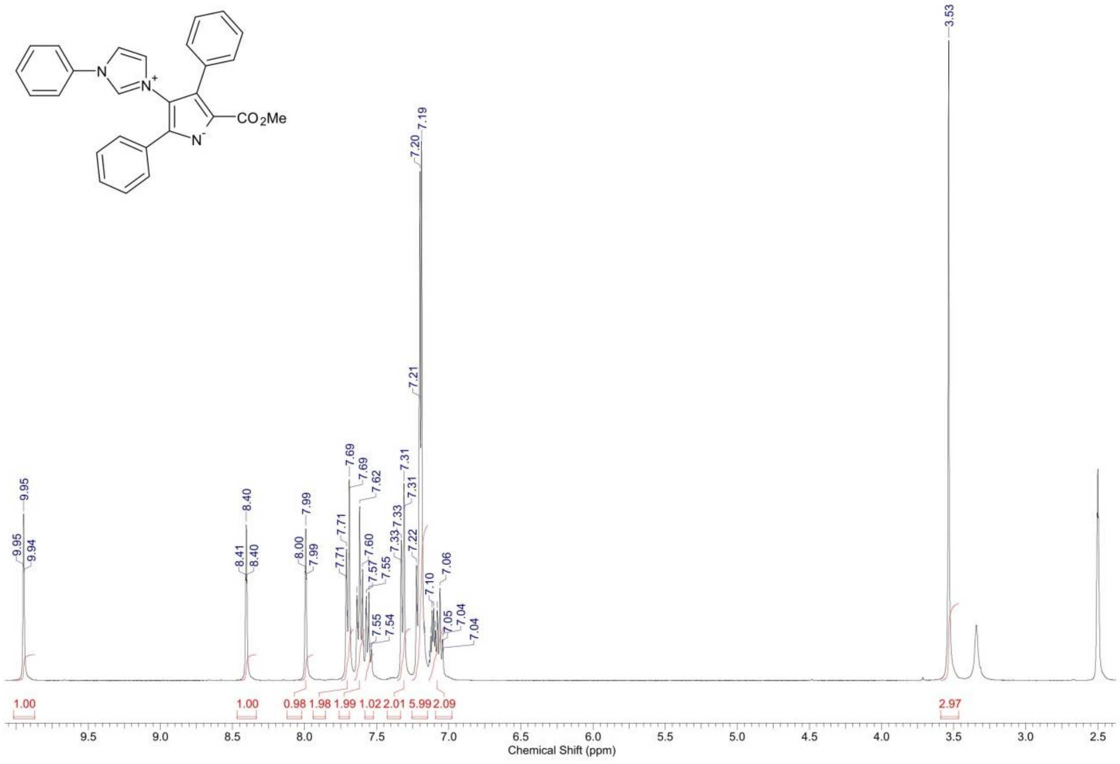

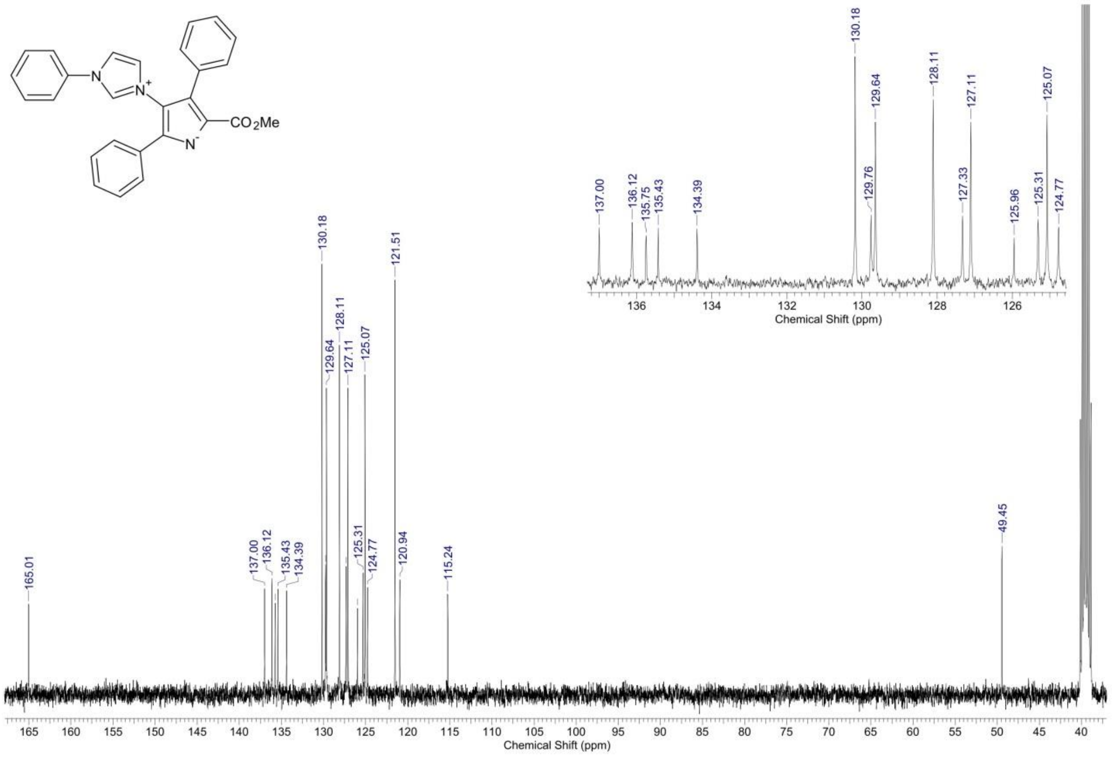

2-(4-Bromophenyl)-5-methoxycarbonyl-4-phenyl-3-(1-phenyl-1H-imidazol-3-ium-3-yl)pyrrol-1-ide (2f), DMSO-d_6

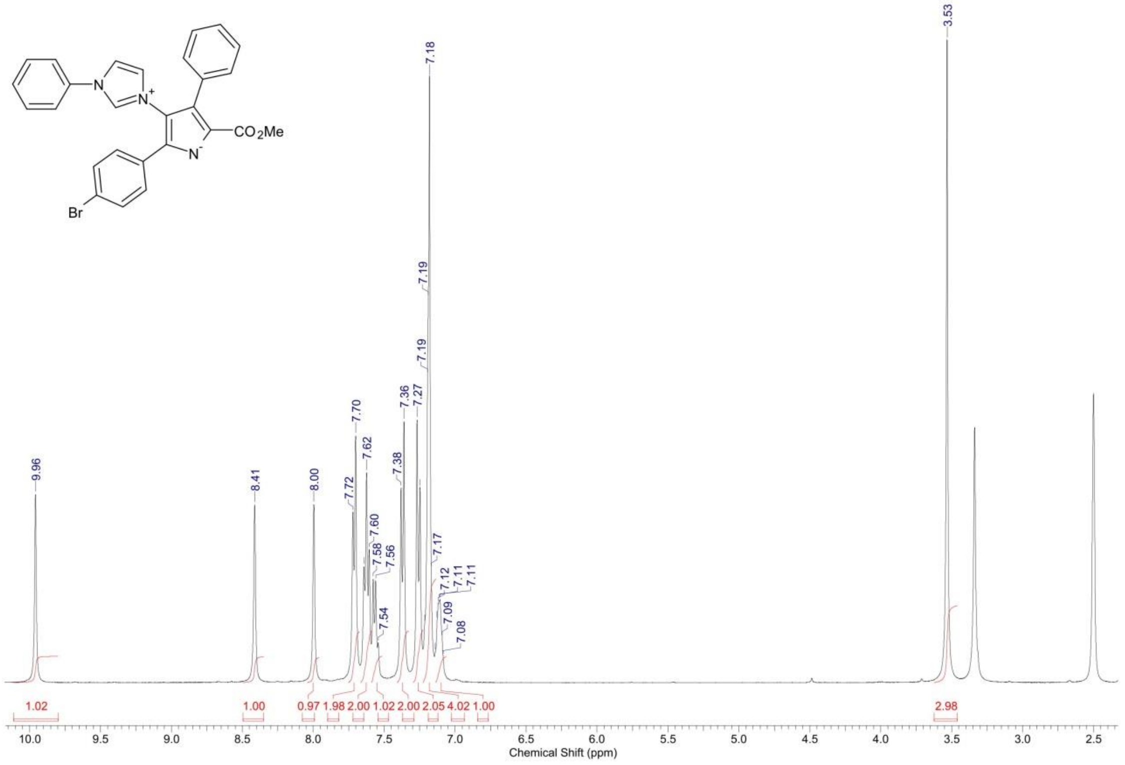

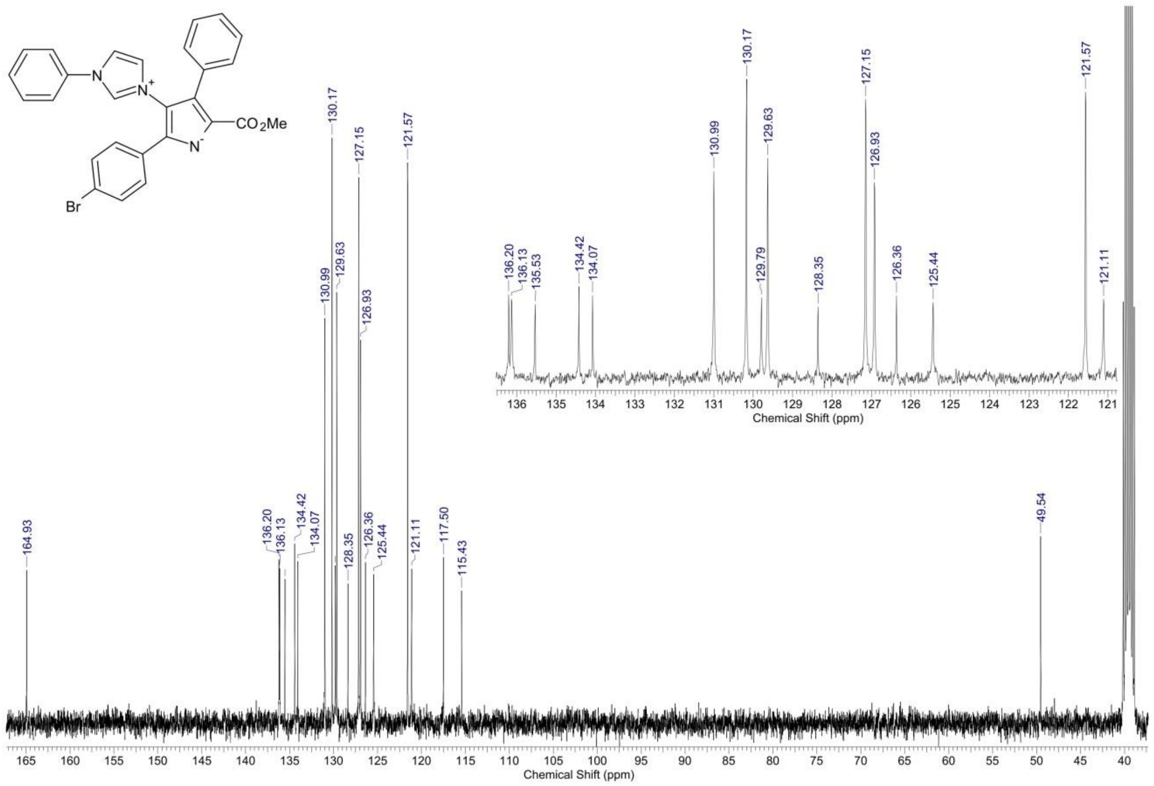

2-Methoxycarbonyl-5-(4-methoxyphenyl)-3-methyl-4-(1-phenyl-1H-imidazol-3-ium-3-yl)pyrrol-1-ide (2g), DMSO-d_6

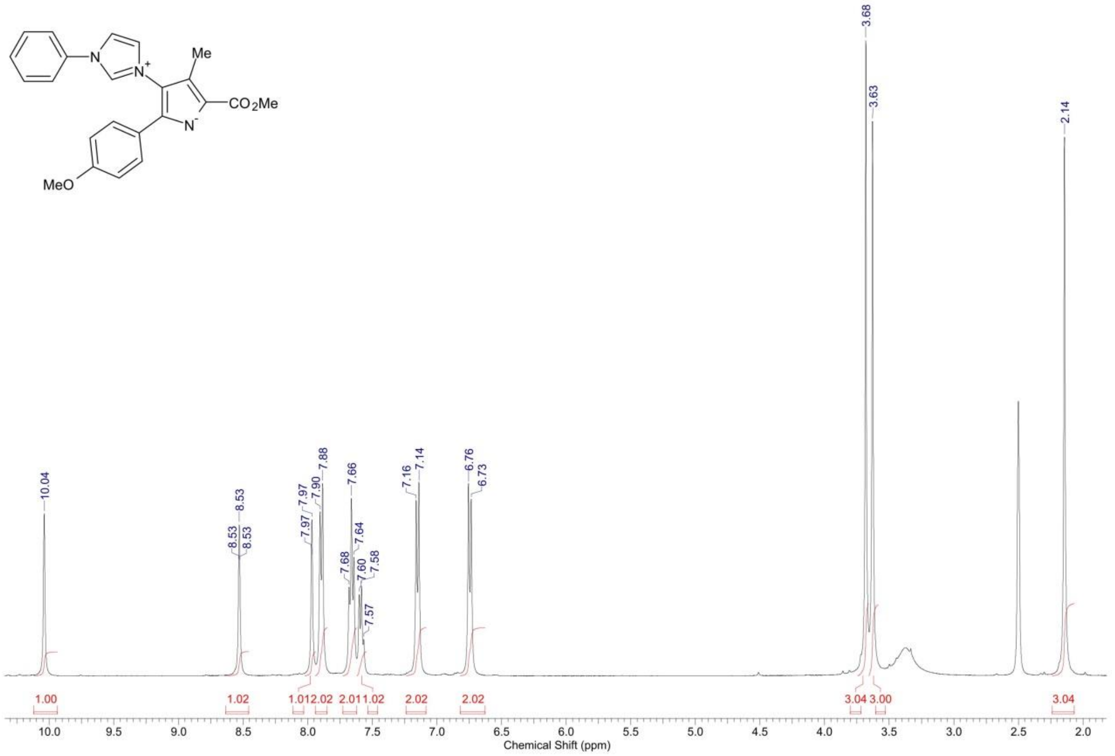

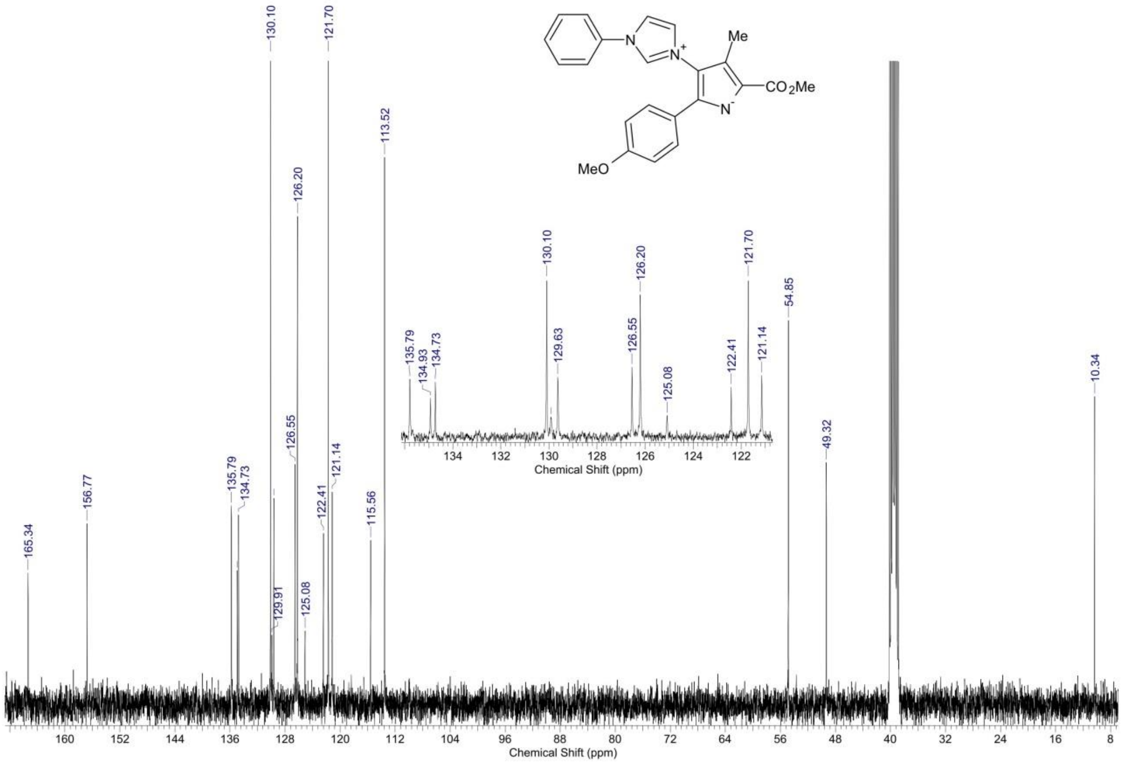

3-(1-Benzyl-1*H*-imidazol-3-ium-3-yl)-2-(4-fluorophenyl)-5-methoxycarbonyl-4-phenylpyrrol-1-ide (2h), DMSO-d_6

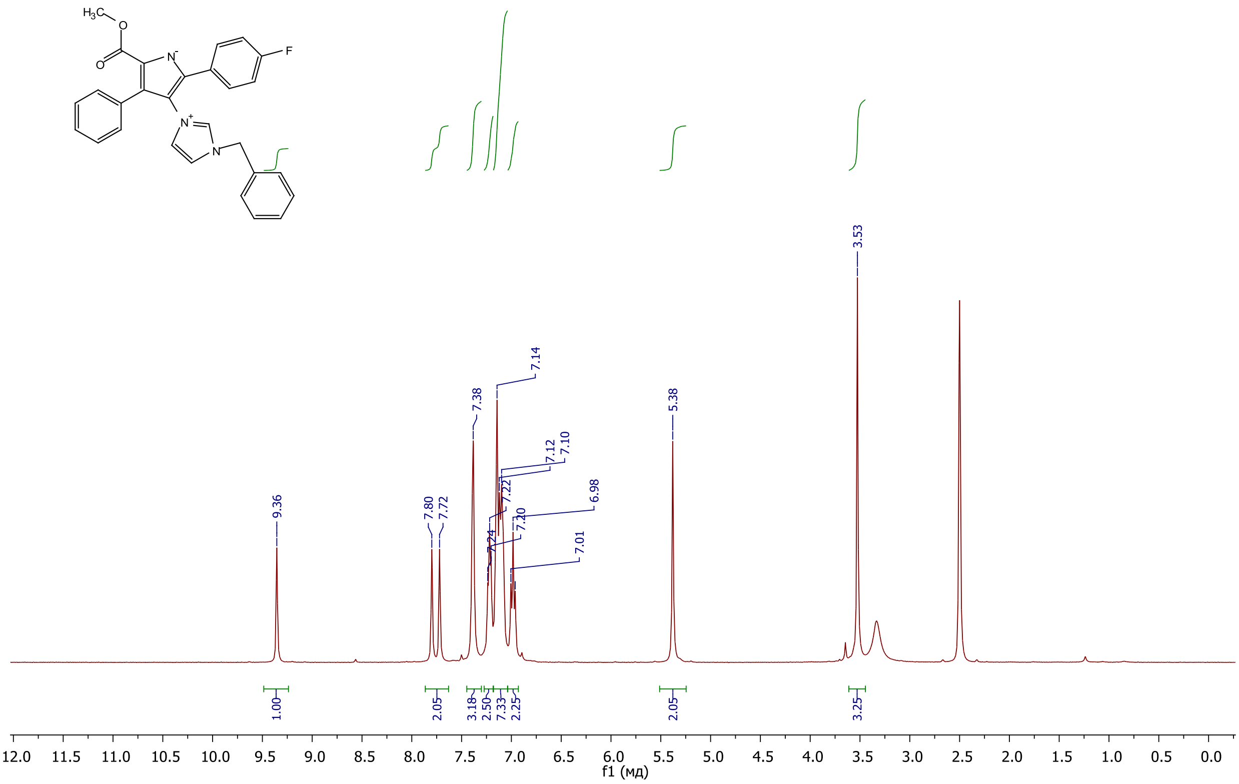

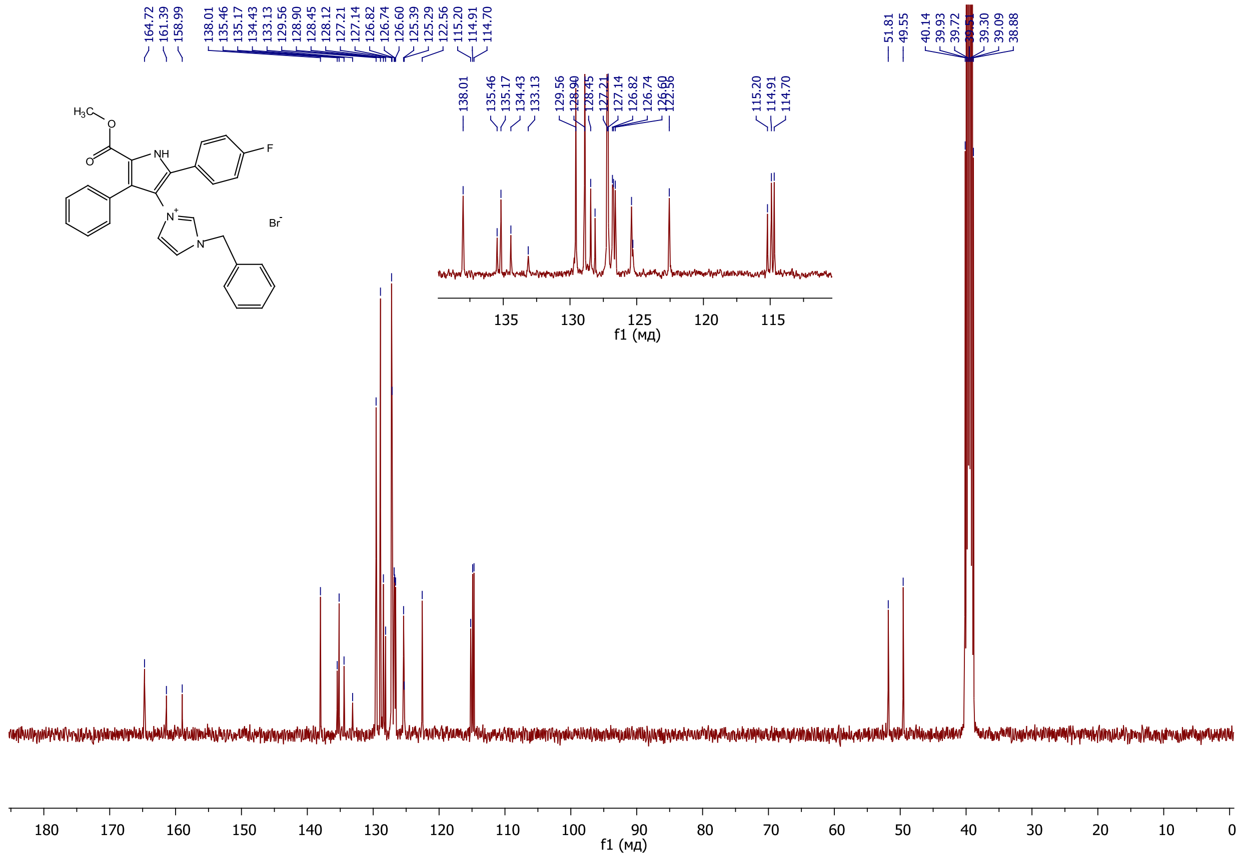

3-(1-Benzyl-1*H*-imidazol-3-ium-3-yl)-2-(4-chlorophenyl)-5-methoxycarbonyl-4-phenylpyrrol-1-ide (2i), DMSO-d_6

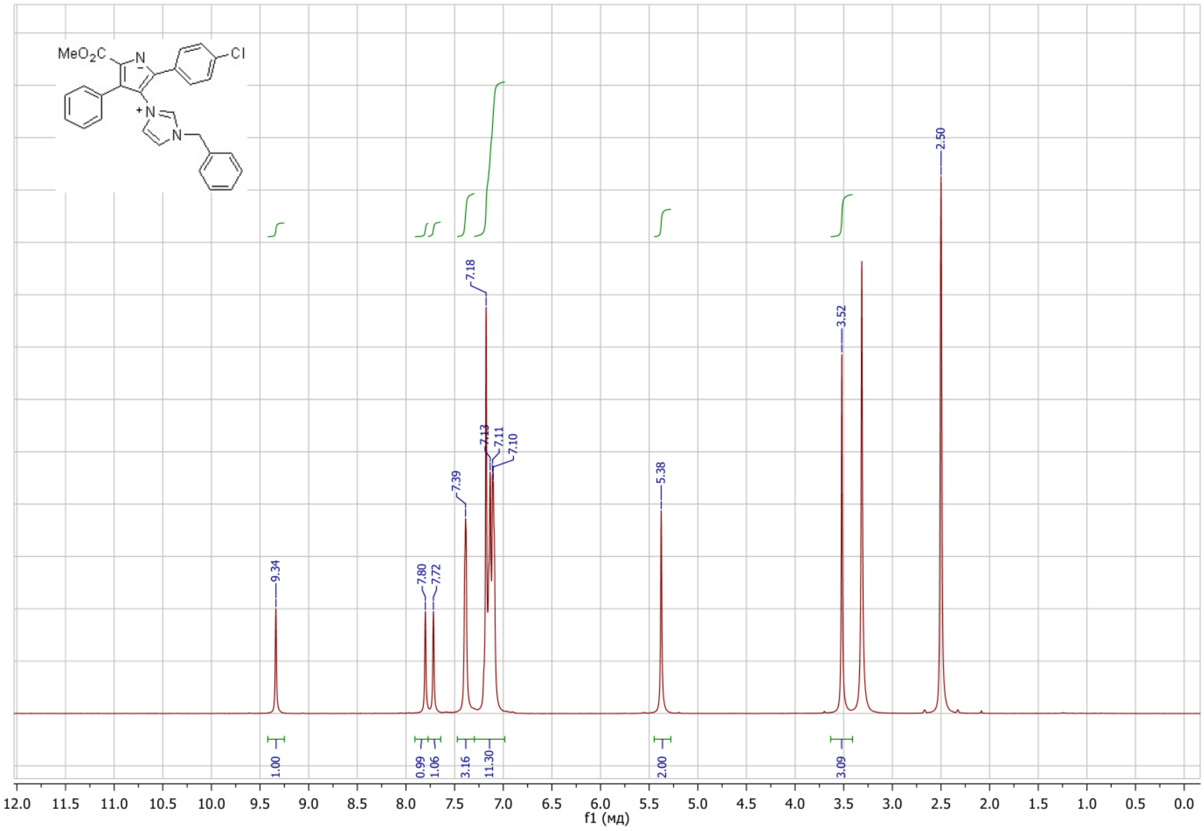

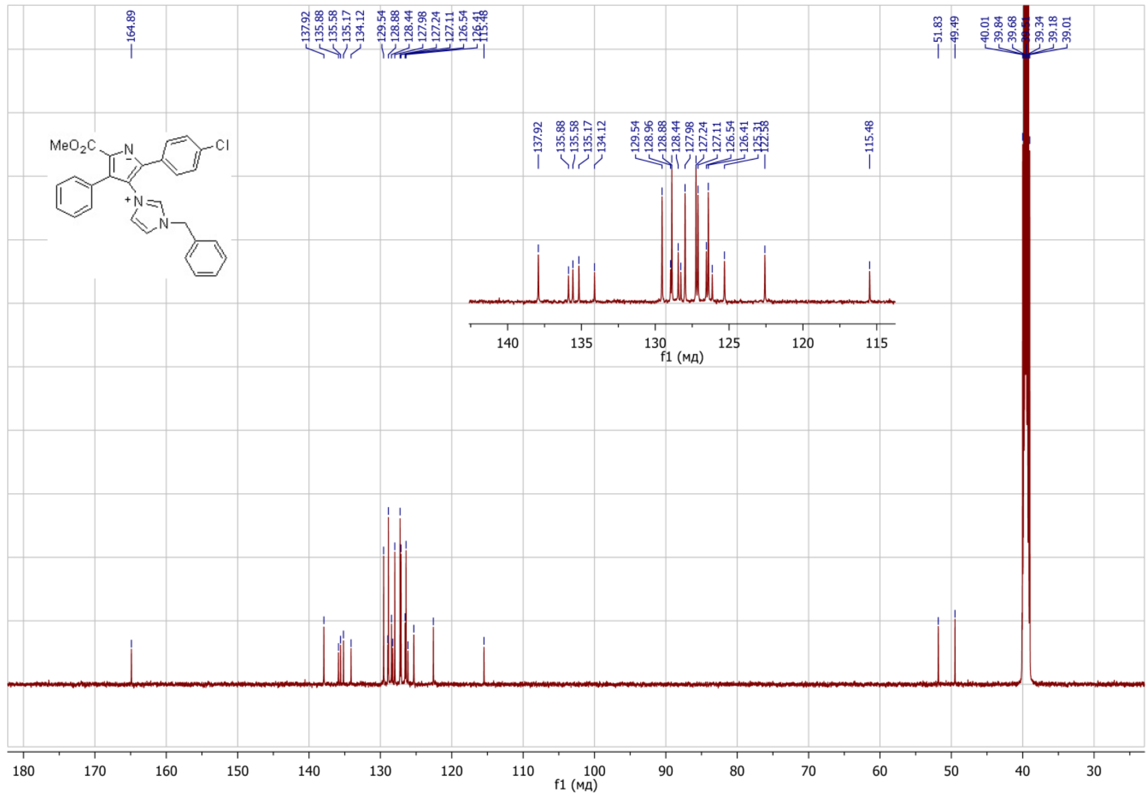

Methyl 4-(3-methyl-2-thioxo-2,3-dihydro-1*H*-imidazol-1-yl)-3,5-diphenyl-1*H*-pyrrole-2-carboxylate (13a), DMSO-d_6

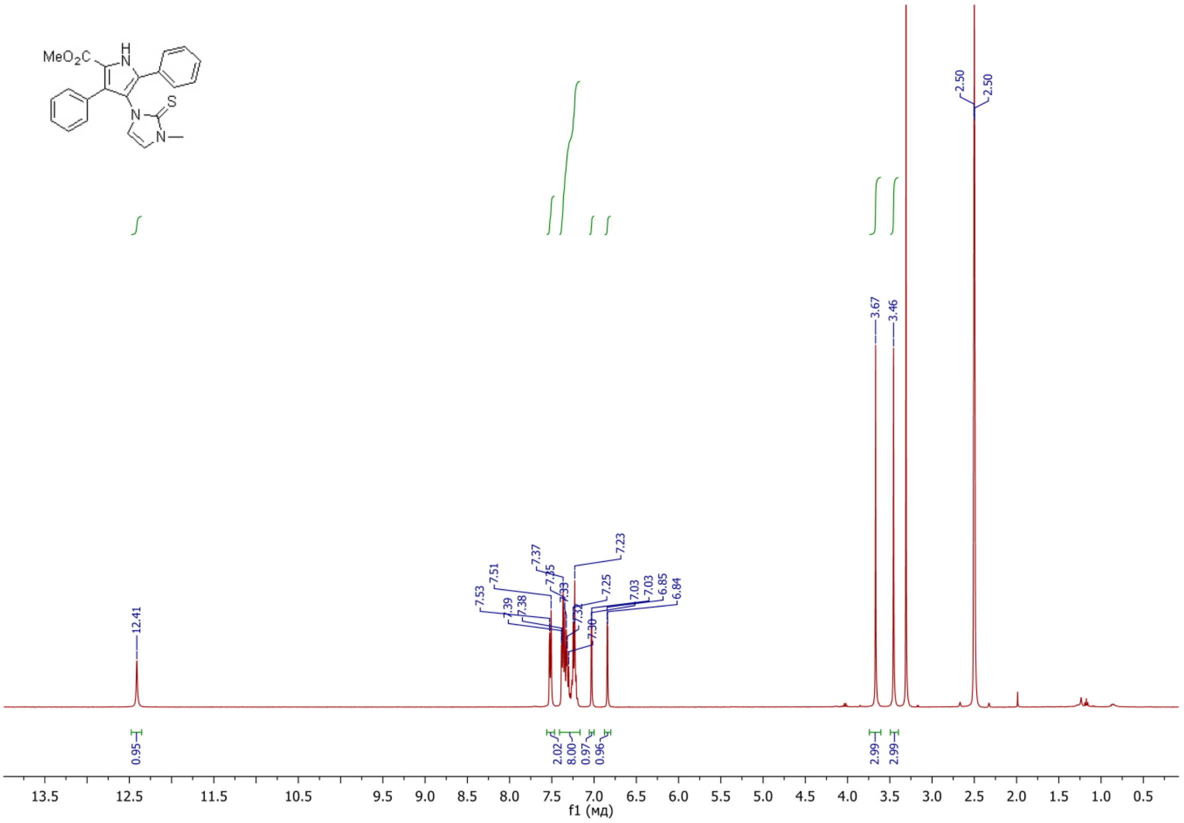

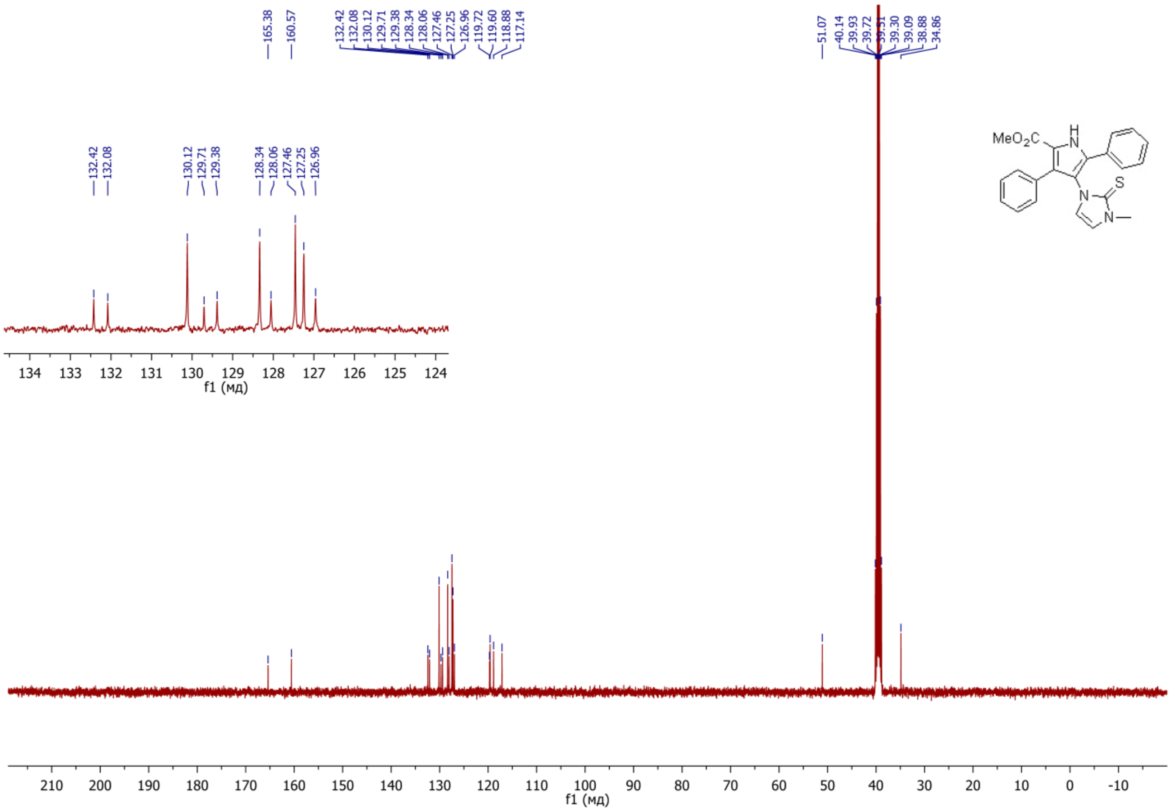

Methyl 5-(4-chlorophenyl)-4-(3-methyl-2-thioxo-2,3-dihydro-1*H*-imidazol-1-yl)-3-phenyl-1*H*-pyrrole-2-carboxylate (13b), DMSO-d_6

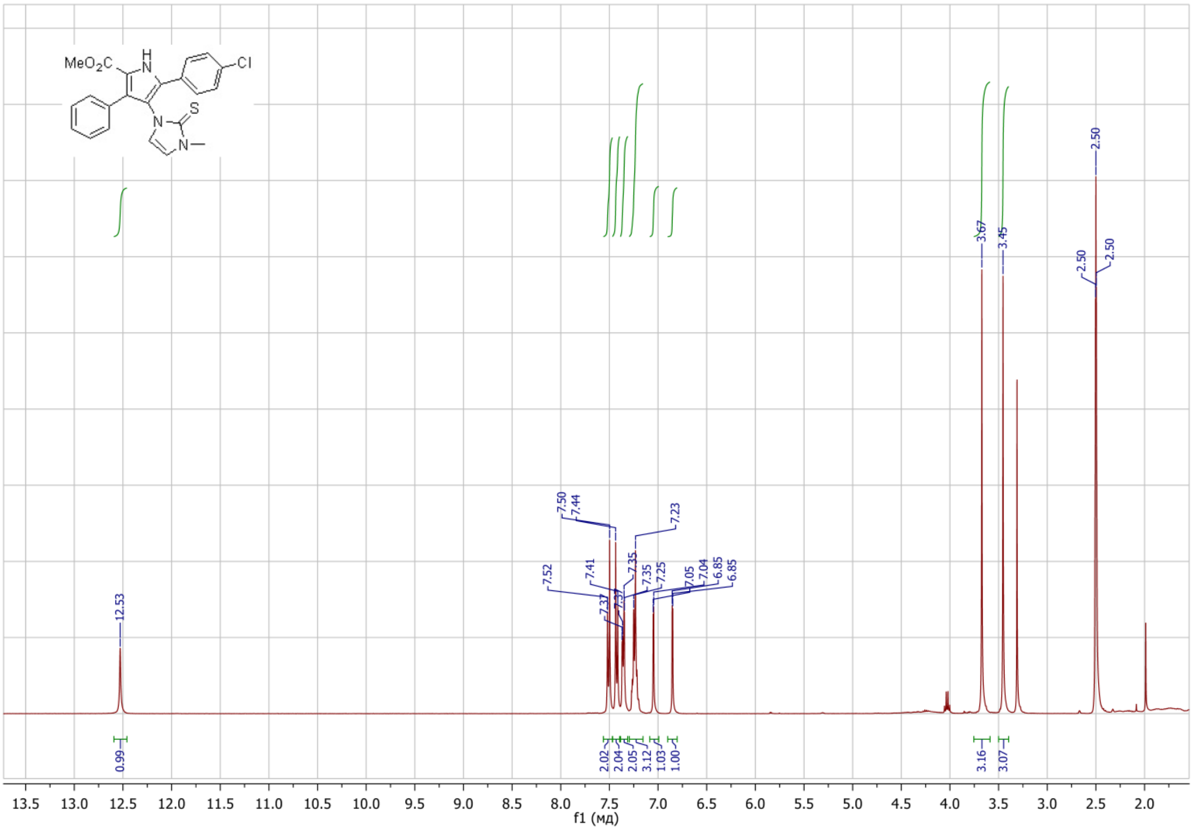

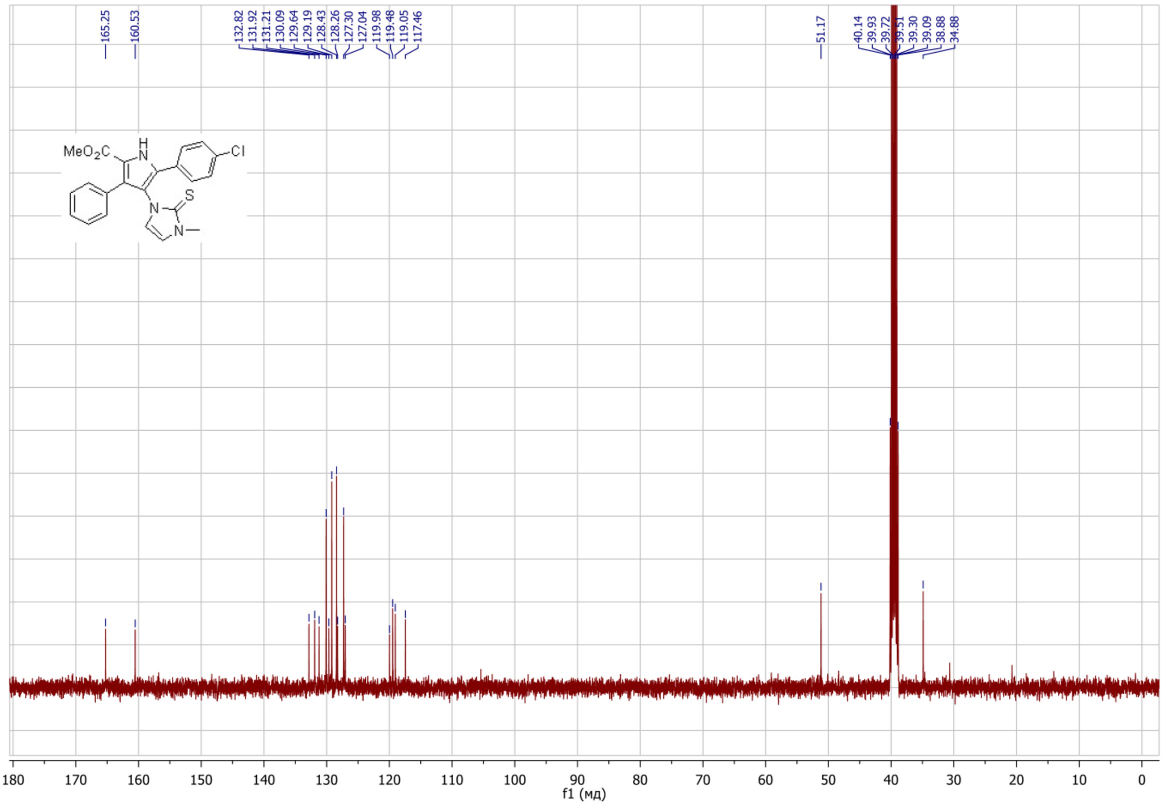

Methyl 4-(3-methyl-2-thioxo-2,3-dihydro-1*H*-imidazol-1-yl)-5-(4-nitrophenyl)-3-phenyl-1*H*-pyrrole-2-carboxylate (13c), DMSO-d_6

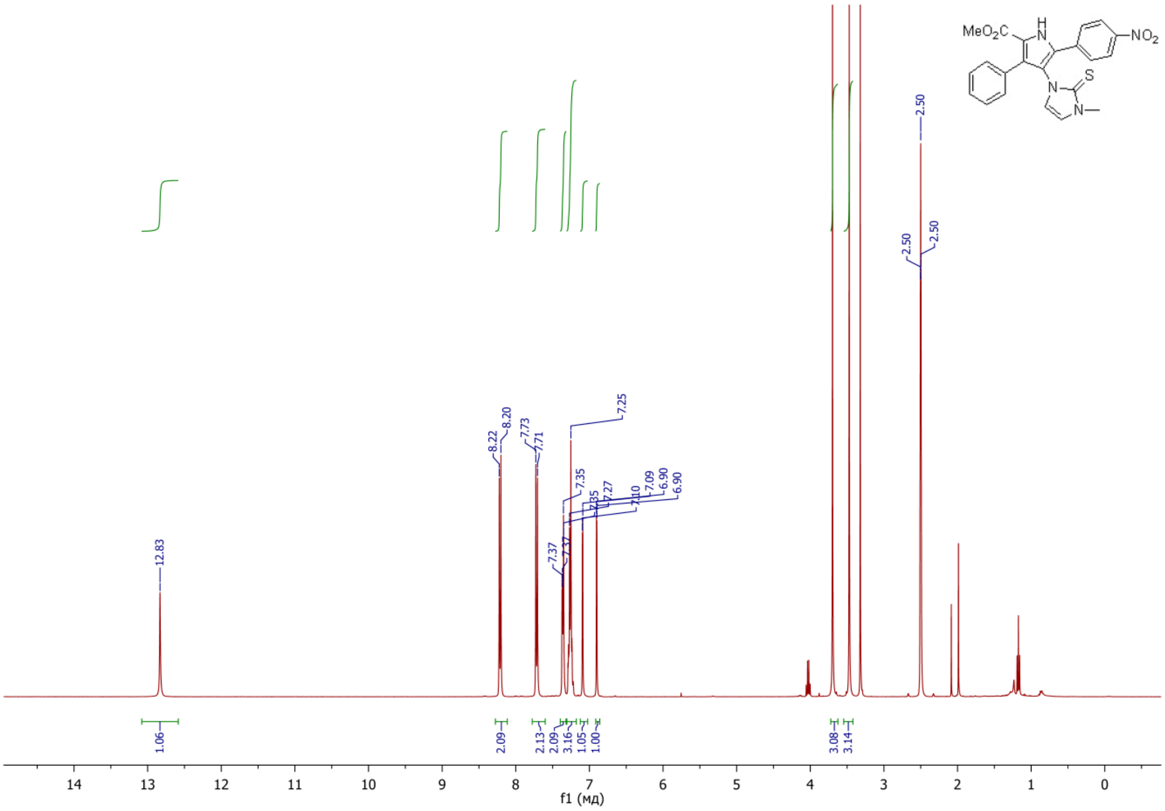

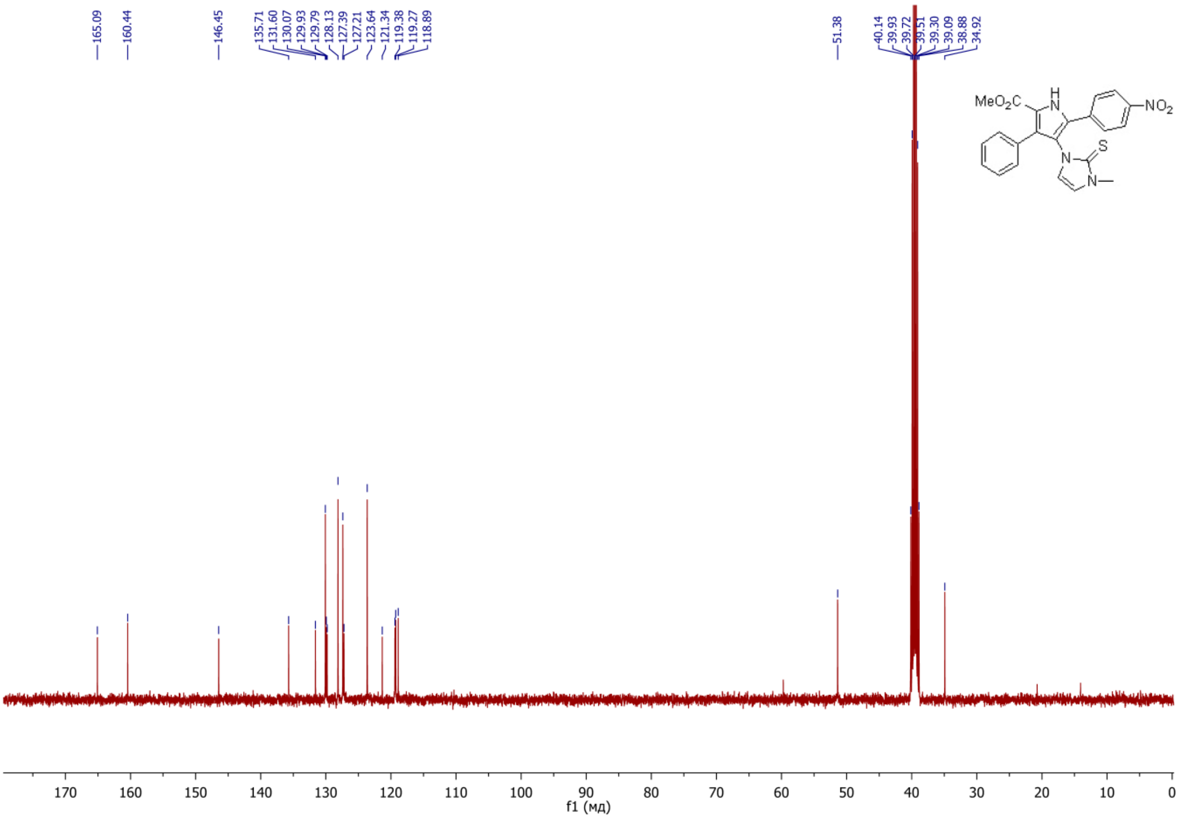

Methyl 5-(3-bromophenyl)-3-(4-bromophenyl)-4-(3-methyl-2-thioxo-2,3-dihydro-1*H*-imidazol-1-yl)-1*H*-pyrrole-2-carboxylate (13d), DMSO-d_6

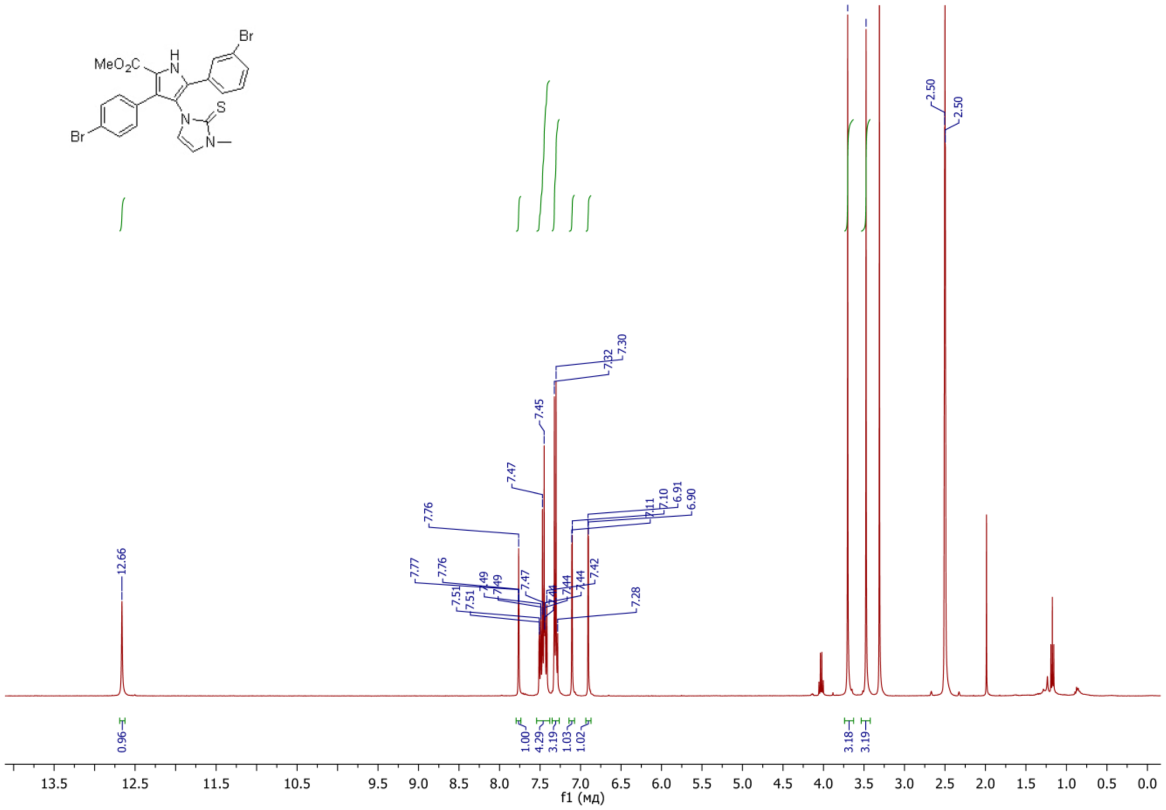

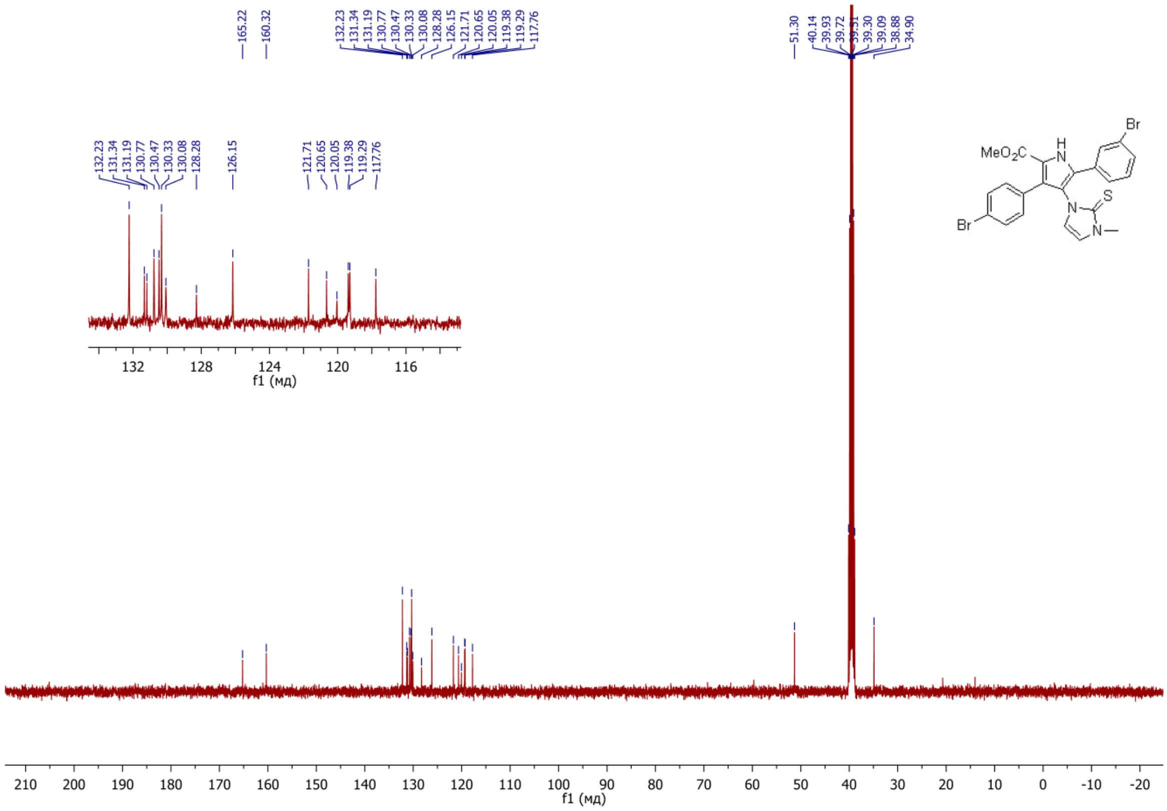

Methyl 3,5-diphenyl-4-(3-phenyl-2-thioxo-2,3-dihydro-1*H*-imidazol-1-yl)-1*H*-pyrrole-2-carboxylate (13e), DMSO-d_6

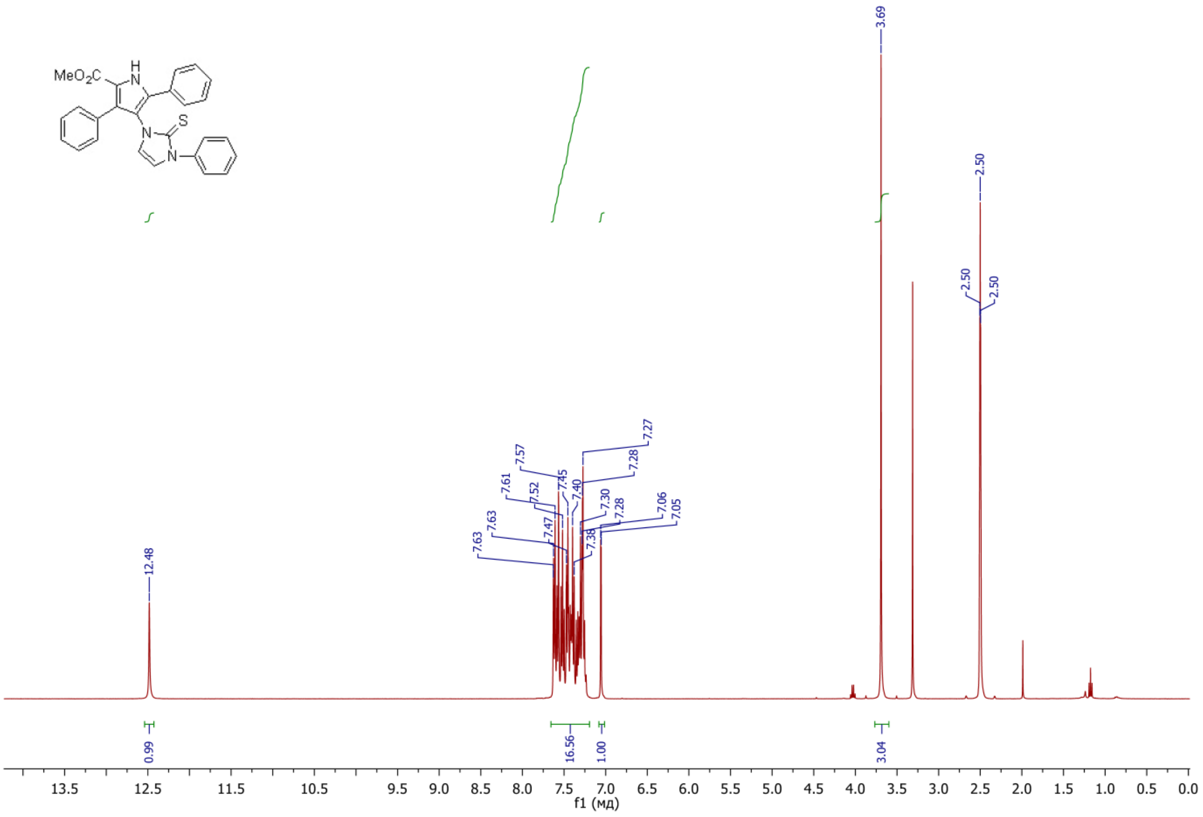

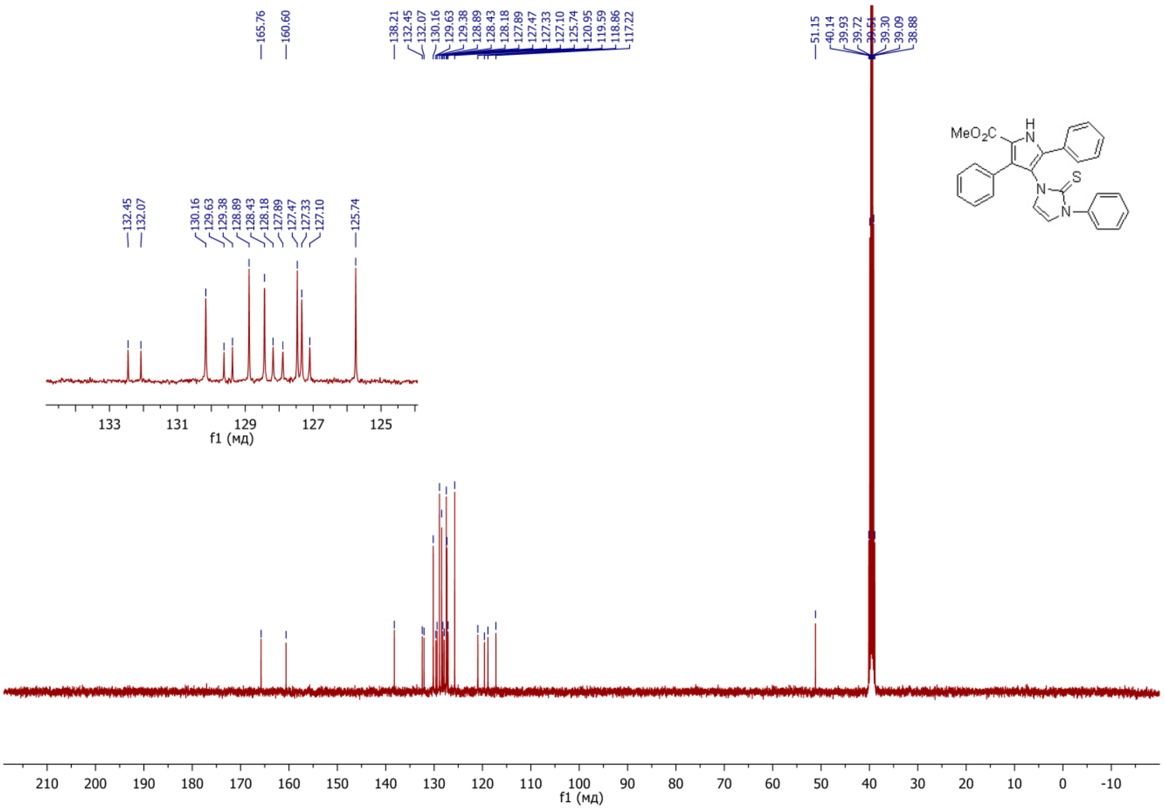

Methyl 5-(4-bromophenyl)-3-phenyl-4-(3-phenyl-2-thioxo-2,3-dihydro-1*H*-imidazol-1-yl)-1*H*-pyrrole-2-carboxylate (13f),^{1}H NMR (CDCl$_3$), ^{13}C NMR (DMSO-d_6)

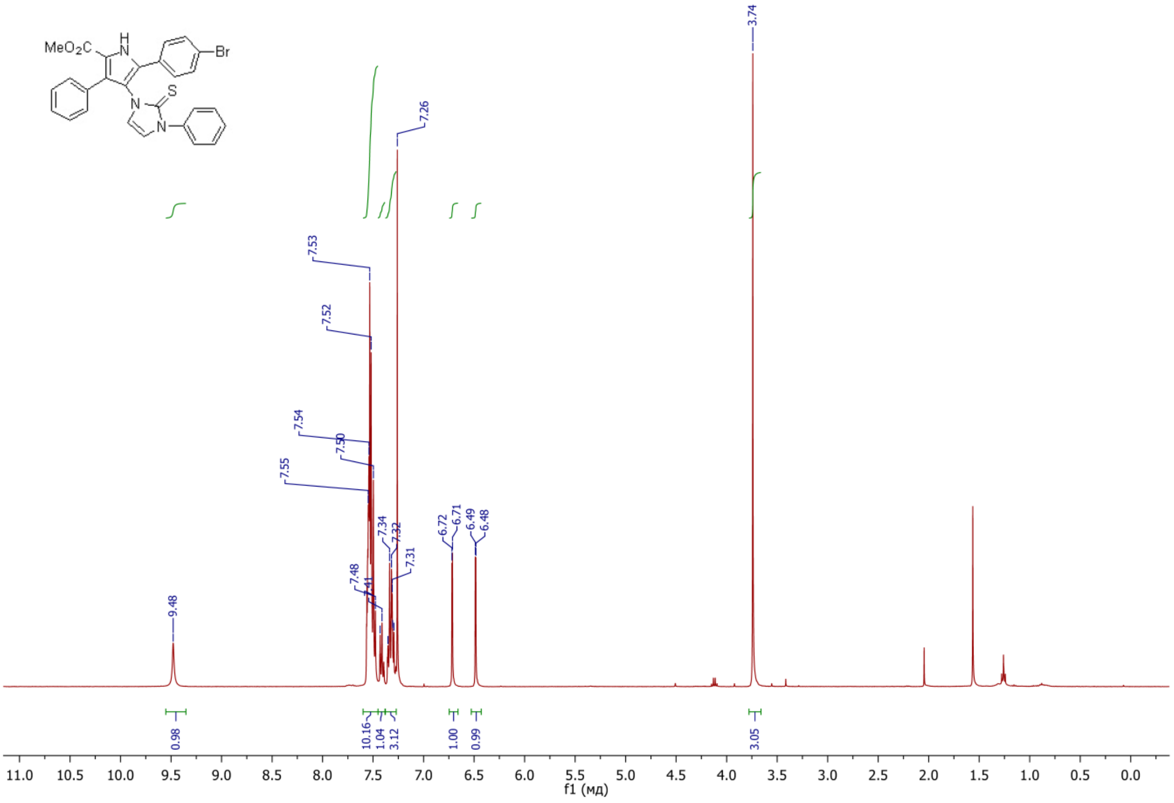

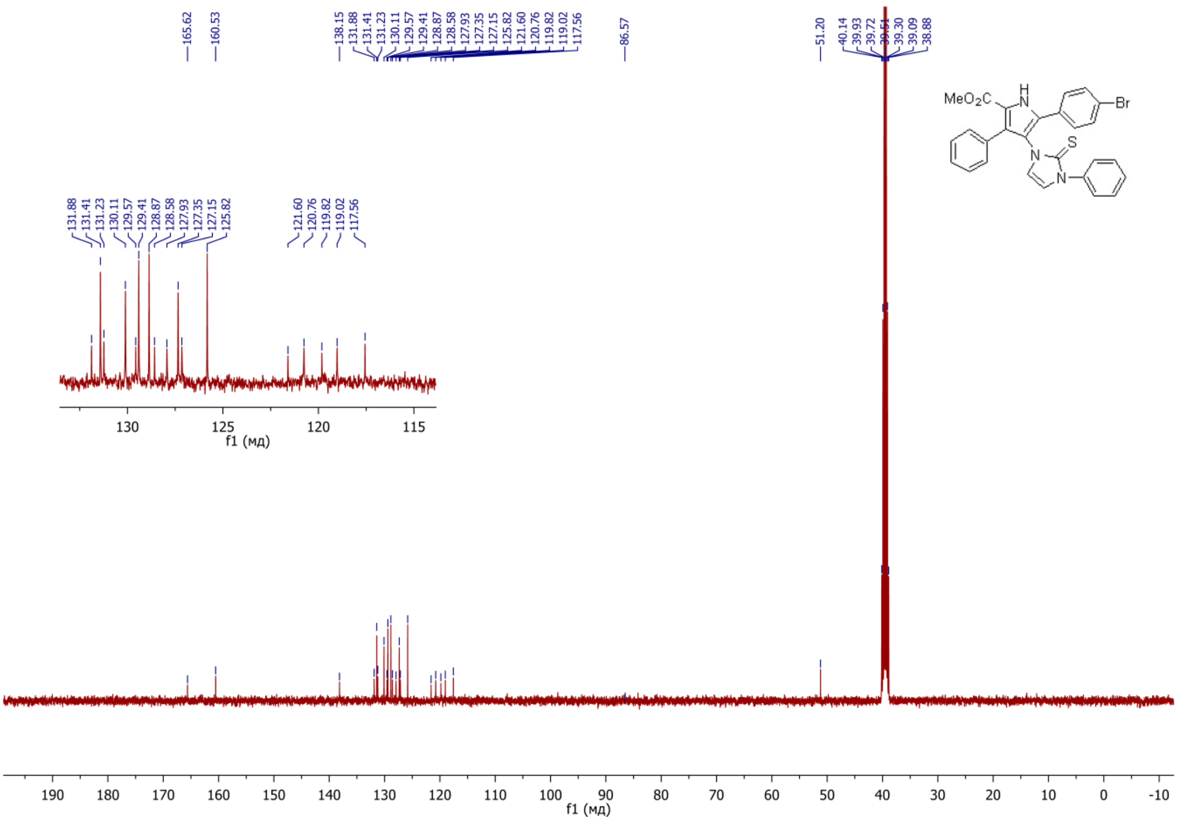

Methyl 5-(4-methoxyphenyl)-3-methyl-4-(3-phenyl-2-thioxo-2,3-dihydro-1*H*-imidazol-1-yl)-1*H*-pyrrole-2-carboxylate (13g), DMSO-d_6

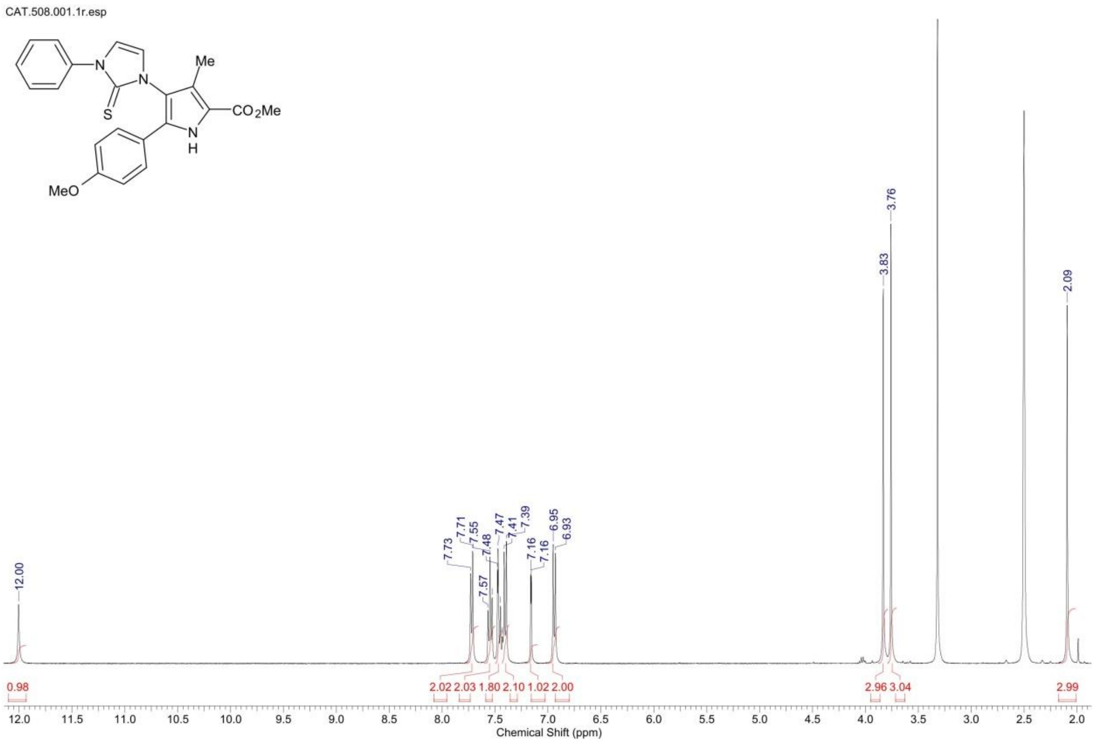

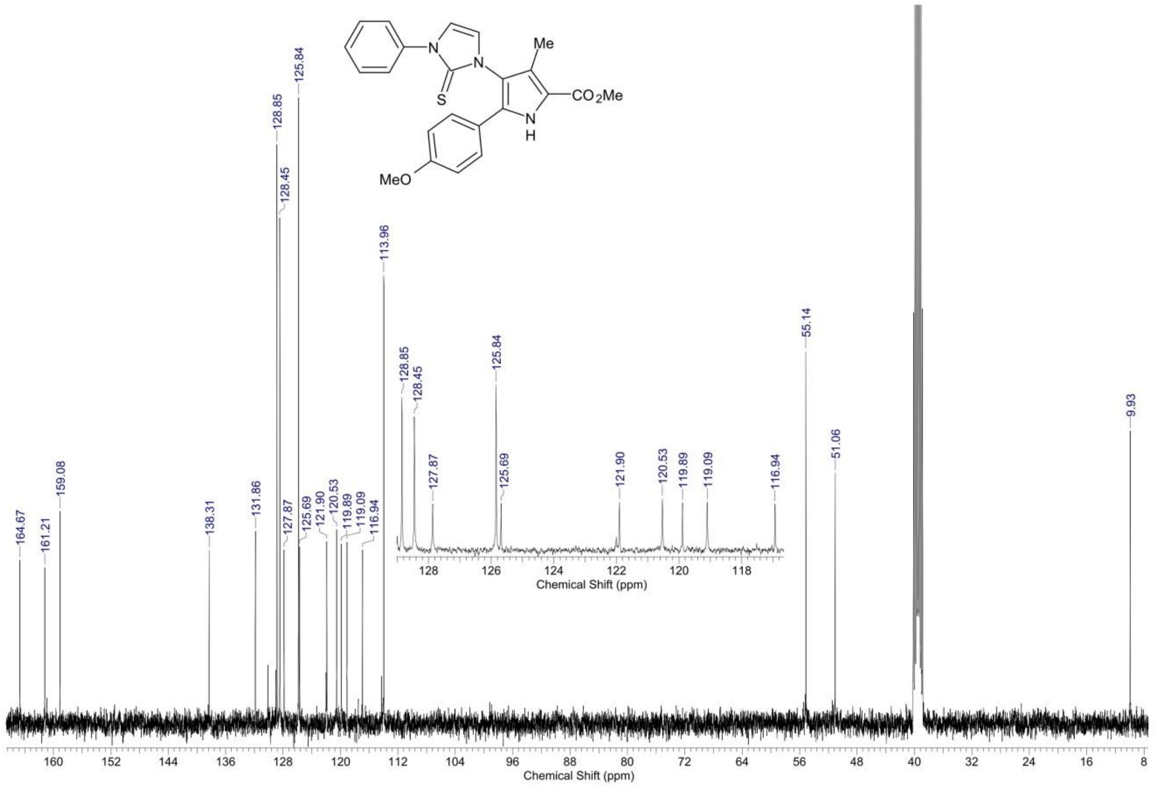

Methyl 4-(3-benzyl-2-thioxo-2,3-dihydro-1*H*-imidazol-1-yl)-5-(4-chlorophenyl)-3-phenyl-1*H*-pyrrole-2-carboxylate (13h), DMSO-d_6

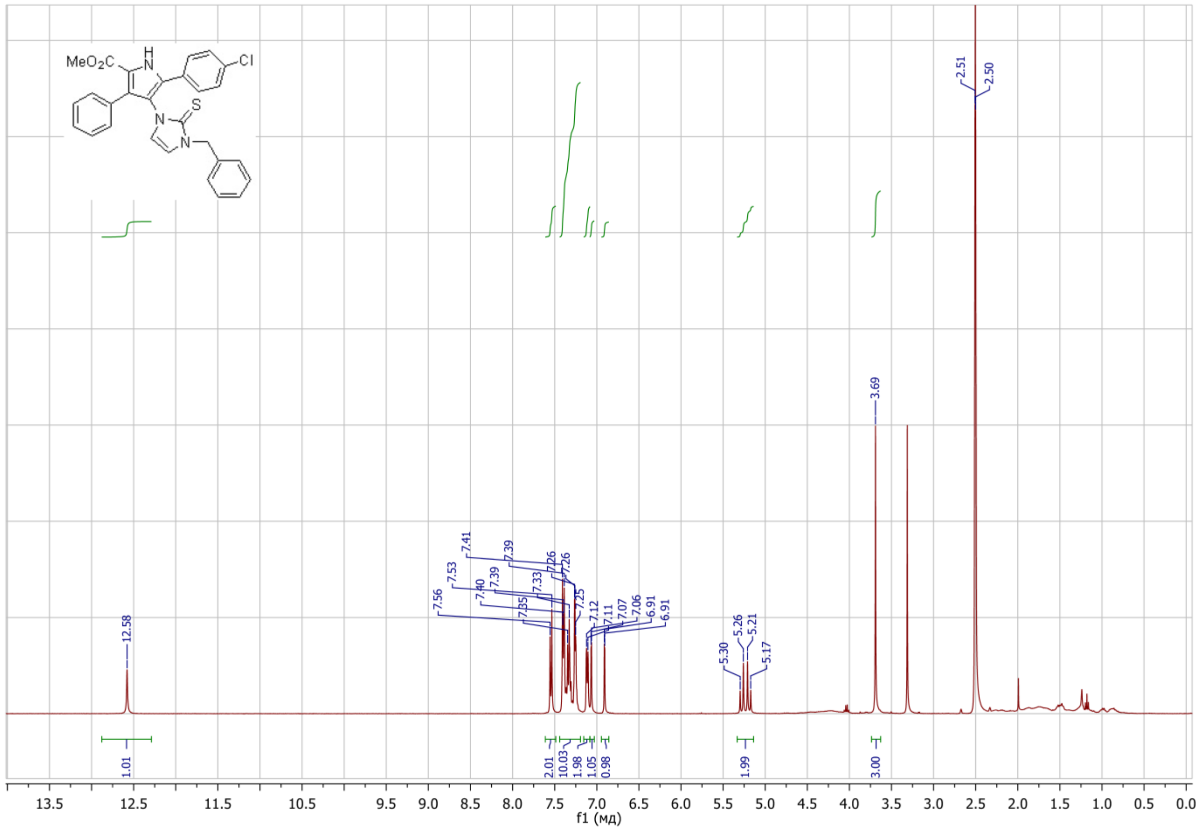

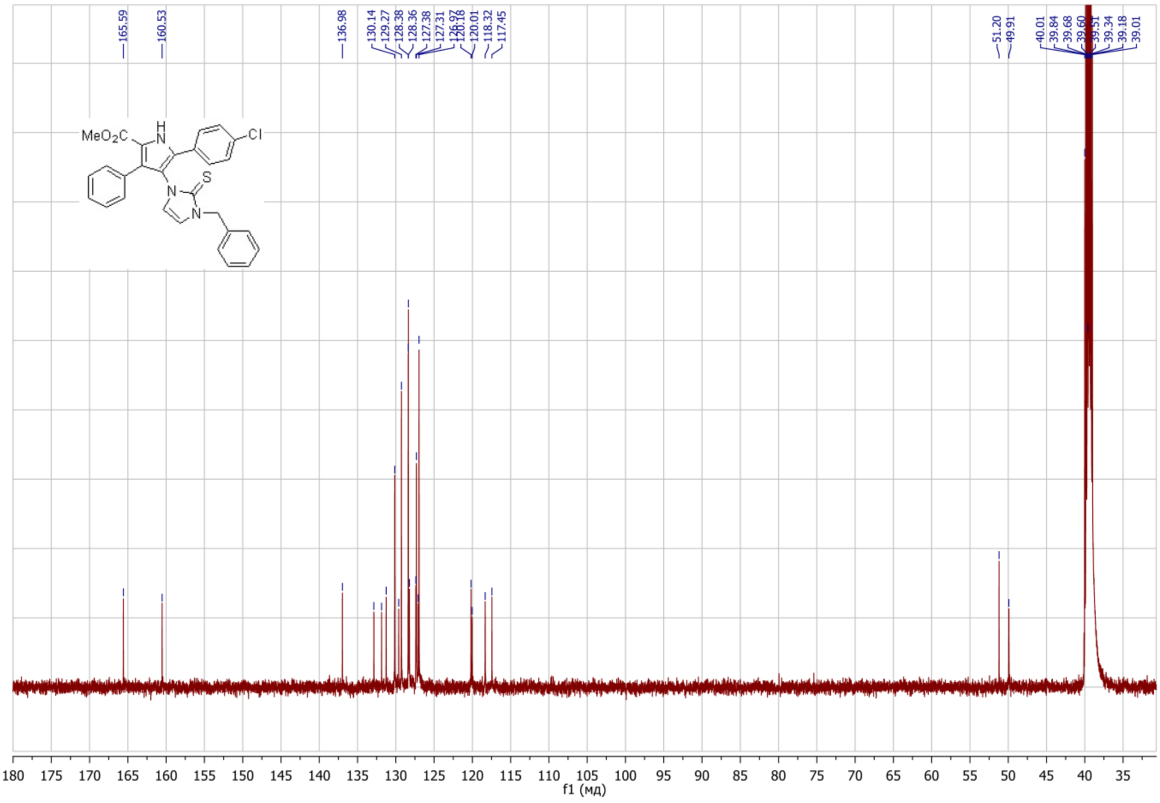

4-(1-Methyl-1*H*-imidazol-3-ium-3-yl)-3,5-diphenyl-1*H*-pyrrole-2-carboxylate (6a), MeOH-*d*$_4$

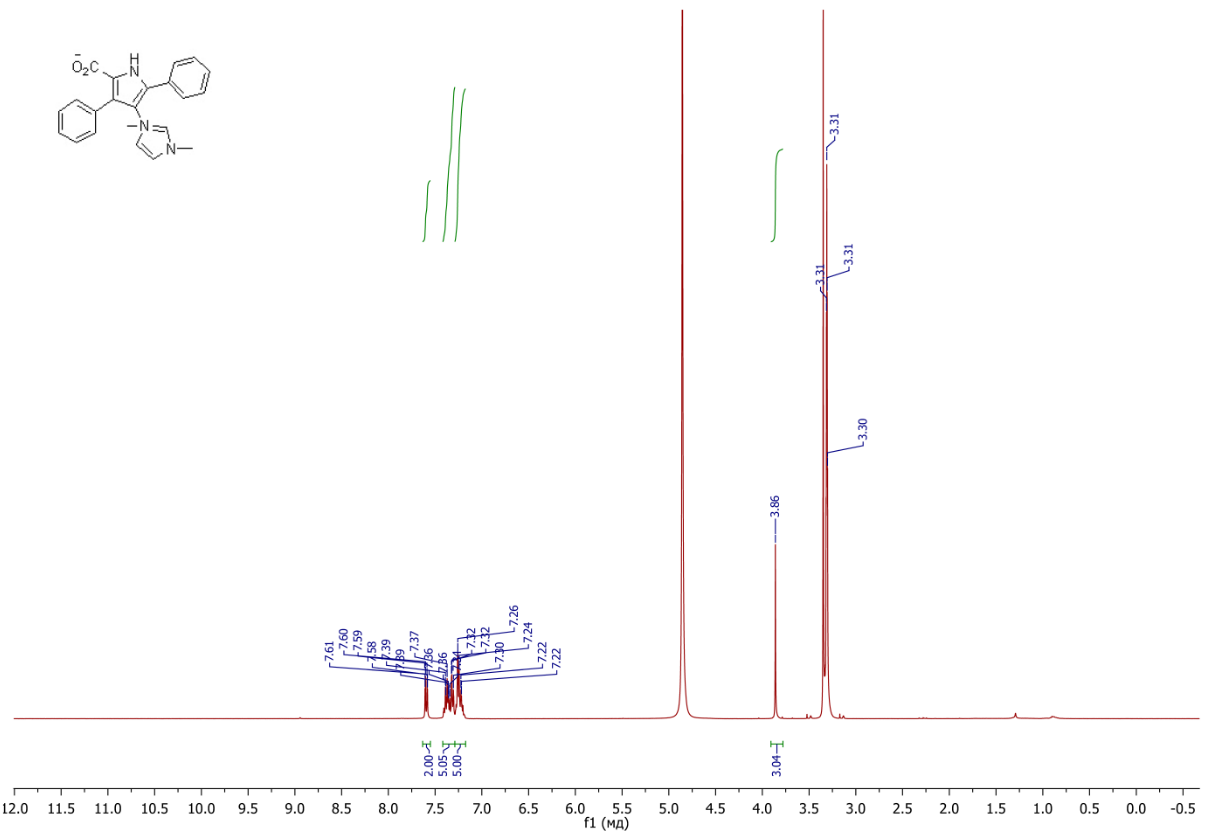

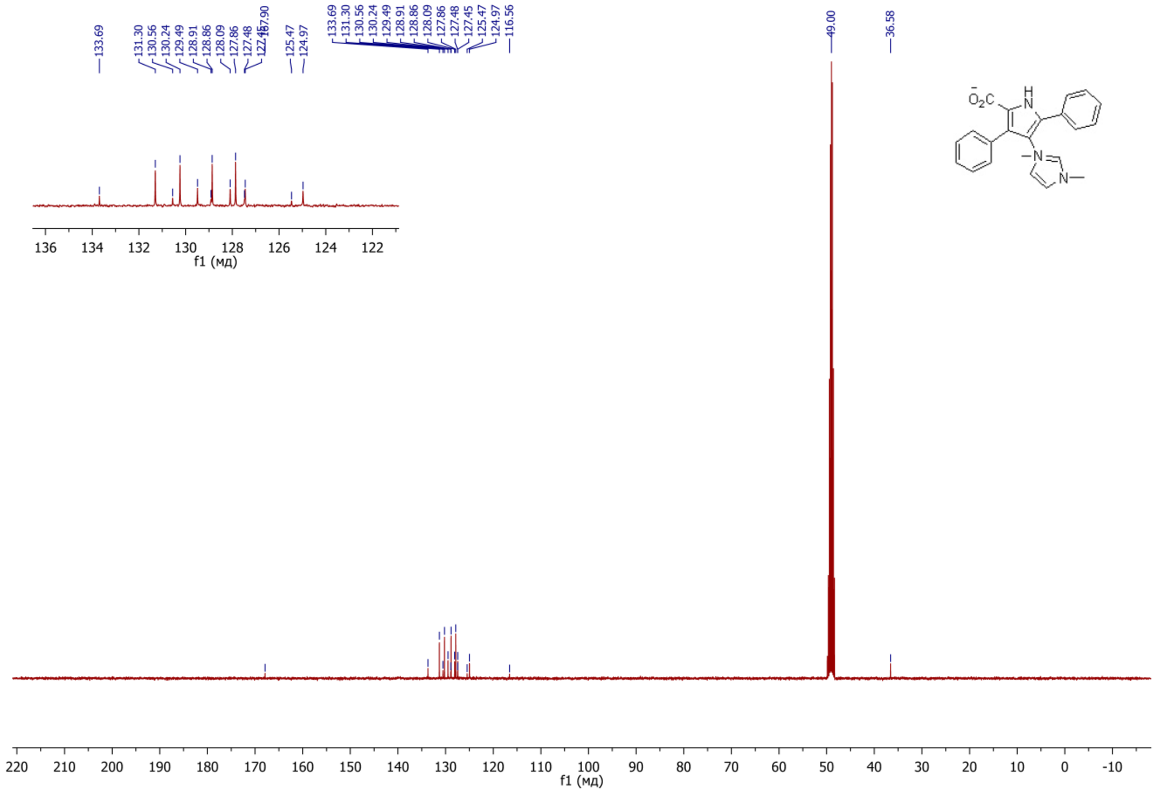

5-(4-Chlorophenyl)-4-(1-methyl-1*H*-imidazol-3-ium-3-yl)-3-phenyl-1*H*-pyrrole-2-carboxylate (6b), DMSO-d_6

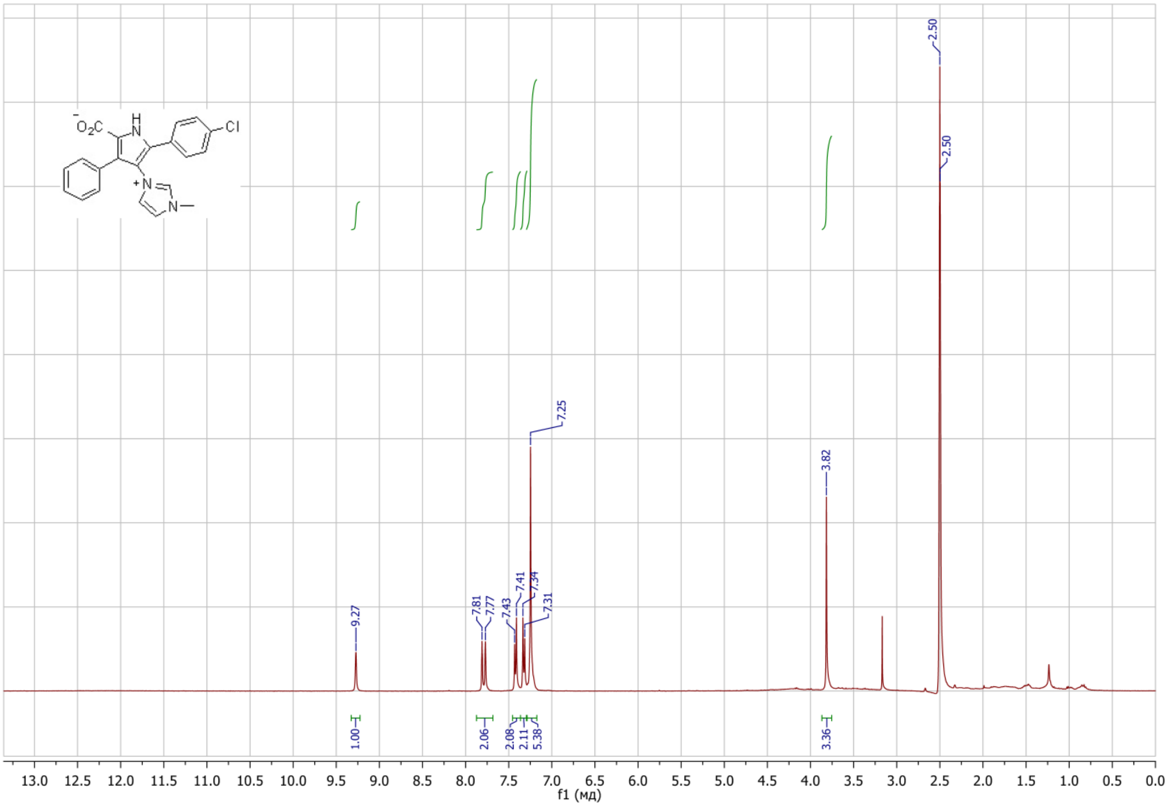

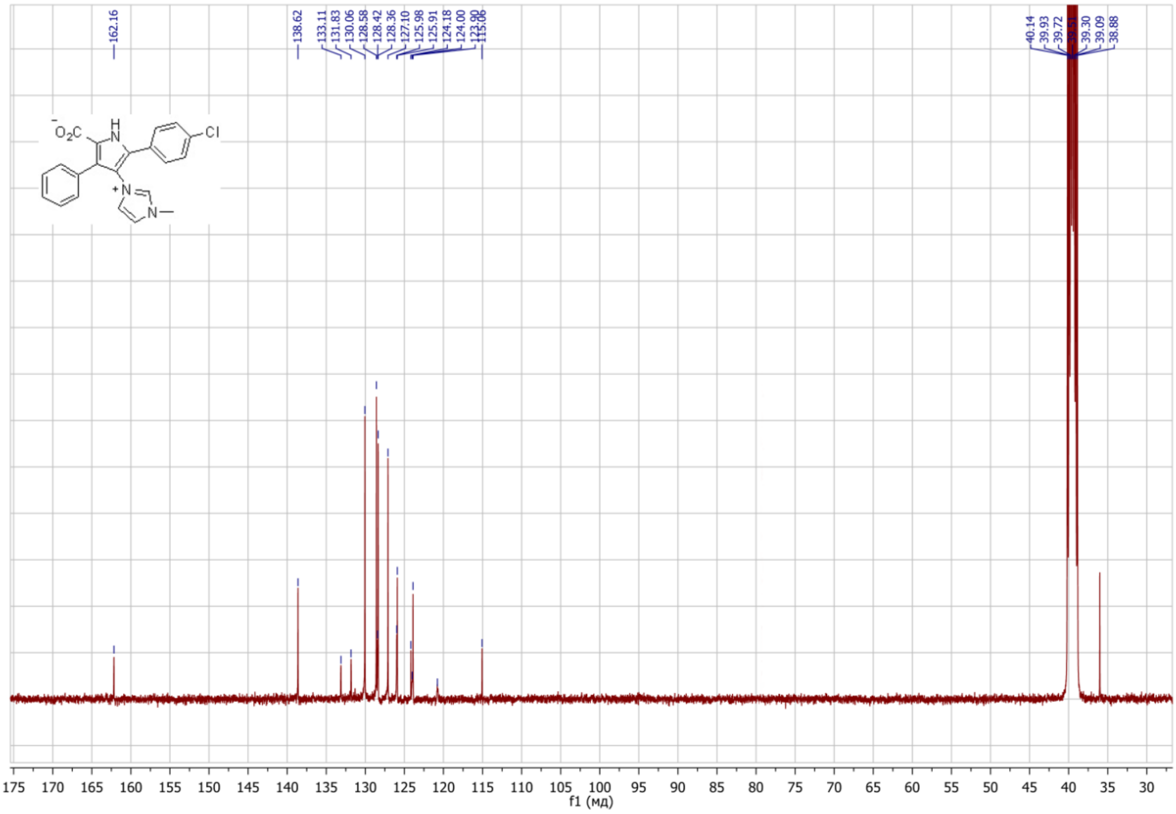

Computational details

All calculations were performed with the B3LYP density functional method[1] by using the Gaussian 09 suite of quantum chemical programs[2] at Resource center "Computer center of Saint Petersburg State University". Geometry optimizations of molecules were performed at the B3LYP/6-31G+(d,p) level in gas phase or with PCM solvent model.

Table. B3LYP/6-31G+(d,p) Absolute Energies (au), Cartesian Coordinates of stationary points

Molecule **2a** (gas phase)				Molecule **2a** (PCM for CH_2Cl_2)			
**E** = -1164.51967896, **H (0K)** = -1164.154362, **H (298K)** = -1164.129805, **G (298K)** = -1164.210466 au.				**E** = -1164.55403995, **H (0K)** = -1164.188399, **H (298K)** = -1164.163958, **G (298K)** = -1164.243983 au			
Imaginary frequency = 0.				Imaginary frequency = 0.			
C	1.3153540	3.5149020	0.6809720	C	1.3756010	3.4561110	0.8470750
C	0.4939550	2.4687230	0.9867620	C	0.6828800	2.3349100	1.1981960
N	0.7974160	1.4327260	0.1163400	N	0.8001080	1.4335930	0.1506410
C	1.7717580	1.8495800	-0.7009430	C	1.5426260	2.0033630	-0.8099040
C	0.2217970	0.1264710	0.1059440	C	0.2476640	0.1127200	0.0955910
C	0.9337360	-1.1049330	0.0822250	C	0.9724090	-1.1101310	0.0786400
N	0.0549410	-2.1169890	0.0076730	N	0.1051700	-2.1366890	-0.0051350
C	-1.1964560	-1.5717060	0.0110490	C	-1.1563470	-1.6019410	-0.0234590
C	-1.1613930	-0.1535600	0.0659100	C	-1.1271560	-0.1817540	0.0347600
C	2.3849020	-1.3522350	0.1495750	C	2.4296410	-1.3344370	0.1327980
C	2.9330670	-2.4555950	-0.5339640	C	2.9909050	-2.4549650	-0.5126010
C	4.3012640	-2.7208350	-0.4873040	C	4.3637930	-2.6997450	-0.4663030
C	5.1626440	-1.8913880	0.2404580	C	5.2167930	-1.8286670	0.2220160
C	4.6320100	-0.8033680	0.9390060	C	4.6748280	-0.7162150	0.8726890
C	3.2599200	-0.5404600	0.8989320	C	3.2989430	-0.4737400	0.8322630
C	-2.2456070	0.8531690	0.0270130	C	-2.2256300	0.8117620	0.0360580
C	-2.2517540	1.8544330	-0.9620970	C	-2.2971560	1.8028200	-0.9596070
C	-3.2384380	2.8439960	-0.9856960	C	-3.3074460	2.7697960	-0.9436720
C	-4.2439670	2.8533520	-0.0160630	C	-4.2674250	2.7657650	0.0720690
C	-4.2562760	1.8592470	0.9684850	C	-4.2082130	1.7856090	1.0697650
C	-3.2708180	0.8719240	0.9899440	C	-3.1981110	0.8224400	1.0522320
H	1.3956830	4.4994100	1.1134820	H	1.5342500	4.3838940	1.3729550
H	-0.2766060	2.3619140	1.7321140	H	0.1267490	2.0932100	2.0888060
H	2.2187760	1.2520080	-1.4784140	H	1.8011040	1.5418870	-1.7494360
H	2.2598070	-3.1008000	-1.0887480	H	2.3330400	-3.1295750	-1.0506780
H	4.6988420	-3.5797810	-1.0214780	H	4.7705670	-3.5706840	-0.9732740
H	6.2280870	-2.1000600	0.2757740	H	6.2857610	-2.0181340	0.2557960
H	5.2833330	-0.1713420	1.5377400	H	5.3214940	-0.0406030	1.4261860
H	2.8598350	0.2753040	1.4956150	H	2.8987190	0.3754580	1.3773440
H	-1.4900220	1.8363520	-1.7376050	H	-1.5661380	1.8034080	-1.7635030
H	-3.2290480	3.5971810	-1.7694580	H	-3.3467790	3.5201020	-1.7287220
H	-5.0165960	3.6169170	-0.0340830	H	-5.0546900	3.5141120	0.0853330
H	-5.0423100	1.8474600	1.7186450	H	-4.9498990	1.7723560	1.8637540
H	-3.2981250	0.0927260	1.7433850	H	-3.1588500	0.0654660	1.8292520
N	2.1175760	3.1078840	-0.3712400	N	1.9099510	3.2265930	-0.4088710
C	3.1646260	3.9005760	-1.0130370	C	2.7308510	4.1712690	-1.1740980
H	2.7339020	4.8061090	-1.4463830	H	2.1533700	5.0755670	-1.3720730

[1] (a) Becke, A. D. *J. Chem. Phys.* **1993**, *98*, 5648–5652. (b) Becke, A. D. *Phys. Rev. A* **1998**, *38*, 3098–3100. (c) Lee, C.; Yang, W.; Parr, R. G. *Phys. Rev. B* **1998**, *37*, 785–789.

[2] Gaussian 09, Revision D.01, M. J. Frisch, G. W. Trucks, H. B. Schlegel, G. E. Scuseria, M. A. Robb, J. R. Cheeseman, G. Scalmani, V. Barone, B. Mennucci, G. A. Petersson, H. Nakatsuji, M. Caricato, X. Li, H. P. Hratchian, A. F. Izmaylov, J. Bloino, G. Zheng, J. L. Sonnenberg, M. Hada, M. Ehara, K. Toyota, R. Fukuda, J. Hasegawa, M. Ishida, T. Nakajima, Y. Honda, O. Kitao, H. Nakai, T. Vreven, J. A. Montgomery, Jr., J. E. Peralta, F. Ogliaro, M. Bearpark, J. J. Heyd, E. Brothers, K. N. Kudin, V. N. Staroverov, T. Keith, R. Kobayashi, J. Normand, K. Raghavachari, A. Rendell, J. C. Burant, S. S. Iyengar, J. Tomasi, M. Cossi, N. Rega, J. M. Millam, M. Klene, J. E. Knox, J. B. Cross, V. Bakken, C. Adamo, J. Jaramillo, R. Gomperts, R. E. Stratmann, O. Yazyev, A. J. Austin, R. Cammi, C. Pomelli, J. W. Ochterski, R. L. Martin, K. Morokuma, V. G. Zakrzewski, G. A. Voth, P. Salvador, J. J. Dannenberg, S. Dapprich, A. D. Daniels, O. Farkas, J. B. Foresman, J. V. Ortiz, J. Cioslowski, and D. J. Fox, Gaussian, Inc., Wallingford CT, **2013**.

H	3.9295830	4.1690290	-0.2807460	H	3.6265200	4.4184560	-0.6023750
H	3.6225540	3.3067720	-1.8043220	H	3.0168060	3.7061650	-2.1164130
C	-2.4008280	-2.4047540	-0.0841990	C	-2.3435180	-2.4426490	-0.1572840
O	-3.5529840	-1.9918160	-0.0195420	O	-3.4986610	-2.0372250	-0.2831220
O	-2.1191920	-3.7216800	-0.2585330	O	-2.0587660	-3.7735410	-0.1387420
C	-3.2580100	-4.5857070	-0.3454680	C	-3.1793070	-4.6603610	-0.2888050
H	-3.8924170	-4.3119780	-1.1935460	H	-3.6828510	-4.4928920	-1.2445750
H	-2.8504390	-5.5883890	-0.4812600	H	-2.7591460	-5.6656920	-0.2546700
H	-3.8558890	-4.5372120	0.5694400	H	-3.8969560	-4.5226060	0.5242480
Molecule **2a**<br>(PCM for DMSO)<br><br>**E =** -1164.56072246, **H (0K)** = -1164.195218,<br>**H (298K)** = -1164.170676, **G (298K)** = -1164.251590 au.<br>Imaginary frequency = 0.				Molecule **3a**<br>(gas phase)<br><br>**E =** -1164.52304147, **H (0K)** = -1164.157250,<br>**H (298K)** = -1164.132737, **G (298K)** = -1164.212817 au.<br>Imaginary frequency = 0.			
C	-1.3712710	-3.4535880	0.8622790	C	1.3528810	3.4083800	0.9406500
C	-0.6925040	-2.3239280	1.2125390	C	0.8169760	2.2143420	1.2987960
N	-0.7983400	-1.4354010	0.1520910	N	0.8360680	1.4234120	0.1470560
C	-1.5209620	-2.0220080	-0.8145880	C	1.3605730	2.0824370	-0.9440210
C	-0.2507500	-0.1128730	0.0913190	C	0.3514400	0.0899740	0.0995270
C	-0.9781040	1.1085390	0.0752700	C	1.1448200	-1.0637710	0.0699950
N	-0.1133580	2.1380310	-0.0083570	C	-1.0312970	-1.6830910	-0.0052000
C	1.1505990	1.6055180	-0.0294530	C	-1.0212110	-0.2836770	0.0526230
C	1.1226030	0.1852280	0.0285720	C	2.5995620	-1.2628060	0.0834220
C	-2.4364350	1.3295390	0.1273740	C	3.1711230	-2.3657610	-0.5786150
C	-3.0007660	2.4457190	-0.5230120	C	4.5486380	-2.5828820	-0.5469150
C	-4.3744060	2.6877780	-0.4773990	C	5.3842200	-1.6976090	0.1388720
C	-5.2245390	1.8172550	0.2151930	C	4.8283530	-0.5934980	0.7915220
C	-4.6796090	0.7081240	0.8693720	C	3.4511430	-0.3762390	0.7672350
C	-3.3031480	0.4687360	0.8297530	C	-2.1633400	0.6590140	0.0529150
C	2.2244190	-0.8052930	0.0340110	C	-2.2446740	1.6636770	-0.9260000
C	2.3158110	-1.7816980	-0.9743060	C	-3.3036220	2.5742770	-0.9215110
C	3.3311950	-2.7436090	-0.9555900	C	-4.2921950	2.5009170	0.0633480
C	4.2751300	-2.7489810	0.0752950	C	-4.2154360	1.5091710	1.0457960
C	4.1946410	-1.7843720	1.0868140	C	-3.1596690	0.5965000	1.0406210
C	3.1793970	-0.8262990	1.0667710	H	1.5221930	4.3039650	1.5193840
H	-1.5339530	-4.3759870	1.3962720	H	0.4247040	1.8646540	2.2410780
H	-0.1547530	-2.0684530	2.1105510	H	2.5410200	-3.0422140	-1.1495240
H	-1.7662200	-1.5753110	-1.7647080	H	4.9690530	-3.4373990	-1.0691420
H	-2.3464200	3.1185900	-1.0676220	H	6.4571890	-1.8636960	0.1607010
H	-4.7838390	3.5544180	-0.9895120	H	5.4691510	0.1007920	1.3274480
H	-6.2941150	2.0032200	0.2474220	H	3.0331360	0.4784890	1.2867420
H	-5.3244650	0.0310730	1.4229000	H	-1.4757890	1.7247050	-1.6900820
H	-2.9018140	-0.3798370	1.3745220	H	-3.3552010	3.3405590	-1.6899150
H	1.5948540	-1.7779660	-1.7871450	H	-5.1145560	3.2107890	0.0668150
H	3.3848250	-3.4844400	-1.7485770	H	-4.9767870	1.4473020	1.8185000
H	5.0647000	-3.4947670	0.0914350	H	-3.1035940	-0.1708840	1.8063190
H	4.9214410	-1.7811920	1.8945340	N	1.6719800	3.3017380	-0.4120760
H	3.1215650	-0.0832900	1.8563840	C	2.2724190	4.3747740	-1.1916380
N	-1.8852570	-3.2421780	-0.4051680	H	1.6297090	5.2612630	-1.1879450
C	-2.6891350	-4.2008670	-1.1719440	H	3.2545530	4.6431760	-0.7885170
H	-2.1064310	-5.1074370	-1.3406740	H	2.3879430	4.0147870	-2.2137280
H	-3.5962460	-4.4383490	-0.6146880	C	-2.0493940	-2.7242920	-0.1220630
H	-2.9538490	-3.7519290	-2.1279430	O	-1.7675240	-3.9168210	-0.1706650
C	2.3340190	2.4489130	-0.1638670	O	-3.3100100	-2.2552020	-0.1805720
O	3.4886160	2.0484120	-0.3174150	C	-4.3421480	-3.2483620	-0.3243300
O	2.0503110	3.7796110	-0.1112970	H	-4.3307350	-3.9379220	0.5232910
C	3.1676190	4.6721480	-0.2634900	H	-5.2758130	-2.6875390	-0.3543450
H	3.6508530	4.5279230	-1.2332510	H	-4.2015910	-3.8135590	-1.2488150
H	2.7474090	5.6756910	-0.1976210	N	0.2772830	-2.1128770	-0.0005860
H	3.9009000	4.5168170	0.5319800	H	0.5134200	-3.0958510	0.0006990

Molecule **3a** (PCM for CH_2Cl_2)				Molecule **3a** (PCM for DMSO)			
**E =** -1164.53778827, **H (0K)** = -1164.172390, **H (298K)** = -1164.147694, **G (298K)** = -1164.228713 au				**E** = -1164.54189341, **H (0K)** = -1164.176284, **H (298K)** = -1164.151710, **G (298K)** = -1164.232045 au			
Imaginary frequency = 0.				Imaginary frequency = 0.			
C	1.2255940	3.5118130	0.9024250	C	1.3375670	3.4089720	0.9052120
C	0.6727980	2.3243720	1.2579210	C	0.7796470	2.2244280	1.2627010
N	0.7492110	1.5100680	0.1252810	N	0.8434620	1.4110590	0.1286280
C	1.3302460	2.1504210	-0.9473860	C	1.4225760	2.0497640	-0.9457440
C	0.2668390	0.1756310	0.0803590	C	0.3530660	0.0793840	0.0844530
C	1.0625410	-0.9767490	0.0643790	C	1.1410120	-1.0793360	0.0636550
C	-1.1113600	-1.6030390	-0.0168790	C	-1.0360330	-1.6891650	-0.0155350
C	-1.1031200	-0.2029220	0.0319290	C	-1.0196570	-0.2890270	0.0385360
C	2.5195840	-1.1656490	0.0994540	C	2.5973470	-1.2761090	0.0885580
C	3.1157650	-2.2349110	-0.5957680	C	3.1830560	-2.3484790	-0.6110800
C	4.4958980	-2.4371460	-0.5436830	C	4.5630780	-2.5552890	-0.5714250
C	5.3067460	-1.5710600	0.1958150	C	5.3837560	-1.6915180	0.1603580
C	4.7250700	-0.5014130	0.8843120	C	4.8121910	-0.6196830	0.8542080
C	3.3452060	-0.2992230	0.8397710	C	3.4325460	-0.4124510	0.8218550
C	-2.2540470	0.7290410	0.0325860	C	-2.1608490	0.6569210	0.0498370
C	-2.3447100	1.7414120	-0.9380540	C	-2.2992810	1.6095750	-0.9736450
C	-3.4130670	2.6422140	-0.9288810	C	-3.3577010	2.5224770	-0.9582600
C	-4.4023860	2.5503280	0.0545780	C	-4.2888800	2.5018530	0.0845020
C	-4.3175210	1.5494470	1.0283790	C	-4.1551550	1.5618220	1.1121630
C	-3.2526570	0.6467630	1.0172150	C	-3.0991750	0.6480070	1.0954450
H	1.3698960	4.4188080	1.4696300	H	1.4932570	4.3137700	1.4727460
H	0.2408090	1.9908540	2.1885200	H	0.3563030	1.8905220	2.1970990
H	2.5048600	-2.8972220	-1.2023760	H	2.5639260	-3.0112080	-1.2086370
H	4.9373320	-3.2649140	-1.0907180	H	4.9964220	-3.3858110	-1.1206670
H	6.3808460	-1.7260420	0.2331260	H	6.4574650	-1.8508360	0.1886160
H	5.3462320	0.1751050	1.4639660	H	5.4407970	0.0539940	1.4290440
H	2.9057110	0.5247540	1.3909430	H	3.0012380	0.4125550	1.3778630
H	-1.5787170	1.8167870	-1.7036180	H	-1.5784550	1.6295570	-1.7852610
H	-3.4714330	3.4138900	-1.6912150	H	-3.4533740	3.2482110	-1.7608180
H	-5.2322470	3.2511300	0.0623640	H	-5.1103480	3.2122850	0.0978090
H	-5.0808150	1.4710940	1.7973420	H	-4.8712180	1.5413200	1.9287540
H	-3.1924480	-0.1274630	1.7752770	H	-2.9981180	-0.0769250	1.8972980
N	1.6141900	3.3801040	-0.4289720	N	1.7176130	3.2769130	-0.4284260
C	2.2563590	4.4472640	-1.1893340	C	2.3586190	4.3440420	-1.1909960
H	1.6139590	5.3318120	-1.2229680	H	1.7091380	5.2226020	-1.2376500
H	3.2141090	4.7175370	-0.7351730	H	3.3084750	4.6259400	-0.7280930
H	2.4263080	4.0842470	-2.2024360	H	2.5426290	3.9757310	-2.1997280
C	-2.2077010	-2.5600820	-0.1302050	C	-2.0750530	-2.7094410	-0.1226090
O	-3.3974900	-2.2872600	-0.1937760	O	-1.8205820	-3.9126350	-0.1422850
O	-1.7379280	-3.8350860	-0.1645250	O	-3.3192080	-2.2130910	-0.2096090
C	-2.7255610	-4.8797600	-0.2891580	C	-4.3898980	-3.1765150	-0.3216820
H	-3.2848450	-4.7636730	-1.2198730	H	-4.4048110	-3.8309480	0.5522620
H	-2.1605380	-5.8106960	-0.2961670	H	-5.3021630	-2.5843470	-0.3717230
H	-3.4134600	-4.8548260	0.5585950	H	-4.2700950	-3.7731550	-1.2283230
N	0.2009900	-2.0310840	-0.0065060	N	0.2710400	-2.1253950	-0.0069160
H	0.4639520	-3.0065340	0.0039170	H	0.5157720	-3.1068760	-0.0014880
Molecule **2b** (gas phase)				Molecule **2b** (PCM for CH_2Cl_2)			
**E** = -1624.11505221, **H (0K)** = -1623.759468, **H (298K)** = -1623.733593, **G (298K)** = -1623.818095 au.				**E** = -1624.14932895, **H (0K)** = -1623.793338, **H (298K)** = -1623.767560, **G (298K)** = -1623.851688 au			
Imaginary frequency = 0.				Imaginary frequency = 0.			
C	-0.0160910	-3.7699300	0.6864610	C	-0.0506920	-3.7219200	0.8532880
C	0.5503130	-2.5698360	1.0055570	C	0.3423900	-2.4683360	1.2197650

N	0.0751860	-1.6318580	0.1018950	N	0.0883280	-1.6270350	0.1466790
C	-0.7486170	-2.2554250	-0.7488820	C	-0.4401300	-2.3610740	-0.8440130
C	0.3684310	-0.2341880	0.0860030	C	0.3410410	-0.2172400	0.0898250
C	-0.5793130	0.8257450	0.0423560	C	-0.6269960	0.8238780	0.0536360
N	0.0755570	1.9958860	-0.0313990	N	0.0036340	2.0110280	-0.0303230
C	1.4106780	1.7178550	-0.0072570	C	1.3487130	1.7566520	-0.0300350
C	1.6650890	0.3226820	0.0616620	C	1.6211720	0.3634880	0.0414850
C	-2.0491710	0.7783880	0.0937420	C	-2.0975960	0.7426190	0.0850870
C	-2.7985200	1.7725950	-0.5661030	C	-2.8650280	1.7601160	-0.5171220
C	-4.1917130	1.7699130	-0.5397540	C	-4.2585090	1.7305280	-0.4996710
C	-4.8629810	0.7602400	0.1531840	C	-4.9087390	0.6649700	0.1251010
C	-4.1545000	-0.2269330	0.8360150	C	-4.1850110	-0.3557460	0.7385810
C	-2.7579100	-0.2082640	0.8066070	C	-2.7890750	-0.3085300	0.7182220
C	2.9327490	-0.4415110	0.0514160	C	2.9064240	-0.3743670	0.0635660
C	3.1651120	-1.4225620	-0.9304450	C	3.2343940	-1.2708180	-0.9689940
C	4.3342440	-2.1884090	-0.9268310	C	4.4262800	-2.0019090	-0.9371470
C	5.2991280	-1.9883960	0.0632190	C	5.3137270	-1.8516770	0.1319820
C	5.0861050	-1.0106530	1.0410340	C	4.9999300	-0.9627920	1.1667240
C	3.9186850	-0.2468230	1.0354590	C	3.8089940	-0.2351470	1.1331520
H	0.0936040	-4.7442830	1.1352160	H	-0.0255900	-4.6565220	1.3901260
H	1.2458130	-2.2932580	1.7805740	H	0.7715960	-2.0974420	2.1358290
H	-1.2717200	-1.7763760	-1.5604510	H	-0.7356640	-1.9796410	-1.8083530
H	-2.2630100	2.5556000	-1.0924550	H	-2.3517110	2.5829290	-1.0023580
H	-4.7538980	2.5423710	-1.0538800	H	-4.8311920	2.5222600	-0.9709820
H	-4.6857420	-0.9843150	1.4031710	H	-4.6990090	-1.1695750	1.2388240
H	-2.2179920	-0.9490990	1.3896190	H	-2.2430760	-1.0909930	1.2341040
H	2.4338270	-1.5624790	-1.7226770	H	2.5586920	-1.3810770	-1.8128200
H	4.4966550	-2.9293630	-1.7054010	H	4.6621660	-2.6828970	-1.7503900
H	6.2122270	-2.5770220	0.0661780	H	6.2410030	-2.4168660	0.1580800
H	5.8366920	-0.8358920	1.8070000	H	5.6835310	-0.8371240	2.0019050
H	3.7697170	0.5231640	1.7842200	H	3.5723720	0.4534230	1.9387170
N	-0.8352810	-3.5544240	-0.4085300	N	-0.5414050	-3.6323200	-0.4379210
C	2.4220420	2.7788430	-0.0929240	C	2.3340460	2.8286860	-0.1562810
O	3.6324250	2.6079410	-0.0092280	O	3.5495960	2.6732330	-0.2652390
O	1.8807590	4.0092630	-0.2810260	O	1.7752800	4.0691620	-0.1522820
C	2.8208240	5.0876270	-0.3576880	C	2.6851850	5.1720920	-0.2981090
H	3.5092220	4.9464560	-1.1959160	H	3.2228610	5.1095350	-1.2478560
H	2.2192140	5.9855700	-0.5042220	H	2.0617670	6.0660120	-0.2755730
H	3.4028370	5.1640300	0.5653690	H	3.4064420	5.1932640	0.5229850
Cl	-6.6261050	0.7405540	0.1789120	Cl	-6.6751610	0.6130840	0.1447810
C	-1.6620320	-4.5583250	-1.0769830	C	-1.0798280	-4.7454510	-1.2276860
H	-1.0330020	-5.3717230	-1.4452010	H	-0.3195610	-5.5219330	-1.3214010
H	-2.4060930	-4.9520920	-0.3807090	H	-1.9649650	-5.1478930	-0.7327760
H	-2.1735930	-4.0913540	-[illegible]	H	-1.3489840	-4.3768830	-[illegible]
Molecule **2b**<br>(PCM for THF)<br><br>**E** = -1624.14784968, **H (0K)** = --1623.791792, **H (298K)** = -1623.766054, **G (298K)** = -1623.849713 au.<br>Imaginary frequency = 0.				Molecule **2b**<br>(PCM for DMSO)<br><br>**E** =-1624.15589001, **H (0K)** = -1623.799929, **H (298K)** = -1623.774139, **G (298K)** = -1623.858531 au<br>Imaginary frequency = 0.			
C	-0.0552700	-3.7263730	0.8401900	C	-0.0505490	-3.7179880	0.8617780
C	0.3554080	-2.4779930	1.2053220	C	0.3253240	-2.4606620	1.2323430
N	0.0852470	-1.6282370	0.1429090	N	0.0876840	-1.6251610	0.1502400
C	-0.4705030	-2.3521350	-0.8400980	C	-0.4144180	-2.3673540	-0.8490450
C	0.3434610	-0.2194410	0.0894220	C	0.3369940	-0.2151180	0.0921200
C	-0.6217000	0.8242350	0.0526560	C	-0.6318980	0.8253780	0.0567720
N	0.0121080	2.0095180	-0.0313740	N	-0.0029490	2.0139810	-0.0261970
C	1.3561810	1.7517040	-0.0297400	C	1.3439210	1.7604490	-0.0289170
C	1.6255200	0.3578590	0.0423530	C	1.6155400	0.3670600	0.0416710
C	-2.0923110	0.7469770	0.0862840	C	-2.1030110	0.7409710	0.0844320
C	-2.8578820	1.7660930	-0.5154700	C	-2.8721400	1.7522960	-0.5258260

C	-4.2513630	1.7399220	-0.4960810	C	-4.2657900	1.7193210	-0.5102580
C	-4.9035230	0.6764760	0.1303260	C	-4.9132160	0.6555440	0.1202480
C	-4.1815560	-0.3452580	0.7441400	C	-4.1877820	-0.3608430	0.7390810
C	-2.7855690	-0.3015810	0.7218020	C	-2.7920430	-0.3097750	0.7207190
C	2.9090990	-0.3823770	0.0650410	C	2.9022500	-0.3694830	0.0643210
C	3.2256070	-1.2964880	-0.9556410	C	3.2503090	-1.2339380	-0.9885810
C	4.4160100	-2.0297180	-0.9233610	C	4.4452080	-1.9605660	-0.9579100
C	5.3140100	-1.8640380	0.1345700	C	5.3141580	-1.8380220	0.1300460
C	5.0120680	-0.9571810	1.1572060	C	4.9782980	-0.9833420	1.1866380
C	3.8225730	-0.2274550	1.1231680	C	3.7841230	-0.2602520	1.1545130
H	-0.0258310	-4.6642610	1.3710450	H	-0.0308780	-4.6503710	1.4025610
H	0.8071830	-2.1163610	2.1142050	H	0.7310800	-2.0839560	2.1566640
H	-0.7841160	-1.9624840	-1.7953900	H	-0.6918310	-1.9929000	-1.8214340
H	-2.3426990	2.5874790	-1.0011760	H	-2.3621420	2.5723910	-1.0192460
H	-4.8227770	2.5327900	-0.9670740	H	-4.8397120	2.5059000	-0.9885950
H	-4.6972240	-1.1568590	1.2462940	H	-4.6992850	-1.1756510	1.2400900
H	-2.2403880	-1.0843630	1.2383000	H	-2.2450330	-1.0906420	1.2374640
H	2.5422120	-1.4183320	-1.7916310	H	2.5869420	-1.3260910	-1.8441890
H	4.6428180	-2.7241220	-1.7278390	H	4.6961390	-2.6186260	-1.7852460
H	6.2403920	-2.4306890	0.1611330	H	6.2426570	-2.4011300	0.1555890
H	5.7042760	-0.8185310	1.9832110	H	5.6453800	-0.8830380	2.0384100
H	3.5957610	0.4759690	1.9185350	H	3.5283670	0.3988710	1.9789150
N	-0.5744610	-3.6250410	-0.4389760	N	-0.5138470	-3.6367430	-0.4399130
C	2.3438850	2.8219760	-0.1572510	C	2.3276350	2.8317920	-0.1564870
O	3.5593710	2.6644900	-0.2609300	O	3.5433420	2.6775490	-0.2779820
O	1.7863670	4.0628750	-0.1612000	O	1.7711540	4.0737050	-0.1396900
C	2.6982470	5.1636870	-0.3082580	C	2.6808190	5.1785920	-0.2835820
H	3.2402690	5.0958960	-1.2552320	H	3.2123360	5.1224990	-1.2369560
H	2.0759070	6.0585480	-0.2927060	H	2.0576420	6.0722050	-0.2515560
H	3.4160980	5.1876780	0.5157760	H	3.4057620	5.1943390	0.5342010
Cl	-6.6700030	0.6284530	0.1517630	Cl	-6.6797770	0.5995980	0.1383750
C	-1.1322680	-4.7297870	-1.2268900	C	-1.0289420	-4.7579540	-1.2348250
H	-0.3604300	-5.4827330	-1.3926050	H	-0.2633260	-5.5316760	-1.3022240
H	-1.9732910	-5.1702400	-0.6894690	H	-1.9241640	-5.1588900	-0.7573950
H	-1.4750180	-4.3403010	-[illegible]	H	-1.2746610	-4.3976050	-[illegible]
Molecule **3b** (gas phase)				Molecule **3b** (PCM for CH_2Cl_2)			
**E** = -1624.11720520, **H (0K)** = -1623.761110, **H (298K)** = -1623.735317, **G (298K)** = -1623.818914 au. Imaginary frequency = 0.				**E** =--1624.13287787, **H (0K)** = -1623.777004, **H (298K)** = -1623.751121, **G (298K)** = -1623.835486 au Imaginary frequency = 0.			
C	0.2385280	3.6385230	0.9282670	C	0.2400140	3.6346800	0.9012710
C	-0.1304370	2.3818280	1.2828470	C	-0.1327820	2.3788660	1.2558660
N	0.0352200	1.5960580	0.1390510	N	0.0429790	1.5895850	0.1162440
C	0.4916350	2.3180460	-0.9431520	C	0.5123120	2.3118260	-0.9597550
C	-0.2468210	0.2061330	0.0907880	C	-0.2413560	0.1996720	0.0699210
C	0.7100150	-0.8151110	0.0498550	C	0.7126850	-0.8257480	0.0361230
C	-1.3479080	-1.7543560	-0.0099110	C	-1.3460790	-1.7594120	-0.0233990
C	-1.5481470	-0.3693250	0.0530530	C	-1.5426960	-0.3732150	0.0362660
C	2.1768400	-0.7883590	0.0515940	C	2.1804790	-0.7989740	0.0467960
C	2.9098700	-1.7879630	-0.6138030	C	2.9177650	-1.7881210	-0.6299580
C	4.3039280	-1.7944980	-0.5985560	C	4.3122850	-1.7904990	-0.6075130
C	4.9813920	-0.7838710	0.0828400	C	4.9804950	-0.7869670	0.0932300
C	4.2815720	0.2265840	0.7434520	C	4.2780530	0.2116090	0.7686960
C	2.8884530	0.2197690	0.7264280	C	2.8843320	0.2000430	0.7438410
C	-2.8183510	0.3920880	0.0599160	C	-2.8138450	0.3883870	0.0615830
C	-3.0468880	1.3811900	-0.9115710	C	-3.0928130	1.3292910	-0.9438470
C	-4.2302010	2.1227720	-0.9042370	C	-4.2772100	2.0705320	-0.9157300
C	-5.1987280	1.8943610	0.0767500	C	-5.1963720	1.8886530	0.1218130
C	-4.9764660	0.9178210	1.0522110	C	-4.9237690	0.9596880	1.1316720
C	-3.7959650	0.1734240	1.0440160	C	-3.7418750	0.2166410	1.1021600

H	0.2603990	4.5522670	1.5028890	H	0.2616350	4.5502540	1.4725940
H	-0.4949500	1.9843750	2.2172880	H	-0.4978290	1.9818330	2.1901630
H	2.3930790	-2.5574570	-1.1799660	H	2.4071290	-2.5547260	-1.2046580
H	4.8571950	-2.5672330	-1.1207490	H	4.8683350	-2.5556480	-1.1375940
H	4.8203350	1.0063580	1.2704470	H	4.8096430	0.9839750	1.3133650
H	2.3517810	1.0017360	1.2510020	H	2.3440580	0.9691150	1.2836050
H	-2.2948200	1.5626710	-1.6732880	H	-2.3814670	1.4742560	-1.7509890
H	-4.3942960	2.8787440	-1.6669800	H	-4.4801730	2.7889740	-1.7047760
H	-6.1181270	2.4729770	0.0827950	H	-6.1161010	2.4660300	0.1447100
H	-5.7215920	0.7366670	1.8219060	H	-5.6300030	0.8148830	1.9443030
H	-3.6275470	-0.5833270	1.8036680	H	-3.5339180	-0.5008840	1.8899040
N	0.6090600	3.5719090	-0.4139230	N	0.6247950	3.5665610	-0.4359420
C	-2.1981750	-2.9380850	-0.1221290	C	-2.2159110	-2.9288810	-0.1259760
O	-1.7376140	-4.0728650	-0.1822540	O	-1.7768950	-4.0768080	-0.1550330
O	-3.5153820	-2.6661580	-0.1617300	O	-3.5225500	-2.6310600	-0.1968530
C	-4.3870380	-3.8037990	-0.3015970	C	-4.4309630	-3.7480450	-0.3093780
H	-4.2587960	-4.4896970	0.5392600	H	-4.3367640	-4.4033740	0.5590420
H	-5.3951210	-3.3907260	-0.3140760	H	-5.4244460	-3.3042460	-0.3489010
H	-4.1753680	-4.3337820	-1.2332990	H	-4.2264330	-4.3129760	-1.2211970
N	0.0101180	-1.9832080	-0.0187300	N	0.0114650	-1.9922270	-0.0296780
H	0.3892410	-2.9204840	-0.0204820	H	0.3996550	-2.9261610	-0.0310790
Cl	6.7388450	-0.7803900	0.1060230	Cl	6.7421080	-0.7784770	0.1243760
C	1.0665100	4.7177360	-1.1875920	C	1.0921580	4.7201310	-1.1978770
H	0.2916250	5.4902130	-1.2287440	H	0.3164660	5.4901420	-1.2396860
H	1.9737620	5.1440150	-0.7471180	H	1.9910510	5.1420630	-0.7392140
H	1.2842820	4.3687380	-2.1966920	H	1.3247790	4.3851740	-2.2080990
Molecule **3b** (PCM for THF)				Molecule **3b** (PCM for DMSO)			
**E** = -1624.13605861, **H (0K)** = --1623.780118, **H (298K)** = -1623.754278, **G (298K)** = -1623.838113 au. Imaginary frequency = 0.				**E** =--1624.13214875, **H (0K)** = -1623.776234, **H (298K)** = -1623.750358, **G (298K)** = -1623.834508 au Imaginary frequency = 0.			
C	0.2450200	3.6338320	0.9036160	C	0.2415450	3.6316910	0.9019940
C	-0.1217090	2.3771450	1.2614410	C	-0.1288370	2.3750780	1.2565980
N	0.0435470	1.5887980	0.1195980	N	0.0449340	1.5867940	0.1160270
C	0.4996470	2.3123570	-0.9612310	C	0.5109790	2.3111980	-0.9595260
C	-0.2406730	0.1987100	0.0740960	C	-0.2394580	0.1968050	0.0685910
C	0.7136740	-0.8263110	0.0392790	C	0.7138180	-0.8296970	0.0366550
C	-1.3450070	-1.7606110	-0.0194730	C	-1.3453840	-1.7614070	-0.0247330
C	-1.5420800	-0.3744210	0.0408650	C	-1.5408130	-0.3751220	0.0337510
C	2.1813680	-0.7987170	0.0482210	C	2.1818300	-0.8024300	0.0476270
C	2.9188510	-1.7903070	-0.6246870	C	2.9198100	-1.7871870	-0.6349820
C	4.3133390	-1.7915970	-0.6034130	C	4.3144720	-1.7871310	-0.6137720
C	4.9815100	-0.7844900	0.0922270	C	4.9812310	-0.7857300	0.0915170
C	4.2788690	0.2168130	0.7633590	C	4.2782300	0.2079760	0.7736440
C	2.8852060	0.2042360	0.7396440	C	2.8843550	0.1939070	0.7500950
C	-2.8130450	0.3869970	0.0656890	C	-2.8118040	0.3872620	0.0593900
C	-3.0789280	1.3464320	-0.9259110	C	-3.1037050	1.3084910	-0.9604770
C	-4.2627980	2.0882460	-0.8991020	C	-4.2879190	2.0505030	-0.9319010
C	-5.1947470	1.8890730	0.1237070	C	-5.1938360	1.8884850	0.1206680
C	-4.9352040	0.9419590	1.1200670	C	-4.9083520	0.9788460	1.1447190
C	-3.7538340	0.1981800	1.0917610	C	-3.7264770	0.2353910	1.1148800
H	0.2712660	4.5491370	1.4752380	H	0.2651800	4.5466540	1.4740440
H	-0.4761160	1.9790580	2.1993990	H	-0.4882510	1.9761720	2.1922550
H	2.4084610	-2.5594700	-1.1961240	H	2.4101020	-2.5531130	-1.2112910
H	4.8696150	-2.5584850	-1.1307580	H	4.8709710	-2.5493220	-1.1475880
H	4.8105600	0.9922370	1.3035680	H	4.8085300	0.9778380	1.3230530
H	2.3445360	0.9761990	1.2748380	H	2.3435490	0.9579210	1.2964740
H	-2.3569270	1.5059880	-1.7207350	H	-2.4032030	1.4375400	-1.7798280
H	-4.4553510	2.8209100	-1.6776430	H	-4.5010370	2.7537050	-1.7318870
H	-6.1141040	2.4670950	0.1458970	H	-6.1132330	2.4662610	0.1441090

H	-5.6513460	0.7834310	1.9214210	H	-5.6042670	0.8497250	1.9687210
H	-3.5562350	-0.5335300	1.8689740	H	-3.5082290	-0.4666000	1.9137880
N	0.6153100	3.5671050	-0.4378180	N	0.6232490	3.5656240	-0.4359990
C	-2.2133570	-2.9309090	-0.1266900	C	-2.2198870	-2.9275690	-0.1233980
O	-1.7728630	-4.0781130	-0.1573920	O	-1.7863660	-4.0781490	-0.1440470
O	-3.5202370	-2.6346390	-0.2008860	O	-3.5244220	-2.6237490	-0.2015100
C	-4.4262720	-3.7522220	-0.3233280	C	-4.4402120	-3.7366990	-0.3033420
H	-4.3356170	-4.4119820	0.5421620	H	-4.3504850	-4.3831210	0.5720470
H	-5.4201500	-3.3094840	-0.3658070	H	-5.4309200	-3.2873970	-0.3472530
H	-4.2162500	-4.3124860	-1.2368110	H	-4.2393480	-4.3112750	-1.2098560
N	0.0126640	-1.9930060	-0.0258140	N	0.0119500	-1.9956420	-0.0292930
H	0.4004630	-2.9270650	-0.0292350	H	0.4011390	-2.9293040	-0.0278510
Cl	6.7430410	-0.7745950	0.1217060	Cl	6.7433900	-0.7741570	0.1205840
C	1.0674150	4.7226100	-1.2057160	C	1.0884580	4.7219130	-1.1963340
H	0.2844630	5.4854680	-1.2447550	H	0.3125810	5.4917060	-1.2325500
H	1.9654500	5.1540920	-0.7542280	H	1.9881470	5.1422680	-0.7382890
H	1.2962580	4.3867110	-2.2164680	H	1.3182350	4.3913310	-2.2087140
Molecule **2e** (gas phase)				Molecule **2e** (PCM for CH_2Cl_2)			
**E =** -1356.26941354, **H (0K)** = -1355.851604, **H (298K)** = -1355.824115, **G (298K)** = -1355.912554 au.				**E =** -1356.30060723, **H (0K)** = -1355.882519, **H (298K)** = -1355.855097, **G (298K)** = -1355.943304 au			
Imaginary frequency = 0.				Imaginary frequency = 0.			
C	-2.0508600	-1.8322600	1.2926730	C	2.2412230	-1.3233330	-1.5718240
C	-0.7396070	-1.4766540	1.4025070	C	0.9238620	-0.9998310	-1.6983400
N	-0.4907160	-0.4958880	0.4516590	N	0.5596800	-0.2906810	-0.5620590
C	-1.6219370	-0.2759220	-0.2263430	C	1.6305290	-0.1949890	0.2357550
C	0.7298310	0.2115560	0.2503020	C	-0.7290810	0.2666000	-0.2802830
C	0.8695380	1.6235760	0.1373230	C	-1.0435320	1.6466930	-0.1432910
N	2.1536940	1.9179510	-0.1168410	N	-2.3478930	1.7738560	0.1636540
C	2.8439700	0.7396700	-0.1434310	C	-2.8785230	0.5108110	0.2094970
C	2.0003200	-0.3794560	0.0775040	C	-1.8999340	-0.4823310	-0.0626340
C	-0.1453280	2.6810140	0.2924200	C	-0.1661900	2.8233920	-0.2942090
C	-0.0483540	3.8570130	-0.4770120	C	-0.4096980	3.9827290	0.4692920
C	-0.9890370	4.8785510	-0.3475730	C	0.3938480	5.1154610	0.3341910
C	-2.0538850	4.7557230	0.5526400	C	1.4675700	5.1200920	-0.5642350
C	-2.1540500	3.6027210	1.3367110	C	1.7183480	3.9799240	-1.3336960
C	-1.2088850	2.5806100	1.2119040	C	0.9096530	2.8474350	-1.2039450
C	2.2846810	-1.8322540	0.0757150	C	-2.0079550	-1.9596410	-0.1185100
C	1.6048540	-2.6894140	-0.8093440	C	-1.2925520	-2.7650180	0.7852280
C	1.8239040	-4.0696930	-0.7917000	C	-1.3572940	-4.1603320	0.7137310
C	2.7304400	-4.6233130	0.1155120	C	-2.1382700	-4.7777890	-0.2671980
C	3.4201020	-3.7826250	0.9959770	C	-2.8548370	-3.9881520	-1.1739750
C	3.2006560	-2.4049910	0.9763740	C	-2.7875950	-2.5956170	-1.1012470
H	-2.6446300	-2.5315880	1.8577340	H	2.9096950	-1.8444490	-2.2371610
H	0.0371530	-1.8173050	2.0667200	H	0.2201230	-1.1929770	-2.4909440
H	-1.7273440	0.4175390	-1.0434740	H	1.6451070	0.2767530	1.2047180
H	0.7852270	3.9523680	-1.1651070	H	-1.2396850	3.9813130	1.1681470
H	-0.8905510	5.7769840	-0.9511100	H	0.1845430	5.9962700	0.9352890
H	-2.7835940	5.5540270	0.6540090	H	2.0943510	6.0011020	-0.6675400
H	-2.9530750	3.5098130	2.0683310	H	2.5366110	3.9746640	-2.0487770
H	-1.2713790	1.7188120	1.8714370	H	1.1000440	1.9891650	-1.8410170
H	0.9197810	-2.2628530	-1.5377480	H	-0.6952120	-2.2935270	1.5609120
H	1.2969750	-4.7087180	-1.4956580	H	-0.8020690	-4.7624000	1.4278780
H	2.9082310	-5.6950310	0.1284770	H	-2.1915890	-5.8612870	-0.3234980
H	4.1388690	-4.2008100	1.6954200	H	-3.4663110	-4.4577980	-1.9396140
H	3.7532410	-1.7568430	1.6475890	H	-3.3469740	-1.9888110	-1.8069610
N	-2.5995270	-1.0614150	0.2757430	N	2.6720810	-0.8042660	-0.3576070
C	4.2843810	0.6938920	-0.4206790	C	-4.2747940	0.2724380	0.5661090
O	4.9758680	-0.3175950	-0.3980400	O	-4.7927700	-0.8326070	0.7232750
O	4.8042020	1.9127930	-0.7171660	O	-4.9898140	1.4191480	0.7267950

C	6.2110400	1.9298490	-0.9852010	C	-6.3707950	1.2552760	1.0898410
H	6.4524070	1.2984750	-1.8452720	H	-6.4629790	0.7301380	2.0441210
H	6.4525000	2.9723400	-1.1968250	H	-6.7693720	2.2663070	1.1750410
H	6.7776530	1.5744370	-0.1195450	H	-6.9118590	0.6989210	0.3198980
C	-3.9633800	-1.0700590	-0.1639980	C	4.0024860	-0.8937900	0.1815220
C	-4.6143420	-2.2911750	-0.3602570	C	4.6556600	-2.1285680	0.1827690
C	-4.6184500	0.1439720	-0.3915980	C	4.6129890	0.2547520	0.6913190
C	-5.9439490	-2.2921830	-0.7848070	C	5.9488770	-2.2085050	0.7032710
H	-4.0833370	-3.2247790	-0.2041220	H	4.1582270	-3.0134310	-0.2002850
C	-5.9426820	0.1273990	-0.8329890	C	5.9009010	0.1567670	1.2224640
H	-4.1079670	1.0843920	-0.2074260	H	4.0996320	1.2102200	0.6586690
C	-6.6081280	-1.0862460	-1.0260620	C	6.5704350	-1.0704020	1.2264780
H	-6.4540190	-3.2375340	-0.9405760	H	6.4631730	-3.1640430	0.7095270
H	-6.4559680	1.0668820	-1.0117080	H	6.3824120	1.0442620	1.6200740
H	-7.6399440	-1.0925370	-1.3628410	H	7.5737500	-1.1395210	1.6345520

Molecule **2e**
(PCM for DMSO)

E = -1356.30683403, **H (0K)** = -1355.888780,
H (298K) = -1355.861342, **G (298K)** = -1355.949935
au Imaginary frequency = 0.

C	2.2564780	-1.2696320	-1.5894880
C	0.9381130	-0.9511500	-1.7145100
N	0.5660950	-0.2672690	-0.5647780
C	1.6341100	-0.1813030	0.2388370
C	-0.7294520	0.2707710	-0.2761730
C	-1.0661670	1.6454970	-0.1396610
N	-2.3733350	1.7523300	0.1652540
C	-2.8844960	0.4798280	0.2117080
C	-1.8879600	-0.4963320	-0.0588750
C	-0.2063330	2.8363450	-0.2858370
C	-0.4649290	3.9888510	0.4832170
C	0.3212730	5.1343050	0.3508440
C	1.3926480	5.1577970	-0.5500850
C	1.6594760	4.0237550	-1.3233910
C	0.8679320	2.8788850	-1.1966080
C	-1.9723660	-1.9752800	-0.1174350
C	-1.2493730	-2.7703900	0.7894340
C	-1.2944080	-4.1666020	0.7169910
C	-2.0624590	-4.7946460	-0.2676670
C	-2.7847100	-4.0150030	-1.1788520
C	-2.7369260	-2.6214990	-1.1055030
H	2.9301650	-1.7731810	-2.2630200
H	0.2393790	-1.1314440	-2.5145080
H	1.6434470	0.2698710	1.2176670
H	-1.2901530	3.9730850	1.1876570
H	0.1015730	6.0089240	0.9571850
H	2.0070670	6.0477540	-0.6504370
H	2.4785390	4.0316140	-2.0373070
H	1.0734660	2.0245220	-1.8338630
H	-0.6585790	-2.2912340	1.5653280
H	-0.7316680	-4.7609030	1.4315910
H	-2.0987930	-5.8787080	-0.3255350
H	-3.3827710	-4.4930090	-1.9498600
H	-3.2976750	-2.0225960	-1.8170500
N	2.6797830	-0.7728200	-0.3633400
C	-4.2764230	0.2215250	0.5669360
O	-4.7760950	-0.8903440	0.7426360
O	-5.0152330	1.3566960	0.7047470
C	-6.3940410	1.1742770	1.0718680
H	-6.4756440	0.6694640	2.0378510
H	-6.8118420	2.1789660	1.1350610

Molecule **3e**
(gas phase)

E = -1356.27250069, **H (0K)** = -1355.854062,
H (298K) = -1355.826583, **G (298K)** = -1355.914546
au.
Imaginary frequency = 0.

C	-2.2620810	-0.9915660	1.7144930
C	-0.9746360	-0.5875410	1.8281020
N	-0.6165790	-0.0751290	0.5766670
C	-1.6322330	-0.1460290	-0.3421460
C	0.6726410	0.4423840	0.2811780
C	0.9882600	1.7973620	0.1260210
C	2.8669190	0.5759880	-0.1972740
C	1.8442870	-0.3401120	0.0802270
C	0.1736330	3.0163430	0.2090710
C	0.4832580	4.1326700	-0.5903510
C	-0.2659650	5.3058420	-0.4995400
C	-1.3449150	5.3840690	0.3847920
C	-1.6669080	4.2781950	1.1769350
C	-0.9162660	3.1059580	1.0938240
C	1.9022330	-1.8188130	0.1410560
C	1.0518390	-2.5858230	-0.6732650
C	1.0750800	-3.9806110	-0.6086610
C	1.9415730	-4.6304950	0.2740130
C	2.7867710	-3.8755420	1.0928350
C	2.7667740	-2.4815560	1.0271980
H	-2.9284110	-1.4136970	2.4492290
H	-0.2946980	-0.6122090	2.6652130
H	1.2940440	4.0756170	-1.3113970
H	-0.0133940	6.1536660	-1.1296390
H	-1.9315660	6.2953820	0.4528580
H	-2.5039360	4.3287780	1.8672650
H	-1.1701240	2.2604820	1.7231670
H	0.3756930	-2.0844760	-1.3589660
H	0.4149250	-4.5580030	-1.2497150
H	1.9576450	-5.7155080	0.3252660
H	3.4608810	-4.3718640	1.7854860
H	3.4244180	-1.8999490	1.6655340
N	-2.6437730	-0.7157330	0.3964530
C	4.2834280	0.4977200	-0.5474650
O	4.9613280	1.4952130	-0.7687710
O	4.7641630	-0.7578840	-0.6116390
C	6.1499880	-0.8806960	-0.9821280
H	6.7862970	-0.3610990	-0.2616480
H	6.3528480	-1.9511630	-0.9766390
H	6.3173390	-0.4613540	-1.9771440

H	-6.9239350	0.5915950	0.3140500
C	4.0092060	-0.8679040	0.1780960
C	4.6659040	-2.1005510	0.1573950
C	4.6147490	0.2729820	0.7102160
C	5.9584010	-2.1861600	0.6789130
H	4.1722640	-2.9792490	-0.2440030
C	5.9021200	0.1691630	1.2418280
H	4.0981650	1.2270370	0.6945460
C	6.5755400	-1.0558990	1.2242360
H	6.4756990	-3.1400370	0.6679340
H	6.3801820	1.0506460	1.6566100
H	7.5785030	-1.1294150	1.6324820

N	2.3183400	1.8392410	-0.1691860
H	2.8823140	2.6671420	-0.3059610
C	-3.9321900	-1.0037690	-0.1458520
C	-4.6781780	-2.0846820	0.3376450
C	-4.4462210	-0.1988770	-1.1695800
C	-5.9433080	-2.3488950	-0.1932160
H	-4.2684550	-2.7322980	1.1058200
C	-5.7049060	-0.4791170	-1.7008300
H	-3.8479080	0.6262200	-1.5374740
C	-6.4623050	-1.5492990	-1.2135600
H	-6.5150660	-3.1908570	0.1860340
H	-6.0979770	0.1483520	-2.4955090
H	-7.4441270	-1.7590710	-1.6271710

Molecule **3e**
(PCM for CH_2Cl_2)

E = -1356.28797291, **H (0K)** = -1355.869798,
H (298K) = -1355.842250, **G (298K)** = -1355.930660
au Imaginary frequency = 0.

C	-2.2458880	-1.0310700	1.6914330
C	-0.9629950	-0.6124940	1.8060760
N	-0.6135080	-0.0825690	0.5596340
C	-1.6328910	-0.1550120	-0.3547030
C	0.6691480	0.4530620	0.2651810
C	0.9668820	1.8132710	0.1122510
C	2.8605370	0.6192050	-0.2163090
C	1.8516190	-0.3113570	0.0651740
C	0.1324180	3.0191250	0.2081280
C	0.3952820	4.1328450	-0.6119960
C	-0.3766910	5.2910660	-0.5088960
C	-1.4297250	5.3557890	0.4085210
C	-1.7029240	4.2525350	1.2237180
C	-0.9299230	3.0948680	1.1280390
C	1.9389550	-1.7886920	0.1448500
C	1.1520880	-2.5869520	-0.7024750
C	1.2042130	-3.9808650	-0.6178460
C	2.0376720	-4.5977680	0.3197340
C	2.8196030	-3.8113250	1.1725550
C	2.7696480	-2.4184470	1.0865540
H	-2.9070890	-1.4715380	2.4203580
H	-0.2836510	-0.6303570	2.6437350
H	1.1891610	4.0882450	-1.3520080
H	-0.1610660	6.1378460	-1.1536640
H	-2.0328060	6.2554400	0.4859460
H	-2.5166020	4.2944060	1.9418020
H	-1.1425020	2.2534730	1.7781540
H	0.5029180	-2.1121200	-1.4316270
H	0.5935660	-4.5827110	-1.2848150
H	2.0769210	-5.6811520	0.3870630
H	3.4670680	-4.2818800	1.9070500
H	3.3761320	-1.8131750	1.7532250
N	-2.6357220	-0.7428390	0.3785160
C	4.2766980	0.5415160	-0.5644290
O	4.9626360	1.5417730	-0.7655310
O	4.7479490	-0.7121970	-0.6545770
C	6.1420450	-0.8494590	-1.0051890
H	6.7715580	-0.3593030	-0.2595160

Molecule **3e**
(PCM for DMSO)

E = -1356.29117934, **H (0K)** = -1355.873109,
H (298K) = -1355.845541, **G (298K)** = -1355.933912
au Imaginary frequency = 0.

C	-2.2357880	-1.0471770	1.6898960
C	-0.9588840	-0.6096820	1.8022540
N	-0.6095850	-0.1009800	0.5470510
C	-1.6232300	-0.2068030	-0.3701860
C	0.6617170	0.4615710	0.2525530
C	0.9273700	1.8251270	0.0706960
C	2.8527670	0.6715280	-0.2128330
C	1.8644060	-0.2771210	0.0811420
C	0.0596700	3.0100810	0.1285160
C	0.2949640	4.1066920	-0.7226090
C	-0.5116120	5.2441550	-0.6571900
C	-1.5713920	5.3044120	0.2529410
C	-1.8163800	4.2181740	1.0996110
C	-1.0091010	3.0813640	1.0415520
C	1.9899490	-1.7491080	0.2027850
C	1.2543830	-2.5933440	-0.6459220
C	1.3442640	-3.9825880	-0.5206200
C	2.1649490	-4.5482710	0.4598510
C	2.8960190	-3.7156590	1.3141810
C	2.8081480	-2.3276260	1.1875290
H	-2.8779040	-1.5289120	2.4097040
H	-0.2776880	-0.6172900	2.6384970
H	1.0953510	4.0647530	-1.4555960
H	-0.3167650	6.0781870	-1.3247170
H	-2.2007180	6.1879360	0.3012300
H	-2.6339920	4.2576400	1.8132780
H	-1.1998400	2.2542420	1.7163540
H	0.6161320	-2.1586450	-1.4091160
H	0.7734280	-4.6207070	-1.1890820
H	2.2335860	-5.6276850	0.5588740
H	3.5327140	-4.1463520	2.0816890
H	3.3744970	-1.6861320	1.8556650
N	-2.6205900	-0.7963160	0.3678680
C	4.2746780	0.6179070	-0.5413590
O	4.9421250	1.6290880	-0.7523660
O	4.7733730	-0.6265010	-0.6028400
C	6.1768460	-0.7415960	-0.9267270
H	6.7826210	-0.2306750	-0.1755370

H	6.3281060	-1.9221920	-1.0190060	H	6.3835430	-1.8105140	-0.9226620
H	6.3304870	-0.4146800	-1.9890410	H	6.3746290	-0.3164790	-1.9128230
N	2.2946070	1.8751930	-0.1863780	N	2.2556940	1.9133490	-0.2177670
H	2.8374300	2.7172170	-0.3256350	H	2.7777890	2.7660860	-0.3714770
C	-3.9268830	-1.0419900	-0.1552610	C	-3.9125320	-1.1069710	-0.1592440
C	-4.6026360	-2.1995820	0.2483100	C	-5.0499220	-0.9547260	0.6424690
C	-4.5136290	-0.1720480	-1.0818840	C	-4.0360450	-1.5646780	-1.4764340
C	-5.8697620	-2.4792420	-0.2707480	C	-6.3097380	-1.2682800	0.1249330
H	-4.1381730	-2.8879680	0.9467250	H	-4.9575510	-0.5787400	1.6561250
C	-5.7737120	-0.4673200	-1.6050620	C	-5.3003850	-1.8625920	-1.9887670
H	-3.9795310	0.7221360	-1.3809750	H	-3.1462910	-1.6852770	-2.0834120
C	-6.4592350	-1.6176600	-1.2000410	C	-6.4410670	-1.7199850	-1.1913460
H	-6.3878320	-3.3797700	0.0451550	H	-7.1882640	-1.1465770	0.7513170
H	-6.2240490	0.2109470	-2.3237580	H	-5.3907120	-2.2179010	-3.0109510
H	-7.4418580	-1.8399380	-1.6048260	H	-7.4215070	-1.9588690	-1.5918240
Molecule **2i** (gas phase)				Molecule **2i** (PCM for CH_2Cl_2)			
**E =** -1855.18115962, **H (0K)** = -1854.744139, **H (298K)** = -1854.714016, **G (298K)** = --1854.810477 au.				**E =** -1855.21450100, **H (0K)** = -1854.777135, **H (298K)** = -1854.747060, **G (298K)** = -1854.843077 au			
Imaginary frequency = 0.				Imaginary frequency = 0.			
C	2.4192230	0.2594280	1.3405000	C	2.0685790	-1.0807860	1.4486770
C	1.1329870	0.6890960	1.4954170	C	1.0258740	-0.2209310	1.6296040
N	0.3868490	0.1579190	0.4543060	N	0.2819610	-0.2211800	0.4584770
C	1.2083340	-0.5659500	-0.3156770	C	0.8692130	-1.0595960	-0.4082230
C	-1.0171120	0.3094910	0.2390020	C	-0.9127180	0.5292670	0.2066680
C	-1.9546990	-0.7403650	0.0336940	C	-2.2190960	0.0079450	-0.0020260
N	-3.1641010	-0.2074490	-0.2060960	N	-3.0670580	1.0249680	-0.2442470
C	-3.0338000	1.1480090	-0.1313590	C	-2.3422760	2.1841050	-0.1775600
C	-1.6971320	1.5422960	0.1433500	C	-0.9711460	1.9309060	0.1030060
C	-1.7613980	-2.1982630	0.0791990	C	-2.6877180	-1.3887730	0.0190780
C	-2.5743310	-3.0305540	-0.7158390	C	-3.8097550	-1.7604670	-0.7483640
C	-2.4286120	-4.4162440	-0.7023900	C	-4.2953760	-3.0671450	-0.7450300
C	-1.4554410	-4.9958810	0.1143730	C	-3.6518870	-4.0300990	0.0341350
C	-0.6487720	-4.2057340	0.9314280	C	-2.5450030	-3.6991470	0.8135700
C	-0.8102380	-2.8182310	0.9128710	C	-2.0741230	-2.3841180	0.8042370
C	-1.0769290	2.8815480	0.2547920	C	0.1798980	2.8523080	0.2687410
C	0.0120960	3.2326770	-0.5644250	C	1.2357450	2.8544390	-0.6591560
C	0.6416430	4.4742850	-0.4405090	C	2.3414880	3.6940000	-0.4896590
C	0.1930190	5.3949800	0.5093070	C	2.4124220	4.5479020	0.6145800
C	-0.8942370	5.0637150	1.3250650	C	1.3690140	4.5546910	1.5473610
C	-1.5212020	3.8238250	1.1998860	C	0.2673030	3.7137480	1.3768430
H	3.3066910	0.4605780	1.9179630	H	2.8752780	-1.3600390	2.1064430
H	0.6793780	1.3260460	2.2367050	H	0.7424920	0.3832570	2.4754800
H	0.9052040	-1.1073520	-1.1971600	H	0.5182880	-1.2649780	-1.4068640
H	-3.3313960	-2.5660510	-1.3387350	H	-4.3024140	-1.0057990	-1.3515080
H	-3.0618580	-5.0429300	-1.3215450	H	-5.1592330	-3.3342590	-1.3444420
H	0.0778080	-4.6692850	1.5908060	H	-2.0643580	-4.4496630	1.4320200
H	-0.2150510	-2.2184390	1.5955220	H	-1.2363250	-2.1350160	1.4468350
H	0.3469410	2.5341950	-1.3272800	H	1.1807950	2.2053810	-1.5288370
H	1.4721300	4.7264670	-1.0949650	H	3.1429530	3.6837470	-1.2232820
H	0.6756060	6.3635850	0.6051840	H	3.2695580	5.2018670	0.7471930
H	-1.2613110	5.7777300	2.0572570	H	1.4144630	5.2138640	2.4099910
H	-2.3759400	3.5823480	1.8217560	H	-0.5390200	3.7231650	2.1044210
N	2.4481250	-0.5330810	0.2058750	N	1.9491430	-1.5997040	0.1707040
C	3.6115840	-1.2804440	-0.3142240	C	2.8452450	-2.6044260	-0.4499820
H	3.7949870	-2.1298430	0.3506120	H	2.7428160	-3.5306940	0.1198710
H	3.3075370	-1.6804790	-1.2854950	H	2.4547350	-2.7812320	-1.4541570
C	-4.1710690	2.0461650	-0.3660990	C	-3.0249750	3.4536360	-0.4297760
O	-4.1446900	3.2652720	-0.2457410	O	-4.2322340	3.5915990	-0.6103120
O	-5.2954170	1.3856950	-0.7442040	O	-2.1762380	4.5168470	-0.4593430

C	-6.4434630	2.2111410	-0.9735330	C	-2.7748200	5.8040220	-0.6850200
H	-6.2520330	2.9328530	-1.7728910	H	-3.5027910	6.0356670	0.0966790
H	-7.2403650	1.5242590	-1.2615440	H	-1.9500000	6.5160310	-0.6571380
H	-6.7191200	2.7571570	-0.0665780	H	-3.2697950	5.8357740	-1.6589240
Cl	-1.2533930	-6.7479800	0.1257350	Cl	-4.2538830	-5.6916310	0.0385520
C	4.8531130	-0.4242730	-0.4383080	C	4.2888570	-2.1508120	-0.4876050
C	5.9812630	-0.7049730	0.3412180	C	5.2394070	-2.7587620	0.3420830
C	4.8952270	0.6455500	-1.3448370	C	4.6922520	-1.1268330	-1.3580430
C	7.1379730	0.0717120	0.2195430	C	6.5758630	-2.3467410	0.3082900
H	5.9616900	-1.5366740	1.0412940	H	4.9379210	-3.5589940	1.0129330
C	6.0464380	1.4235990	-1.4635730	C	6.0245870	-0.7124540	-1.3895260
H	4.0258410	0.8732050	-1.9566790	H	3.9653620	-0.6533480	-2.0129400
C	7.1708200	1.1374040	-0.6811770	C	6.9692870	-1.3218480	-0.5553590
H	8.0078270	-0.1572210	0.8276380	H	7.3044950	-2.8266000	0.9546300
H	6.0687750	2.2496660	-2.1677910	H	6.3271500	0.0800470	-2.0673220
H	8.0673660	1.7423420	-0.7767590	H	8.0062330	-1.0007730	-0.5830400

Molecule **2i** (PCM for THF)

E = -1855.21349297, **H (0K)** = -1854.776162, **H (298K)** = -1854.746120, **G (298K)** = -1854.841912 au.
Imaginary frequency = 0.

Molecule **2i** (PCM for DMSO)

E =-1855.22168348, **H (0K)** = -1854.784430, **H (298K)** = -1854.754309, **G (298K)** = -1854.852249 au
Imaginary frequency = 0.

C	2.2629020	0.8282720	-1.4282810	C	2.1920840	-0.9501380	1.5331410
C	1.1371340	0.0853530	-1.6289210	C	1.1072290	-0.1406420	1.6941660
N	0.3888280	0.1463180	-0.4625390	N	0.3795880	-0.1871370	0.5132000
C	1.0539630	0.9055680	0.4204640	C	1.0169830	-1.0051680	-0.3384030
C	-0.8781420	-0.4803970	-0.2261210	C	-0.8442570	0.5056170	0.2408880
C	-2.1274970	0.1715830	-0.0366180	C	-2.1230070	-0.0767960	0.0241110
N	-3.0791490	-0.7532870	0.1931760	N	-3.0179850	0.8973360	-0.2311420
C	-2.4737890	-1.9793520	0.1366540	C	-2.3462460	2.0904790	-0.1637880
C	-1.0805680	-1.8690810	-0.1233350	C	-0.9680200	1.9018560	0.1292900
C	-2.4497240	1.6088830	-0.0574450	C	-2.5209800	-1.4959090	0.0454550
C	-3.5362810	2.0915760	0.6994870	C	-3.5862570	-1.9376880	-0.7640920
C	-3.8842640	3.4414310	0.6974510	C	-4.0034770	-3.2681600	-0.7585640
C	-3.1353440	4.3363280	-0.0685850	C	-3.3449090	-4.1830850	0.0643730
C	-2.0591790	3.8959530	-0.8367640	C	-2.2898240	-3.7833360	0.8827350
C	-1.7269470	2.5392380	-0.8294450	C	-1.8886070	-2.4455720	0.8710330
C	-0.0377590	-2.9113290	-0.2680670	C	0.1261320	2.8870310	0.2999650
C	1.0776490	-2.9320070	0.5881070	C	1.2400900	2.8786150	-0.5579170
C	2.0875770	-3.8870960	0.4345350	C	2.2920440	3.7833710	-0.3804070
C	2.0018480	-4.8419750	-0.5821030	C	2.2505810	4.7129850	0.6625990
C	0.8965280	-4.8343180	-1.4409980	C	1.1488800	4.7299400	1.5262710
C	-0.1096060	-3.8793530	-1.2862720	C	0.1005030	3.8254660	1.3474610
H	3.1000440	1.0299680	-2.0761780	H	2.9998050	-1.1882880	2.2055540
H	0.7990460	-0.4744940	-2.4852100	H	0.7833850	0.4535550	2.5325030
H	0.7168770	1.1313110	1.4195600	H	0.6907830	-1.2347600	-1.3401790
H	-4.1113110	1.3898200	1.2937980	H	-4.0886140	-1.2230800	-1.4069280
H	-4.7229660	3.7938860	1.2884500	H	-4.8236860	-3.5898250	-1.3915320
H	-1.4961130	4.5955880	-1.4452340	H	-1.7943610	-4.4982810	1.5307420
H	-0.9116480	2.2077580	-1.4637550	H	-1.0900810	-2.1434380	1.5402960
H	1.1437940	-2.2055780	1.3933480	H	1.2755040	2.1674220	-1.3784880
H	2.9360520	-3.8874580	1.1133640	H	3.1404250	3.7619860	-1.0588220
H	2.7838750	-5.5862030	-0.7025420	H	3.0665000	5.4162840	0.8022840
H	0.8184320	-5.5736860	-2.2333940	H	1.1082010	5.4462410	2.3422870
H	-0.9646760	-3.8810710	-1.9552100	H	-0.7498010	3.8430100	2.0225990
N	2.1895790	1.3372250	-0.1429070	N	2.1134540	-1.4864520	0.2593040
C	3.1830790	2.2331940	0.4979200	C	3.0767650	-2.4396120	-0.3445930
H	3.1738930	3.1765690	-0.0527990	H	3.0968280	-3.3252570	0.2937250
H	2.8143770	2.4261810	1.5070140	H	2.6573590	-2.7273080	-1.3100110
C	-3.2117970	-3.2148320	0.3929280	C	-3.0097700	3.3626130	-0.4341170

O	-2.7156840	-4.3368230	0.4795350	O	-2.4553020	4.4601070	-0.4994840
O	-4.5493800	-3.0174580	0.5428120	O	-4.3511830	3.2363920	-0.6259430
C	-5.3309470	-4.1921560	0.8165390	C	-5.0667080	4.4502900	-0.9152670
H	-5.0039220	-4.6694820	1.7439300	H	-4.6905730	4.9140840	-1.8306370
H	-6.3579780	-3.8400710	0.9139480	H	-6.1063080	4.1494980	-1.0443370
H	-5.2531600	-4.9107480	-0.0036040	H	-4.9787220	5.1595630	-0.0883110
Cl	-3.5623990	6.0511060	-0.0708500	Cl	-3.8604850	-5.8736450	0.0728120
C	4.5729360	1.6356680	0.5235390	C	4.4614770	-1.8468820	-0.4979710
C	5.5519440	2.0889170	-0.3699480	C	5.4758850	-2.1727840	0.4121420
C	4.8977390	0.6286570	1.4447510	C	4.7444130	-0.9682150	-1.5545870
C	6.8372840	1.5378710	-0.3510230	C	6.7534890	-1.6200010	0.2758430
H	5.3126580	2.8776470	-1.0788250	H	5.2718470	-2.8628290	1.2269730
C	6.1795750	0.0765130	1.4628840	C	6.0191260	-0.4149150	-1.6904220
H	4.1495210	0.2781600	2.1510910	H	3.9698080	-0.7186350	-2.2751010
C	7.1515760	0.5296490	0.5634960	C	7.0260520	-0.7388560	-0.7739420
H	7.5889000	1.8981240	-1.0468700	H	7.5320910	-1.8806190	0.9863860
H	6.4219630	-0.7010130	2.1809880	H	6.2281420	0.2622180	-2.5131780
H	8.1494810	0.1019090	0.5807100	H	8.0181640	-0.3108140	-0.8821600
Molecule **3i** (gas phase)				Molecule **3i** (PCM for CH_2Cl_2)			
**E =** -1855.18280095, **H (0K)** = -1854.745409, **H (298K)** = -1854.715230, **G (298K)** = -1854.811743 au.				**E =** -1855.19949626, **H (0K)** = -1854.762151, **H (298K)** = -1854.732011, **G (298K)** = -1854.827828 au			
Imaginary frequency = 0.				Imaginary frequency = 0.			
C	1.9387230	-1.2997890	1.5726190	C	1.8467960	-1.4414340	1.5299870
C	0.9621110	-0.3699240	1.7174100	C	0.9064740	-0.4772290	1.6922020
N	0.2979050	-0.3131640	0.4882530	N	0.2652410	-0.3511010	0.4565380
C	0.8270400	-1.1797040	-0.4438820	C	0.7761890	-1.2076260	-0.4934600
C	-0.8099220	0.5340140	0.2276890	C	-0.7988270	0.5550280	0.2083450
C	-2.1223180	0.1015570	0.0039890	C	-2.1373720	0.1975240	0.0011260
C	-2.0709200	2.3568820	-0.1661090	C	-1.9626100	2.4458720	-0.1619330
C	-0.7616360	1.9530470	0.1257660	C	-0.6740800	1.9685400	0.1126190
C	-2.7286080	-1.2345740	-0.0038860	C	-2.8130610	-1.1061160	-0.0045160
C	-3.8072080	-1.5249830	-0.8582460	C	-3.9072180	-1.3411610	-0.8570010
C	-4.4157990	-2.7796210	-0.8535320	C	-4.5747750	-2.5659510	-0.8524530
C	-3.9373450	-3.7638810	0.0105320	C	-4.1390650	-3.5718230	0.0093930
C	-2.8631480	-3.5071880	0.8637300	C	-3.0532830	-3.3713300	0.8626000
C	-2.2661840	-2.2485040	0.8537740	C	-2.3977210	-2.1412220	0.8528000
C	0.4550900	2.7814230	0.2904510	C	0.5859160	2.7303780	0.2819340
C	1.5844320	2.5365070	-0.5082530	C	1.6774100	2.4887160	-0.5687260
C	2.7478970	3.2908740	-0.3434580	C	2.8789430	3.1818800	-0.3986470
C	2.8037900	4.2964790	0.6250300	C	3.0096710	4.1213680	0.6282730
C	1.6871160	4.5434490	1.4290330	C	1.9304290	4.3640310	1.4846080
C	0.5229750	3.7916820	1.2634330	C	0.7290160	3.6732220	1.3134910
H	2.6910800	-1.6426300	2.2662400	H	2.5645510	-1.8477570	2.2258970
H	0.6886960	0.2486420	2.5579600	H	0.6369630	0.1151410	2.5526090
H	-4.1616710	-0.7764120	-1.5609830	H	-4.2331700	-0.5745640	-1.5533860
H	-5.2425700	-2.9939770	-1.5216800	H	-5.4134310	-2.7363800	-1.5182100
H	-2.5027290	-4.2811700	1.5324000	H	-2.7279130	-4.1603500	1.5314900
H	-1.4392910	-2.0512020	1.5264580	H	-1.5649870	-1.9849570	1.5289580
H	1.5438640	1.7544580	-1.2603200	H	1.5800000	1.7603370	-1.3679420
H	3.6107550	3.0922660	-0.9727200	H	3.7109940	2.9877540	-1.0693050
H	3.7100710	4.8812330	0.7542790	H	3.9441630	4.6586600	0.7616200
H	1.7228530	5.3200540	2.1879960	H	2.0243830	5.0887740	2.2883550
H	-0.3412060	3.9855240	1.8909860	H	-0.1045000	3.8627300	1.9829020
N	1.8325230	-1.7744560	0.2656110	N	1.7455970	-1.8668420	0.2063250
C	2.6975420	-2.8094850	-0.3045750	C	2.5866400	-2.9170140	-0.3792790
H	2.6319980	-3.7091070	0.3175800	H	2.4706320	-3.8304320	0.2124180
H	2.2636730	-3.0429910	-1.2797690	H	2.1737990	-3.1053140	-1.3727730
C	-2.7429390	3.6235530	-0.4494260	C	-2.5499470	3.7576000	-0.4244610
O	-3.9408010	3.6886810	-0.7026240	O	-3.7510690	3.9119440	-0.6359200

O	-1.9298750	4.6953020	-0.4147800	O	-1.6604090	4.7624460	-0.4243350
C	-2.5477410	5.9612210	-0.7137280	C	-2.1838660	6.0829490	-0.6855480
H	-3.3412100	6.1788750	0.0053130	H	-2.9214870	6.3558110	0.0720500
H	-1.7457560	6.6948630	-0.6388220	H	-1.3215080	6.7459080	-0.6375700
H	-2.9690550	5.9512310	-1.7217670	H	-2.6422580	6.1202830	-1.6759250
N	-2.8530530	1.2260780	-0.2418200	N	-2.8081640	1.3607410	-0.2301070
H	-3.8500650	1.2797170	-0.4013530	H	-3.8038020	1.4592280	-0.3784480
Cl	-4.6946720	-5.3501430	0.0226580	Cl	-4.9730440	-5.1240320	0.0201550
C	4.1471730	-2.3836610	-0.4451750	C	4.0543660	-2.5407050	-0.4674660
C	4.4910380	-1.3009360	-1.2686160	C	4.4681130	-1.4669660	-1.2712650
C	5.1642620	-3.0753860	0.2225290	C	5.0205270	-3.2723560	0.2341500
C	5.8247270	-0.9202260	-1.4175110	C	5.8198630	-1.1319720	-1.3671190
H	3.7061200	-0.7593720	-1.7897960	H	3.7272360	-0.8935240	-1.8220290
C	6.5031650	-2.6971710	0.0737380	C	6.3771440	-2.9408510	0.1380720
H	4.9107770	-3.9194390	0.8600880	H	4.7130500	-4.1083020	0.8577580
C	6.8356440	-1.6175120	-0.7459370	C	6.7793720	-1.8687160	-0.6617140
H	6.0782960	-0.0822700	-2.0607810	H	6.1264880	-0.2998450	-1.9944970
H	7.2810180	-3.2449910	0.5980190	H	7.1142730	-3.5186940	0.6881670
H	7.8738250	-1.3207950	-0.8639110	H	7.8311000	-1.6084720	-0.7380870
Molecule **3i** (PCM for THF)<br><br>**E =** -1855.19873591, **H (0K)** = -1854.761395, **H (298K)** = -1854.731252, **G (298K)** = -1854.827113 au.<br>Imaginary frequency = 0.				Molecule **3i** (PCM for DMSO)<br><br>**E =** -1855.20290919, **H (0K)** = -1854.765737, **H (298K)** = -1854.735508, **G (298K)** = -1854.832562 au<br>Imaginary frequency = 0.			
C	1.8573790	-1.4267700	1.5305870	C	1.7886900	-1.5222170	1.5197490
C	0.9123600	-0.4670560	1.6919180	C	0.8692640	-0.5392280	1.6889550
N	0.2686160	-0.3472310	0.4569220	N	0.2471290	-0.3741270	0.4481030
C	0.7823870	-1.2034010	-0.4919730	C	0.7500350	-1.2246350	-0.5109680
C	-0.8007180	0.5524760	0.2082630	C	-0.7899290	0.5644730	0.2046960
C	-2.1368540	0.1867080	0.0002220	C	-2.1405340	0.2498700	0.0046850
C	-1.9758250	2.4361750	-0.1628960	C	-1.8955380	2.4915360	-0.1548350
C	-0.6845460	1.9668070	0.1124880	C	-0.6213740	1.9731260	0.1111640
C	-2.8050420	-1.1207350	-0.0051260	C	-2.8548490	-1.0332330	-0.0029020
C	-3.8985010	-1.3618600	-0.8567610	C	-3.9554470	-1.2340220	-0.8561930
C	-4.5596480	-2.5900860	-0.8514030	C	-4.6575560	-2.4393950	-0.8555800
C	-4.1182050	-3.5934570	0.0104430	C	-4.2501680	-3.4601170	0.0027990
C	-3.0327440	-3.3869450	0.8626400	C	-3.1593090	-3.2937550	0.8569110
C	-2.3835910	-2.1535030	0.8519970	C	-2.4691770	-2.0824560	0.8513840
C	0.5707770	2.7361640	0.2820940	C	0.6625730	2.6962130	0.2737240
C	1.6656820	2.4970730	-0.5649280	C	1.7295730	2.4496990	-0.6061280
C	2.8630950	3.1970720	-0.3943240	C	2.9510200	3.1102670	-0.4459950
C	2.9863300	4.1410400	0.6293650	C	3.1260340	4.0207550	0.6005080
C	1.9036910	4.3812620	1.4820330	C	2.0716370	4.2667900	1.4865970
C	0.7063580	3.6836090	1.3104130	C	0.8503590	3.6088430	1.3251680
H	2.5790260	-1.8268140	2.2261090	H	2.4849600	-1.9619720	2.2171350
H	0.6418310	0.1267010	2.5510540	H	0.5998860	0.0406180	2.5578560
H	-4.2285990	-0.5972480	-1.5534080	H	-4.2611250	-0.4558210	-1.5486660
H	-5.3977750	-2.7652520	-1.5166100	H	-5.5011440	-2.5827450	-1.5214430
H	-2.7027300	-4.1742100	1.5313350	H	-2.8564340	-4.0933490	1.5237390
H	-1.5508840	-1.9927510	1.5271400	H	-1.6337800	-1.9522800	1.5297830
H	1.5740420	1.7651350	-1.3615610	H	1.5981220	1.7437840	-1.4205100
H	3.6978930	3.0047410	-1.0620860	H	3.7636510	2.9133120	-1.1392560
H	3.9176910	4.6836630	0.7630880	H	4.0756150	4.5327940	0.7260270
H	1.9918220	5.1094880	2.2833000	H	2.2001680	4.9690570	2.3052750
H	-0.1298790	3.8712600	1.9769270	H	0.0361150	3.8011680	2.0171340
N	1.7562510	-1.8558760	0.2080940	N	1.6952100	-1.9197250	0.1869570
C	2.6005480	-2.9038990	-0.3763750	C	2.5239840	-2.9747860	-0.4069430
H	2.4881940	-3.8169610	0.2166980	H	2.3851450	-3.8956950	0.1675100
H	2.1874140	-3.0950730	-1.3692040	H	2.1207340	-3.1395180	-1.4087190
C	-2.5721010	3.7437690	-0.4261730	C	-2.4391590	3.8241060	-0.4060540

O	-3.7737780	3.8893540	-0.6400040	O	-3.6363600	4.0219870	-0.6050970
O	-1.6899300	4.7552590	-0.4236900	O	-1.5148090	4.7966500	-0.4109740
C	-2.2226030	6.0714410	-0.6869760	C	-1.9932840	6.1379100	-0.6557960
H	-2.9642080	6.3393830	0.0685350	H	-2.7116360	6.4304400	0.1127200
H	-1.3653380	6.7409010	-0.6374840	H	-1.1073080	6.7690980	-0.6117430
H	-2.6791010	6.1047140	-1.6784000	H	-2.4598110	6.2000970	-1.6410560
N	-2.8145760	1.3458110	-0.2316960	N	-2.7756540	1.4340180	-0.2204780
H	-3.8106750	1.4386750	-0.3804780	H	-3.7690700	1.5628370	-0.3615760
Cl	-4.9440780	-5.1498020	0.0224090	Cl	-5.1280460	-4.9883640	0.0084610
C	4.0668940	-2.5228110	-0.4663830	C	3.9992800	-2.6226230	-0.4704300
C	4.4761430	-1.4484420	-1.2716170	C	4.4378170	-1.5121230	-1.2086180
C	5.0362870	-3.2506730	0.2346470	C	4.9476270	-3.4152780	0.1880670
C	5.8266520	-1.1092700	-1.3695000	C	5.7968600	-1.2010920	-1.2831450
H	3.7325750	-0.8779520	-1.8218010	H	3.7109080	-0.8898840	-1.7238990
C	6.3916930	-2.9149210	0.1365870	C	6.3113990	-3.1080230	0.1127790
H	4.7323020	-4.0871730	0.8592680	H	4.6203590	-4.2782580	0.7625860
C	6.7894180	-1.8422680	-0.6646820	C	6.7386750	-1.9992960	-0.6218790
H	6.1298430	-0.2767720	-1.9980650	H	6.1226520	-0.3396040	-1.8590450
H	7.1313960	-3.4899150	0.6862220	H	7.0344460	-3.7325010	0.6295360
H	7.8402110	-1.5787350	-0.7426780	H	7.7958760	-1.7574950	-0.6810510
Molecule **2j** (gas phase)				Molecule **2j** (PCM for CH_2Cl_2)			
**E =** -1470.79691531, **H (0K)** = -1470.346700, **H (298K)** = -1470.316588, **G (298K)** = -1470.410704 au.				**E =** -1470.83039570, **H (0K)** = -1470.379866, **H (298K)** = -1470.349801, **G (298K)** = -1470.443902 au			
Imaginary frequency = 0.				Imaginary frequency = 0.			
C	1.1224350	-2.6086720	-1.2859690	C	1.3843760	-2.2571280	-1.5745750
C	-0.0269850	-1.8848180	-1.3976160	C	0.2706170	-1.4839920	-1.7085580
N	-0.0054750	-0.9085700	-0.4100920	N	0.1203190	-0.7647730	-0.5311730
C	1.1241060	-1.0506760	0.2912930	C	1.1162230	-1.1011560	0.2975000
C	-0.9772070	0.1101260	-0.1933670	C	-0.9084400	0.1864060	-0.2341620
C	-0.7084500	1.4950130	-0.0070710	C	-0.7340450	1.5802690	-0.0143720
N	-1.8606180	2.1368780	0.2334830	N	-1.9236880	2.1311230	0.2877380
C	-2.8633710	1.2081210	0.1816570	C	-2.8580030	1.1262170	0.2517890
C	-2.3711970	-0.0948620	-0.0829040	C	-2.2717690	-0.1246710	-0.0754030
C	0.5751320	2.2180570	-0.0823020	C	0.5001360	2.3866420	-0.0840560
C	0.8136620	3.3120410	0.7766370	C	0.6631830	3.5058000	0.7601830
C	2.0025860	4.0275270	0.7251760	C	1.8031960	4.2981670	0.7043140
C	3.0041050	3.6741040	-0.1929540	C	2.8328400	3.9955340	-0.2018710
C	2.7892980	2.6013780	-1.0651210	C	2.6951400	2.8932390	-1.0532300
C	1.5805510	1.8927760	-1.0052820	C	1.5364650	2.1084730	-0.9893200
C	-3.0616360	-1.4014290	-0.1657670	C	-2.8790410	-1.4675470	-0.2319870
C	-2.6834270	-2.4616210	0.6790580	C	-2.5297910	-2.5185230	0.6342530
C	-3.2903440	-3.7168880	0.5820550	C	-3.0716820	-3.7974690	0.4703930
C	-4.2922590	-3.9390530	-0.3657120	C	-3.9723200	-4.0508480	-0.5679830
C	-4.6854180	-2.8920130	-1.2064220	C	-4.3270460	-3.0143140	-1.4391610
C	-4.0786530	-1.6395990	-1.1078590	C	-3.7841270	-1.7387670	-1.2737970
H	1.5019630	-3.4289550	-1.8724390	H	1.8606210	-2.9375360	-2.2610020
H	-0.8532290	-1.9624790	-2.0844910	H	-0.4157710	-1.3669850	-2.5306870
H	1.4040560	-0.4490780	1.1393890	H	1.2436850	-0.7270610	1.3000830
H	0.0343540	3.5960430	1.4762350	H	-0.1252430	3.7479150	1.4650870
H	2.1774150	4.8716090	1.3851140	H	1.9167770	5.1568870	1.3592280
H	3.5270940	2.3284900	-1.8113280	H	3.4625520	2.6433830	-1.7763440
H	1.4118470	1.0996290	-1.7292560	H	1.4402900	1.2829830	-1.6880350
H	-1.9259990	-2.2879380	1.4393100	H	-1.8421350	-2.3264090	1.4534370
H	-2.9902900	-4.5148610	1.2564760	H	-2.7938120	-4.5920590	1.1575920
H	-4.7710450	-4.9114640	-0.4403240	H	-4.3958870	-5.0429070	-0.6961360
H	-5.4746720	-3.0489260	-1.9366460	H	-5.0267580	-3.2001530	-2.2494630
H	-4.4021120	-0.8267960	-1.7486180	H	-4.0637790	-0.9389680	-1.9530720
N	1.8456480	-2.0673650	-0.2308880	N	1.9082690	-1.9997130	-0.3140180
C	-4.2637340	1.5694490	0.4274160	C	-4.2589860	1.3603160	0.5894260

O	-5.2186160	0.8072240	0.3292830	O	-5.1280030	0.4928820	0.6778520
O	-4.4180910	2.8700250	0.7877440	O	-4.5432980	2.6721300	0.8202610
C	-5.7682390	3.2835440	1.0260460	C	-5.9040100	2.9685180	1.1745200
H	-6.2159020	2.7063630	1.8403810	H	-6.1867640	2.4556700	2.0976360
H	-5.7042030	4.3385570	1.2959190	H	-5.9370140	4.0487290	1.3175700
H	-6.3801980	3.1553960	0.1283050	H	-6.5878120	2.6717980	0.3749500
C	3.1354550	-2.4888800	0.2278530	C	3.0894910	-2.5908530	0.2537240
C	3.4049760	-3.8536770	0.3633100	C	3.2818190	-3.9702010	0.1437180
C	4.1025440	-1.5272090	0.5360200	C	4.0187970	-1.7753440	0.9042570
C	4.6651130	-4.2574200	0.8074780	C	4.4317260	-4.5386350	0.6952450
H	2.6349040	-4.5868490	0.1457420	H	2.5400760	-4.5908460	-0.3479750
C	5.3523990	-1.9450450	0.9964990	C	5.1566510	-2.3597440	1.4644970
H	3.8872540	-0.4717270	0.4000850	H	3.8660210	-0.7024750	0.9585040
C	5.6378370	-3.3066420	1.1288740	C	5.3666360	-3.7377240	1.3583080
H	4.8789890	-5.3160050	0.9160320	H	4.5877670	-5.6096010	0.6159580
H	6.1060890	-1.2025350	1.2388240	H	5.8829100	-1.7329360	1.9716970
H	6.6136200	-3.6256000	1.4813160	H	6.2558770	-4.1859510	1.7899290
O	4.1418360	4.4401930	-0.1626140	O	3.9182620	4.8313580	-0.1795380
C	5.1535830	4.1864760	-1.1249000	C	4.9857100	4.5834580	-1.0945300
H	4.7764020	4.3147350	-2.1477600	H	4.6416060	4.6494900	-2.1331900
H	5.9386010	4.9199120	-0.9348510	H	5.7251640	5.3622930	-0.9068820
H	5.5699720	3.1756860	-1.0170040	H	5.4391550	3.6005890	-0.9200230
Molecule **2j**<br>(PCM for DMSO)<br><br>**E =** -1470.83697591, **H (0K)** = -1470.386724,<br>**H (298K)** = -1470.356569, **G (298K)** = -1470.451639 au.<br>Imaginary frequency = 0.				Molecule **3j**<br>(gas phase)<br><br>**E =** -1470.80179502, **H (0K)** = -1470.350991,<br>**H (298K)** = -1470.320835, **G (298K)** = -1470.415010 au.<br>Imaginary frequency = 0.			
C	1.1057740	2.2801000	1.6171650	C	1.3411670	2.1029290	1.7076030
C	0.1089400	1.3588910	1.7307290	C	0.3592370	1.1769620	1.8196470
N	0.0529410	0.6585120	0.5333280	N	0.1849590	0.6377370	0.5409330
C	0.9900600	1.1507110	-0.2869840	C	1.0192090	1.1953200	-0.3930750
C	-0.8428870	-0.4128620	0.2137670	C	-0.7670670	-0.3722860	0.2368790
C	-0.4918510	-1.7707130	-0.0170090	C	-0.4727860	-1.7248340	0.0230850
N	-1.6018290	-2.4666190	-0.3232910	C	-2.7039040	-1.4191440	-0.2263000
C	-2.6589560	-1.5904090	-0.2811690	C	-2.1665040	-0.1634060	0.0838610
C	-2.2347000	-0.2762140	0.0557540	C	0.7880890	-2.4740680	0.0396920
C	0.8361690	-2.4128720	0.0382540	C	0.9667930	-3.5961620	-0.7961700
C	1.1340510	-3.4966660	-0.8151570	C	2.1440190	-4.3310800	-0.7762740
C	2.3685680	-4.1337370	-0.7766640	C	3.1919740	-3.9597350	0.0800750
C	3.3599680	-3.7038030	0.1210120	C	3.0392880	-2.8427030	0.9113200
C	3.0885700	-2.6335580	0.9815990	C	1.8473080	-2.1155190	0.8863850
C	1.8371650	-2.0065130	0.9350230	C	-2.8550350	1.1408230	0.2179770
C	-2.9951070	0.9864080	0.2241650	C	-2.4355420	2.2382520	-0.5530880
C	-2.7879390	2.0677250	-0.6502290	C	-3.0610830	3.4797930	-0.4197600
C	-3.4719810	3.2754320	-0.4762320	C	-4.1096330	3.6465760	0.4886360
C	-4.3757670	3.4251150	0.5796080	C	-4.5288810	2.5624150	1.2655520
C	-4.5888500	2.3578350	1.4599290	C	-3.9060060	1.3206770	1.1321270
C	-3.9035280	1.1536750	1.2848470	H	1.8005760	2.7260110	2.4576270
H	1.4879440	3.0002350	2.3217060	H	-0.2191370	0.8558850	2.6718320
H	-0.5499070	1.1262380	2.5507180	H	0.1864130	-3.8822000	-1.4958690
H	1.1653920	0.8258860	-1.2996720	H	2.2800890	-5.1890350	-1.4264040
H	0.3784450	-3.8340860	-1.5170860	H	3.8306650	-2.5316600	1.5826020
H	2.5864430	-4.9645450	-1.4412210	H	1.7455700	-1.2614480	1.5463310
H	3.8254490	-2.2868270	1.6962060	H	-1.6198760	2.1128980	-1.2585600
H	1.6444040	-1.2020050	1.6379370	H	-2.7276560	4.3159430	-1.0279190
H	-2.0960540	1.9563870	-1.4806580	H	-4.5948070	4.6130350	0.5925040
H	-3.2997410	4.0958200	-1.1674810	H	-5.3402170	2.6837970	1.9779910
H	-4.9081740	4.3619940	0.7162730	H	-4.2342390	0.4821710	1.7381480
H	-5.2866440	2.4647250	2.2859450	N	1.7296240	2.0999400	0.3628800
H	-4.0714580	0.3304870	1.9731390	C	-4.0232020	-1.9453190	-0.5646400

N	1.6533610	2.1313570	0.3492060	O	-4.2125730	-3.1293390	-0.8231500
C	-3.9914240	-2.0823660	-0.6197100	O	-4.9992610	-1.0173540	-0.5750620
O	-4.2836500	-3.2556160	-0.8527580	C	-6.3108010	-1.4913800	-0.9313250
O	-4.9289550	-1.0979840	-0.6739930	H	-6.6467520	-2.2554440	-0.2259800
C	-6.2720560	-1.5091530	-0.9820700	H	-6.9539460	-0.6130290	-0.8855330
H	-6.6393120	-2.2218590	-0.2393430	H	-6.3038550	-1.9122000	-1.9396870
H	-6.8674960	-0.5966470	-0.9566000	N	-1.6639710	-2.3239320	-0.2588030
H	-6.3203280	-1.9640370	-1.9746800	H	-1.8219310	-3.3076510	-0.4299080
C	2.7420600	2.8939730	-0.2006730	C	2.7347380	2.9496360	-0.1890950
C	2.7261720	4.2857430	-0.0820910	C	2.9988010	4.1995930	0.3826650
C	3.7905760	2.2280110	-0.8397540	C	3.4555850	2.5273250	-1.3129630
C	3.7871010	5.0212670	-0.6142290	C	3.9928560	5.0172190	-0.1610260
H	1.8942490	4.7863820	0.4017060	H	2.4190640	4.5481300	1.2309810
C	4.8377780	2.9775990	-1.3802600	C	4.4370530	3.3567790	-1.8547510
H	3.7979740	1.1447550	-0.8993510	H	3.2275700	1.5615160	-1.7480090
C	4.8397350	4.3710040	-1.2659870	C	4.7158540	4.6015590	-1.2808300
H	3.7821460	6.1029790	-0.5279930	H	4.1892620	5.9866730	0.2875890
H	5.6563840	2.4682110	-1.8782450	H	4.9913540	3.0235100	-2.7273960
H	5.6592630	4.9483050	-1.6820400	H	5.4846470	5.2408210	-1.7043160
O	4.5452200	-4.3887940	0.0807580	O	4.3096660	-4.7411290	0.0264940
C	5.5898330	-3.9942210	0.9729310	C	5.4151720	-4.4084640	0.8588530
H	5.2817120	-4.1074370	2.0185270	H	5.1451220	-4.4601680	1.9209530
H	6.4254840	-4.6628780	0.7658550	H	6.1823630	-5.1531200	0.6440810
H	5.8967470	-2.9581160	0.7902140	H	5.8025900	-3.4086330	0.6272480

Molecule **3j** (PCM for CH_2Cl_2)	Molecule **3j** (PCM for DMSO)
**E =** -1470.81914618, **H (0K)** = -1470.368673, **H (298K)** = -1470.338466, **G (298K)** = -1470.433042 au<br>Imaginary frequency = 0.	**E** =-1470.82274383, **H (0K)** = -1470.372309, **H (298K)** = -1470.342101, **G (298K)** = -1470.436508 au<br>Imaginary frequency = 0.

C	1.2561790	2.1758340	1.6545240	C	1.2728400	2.1529990	1.6656230
C	0.2775280	1.2457920	1.7627240	C	0.3026770	1.2141060	1.7744980
N	0.1640080	0.6505370	0.5021960	N	0.1731610	0.6380980	0.5066310
C	1.0375210	1.1762240	-0.4153750	C	1.0281210	1.1852080	-0.4151120
C	-0.7721610	-0.3751370	0.2005540	C	-0.7633700	-0.3863990	0.2009880
C	-0.4606570	-1.7254900	-0.0095860	C	-0.4526860	-1.7366000	-0.0120860
C	-2.6952420	-1.4513890	-0.2550790	C	-2.6867140	-1.4600810	-0.2591070
C	-2.1740740	-0.1883570	0.0540800	C	-2.1649190	-0.1981220	0.0544190
C	0.8117650	-2.4562620	0.0202440	C	0.8203350	-2.4667400	0.0163200
C	1.0337140	-3.5501590	-0.8423040	C	1.0438520	-3.5582730	-0.8490610
C	2.2243820	-4.2639630	-0.8064810	C	2.2356050	-4.2706660	-0.8143130
C	3.2392890	-3.9003870	0.0936950	C	3.2497660	-3.9085130	0.0877010
C	3.0405500	-2.8126400	0.9552700	C	3.0492020	-2.8230670	0.9521560
C	1.8365410	-2.1060650	0.9130220	C	1.8443660	-2.1178080	0.9110060
C	-2.8861660	1.1026140	0.2073790	C	-2.8769440	1.0920630	0.2144560
C	-2.5721580	2.1867490	-0.6290900	C	-2.5618890	2.1815610	-0.6149920
C	-3.2192530	3.4158420	-0.4740520	C	-3.2097000	3.4097070	-0.4540730
C	-4.1843130	3.5817450	0.5238030	C	-4.1766050	3.5691980	0.5431900
C	-4.4988490	2.5099860	1.3661080	C	-4.4918850	2.4922640	1.3789330
C	-3.8539680	1.2812640	1.2098720	C	-3.8463700	1.2645220	1.2167020
H	1.6788000	2.8383060	2.3928650	H	1.7034040	2.8059680	2.4077620
H	-0.3290310	0.9495960	2.6040620	H	-0.2866050	0.8983330	2.6209410
H	0.2778080	-3.8335540	-1.5690160	H	0.2881520	-3.8424410	-1.5756020
H	2.3915340	-5.1002400	-1.4776310	H	2.4027780	-5.1054440	-1.4874040
H	3.8034320	-2.5118960	1.6628510	H	3.8105320	-2.5235930	1.6618800
H	1.6960120	-1.2782120	1.5992320	H	1.7028130	-1.2931570	1.6008230
H	-1.8236790	2.0623090	-1.4056690	H	-1.8119110	2.0624380	-1.3909600
H	-2.9688170	4.2417320	-1.1337030	H	-2.9585320	4.2397470	-1.1081880
H	-4.6860640	4.5374030	0.6453670	H	-4.6789990	4.5238360	0.6694810
H	-5.2443150	2.6308080	2.1470330	H	-5.2385240	2.6083200	2.1593430
H	-4.0994710	0.4533580	1.8680030	H	-4.0924220	0.4330120	1.8699580

N	1.7072990	2.1159470	0.3310230	N	1.7030390	2.1182770	0.3343150
C	-4.0179290	-1.9769050	-0.5757450	C	-4.0105240	-1.9817390	-0.5815220
O	-4.2166050	-3.1664780	-0.8172020	O	-4.2131340	-3.1719950	-0.8185150
O	-4.9859970	-1.0456350	-0.5914440	O	-4.9745280	-1.0470690	-0.6061110
C	-6.3163020	-1.5087000	-0.9076470	C	-6.3079160	-1.5062000	-0.9189390
H	-6.6444710	-2.2509470	-0.1768440	H	-6.6405410	-2.2382790	-0.1801210
H	-6.9455880	-0.6212460	-0.8616480	H	-6.9323390	-0.6150210	-0.8821510
H	-6.3394810	-1.9435320	-1.9091160	H	-6.3326180	-1.9503070	-1.9161690
N	-1.6426200	-2.3418840	-0.2907360	N	-1.6347280	-2.3514270	-0.2956530
H	-1.7752280	-3.3301460	-0.4591210	H	-1.7663830	-3.3396190	-0.4661110
C	2.7383590	2.9512410	-0.1987410	C	2.7164930	2.9734670	-0.1990500
C	2.8382680	4.2862220	0.2100340	C	2.8080160	4.3028330	0.2300270
C	3.6486190	2.4284600	-1.1247450	C	3.6169040	2.4768720	-1.1489840
C	3.8565810	5.0939840	-0.3033730	C	3.8072460	5.1310780	-0.2885110
H	2.1169840	4.6985570	0.9080970	H	2.0962320	4.6951300	0.9490180
C	4.6542930	3.2470650	-1.6421030	C	4.6031920	3.3160090	-1.6713630
H	3.5597600	1.3917660	-1.4278480	H	3.5389720	1.4434380	-1.4660520
C	4.7661480	4.5798760	-1.2317920	C	4.7058090	4.6435820	-1.2418890
H	3.9276360	6.1294460	0.0158540	H	3.8721650	6.1616590	0.0471680
H	5.3580110	2.8371610	-2.3605300	H	5.2995400	2.9258380	-2.4076580
H	5.5535490	5.2110270	-1.6324000	H	5.4782870	5.2907770	-1.6457900
O	4.3721070	-4.6588770	0.0533740	O	4.3834970	-4.6651840	0.0467110
C	5.4480040	-4.3330550	0.9375530	C	5.4569170	-4.3425540	0.9372450
H	5.1397470	-4.4234920	1.9851070	H	5.1433840	-4.4363090	1.9826400
H	6.2337820	-5.0578760	0.7254520	H	6.2429580	-5.0671220	0.7258270
H	5.8222180	-3.3209400	0.7479010	H	5.8317630	-3.3301040	0.7520550

Molecule **4a**
(gas phase)

E = -1125.22416241, **H (0K)** = -1124.886059,
H (298K) = -1124.863402, **G (298K)** = -1124.939315 au.
Imaginary frequency = 0.

C	0.4152530	3.4626760	0.7833220
C	-0.1528670	2.2512120	1.0512430
N	0.3519400	1.3434380	0.1332490
C	1.1960340	1.9951450	-0.6751530
C	0.0719780	-0.0559510	0.0732380
C	1.0405840	-1.0928250	0.0370680
N	0.3983350	-2.2718260	-0.0591730
C	-0.9439780	-2.0142920	-0.0569010
C	-1.2205520	-0.6313080	0.0150160
C	2.5099870	-1.0101850	0.1088720
C	3.2989490	-1.9145710	-0.6280570
C	4.6916370	-1.8638980	-0.5703350
C	5.3351450	-0.9061580	0.2213830
C	4.5661190	-0.0098210	0.9689740
C	3.1709750	-0.0640620	0.9174280
C	-2.5079020	0.0948860	-0.0117920
C	-2.7056960	1.1689560	-0.9004760
C	-3.9039730	1.8872400	-0.9124230
C	-4.9351980	1.5436600	-0.0348240
C	-4.7577130	0.4709000	0.8450690
C	-3.5603160	-0.2448400	0.8576080
H	0.2876280	4.4228640	1.2571720
H	-0.8668230	1.9482140	1.7991170
H	1.7464580	1.5420490	-1.4836160
H	2.7987270	-2.6580810	-1.2400100
H	5.2781610	-2.5744830	-1.1464130
H	6.4197080	-0.8690760	0.2664250
H	5.0526660	0.7173890	1.6141030
H	2.5898150	0.6042870	1.5473930

H	-1.9245300	1.4202610	-1.6134840
H	-4.0374410	2.7026910	-1.6186450
H	-5.8712110	2.0949170	-0.0459230
H	-5.5590220	0.1830110	1.5198260
H	-3.4402620	-1.0896020	1.5259100
N	1.2655110	3.2834280	-0.2947810
C	2.1011650	4.3116090	-0.9135930
H	1.4741510	5.1205940	-1.2948660
H	2.8090600	4.7058900	-0.1809810
H	2.6547130	3.8660250	-1.7402130
C	-1.8622230	-3.1656090	-0.1658250
O	-3.0788910	-3.1132180	-0.1680860
O	-1.2148370	-4.3553410	-0.2635970
H	-0.2569210	-4.1503360	-0.2264430

Molecule **4a**
(PCM for CH_2Cl_2)

E = -1125.25935813, **H (0K)** = -1124.921154 ,
H (298K) = -1124.898475, **G (298K)** = -1124.975060 au
Imaginary frequency = 0.

C	0.3681680	3.4456110	0.8987790
C	-0.0179480	2.1801270	1.2291520
N	0.3151420	1.3573240	0.1632050
C	0.8854450	2.1134330	-0.7869730
C	0.1056940	-0.0574970	0.0803920
C	1.1146810	-1.0572380	0.0499060
N	0.5229630	-2.2630950	-0.0599760
C	-0.8328050	-2.0562200	-0.0767170
C	-1.1570180	-0.6817180	0.0052460
C	2.5813810	-0.9159550	0.1138340
C	3.4045720	-1.8486440	-0.5479410
C	4.7950560	-1.7480800	-0.4905540
C	5.4011080	-0.7092110	0.2257670
C	4.5975550	0.2208260	0.8919160
C	3.2049350	0.1173080	0.8405760
C	-2.4758320	-0.0107420	0.0144630
C	-2.7784940	0.9944930	-0.9222130
C	-4.0125120	1.6511040	-0.9000050
C	-4.9697100	1.3148750	0.0614100
C	-4.6828500	0.3148180	0.9974830
C	-3.4495480	-0.3388640	0.9750990
H	0.2920720	4.3730230	1.4431340
H	-0.4902140	1.7899910	2.1155650
H	1.2456150	1.7495590	-1.7359110
H	2.9381880	-2.6517010	-1.1092290
H	5.4076690	-2.4795990	-1.0104340
H	6.4834330	-0.6297880	0.2684160
H	5.0539870	1.0226410	1.4658820
H	2.6033810	0.8287860	1.3973890
H	-2.0505220	1.2481140	-1.6877200
H	-4.2274500	2.4182260	-1.6388580
H	-5.9301450	1.8219500	0.0789920
H	-5.4209140	0.0436280	1.7472290
H	-3.2347480	-1.1142380	1.7033130
N	0.9353580	3.3813720	-0.3624170
C	1.4819310	4.5172880	-1.1136790
H	0.6890980	5.2430230	-1.3001120
H	2.2832870	4.9804590	-0.5365420
H	1.8771070	4.1548670	-2.0615140
C	-1.6866580	-3.2325400	-0.2358310

Molecule **4a**
(PCM for DMSO)

E = -1125.26573227, **H (0K)** = -1124.927537,
H (298K) = -1124.904921, **G (298K)** = -1124.981025 au
Imaginary frequency = 0.

C	0.3431540	3.4437670	0.9211690
C	-0.0052940	2.1687590	1.2554430
N	0.3064570	1.3607100	0.1712610
C	0.8269300	2.1359250	-0.7931030
C	0.1129430	-0.0559960	0.0833230
C	1.1310550	-1.0475550	0.0524200
N	0.5508890	-2.2592780	-0.0555580
C	-0.8078470	-2.0640580	-0.0722760
C	-1.1428940	-0.6916780	0.0087290
C	2.5973580	-0.8941370	0.1096460
C	3.4243070	-1.8260480	-0.5490320
C	4.8144610	-1.7157140	-0.4983550
C	5.4158910	-0.6673830	0.2080890
C	4.6085310	0.2625760	0.8700110
C	3.2163020	0.1493710	0.8253850
C	-2.4688810	-0.0339440	0.0155270
C	-2.7907580	0.9450250	-0.9422200
C	-4.0322000	1.5880400	-0.9238080
C	-4.9770910	1.2639070	0.0540760
C	-4.6701070	0.2908780	1.0122780
C	-3.4292670	-0.3490340	0.9938740
H	0.2682740	4.3654740	1.4751420
H	-0.4364950	1.7631500	2.1557270
H	1.1589660	1.7873040	-1.7579400
H	2.9622090	-2.6356310	-1.1045050
H	5.4299780	-2.4459060	-1.0166330
H	6.4977430	-0.5793350	0.2448380
H	5.0613440	1.0735860	1.4335790
H	2.6130870	0.8640290	1.3757370
H	-2.0714890	1.1911750	-1.7181920
H	-4.2610190	2.3368490	-1.6769670
H	-5.9423790	1.7616510	0.0692190
H	-5.3969900	0.0321870	1.7772280
H	-3.1970860	-1.1007120	1.7416770
N	0.8642550	3.4005440	-0.3604770
C	1.3663690	4.5537900	-1.1175340
H	0.5688100	5.2906650	-1.2195590
H	2.2141490	4.9914680	-0.5887890
H	1.6825100	4.2165060	-2.1034530
C	-1.6496060	-3.2454070	-0.2295590

O	-2.9096750	-3.2578450	-0.3176220
O	-0.9852420	-4.3996390	-0.3019480
H	-0.0376650	-4.1473130	-0.2293840

O	-2.8741370	-3.2849590	-0.3116130
O	-0.9395370	-4.4081290	-0.2951140
H	0.0064370	-4.1480960	-0.2243730

Molecule **5a**
(gas phase)

E = -1125.21935653, **H (0K)** = -1124.881360,
H (298K) = -1124.858569, **G (298K)** = -1124.934708 au.
Imaginary frequency = 0.

C	0.3966680	3.4185490	0.9799120
C	0.1218850	2.1328020	1.3148300
N	0.3399000	1.3796500	0.1580700
C	0.7376690	2.1502100	-0.9135350
C	0.1588730	-0.0261280	0.0883100
C	1.1871550	-0.9775450	0.0507690
C	-0.7981800	-2.0581210	-0.0511060
C	-1.0968050	-0.6905280	0.0239930
C	2.6496660	-0.8486400	0.0749970
C	3.4561210	-1.7812840	-0.6037030
C	4.8471080	-1.6853940	-0.5625940
C	5.4594710	-0.6514170	0.1503380
C	4.6676360	0.2855650	0.8203800
C	3.2769850	0.1902210	0.7861120
C	-2.4186580	-0.0233830	0.0190650
C	-2.7025510	0.9592320	-0.9446270
C	-3.9374980	1.6108840	-0.9496020
C	-4.9027790	1.2982140	0.0112070
C	-4.6258240	0.3277520	0.9784640
C	-3.3934610	-0.3271980	0.9828450
H	0.3546640	4.3220680	1.5693830
H	-0.2077440	1.6948700	2.2441540
H	2.9958130	-2.5683630	-1.1946320
H	5.4510240	-2.4119510	-1.0982890
H	6.5422630	-0.5738710	0.1795080
H	5.1342170	1.0930260	1.3772430
H	2.6752130	0.9176840	1.3188710
H	-1.9527350	1.2057130	-1.6901330
H	-4.1446070	2.3623780	-1.7062780
H	-5.8628020	1.8066460	0.0073860
H	-5.3690900	0.0804850	1.7312620
H	-3.1832480	-1.0803400	1.7355790
N	0.7652140	3.4007220	-0.3644130
C	1.1324850	4.5892340	-1.1212420
H	0.3027400	5.3032690	-1.1456480
H	2.0089080	5.0738920	-0.6785840
H	1.3686240	4.2733500	-2.1371220
C	-1.5621390	-3.2888190	-0.1903230
O	-1.0370810	-4.3945440	-0.2607110
O	-2.9040680	-3.1239100	-0.2448230
N	0.5739770	-2.1902230	-0.0404270
H	1.0228410	-3.0960450	-0.0505260
H	-3.2834920	-4.0114540	-0.3574440

Molecule **5a**
(PCM for CH_2Cl_2)

E = -1125.23601116, **H (0K)** = -1124.898262,
H (298K) = -1124.875401, **G (298K)** = -1124.951980 au
Imaginary frequency = 0.

C	0.3991500	3.4176940	0.9515700
C	0.1110390	2.1347100	1.2869590
N	0.3501740	1.3743380	0.1393120
C	0.7768570	2.1412280	-0.9232050
C	0.1621800	-0.0310440	0.0727720
C	1.1847300	-0.9907260	0.0447880
C	-0.8058330	-2.0585450	-0.0599940
C	-1.0957500	-0.6884520	0.0106300
C	2.6483970	-0.8654880	0.0786500
C	3.4578870	-1.7830270	-0.6177080
C	4.8492520	-1.6843470	-0.5687870
C	5.4570320	-0.6643800	0.1694930
C	4.6614000	0.2557440	0.8597760
C	3.2702740	0.1580350	0.8176310
C	-2.4166040	-0.0169120	0.0189010
C	-2.7426930	0.9080770	-0.9871790
C	-3.9771990	1.5626900	-0.9777980
C	-4.8997290	1.3093560	0.0417160
C	-4.5801810	0.3966210	1.0524830
C	-3.3479070	-0.2601710	1.0418850
H	0.3528990	4.3248840	1.5345990
H	-0.2321520	1.7015700	2.2135020
H	3.0021440	-2.5623080	-1.2218900
H	5.4567280	-2.3985380	-1.1166280
H	6.5394590	-0.5856280	0.2043940
H	5.1243990	1.0495950	1.4384440
H	2.6656490	0.8692400	1.3691930
H	-2.0280360	1.1082010	-1.7795090
H	-4.2166140	2.2694300	-1.7671680
H	-5.8587190	1.8193060	0.0500070
H	-5.2891940	0.1968500	1.8508550
H	-3.1037630	-0.9659810	1.8298420
N	0.7984930	3.3938190	-0.3829480
C	1.1888430	4.5868110	-1.1272390
H	0.3592000	5.2982580	-1.1715190
H	2.0487160	5.0686300	-0.6530060
H	1.4574350	4.2806900	-2.1377050
C	-1.5953590	-3.2723500	-0.1940700
O	-1.0963630	-4.3950770	-0.2351940
O	-2.9269610	-3.0720840	-0.2834940
N	0.5656920	-2.1997500	-0.0439810
H	1.0189230	-3.1038760	-0.0500590
H	-3.3457130	-3.9450400	-0.3781780

Molecule **5a**
(PCM for DMSO)

E = -1125.23936276, **H (0K)** = -1124.901685,
H (298K) = -1124.878805, **G (298K)** = -1124.955450 au.
Imaginary frequency = 0.

Molecule **6a**
(gas phase)

E = -1125.20563950, **H (0K)** = -1124.867603,
H (298K) = -1124.844876, **G (298K)** = -1124.921071 au.
Imaginary frequency = 0.

C	0.3930180	3.4181710	0.9429260	C	0.3593970	3.4429990	0.8712780
C	0.1005860	2.1363240	1.2793590	C	-0.1489940	2.2047190	1.1339350
N	0.3515220	1.3720670	0.1369380	N	0.3411180	1.3444230	0.1653990
C	0.7904560	2.1359860	-0.9223710	C	1.1161080	2.0477360	-0.6684060
C	0.1614670	-0.0331080	0.0714770	C	0.0781810	-0.0574640	0.0720650
C	1.1827670	-0.9940850	0.0458460	C	1.0738430	-1.0406170	0.0352440
C	-0.8082740	-2.0598000	-0.0617350	C	-0.9708390	-2.0334160	-0.0665700
C	-1.0969140	-0.6890600	0.0078620	C	-1.2202420	-0.6576710	0.0007470
C	2.6467690	-0.8685020	0.0815940	C	2.5387970	-0.9714980	0.0867860
C	3.4561830	-1.7708910	-0.6345500	C	3.3180980	-1.8372700	-0.7059210
C	4.8477020	-1.6708830	-0.5857400	C	4.7116170	-1.7914610	-0.6556440
C	5.4550010	-0.6650350	0.1722740	C	5.3602920	-0.8739930	0.1762560
C	4.6591850	0.2395250	0.8831170	C	4.5993300	-0.0112060	0.9710560
C	3.2678810	0.1405440	0.8410520	C	3.2050790	-0.0631280	0.9336360
C	-2.4170050	-0.0152150	0.0164510	C	-2.5119580	0.0615030	-0.0361200
C	-2.7500290	0.8974250	-0.9986570	C	-2.6651750	1.2214210	-0.8196070
C	-3.9824540	1.5563830	-0.9865360	C	-3.8714530	1.9260820	-0.8413340
C	-4.8965810	1.3186950	0.0444880	C	-4.9544570	1.4795530	-0.0804980
C	-4.5707170	0.4172470	1.0635200	C	-4.8213930	0.3170920	0.6857610
C	-3.3403230	-0.2433380	1.0503910	C	-3.6173870	-0.3877840	0.7092840
H	0.3428460	4.3272720	1.5225120	H	0.2206170	4.3851630	1.3770460
H	-0.2502890	1.7056270	2.2040970	H	-0.8165890	1.8551590	1.9044140
H	3.0003980	-2.5395540	-1.2520020	H	1.6311200	1.6398780	-1.5233230
H	5.4554600	-2.3733840	-1.1481120	H	2.8257630	-2.5401310	-1.3717850
H	6.5373410	-0.5856940	0.2076780	H	5.2914390	-2.4717870	-1.2725910
H	5.1220430	1.0210960	1.4782920	H	6.4448940	-0.8402130	0.2143510
H	2.6631600	0.8379520	1.4100110	H	5.0927330	0.6861110	1.6426600
H	-2.0431550	1.0849110	-1.8010670	H	2.6280650	0.5738940	1.5980400
H	-4.2267690	2.2540320	-1.7824160	H	-1.8468820	1.5541640	-1.4535950
H	-5.8537360	1.8319120	0.0550190	H	-3.9700840	2.8090760	-1.4675570
H	-5.2729310	0.2299110	1.8707810	H	-5.8970880	2.0193310	-0.0999850
H	-3.0909920	-0.9389300	1.8457630	H	-5.6655450	-0.0550890	1.2592350
N	0.8072230	3.3902030	-0.3868150	H	-3.5358050	-1.3185110	1.2575330
C	1.2100400	4.5817000	-1.1279570	N	1.1554590	3.3253340	-0.2565820
H	0.3837180	5.2960840	-1.1806270	C	1.9062860	4.4073560	-0.8955920
H	2.0660730	5.0602010	-0.6440580	H	1.2195540	5.1913230	-1.2216230
H	1.4885250	4.2752720	-2.1356890	H	2.6325170	4.8196770	-0.1917980
C	-1.6007840	-3.2720240	-0.1926920	H	2.4337260	4.0079750	-1.7618720
O	-1.1044130	-4.3964640	-0.2296470	C	-1.7947210	-3.3379160	-0.1317820
O	-2.9311710	-3.0685640	-0.2844560	O	-3.0340320	-3.2254130	-0.0566790
N	0.5630800	-2.2023280	-0.0441930	O	-1.0525650	-4.3517730	-0.2364750
H	1.0170180	-3.1063080	-0.0473870	N	0.3827060	-2.2111700	-0.0664980
H	-3.3546700	-3.9401250	-0.3722110	H	0.7069100	-3.1788510	-0.0875840

Molecule **6a**
(PCM for CH_2Cl_2)

E = -1125.26280907, **H (0K)** = -1124.923913,
H (298K) = -1124.901251, **G (298K)** = -1124.977474
au
Imaginary frequency = 0.

C	0.3005120	3.4250800	0.9066070
C	-0.0339360	2.1476780	1.2440030
N	0.3053670	1.3397760	0.1682370
C	0.8247540	2.1168370	-0.7958420
C	0.1225700	-0.0752110	0.0830110
C	1.1528710	-1.0176290	0.0528440
C	-0.8475730	-2.0929150	-0.0870390
C	-1.1431810	-0.7320150	-0.0011960
C	2.6151400	-0.8839380	0.1075850
C	3.4386300	-1.7499350	-0.6376660
C	4.8286010	-1.6376260	-0.5776720

Molecule **6a**
(PCM for DMSO)

E = -1125.27285057, **H (0K)** = -1124.933961,
H (298K) = -1124.911301, **G (298K)** = -1124.987399
au
Imaginary frequency = 0.

C	0.2937990	3.4219240	0.9102160
C	-0.0272530	2.1427360	1.2526630
N	0.3003650	1.3373680	0.1704770
C	0.8005140	2.1187460	-0.8013800
C	0.1253500	-0.0779970	0.0875750
C	1.1587100	-1.0169680	0.0577370
C	-0.8370270	-2.1003400	-0.0806100
C	-1.1365870	-0.7404190	0.0044780
C	2.6208730	-0.8764270	0.1078050
C	3.4458190	-1.7388200	-0.6399240
C	4.8354380	-1.6181920	-0.5858850

C	5.4238110	-0.6524640	0.2166750	C	5.4276870	-0.6284160	0.2051490
C	4.6158580	0.2147550	0.9589960	C	4.6177130	0.2353910	0.9493840
C	3.2257860	0.0980840	0.9111110	C	3.2280880	0.1107200	0.9072990
C	-2.4626510	-0.0585120	0.0134060	C	-2.4583370	-0.0693510	0.0165020
C	-2.7949770	0.8858280	-0.9728490	C	-2.8167010	0.8187920	-1.0117910
C	-4.0276470	1.5454380	-0.9470530	C	-4.0506680	1.4766250	-0.9915480
C	-4.9475840	1.2726300	0.0689950	C	-4.9443100	1.2598070	0.0611520
C	-4.6267040	0.3348030	1.0569580	C	-4.5954530	0.3809900	1.0930840
C	-3.3953170	-0.3223850	1.0305430	C	-3.3627680	-0.2748050	1.0718760
H	0.2042750	4.3482860	1.4547570	H	0.2016240	4.3443910	1.4601970
H	-0.4736420	1.7406310	2.1395580	H	-0.4479410	1.7330510	2.1560460
H	1.1725050	1.7666060	-1.7547110	H	1.1359120	1.7721560	-1.7659040
H	2.9908500	-2.5019960	-1.2806990	H	3.0002480	-2.4932370	-1.2817360
H	5.4459730	-2.3152650	-1.1598980	H	5.4545210	-2.2917310	-1.1710360
H	6.5051170	-0.5639660	0.2597750	H	6.5085590	-0.5323160	0.2425570
H	5.0679370	0.9746640	1.5896890	H	5.0674680	1.0007700	1.5749180
H	2.6141300	0.7542760	1.5218590	H	2.6153620	0.7672350	1.5163690
H	-2.0945340	1.0886280	-1.7783760	H	-2.1341630	0.9823190	-1.8411250
H	-4.2703190	2.2640580	-1.7248770	H	-4.3126440	2.1537220	-1.7996140
H	-5.9071960	1.7811440	0.0890700	H	-5.9033530	1.7693870	0.0781160
H	-5.3375160	0.1128090	1.8479180	H	-5.2830690	0.2072600	1.9160060
H	-3.1532410	-1.0543600	1.7941470	H	-3.0959150	-0.9551540	1.8744800
N	0.8379540	3.3831550	-0.3689420	N	0.8115070	3.3837480	-0.3734610
C	1.3265930	4.5418560	-1.1272770	C	1.2850550	4.5460330	-1.1363810
H	0.5138570	5.2589750	-1.2495580	H	0.4691380	5.2622090	-1.2396370
H	2.1545320	5.0035210	-0.5875600	H	2.1221340	5.0055200	-0.6092020
H	1.6679970	4.2046620	-2.1048360	H	1.6082830	4.2124540	-2.1211570
C	-1.6707910	-3.3557490	-0.2375550	C	-1.6605990	-3.3572430	-0.2287510
O	-2.9154110	-3.2321080	-0.3632220	O	-2.9072100	-3.2342630	-0.3608180
O	-0.9881530	-4.4249230	-0.2293450	O	-0.9892570	-4.4346390	-0.2150130
N	0.5193280	-2.2216460	-0.0616170	N	0.5316980	-2.2244830	-0.0546720
H	0.9406570	-3.1430660	-0.0790000	H	0.9645170	-3.1399610	-0.0709480
Molecule **4b** (gas phase)				Molecule **4b** (PCM for CH_2Cl_2)			
**E =** -1584.81898061, **H (0K)** = -1584.490563, **H (298K)** = -1584.466616, **G (298K)** = -1584.546109 au. Imaginary frequency = 0.				**E =** -1584.85441545, **H (0K)** = -1584.525813, **H (298K)** = -1584.501964, **G (298K)** = -1584.581308 au Imaginary frequency = 0.			
C	-0.4078490	3.5074530	0.7898480	C	-0.3779290	3.4724400	0.9184650
C	-0.8651780	2.2526070	1.0700230	C	-0.6832350	2.1843720	1.2454330
N	-0.3246510	1.3933290	0.1262690	N	-0.3406200	1.3927280	0.1590520
C	0.4314650	2.1153160	-0.7092680	C	0.1543360	2.1895770	-0.8004510
C	-0.5021700	-0.0231790	0.0619940	C	-0.4774010	-0.0305900	0.0681710
C	0.5360950	-0.9894030	0.0078430	C	0.5811830	-0.9769350	0.0224670
N	-0.0228910	-2.2106580	-0.0879580	N	0.0519970	-2.2122910	-0.0795040
C	-1.3790940	-2.0477430	-0.0681690	C	-1.3117760	-2.0761660	-0.0760790
C	-1.7509760	-0.6876700	0.0153930	C	-1.7065350	-0.7199060	0.0092700
C	1.9954010	-0.8078820	0.0608530	C	2.0386270	-0.7654850	0.0608020
C	2.8361240	-1.6738420	-0.6649320	C	2.8933560	-1.6717710	-0.5970330
C	4.2227850	-1.5358970	-0.6340190	C	4.2784050	-1.5152950	-0.5724190
C	4.7933120	-0.5154030	0.1291460	C	4.8281900	-0.4327980	0.1160770
C	3.9925740	0.3489120	0.8747300	C	4.0147900	0.4804350	0.7847980
C	2.6050970	0.1943180	0.8403060	C	2.6293700	0.3065870	0.7565600
C	-3.0857960	-0.0518880	0.0086170	C	-3.0580200	-0.1166800	0.0247850
C	-3.3707890	1.0038070	-0.8781460	C	-3.4130230	0.8716630	-0.9117040
C	-4.6156810	1.6379050	-0.8718890	C	-4.6772910	1.4675260	-0.8852930
C	-5.6063780	1.2264930	0.0230110	C	-5.6135810	1.0860410	0.0798180
C	-5.3415820	0.1707710	0.9015110	C	-5.2751800	0.1012860	1.0147930
C	-4.0979330	-0.4613090	0.8954100	C	-4.0118950	-0.4920780	0.9879960
H	-0.5938030	4.4519030	1.2758770	H	-0.4865940	4.3882760	1.4766920

H	-1.5237710	1.8891360	1.8415090	H	-1.1042630	1.7590980	2.1415000
H	0.9805200	1.7133990	-1.5453270	H	0.5019300	1.8561390	-1.7652180
H	2.3834940	-2.4640720	-1.2544710	H	2.4593950	-2.5073350	-1.1354770
H	4.8563380	-2.2106890	-1.1998200	H	4.9207580	-2.2216680	-1.0873020
H	4.4484110	1.1152520	1.4930040	H	4.4528790	1.3074800	1.3330710
H	1.9950960	0.8375410	1.4682490	H	2.0123970	1.0036510	1.3136390
H	-2.6204010	1.3060420	-1.6043860	H	-2.7016920	1.1590920	-1.6808960
H	-4.8164030	2.4399810	-1.5774330	H	-4.9317170	2.2227290	-1.6238540
H	-6.5782420	1.7117900	0.0260660	H	-6.5973600	1.5459850	0.1007490
H	-6.1104130	-0.1697210	1.5893770	H	-5.9965220	-0.2055670	1.7670370
H	-3.9095800	-1.2941790	1.5629490	H	-3.7579030	-1.2562780	1.7153640
N	0.4108720	3.4029090	-0.3219850	N	0.1481440	3.4535360	-0.3618500
C	-2.2187420	-3.2587090	-0.1710950	C	-2.1057520	-3.2971210	-0.2136140
O	-3.4358010	-3.2869530	-0.1589300	O	-3.3269430	-3.3856790	-0.2650280
O	-1.4948090	-4.4021450	-0.2811210	O	-1.3463970	-4.4258850	-0.2934090
H	-0.5523000	-4.1355740	-0.2539020	H	-0.4118590	-4.1258460	-0.2404920
Cl	6.5441410	-0.3242800	0.1627250	Cl	6.5811480	-0.2205300	0.1459490
C	1.1353820	4.4997800	-0.9639950	C	0.6163990	4.6260220	-1.1104760
H	0.4301630	5.2627490	-1.3004970	H	-0.2037320	5.3375120	-1.2155650
H	1.8468500	4.9375600	-0.2601900	H	1.4463310	5.0894190	-0.5750320
H	1.6788140	4.1087500	-1.8242520	H	0.9502730	4.3042780	-2.0959170
Molecule **4b** (PCM for THF)				Molecule **4b** (PCM for DMSO)			
**E =** -1584.85296827, **H (0K)** = -1584.524369, **H (298K)** = -1584.500514, **G (298K)** = -1584.579932 au.				**E =** -1584.86076315, **H (0K)** = -1584.532266, **H (298K)** = -1584.508379, **G (298K)** = -1584.587894 au			
Imaginary frequency = 0.				Imaginary frequency = 0.			
C	-0.3774920	3.4724580	0.9187000	C	-0.3800240	3.4739790	0.9143630
C	-0.6855770	2.1847410	1.2446660	C	-0.6717460	2.1846750	1.2478320
N	-0.3403600	1.3925210	0.1596860	N	-0.3413340	1.3941490	0.1560700
C	0.1588510	2.1884640	-0.7981170	C	0.1335380	2.1938710	-0.8122280
C	-0.4780360	-0.0307640	0.0686200	C	-0.4744230	-0.0292830	0.0666900
C	0.5802560	-0.9771900	0.0225560	C	0.5855430	-0.9750970	0.0211990
N	0.0504720	-2.2122980	-0.0795390	N	0.0590830	-2.2116120	-0.0811510
C	-1.3129640	-2.0758930	-0.0757370	C	-1.3060820	-2.0768610	-0.0792260
C	-1.7076920	-0.7197000	0.0096690	C	-1.7012960	-0.7204030	0.0074460
C	2.0376560	-0.7662720	0.0609650	C	2.0431320	-0.7613920	0.0598580
C	2.8919910	-1.6719530	-0.5982400	C	2.8998990	-1.6711310	-0.5905480
C	4.2770420	-1.5158040	-0.5739330	C	4.2849230	-1.5136060	-0.5634810
C	4.8275240	-0.4343260	0.1156450	C	4.8314200	-0.4261880	0.1198110
C	4.0145280	0.4780300	0.7860590	C	4.0159090	0.4917010	0.7794730
C	2.6290480	0.3045590	0.7581050	C	2.6307340	0.3165320	0.7490140
C	-3.0586960	-0.1155610	0.0239380	C	-3.0549620	-0.1217950	0.0303670
C	-3.4094300	0.8784140	-0.9082700	C	-3.4270020	0.8498750	-0.9166580
C	-4.6729250	1.4758230	-0.8830150	C	-4.6949770	1.4380990	-0.8829180
C	-5.6130490	1.0902430	0.0766770	C	-5.6166560	1.0655260	0.0998170
C	-5.2792390	0.0995280	1.0069250	C	-5.2597980	0.0990360	1.0472580
C	-4.0167330	-0.4954210	0.9812620	C	-3.9926790	-0.4863260	1.0135050
H	-0.4871590	4.3884750	1.4764580	H	-0.4846610	4.3897930	1.4732870
H	-1.1106150	1.7599710	2.1390860	H	-1.0741640	1.7583430	2.1518660
H	0.5094800	1.8541910	-1.7615040	H	0.4672060	1.8628840	-1.7827030
H	2.4573760	-2.5068570	-1.1371790	H	2.4690090	-2.5098360	-1.1265850
H	4.9191010	-2.2217760	-1.0897420	H	4.9288310	-2.2223200	-1.0731420
H	4.4532620	1.3038010	1.3357670	H	4.4507830	1.3248980	1.3207990
H	2.0123200	1.0004350	1.3170410	H	2.0125650	1.0195910	1.2965810
H	-2.6954210	1.1684050	-1.6740360	H	-2.7257900	1.1335780	-1.6962900
H	-4.9240400	2.2350360	-1.6186260	H	-4.9622970	2.1823060	-1.6278900
H	-6.5964170	1.5511250	0.0965930	H	-6.6024080	1.5208180	0.1269610
H	-6.0038570	-0.2112550	1.7543860	H	-5.9682860	-0.1971790	1.8158300
H	-3.7668460	-1.2649310	1.7043620	H	-3.7221810	-1.2333210	1.7530010
N	0.1529790	3.4527610	-0.3598120	N	0.1253600	3.4569700	-0.3741720

C	-2.1076520	-3.2973090	-0.2121980	C	-2.0966480	-3.2956590	-0.2237870
O	-3.3285810	-3.3847820	-0.2619540	O	-3.3190070	-3.3889650	-0.2847780
O	-1.3483400	-4.4258660	-0.2923800	O	-1.3371170	-4.4251490	-0.3021820
H	-0.4138380	-4.1259000	-0.2398310	H	-0.4023970	-4.1250910	-0.2458080
Cl	6.5803950	-0.2224320	0.1450290	Cl	6.5847240	-0.2128910	0.1532500
C	0.6247450	4.6243910	-1.1074090	C	0.5774720	4.6316920	-1.1299330
H	-0.1942880	5.3367480	-1.2156930	H	-0.2465930	5.3404520	-1.2192230
H	1.4534860	5.0874610	-0.5697860	H	1.4150570	5.0959660	-0.6075160
H	0.9615410	4.3019520	-2.0916720	H	0.8946040	4.3111510	-2.1210850
Molecule **5b** (gas phase)				Molecule **5b** (PCM for CH_2Cl_2)			
**E =** -1584.81341324, **H (0K)** = -1584.485137, **H (298K)** = -1584.461084, **G (298K)** = -1584.540602 au. Imaginary frequency = 0.				**E =** -1584.83016009, **H (0K)** = -1584.502232, **H (298K)** = -1584.478062, **G (298K)** = -1584.558235 au Imaginary frequency = 0.			
C	-0.3322220	3.4517630	0.9565340	C	-0.3245410	3.4484760	0.9325420
C	-0.5685510	2.1589410	1.2937110	C	-0.5642440	2.1561110	1.2699280
N	-0.3032210	1.4085050	0.1447850	N	-0.2925520	1.4034630	0.1241180
C	0.0878310	2.1875470	-0.9235840	C	0.1077500	2.1844200	-0.9388270
C	-0.4341700	-0.0024650	0.0770530	C	-0.4285480	-0.0075780	0.0581040
C	0.6284290	-0.9140940	0.0274560	C	0.6292720	-0.9268540	0.0172160
C	-1.3141870	-2.0692120	-0.0575990	C	-1.3177110	-2.0711970	-0.0678780
C	-1.6643100	-0.7142390	0.0233560	C	-1.6604580	-0.7135740	0.0080280
C	2.0835570	-0.7266070	0.0391200	C	2.0856540	-0.7409770	0.0362870
C	2.9238430	-1.6216440	-0.6476000	C	2.9278560	-1.6167120	-0.6730900
C	4.3100450	-1.4753610	-0.6231120	C	4.3140470	-1.4656160	-0.6430260
C	4.8695290	-0.4153370	0.0897640	C	4.8660370	-0.4214690	0.0984640
C	4.0604760	0.4940460	0.7723250	C	4.0567360	0.4678360	0.8063690
C	2.6766530	0.3350910	0.7452550	C	2.6729200	0.3036150	0.7729270
C	-3.0103520	-0.0973650	0.0316140	C	-3.0058530	-0.0936230	0.0322520
C	-3.3384280	0.8777470	-0.9255870	C	-3.3749380	0.8283890	-0.9615960
C	-4.5962310	1.4838160	-0.9171580	C	-4.6328870	1.4360820	-0.9350260
C	-5.5411370	1.1320900	0.0504700	C	-5.5367020	1.1373800	0.0890100
C	-5.2207140	0.1681820	1.0107420	C	-5.1747870	0.2265670	1.0870710
C	-3.9652790	-0.4412860	1.0018430	C	-3.9188770	-0.3831520	1.0596740
H	-0.4170930	4.3560940	1.5401010	H	-0.4074680	4.3542840	1.5136270
H	-0.9015630	1.7144890	2.2186960	H	-0.8939540	1.7117780	2.1960260
H	2.4964510	-2.4272480	-1.2375850	H	2.5058070	-2.4123480	-1.2794120
H	4.9470220	-2.1679790	-1.1619990	H	4.9520820	-2.1438950	-1.1983950
H	4.5084100	1.3135240	1.3235070	H	4.4989870	1.2733390	1.3819740
H	2.0547770	1.0390570	1.2860810	H	2.0497010	0.9880110	1.3368900
H	-2.6054610	1.1537450	-1.6774590	H	-2.6756030	1.0628290	-1.7581810
H	-4.8371460	2.2302580	-1.6687470	H	-4.9053540	2.1414440	-1.7148310
H	-6.5190540	1.6050500	0.0568960	H	-6.5140910	1.6106620	0.1102880
H	-5.9480540	-0.1095690	1.7683950	H	-5.8692410	-0.0082510	1.8886690
H	-3.7216520	-1.1894530	1.7494290	H	-3.6420230	-1.0872450	1.8382040
N	0.0620720	3.4404620	-0.3805160	N	0.0797250	3.4375120	-0.4006340
C	-2.0309840	-3.3295040	-0.1914540	C	-2.0600790	-3.3162740	-0.1946510
O	-1.4619520	-4.4124140	-0.2699500	O	-1.5154620	-4.4169090	-0.2442780
O	-3.3781820	-3.2177210	-0.2305730	O	-3.3990980	-3.1697520	-0.2661640
N	0.0617570	-2.1496360	-0.0615070	N	0.0579100	-2.1595260	-0.0677930
H	0.5425880	-3.0389780	-0.0741390	H	0.5441530	-3.0464910	-0.0759980
H	-3.7245930	-4.1190370	-0.3405770	H	-3.7842250	-4.0585520	-0.3566700
Cl	6.6153860	-0.2197050	0.1250560	Cl	6.6155430	-0.2199820	0.1400750
C	0.4037150	4.6376680	-1.1362720	C	0.4274030	4.6432750	-1.1457480
H	-0.4492390	5.3226820	-1.1806630	H	-0.4293430	5.3213150	-1.1971750
H	1.2539790	5.1538320	-0.6787110	H	1.2644020	5.1596510	-0.6671620
H	0.6708050	4.3256240	-2.1456010	H	0.7145320	4.3455400	-2.1535880
Molecule **5b** (PCM for THF)				Molecule **5b** (PCM for DMSO)			

E = -1584.82942390, **H (0K)** = -1584.501489,
H (298K) = -1584.477318, **G (298K)** = -1584.557502 au.
Imaginary frequency = 0.

C	-0.3255470	3.4487810	0.9333800
C	-0.5648010	2.1563230	1.2707070
N	-0.2929710	1.4038380	0.1247960
C	0.1070360	2.1848260	-0.9383420
C	-0.4286880	-0.0072060	0.0587690
C	0.6293570	-0.9260990	0.0174110
C	-1.3173440	-2.0710600	-0.0678260
C	-1.6604750	-0.7135890	0.0086300
C	2.0856780	-0.7401580	0.0361540
C	2.9278150	-1.6167630	-0.6721470
C	4.3140140	-1.4659990	-0.6420700
C	4.8662490	-0.4212370	0.0983350
C	4.0569290	0.4690700	0.8049440
C	2.6731180	0.3051730	0.7715160
C	-3.0059620	-0.0939500	0.0323900
C	-3.3728310	0.8316910	-0.9588710
C	-4.6308670	1.4390620	-0.9328480
C	-5.5369520	1.1365180	0.0879930
C	-5.1772380	0.2220900	1.0834630
C	-3.9212650	-0.3874160	1.0565970
H	-0.4091710	4.3545080	1.5145150
H	-0.8950510	1.7119420	2.1965970
H	2.5054580	-2.4125920	-1.2780340
H	4.9520830	-2.1448310	-1.1967360
H	4.4994400	1.2752450	1.3794170
H	2.0498090	0.9906330	1.3340960
H	-2.6715520	1.0691290	-1.7528390
H	-4.9016690	2.1472290	-1.7106980
H	-6.5144660	1.6095940	0.1087690
H	-5.8735440	-0.0158420	1.8825510
H	-3.6462000	-1.0945850	1.8329680
N	0.0786730	3.4379100	-0.3998760
C	-2.0586430	-3.3167520	-0.1950070
O	-1.5129810	-4.4166840	-0.2460000
O	-3.3980300	-3.1716620	-0.2648160
N	0.0583110	-2.1589570	-0.0678960
H	0.5444600	-3.0459290	-0.0763950
H	-3.7815580	-4.0609560	-0.3566670
Cl	6.6156470	-0.2201840	0.1400780
C	0.4257140	4.6434160	-1.1454580
H	-0.4313230	5.3211800	-1.1972270
H	1.2628080	5.1603660	-0.6674870
H	0.7126850	4.3449200	-2.1531220

E = -1584.83347935, **H (0K)** = -1584.505574,
H (298K) = -1584.481410, **G (298K)** = -1584.561552 au
Imaginary frequency = 0.

C	-0.3199330	3.4469950	0.9309290
C	-0.5624770	2.1551100	1.2685710
N	-0.2905430	1.4013820	0.1236180
C	0.1132660	2.1817950	-0.9379780
C	-0.4284550	-0.0095300	0.0577120
C	0.6281920	-0.9304210	0.0187040
C	-1.3198110	-2.0720010	-0.0675240
C	-1.6609600	-0.7138790	0.0070360
C	2.0848580	-0.7446210	0.0379280
C	2.9260450	-1.6127230	-0.6821180
C	4.3122600	-1.4602880	-0.6535840
C	4.8643110	-0.4228130	0.0972640
C	4.0561930	0.4582700	0.8168110
C	2.6723290	0.2927300	0.7847700
C	-3.0058680	-0.0917460	0.0303700
C	-3.3836180	0.8098080	-0.9789650
C	-4.6406190	1.4201160	-0.9531690
C	-5.5348560	1.1441310	0.0857370
C	-5.1643710	0.2536210	1.0991020
C	-3.9093390	-0.3584170	1.0724780
H	-0.4002550	4.3533340	1.5114440
H	-0.8916100	1.7112690	2.1950740
H	2.5036610	-2.4041080	-1.2936370
H	4.9494800	-2.1330290	-1.2165410
H	4.4985310	1.2575920	1.4008630
H	2.0499400	0.9693330	1.3590170
H	-2.6922870	1.0267000	-1.7875000
H	-4.9196350	2.1096880	-1.7446630
H	-6.5112120	1.6194360	0.1067760
H	-5.8510780	0.0368900	1.9123110
H	-3.6254940	-1.0458410	1.8633370
N	0.0865360	3.4351870	-0.4013880
C	-2.0665370	-3.3148150	-0.1899390
O	-1.5263080	-4.4181450	-0.2346750
O	-3.4044340	-3.1628770	-0.2639320
N	0.0556450	-2.1622760	-0.0664490
H	0.5422940	-3.0492160	-0.0716720
H	-3.7956770	-4.0499400	-0.3469370
Cl	6.6142550	-0.2196040	0.1366470
C	0.4390480	4.6415240	-1.1444070
H	-0.4148350	5.3230350	-1.1928540
H	1.2780160	5.1529850	-0.6644550
H	0.7243580	4.3461860	-2.1535250

Molecule **6b**
(gas phase)

E = -1584.79915552, **H (0K)** = -1584.470837,
H (298K) = -1584.446867, **G (298K)** = -1584.526324 au.
Imaginary frequency = 0.

C	-0.4406540	3.4813070	0.8659760
C	-0.8488220	2.2083510	1.1378290
N	-0.3279770	1.3892150	0.1498780
C	0.3662700	2.1502220	-0.7045990
C	-0.4992560	-0.0271640	0.0539820
C	0.5572960	-0.9447840	0.0028880
C	-1.4200500	-2.0658400	-0.0819730
C	-1.7565530	-0.7088150	-0.0054410

Molecule **6b**
(PCM for CH_2Cl_2)

E = -1584.85673207, **H (0K)** = -1584.527548,
H (298K) = --1584.503619, **G (298K)** = -1584.583277 au
Imaginary frequency = 0.

C	-0.4276420	3.4494280	0.9042230
C	-0.7097390	2.1578730	1.2355100
N	-0.3418390	1.3700630	0.1540420
C	0.1426060	2.1727940	-0.8075770
C	-0.4676420	-0.0507380	0.0644200
C	0.6013030	-0.9487100	0.0207840
C	-1.3515090	-2.1093020	-0.0901700
C	-1.7052920	-0.7612510	-0.0032530

C	2.0132500	-0.7819170	0.0438980	C	2.0564900	-0.7557740	0.0536480
C	2.8465570	-1.6095560	-0.7342330	C	2.9029770	-1.5839160	-0.7076940
C	4.2339890	-1.4761470	-0.7008230	C	4.2880800	-1.4248330	-0.6748840
C	4.8095120	-0.4962470	0.1092860	C	4.8381540	-0.4181840	0.1188730
C	4.0122660	0.3342710	0.8983950	C	4.0267850	0.4204630	0.8835130
C	2.6262890	0.1826700	0.8678880	C	2.6436940	0.2437690	0.8516860
C	-3.0923960	-0.0742720	-0.0187940	C	-3.0510670	-0.1414600	0.0214180
C	-3.3328580	1.0768730	-0.7931330	C	-3.4175430	0.8092770	-0.9467900
C	-4.5817260	1.7032680	-0.7894310	C	-4.6728640	1.4236970	-0.9089830
C	-5.6200160	1.1852470	-0.0117770	C	-5.5827970	1.0982470	0.1006170
C	-5.3997690	0.0300620	0.7452870	C	-5.2291750	0.1519530	1.0689880
C	-4.1532330	-0.5968740	0.7432760	C	-3.9753130	-0.4605410	1.0305990
H	-0.6316380	4.4092360	1.3809710	H	-0.5581520	4.3650760	1.4580060
H	-1.4652450	1.8086590	1.9264430	H	-1.1302510	1.7285460	2.1298920
H	0.8799240	1.7845020	-1.5792560	H	0.5000120	1.8432460	-1.7702310
H	2.4039550	-2.3585770	-1.3840450	H	2.4807080	-2.3510520	-1.3493310
H	4.8621550	-2.1215750	-1.3050200	H	4.9278340	-2.0691280	-1.2676500
H	4.4724300	1.0709950	1.5482650	H	4.4669200	1.1897590	1.5083060
H	2.0177510	0.7952710	1.5263790	H	2.0234590	0.8757350	1.4782900
H	-2.5494060	1.4633860	-1.4404160	H	-2.7254750	1.0529360	-1.7482150
H	-4.7478980	2.5805830	-1.4092290	H	-4.9406220	2.1487260	-1.6724730
H	-6.5951820	1.6640600	-0.0114200	H	-6.5596120	1.5723190	0.1303340
H	-6.2081140	-0.3975090	1.3315000	H	-5.9318070	-0.1115220	1.8544850
H	-4.0026240	-1.5226570	1.2852160	H	-3.7082420	-1.1987970	1.7793730
N	0.3250930	3.4257280	-0.2873510	N	0.1071470	3.4359220	-0.3730330
C	-2.1584670	-3.4213550	-0.1431110	C	-2.1121610	-3.4146250	-0.2197630
O	-3.4012740	-3.3892220	-0.0568790	O	-3.3640620	-3.3595750	-0.3094340
O	-1.3518230	-4.3835960	-0.2571170	O	-1.3713550	-4.4441490	-0.2305490
Cl	6.5562440	-0.3111120	0.1459450	Cl	6.5862250	-0.2043730	0.1596810
N	-0.0586900	-2.1571020	-0.0972450	N	0.0189940	-2.1785420	-0.0845920
H	0.3226260	-3.1041270	-0.1235960	H	0.4762290	-3.0830560	-0.1032600
C	0.9755950	4.5623120	-0.9426140	C	0.5548890	4.6159900	-1.1238030
H	0.2246770	5.3012280	-1.2296880	H	-0.2836610	5.3023000	-1.2482980
H	1.7013610	5.0150140	-0.2636390	H	1.3613780	5.1061620	-0.5767550
H	1.4915500	4.2084000	-1.8351850	H	0.9152670	4.2958270	-2.1002890

Molecule **6b**
(PCM for THF)

E = -1584.85442422, **H (0K)** = -1584.525179,
H (298K) = -1584.501289, **G (298K)** = -1584.580711 au.
Imaginary frequency = 0.

Molecule **6b**
(PCM for DMSO)

E = -1584.86682991, **H (0K)** = -1584.537844,
H (298K) = -1584.513786, **G (298K)** = -1584.594255 au
Imaginary frequency = 0.

C	-0.4303040	3.4497010	0.9075380	C	-0.4199410	3.4448320	0.9025240
C	-0.7141620	2.1579410	1.2367690	C	-0.6867450	2.1531040	1.2444120
N	-0.3424580	1.3706960	0.1563700	N	-0.3413130	1.3643470	0.1553320
C	0.1460010	2.1737130	-0.8027150	C	0.1156800	2.1677660	-0.8201030
C	-0.4685440	-0.0501810	0.0657530	C	-0.4628100	-0.0564100	0.0706520
C	0.6007540	-0.9478460	0.0218350	C	0.6062370	-0.9539470	0.0264260
C	-1.3519780	-2.1087620	-0.0885250	C	-1.3450500	-2.1160160	-0.0860260
C	-1.7064110	-0.7607620	-0.0019260	C	-1.6981010	-0.7685840	0.0025520
C	2.0558870	-0.7552590	0.0539630	C	2.0612660	-0.7567770	0.0564070
C	2.9016020	-1.5839370	-0.7077630	C	2.9088120	-1.5799070	-0.7088020
C	4.2867780	-1.4255340	-0.6760470	C	4.2933450	-1.4147230	-0.6787980
C	4.8381800	-0.4189970	0.1169540	C	4.8398170	-0.4072560	0.1163520
C	4.0276520	0.4199540	0.8822070	C	4.0270670	0.4273420	0.8837440
C	2.6444480	0.2439400	0.8515180	C	2.6446890	0.2447130	0.8543870
C	-3.0522060	-0.1410250	0.0195710	C	-3.0445890	-0.1479640	0.0286800
C	-3.4120750	0.8177370	-0.9432960	C	-3.4485270	0.7221870	-0.9981060
C	-4.6671980	1.4326150	-0.9090030	C	-4.7040280	1.3371320	-0.9621460
C	-5.5840090	1.0995140	0.0917740	C	-5.5743600	1.0938370	0.1042200
C	-5.2375340	0.1445730	1.0541560	C	-5.1808690	0.2313820	1.1337790
C	-3.9839720	-0.4685880	1.0191060	C	-3.9265150	-0.3812240	1.0972900

H	-0.5627090	4.3650210	1.4614420	H	-0.5401760	4.3613550	1.4570400
H	-1.1389450	1.7280540	2.1288780	H	-1.0800750	1.7243250	2.1512570
H	0.5070620	1.8445240	-1.7641330	H	0.4516150	1.8382520	-1.7904140
H	2.4783630	-2.3511530	-1.3486820	H	2.4889290	-2.3466900	-1.3523510
H	4.9257740	-2.0704310	-1.2689920	H	4.9344660	-2.0541120	-1.2753460
H	4.4688680	1.1886570	1.5069970	H	4.4641120	1.1996600	1.5068370
H	2.0250060	0.8756690	1.4792050	H	2.0230320	0.8757970	1.4802250
H	-2.7151500	1.0665470	-1.7389360	H	-2.7840670	0.9056550	-1.8378840
H	-4.9297580	2.1634460	-1.6687930	H	-5.0008890	2.0015470	-1.7686590
H	-6.5608830	1.5736510	0.1186940	H	-6.5499880	1.5702960	0.1333840
H	-5.9459620	-0.1259060	1.8320190	H	-5.8503020	0.0372080	1.9670340
H	-3.7230820	-1.2144350	1.7623780	H	-3.6249220	-1.0485640	1.8985060
N	0.1095340	3.4368480	-0.3676310	N	0.0833050	3.4305900	-0.3874790
C	-2.1106580	-3.4167570	-0.2162340	C	-2.1137440	-3.4104000	-0.2168850
O	-3.3625500	-3.3636610	-0.2986030	O	-3.3665000	-3.3469030	-0.3256440
O	-1.3658390	-4.4430010	-0.2319980	O	-1.3912300	-4.4539990	-0.2123810
Cl	6.5861680	-0.2059210	0.1562760	Cl	6.5879380	-0.1862860	0.1539920
N	0.0181770	-2.1775230	-0.0835740	N	0.0270500	-2.1853430	-0.0790180
H	0.4735770	-3.0831560	-0.1024860	H	0.4933180	-3.0845050	-0.0970380
C	0.5599170	4.6171190	-1.1162710	C	0.5107060	4.6108640	-1.1500800
H	-0.2780740	5.3036340	-1.2437090	H	-0.3311710	5.2965930	-1.2512010
H	1.3645650	5.1072760	-0.5664650	H	1.3314530	5.1002300	-0.6241000
H	0.9238410	4.2974490	-2.0916500	H	0.8442130	4.2901500	-2.1356760
Molecule **4e** (gas phase)				Molecule **4e** (PCM for CH_2Cl_2)			
**E =** -1316.97385134, **H (0K)** = -1316.583121 , **H (298K)** = -1316.557605, **G (298K)** = -1316.640531 au.				**E =** -1317.00587213, **H (0K)** = -1316.615093, **H (298K)** = -1316.589591, **G (298K)** = -1316.672794 au			
Imaginary frequency = 0.				Imaginary frequency = 0.			
C	-1.6047920	-1.8821580	1.3579120	C	-1.8376350	-1.4427700	1.5885870
C	-0.3318970	-1.3991270	1.4201810	C	-0.5438790	-1.0237520	1.6649690
N	-0.2008340	-0.4385690	0.4270720	N	-0.2815230	-0.2795590	0.5229580
C	-1.3625970	-0.3540010	-0.2292270	C	-1.3894740	-0.2551670	-0.2291310
C	0.9464460	0.3685310	0.1687670	C	0.9551670	0.3635470	0.1959020
C	0.9490260	1.7826320	0.0399860	C	1.1590860	1.7612510	0.0450810
N	2.2031740	2.1829170	-0.2379290	N	2.4474500	1.9750190	-0.2850170
C	2.9973220	1.0698330	-0.2623810	C	3.0707900	0.7537000	-0.3248460
C	2.2645060	-0.1124880	-0.0224320	C	2.1771320	-0.3039290	-0.0349170
C	-0.1595530	2.7416720	0.1914680	C	0.2031080	2.8746060	0.1970030
C	-0.2164360	3.8821570	-0.6325620	C	0.3383670	4.0264710	-0.6028760
C	-1.2499300	4.8101750	-0.5031890	C	-0.5404330	5.1019480	-0.4677350
C	-2.2566210	4.6232410	0.4505610	C	-1.5805030	5.0531220	0.4677840
C	-2.2066440	3.5034790	1.2860990	C	-1.7231950	3.9193380	1.2733600
C	-1.1683340	2.5766120	1.1615830	C	-0.8398190	2.8442580	1.1432240
C	2.6950340	-1.5268350	-0.0175580	C	2.4089510	-1.7645240	0.0103490
C	2.0058420	-2.4860880	-0.7836820	C	1.6090010	-2.6421770	-0.7446950
C	2.3784000	-3.8323630	-0.7624980	C	1.8034280	-4.0251300	-0.6849090
C	3.4536830	-4.2490730	0.0260880	C	2.8041470	-4.5592490	0.1318810
C	4.1553220	-3.3050340	0.7830940	C	3.6091050	-3.6977300	0.8856430
C	3.7819680	-1.9609800	0.7625100	C	3.4121690	-2.3169930	0.8267510
H	-2.1152750	-2.6125760	1.9636390	H	-2.4377030	-2.0191800	2.2733540
H	0.4894650	-1.6376710	2.0752830	H	0.2056910	-1.1718340	2.4245420
H	-1.5524890	0.2931400	-1.0690570	H	-1.4798640	0.2227350	-1.1909860
H	0.5667640	4.0279690	-1.3693900	H	1.1409570	4.0655420	-1.3322620
H	-1.2700050	5.6836970	-1.1491900	H	-0.4162750	5.9786630	-1.0974790
H	-3.0587430	5.3485320	0.5523630	H	-2.2656340	5.8894380	0.5713120
H	-2.9616020	3.3650580	2.0560240	H	-2.5145830	3.8755100	2.0165860
H	-1.1151520	1.7425080	1.8564010	H	-0.9458960	1.9917710	1.8070260
H	1.1915080	-2.1664640	-1.4289710	H	0.8436740	-2.2372860	-1.4007900
H	1.8397040	-4.5507600	-1.3749900	H	1.1788680	-4.6826010	-1.2834320
H	3.7513880	-5.2937340	0.0397500	H	2.9589750	-5.6333920	0.1774740

H	5.0043070	-3.6144180	1.3862760	H	4.3918040	-4.1016360	1.5217380
H	4.3458600	-1.2326320	1.3338480	H	4.0411240	-1.6554090	1.4131710
N	-2.2473030	-1.2089160	0.3264700	N	-2.3561490	-0.9457170	0.3996700
C	4.4315170	1.2565860	-0.5600350	C	4.4843230	0.7393600	-0.7008550
O	5.2722730	0.3770020	-0.6086590	O	5.2112500	-0.2401710	-0.8226400
O	4.7649900	2.5541370	-0.7825680	O	4.9898960	1.9837480	-0.9322880
H	3.9395960	3.0720050	-0.6765790	H	4.2442500	2.6078060	-0.7857240
C	-3.6139320	-1.3680490	-0.0773450	C	-3.6969300	-1.1378130	-0.0854590
C	-4.1452590	-2.6538240	-0.2088570	C	-4.2367400	-2.4261360	-0.0934090
C	-4.3899720	-0.2336850	-0.3323980	C	-4.4295260	-0.0362880	-0.5339410
C	-5.4781740	-2.8013730	-0.5959230	C	-5.5402520	-2.6093500	-0.5587480
H	-3.5215110	-3.5242050	-0.0317840	H	-3.6447750	-3.2711450	0.2422500
C	-5.7164540	-0.3969260	-0.7358610	C	-5.7270760	-0.2368200	-1.0102360
H	-3.9703950	0.7585660	-0.1973750	H	-4.0030270	0.9607200	-0.4957320
C	-6.2633110	-1.6765270	-0.8638320	C	-6.2841030	-1.5189170	-1.0203270
H	-5.8963930	-3.7973430	-0.7015510	H	-5.9674160	-3.6068280	-0.5702080
H	-6.3240840	0.4798670	-0.9357920	H	-6.3037750	0.6132120	-1.3600790
H	-7.2972620	-1.7970050	-1.1711770	H	-7.2952850	-1.6680340	-1.3853150
Molecule **4e** (PCM for DMSO)				Molecule **5e** (gas phase)			
**E =** -1317.01162421, **H (0K)** = -1316.621026, **H (298K)** = -1316.595470, **G (298K)** = -1316.678989 au.				**E =** -1316.96868083, **H (0K)** = -1316.578101, **H (298K)** = -1316.552354, **G (298K)** = -1316.636213 au.			
Imaginary frequency = 0.				Imaginary frequency = 0.			
C	-1.9078210	-1.2613940	1.6604980	C	-2.0190730	-0.9773030	1.7045840
C	-0.6080910	-0.8627040	1.7374490	C	-0.7087160	-0.6459790	1.7861200
N	-0.2982890	-0.2290660	0.5413890	N	-0.3655370	-0.1119330	0.5393570
C	-1.3861600	-0.2465230	-0.2402930	C	-1.4140010	-0.0974910	-0.3450070
C	0.9589810	0.3672110	0.2005680	C	0.9375200	0.3491180	0.2152650
C	1.2119610	1.7538570	0.0184740	C	1.3101900	1.6910920	0.0644160
N	2.5085330	1.9163200	-0.3098370	C	3.1221260	0.3910680	-0.3187300
C	3.0890330	0.6721310	-0.3193810	C	2.0663520	-0.4819750	-0.0227090
C	2.1568680	-0.3461400	-0.0088180	C	0.5527010	2.9435830	0.1841360
C	0.2906780	2.9008720	0.1325800	C	0.8822050	4.0517390	-0.6182990
C	0.4768820	4.0322790	-0.6863650	C	0.1877520	5.2550560	-0.4925560
C	-0.3702950	5.1369720	-0.5889730	C	-0.8544870	5.3716710	0.4307130
C	-1.4294790	5.1377990	0.3263220	C	-1.1957430	4.2742060	1.2265110
C	-1.6233980	4.0242860	1.1492720	C	-0.5000870	3.0715400	1.1080470
C	-0.7713830	2.9203190	1.0574780	C	2.0625770	-1.9619660	0.0261350
C	2.3389100	-1.8127730	0.0769070	C	1.1590890	-2.6858640	-0.7703340
C	1.5496220	-2.6800850	-0.7005010	C	1.1251290	-4.0805970	-0.7147720
C	1.6968840	-4.0671190	-0.6039680	C	1.9859980	-4.7725020	0.1413350
C	2.6378930	-4.6145150	0.2729260	C	2.8837870	-4.0602010	0.9417110
C	3.4308580	-3.7630120	1.0508430	C	2.9216690	-2.6662700	0.8848550
C	3.2813930	-2.3780920	0.9548810	H	-2.6823820	-1.3882290	2.4483180
H	-2.5256310	-1.8007070	2.3593350	H	-0.0039170	-0.7351380	2.5978790
H	0.1208590	-0.9705790	2.5234200	H	1.6633620	3.9655520	-1.3685750
H	-1.4532500	0.1866510	-1.2249940	H	0.4537210	6.0966210	-1.1254520
H	1.2915390	4.0323540	-1.4032600	H	-1.3984020	6.3067170	0.5261080
H	-0.2076230	5.9965240	-1.2334070	H	-2.0045900	4.3550340	1.9468290
H	-2.0911450	5.9958860	0.3995510	H	-0.7676690	2.2319150	1.7395730
H	-2.4323620	4.0169550	1.8744280	H	0.4877080	-2.1516310	-1.4356420
H	-0.9207610	2.0832020	1.7319070	H	0.4252280	-4.6252110	-1.3421160
H	0.8285130	-2.2656400	-1.3991790	H	1.9576670	-5.8575480	0.1852130
H	1.0800930	-4.7172680	-1.2181110	H	3.5551820	-4.5893970	1.6121300
H	2.7542660	-5.6917530	0.3486670	H	3.6210230	-2.1181850	1.5083120
H	4.1646660	-4.1780800	1.7361450	N	-2.4292160	-0.6372080	0.4103200
H	3.8978960	-1.7242620	1.5635210	C	4.5183370	0.2494550	-0.7047040
N	-2.3816040	-0.8649150	0.4161600	O	5.2442690	1.2086690	-0.9407450
C	4.4983320	0.5972720	-0.6908580	O	4.9584320	-1.0267440	-0.7920410

O	5.1905190	-0.4112250	-0.7955460	N	2.6314890	1.6782410	-0.2653010
O	5.0542260	1.8163250	-0.9450460	H	3.2264780	2.4829320	-0.4092940
H	4.3329480	2.4721380	-0.8148160	H	5.8871630	-0.9768150	-1.0737670
C	-3.7092970	-1.0850680	-0.0938650	C	-3.7498220	-0.8359700	-0.0936280
C	-4.8061850	-0.7787690	0.7148660	C	-4.5360040	-1.8928200	0.3791700
C	-3.8727560	-1.6018550	-1.3811860	C	-4.2551300	0.0325050	-1.0684760
C	-6.0929290	-0.9987220	0.2193650	C	-5.8317810	-2.0692430	-0.1127260
H	-4.6586810	-0.3624500	1.7056670	H	-4.1353910	-2.5898830	1.1079800
C	-5.1659050	-1.8028940	-1.8691450	C	-5.5453960	-0.1602790	-1.5616050
H	-3.0084690	-1.8590570	-1.9845740	H	-3.6262040	0.8375100	-1.4298110
C	-6.2747210	-1.5057080	-1.0711970	C	-6.3423770	-1.2057910	-1.0841070
H	-6.9510080	-0.7623150	0.8401160	H	-6.4347860	-2.8930320	0.2579220
H	-5.3022410	-2.2042820	-2.8680420	H	-5.9315130	0.5163760	-2.3184310
H	-7.2773170	-1.6702490	-1.4528990	H	-7.3483130	-1.3473780	-1.4675810

Molecule **5e** (PCM for CH_2Cl_2)				Molecule **5e** (PCM for DMSO)			
**E =** -1316.98528398, **H (0K)** = -1316.594876, **H (298K)** = -1316.569119, **G (298K)** = -1316.652812 au				**E =** -1316.98867650, **H (0K)** = -1316.598431, **H (298K)** = -1316.572600, **G (298K)** = -1316.656942 au			
Imaginary frequency = 0.				Imaginary frequency = 0.			
C	-2.0038540	-1.0139560	1.6809620	C	-2.0076940	-1.0011340	1.6838650
C	-0.6975400	-0.6663990	1.7633090	C	-0.7010720	-0.6542070	1.7659500
N	-0.3629770	-0.1152560	0.5218170	N	-0.3644940	-0.1089770	0.5225380
C	-1.4153580	-0.1045670	-0.3578860	C	-1.4164160	-0.1016010	-0.3574810
C	0.9347220	0.3628100	0.1984180	C	0.9344490	0.3651070	0.1977050
C	1.2918520	1.7104280	0.0510560	C	1.2967610	1.7115010	0.0486000
C	3.1179500	0.4346280	-0.3383580	C	3.1173380	0.4284290	-0.3410340
C	2.0737120	-0.4522310	-0.0390710	C	2.0700190	-0.4540650	-0.0403060
C	0.5155600	2.9513240	0.1829810	C	0.5241460	2.9550680	0.1790470
C	0.8034410	4.0605030	-0.6345550	C	0.8130050	4.0609700	-0.6428530
C	0.0863410	5.2501280	-0.4978380	C	0.0985770	5.2526220	-0.5083630
C	-0.9349970	5.3509610	0.4515700	C	-0.9205420	5.3587070	0.4430710
C	-1.2322910	4.2525180	1.2648390	C	-1.2180400	4.2638160	1.2613230
C	-0.5145850	3.0630560	1.1351020	C	-0.5029790	3.0722860	1.1340250
C	2.0969630	-1.9321770	0.0284970	C	2.0895480	-1.9343730	0.0290250
C	1.2472290	-2.6886790	-0.7962380	C	1.2621960	-2.6917200	-0.8172840
C	1.2392980	-4.0838060	-0.7194130	C	1.2540970	-4.0871920	-0.7408360
C	2.0744770	-4.7431150	0.1873800	C	2.0668250	-4.7451030	0.1874540
C	2.9192390	-3.9980700	1.0168510	C	2.8885660	-3.9988790	1.0390100
C	2.9296000	-2.6039060	0.9388210	C	2.8989620	-2.6045300	0.9612790
H	-2.6625650	-1.4440020	2.4183190	H	-2.6680450	-1.4274990	2.4219030
H	0.0075040	-0.7468710	2.5756420	H	0.0029620	-0.7296400	2.5795880
H	1.5715620	3.9882570	-1.3991250	H	1.5811780	3.9852750	-1.4069050
H	0.3193170	6.0932820	-1.1412510	H	0.3326550	6.0935940	-1.1541570
H	-1.4955100	6.2751670	0.5551110	H	-1.4783460	6.2846640	0.5452090
H	-2.0216680	4.3226330	2.0073720	H	-2.0044470	4.3386540	2.0064700
H	-0.7455910	2.2244570	1.7825100	H	-0.7326280	2.2374800	1.7868230
H	0.5969070	-2.1814350	-1.5022130	H	0.6296000	-2.1849610	-1.5396770
H	0.5808020	-4.6536430	-1.3686450	H	0.6134510	-4.6583170	-1.4065540
H	2.0669010	-5.8275170	0.2484400	H	2.0592130	-5.8294720	0.2482460
H	3.5688700	-4.5014930	1.7271580	H	3.5197470	-4.5015380	1.7661900
H	3.5845390	-2.0309390	1.5879380	H	3.5353640	-2.0298700	1.6271210
N	-2.4220910	-0.6626640	0.3924010	N	-2.4245770	-0.6547750	0.3935450
C	4.5126320	0.2916670	-0.7256970	C	4.5124710	0.2759860	-0.7233120
O	5.2473810	1.2521400	-0.9459460	O	5.2542180	1.2314290	-0.9436710
O	4.9396400	-0.9831710	-0.8378810	O	4.9313190	-1.0016170	-0.8302360
N	2.6114340	1.7158490	-0.2816770	N	2.6157980	1.7116000	-0.2858330
H	3.1866210	2.5348330	-0.4286220	H	3.1933530	2.5292290	-0.4319850
H	5.8737890	-0.9533880	-1.1078180	H	5.8680500	-0.9810530	-1.0926280
C	-3.7460460	-0.8736400	-0.1020480	C	-3.7487590	-0.8686560	-0.1006250

C	-4.4613800	-2.0166830	0.2735660	C	-4.4514250	-2.0247960	0.2585500
C	-4.3245890	0.0680010	-0.9610740	C	-4.3397880	0.0824190	-0.9406440
C	-5.7595320	-2.2106020	-0.2063340	C	-5.7493840	-2.2232870	-0.2202300
H	-4.0044830	-2.7595420	0.9192740	H	-3.9853070	-2.7725140	0.8919020
C	-5.6167070	-0.1416650	-1.4463070	C	-5.6318780	-0.1314340	-1.4249970
H	-3.7598410	0.9504690	-1.2381310	H	-3.7871870	0.9771390	-1.2029770
C	-6.3411750	-1.2774670	-1.0691180	C	-6.3432460	-1.2811010	-1.0650370
H	-6.3086700	-3.1002660	0.0871240	H	-6.2890720	-3.1227310	0.0603520
H	-6.0608300	0.5917310	-2.1128090	H	-6.0862620	0.6093840	-2.0761210
H	-7.3481640	-1.4333350	-1.4439290	H	-7.3501020	-1.4402470	-1.4387060
Molecule **6e**<br>(gas phase)<br><br>**E =** -1316.95522916, **H (0K)** = -1316.564550,<br>**H (298K)** = -1316.538979, **G (298K)** = -1316.622112 au.<br>Imaginary frequency = 0.				Molecule **6e**<br>(PCM for CH_2Cl_2)<br><br>**E =** -1317.00878179, **H (0K)** = -1316.617544,<br>**H (298K)** = -1316.591916, **G (298K)** = -1316.675834 au<br>Imaginary frequency = 0.			
C	-1.6685540	-1.7339210	1.4573060	C	-1.8413190	-1.3611950	1.6248800
C	-0.3895570	-1.2669640	1.5129300	C	-0.5485390	-0.9403400	1.6962680
N	-0.2274760	-0.3788240	0.4607710	N	-0.2771750	-0.2464420	0.5248580
C	-1.3741290	-0.3209450	-0.2243640	C	-1.3793260	-0.2513160	-0.2380000
C	0.9529760	0.3679930	0.1634040	C	0.9616200	0.3828720	0.1901660
C	0.9828660	1.7619950	0.0356720	C	1.1392750	1.7582420	0.0256070
C	3.0751840	0.9296180	-0.2882250	C	3.1244940	0.7021990	-0.3223620
C	2.2590630	-0.1810190	-0.0442840	C	2.1989210	-0.3006990	-0.0250990
C	-0.0552330	2.7907510	0.1687090	C	0.2159550	2.8975080	0.1264040
C	-0.0732210	3.9002400	-0.6993720	C	0.3385230	3.9885050	-0.7555570
C	-1.0483220	4.8903640	-0.5729840	C	-0.5216910	5.0832130	-0.6573610
C	-2.0340210	4.7917690	0.4136430	C	-1.5270390	5.1057170	0.3144840
C	-2.0253020	3.6983780	1.2854160	C	-1.6593020	4.0266540	1.1942390
C	-1.0436460	2.7127990	1.1706430	C	-0.7937050	2.9350300	1.1068290
C	2.6099100	-1.6176880	-0.0386350	C	2.3923560	-1.7677460	0.0509720
C	1.7746330	-2.5610480	-0.6671570	C	1.6046470	-2.6308300	-0.7309310
C	2.0764080	-3.9249510	-0.6413550	C	1.7552700	-4.0179180	-0.6424920
C	3.2266870	-4.3729880	0.0123190	C	2.6970610	-4.5663900	0.2322730
C	4.0759920	-3.4433890	0.6213580	C	3.4885360	-3.7172160	1.0137310
C	3.7767770	-2.0807480	0.5964440	C	3.3369010	-2.3323500	0.9248850
H	-2.2023560	-2.4166470	2.0976060	H	-2.4339880	-1.9460820	2.3086530
H	0.4181780	-1.4777010	2.1945510	H	0.1997270	-1.0721900	2.4601870
H	-1.5361200	0.2656170	-1.1135990	H	-1.4736230	0.2258530	-1.2000180
H	0.6754280	3.9776010	-1.4825300	H	1.0975160	3.9722420	-1.5322460
H	-1.0393620	5.7389640	-1.2508040	H	-0.4110750	5.9146020	-1.3471710
H	-2.7907600	5.5643530	0.5112980	H	-2.1979930	5.9562140	0.3877270
H	-2.7675180	3.6271890	2.0759470	H	-2.4282690	4.0401690	1.9610820
H	-1.0162580	1.8994590	1.8902590	H	-0.8861000	2.1227220	1.8206630
H	0.9014180	-2.2224970	-1.2193650	H	0.8852510	-2.2141340	-1.4302840
H	1.4249170	-4.6318590	-1.1486410	H	1.1428850	-4.6666900	-1.2624960
H	3.4696430	-5.4317770	0.0293620	H	2.8176660	-5.6436770	0.3011140
H	4.9879660	-3.7779930	1.1075120	H	4.2271100	-4.1338990	1.6927090
H	4.4651510	-1.3582430	1.0179450	H	3.9580380	-1.6787420	1.5275490
N	-2.2790980	-1.1244950	0.3676840	N	-2.3478880	-0.9183960	0.4091130
C	4.5692520	1.2047270	-0.5635120	C	4.5983610	0.7374220	-0.6767090
O	5.3382070	0.2241960	-0.5262330	O	5.2100420	-0.3566730	-0.7623350
O	4.7857980	2.4292690	-0.7722000	O	5.0586260	1.9059500	-0.8552780
N	2.2846220	2.0424730	-0.2549160	N	2.4573810	1.9015400	-0.2951510
H	2.7618620	2.9342040	-0.3931530	H	2.9645390	2.7643250	-0.4553440
C	-3.6384400	-1.3045450	-0.0598100	C	-3.6833580	-1.1386400	-0.0827750
C	-4.1573430	-2.5977800	-0.1604820	C	-4.7676630	-0.8713810	0.7560650
C	-4.4146710	-0.1837270	-0.3661410	C	-3.8648400	-1.6142350	-1.3832310
C	-5.4807210	-2.7662520	-0.5710170	C	-6.0608600	-1.0882200	0.2766180
H	-3.5317540	-3.4573880	0.0579720	H	-4.6068040	-0.4865900	1.7576180
C	-5.7316360	-0.3683480	-0.7913400	C	-5.1645370	-1.8126870	-1.8541580

H	-4.0035330	0.8148920	-0.2548080	H	-3.0088010	-1.8417440	-2.0099040
C	-6.2667160	-1.6554210	-0.8902030	C	-6.2610110	-1.5535470	-1.0267420
H	-5.8906480	-3.7676740	-0.6541830	H	-6.9098490	-0.8817930	0.9201210
H	-6.3408770	0.4970710	-1.0314720	H	-5.3154180	-2.1821330	-2.8631820
H	-7.2931490	-1.7927740	-1.2149860	H	-7.2687690	-1.7154840	-1.3956360

Molecule **6e**
(PCM for DMSO)

E = -1317.01845877, **H (0K)** = -1316.627216,
H (298K) = -1316.601571, **G (298K)** = -1316.685398 au.
Imaginary frequency = 0.

C	-1.8745380	-1.2791090	1.6505650
C	-0.5805380	-0.8646520	1.7306180
N	-0.2849620	-0.2108740	0.5414040
C	-1.3747220	-0.2333300	-0.2396050
C	0.9648360	0.3919060	0.2013740
C	1.1753180	1.7605980	0.0209410
C	3.1266290	0.6505130	-0.3468620
C	2.1802710	-0.3221080	-0.0239660
C	0.2795620	2.9213340	0.1254540
C	0.4177250	4.0069460	-0.7606790
C	-0.4171230	5.1207030	-0.6563010
C	-1.4117740	5.1671670	0.3258580
C	-1.5600330	4.0925490	1.2086780
C	-0.7199560	2.9816770	1.1149860
C	2.3396950	-1.7934780	0.0741590
C	1.6755140	-2.6439390	-0.8257470
C	1.7963420	-4.0331310	-0.7188110
C	2.5806090	-4.5936140	0.2934880
C	3.2431000	-3.7557290	1.1976410
C	3.1209610	-2.3686570	1.0902220
H	-2.4833000	-1.8351060	2.3441890
H	0.1520720	-0.9743690	2.5128900
H	-1.4496320	0.2109890	-1.2188870
H	1.1671540	3.9721310	-1.5459960
H	-0.2960770	5.9476740	-1.3496480
H	-2.0635340	6.0320910	0.4034850
H	-2.3225850	4.1229960	1.9813020
H	-0.8263310	2.1711390	1.8286810
H	1.0739300	-2.2164410	-1.6230790
H	1.2804330	-4.6742400	-1.4279640
H	2.6755600	-5.6723210	0.3775890
H	3.8538970	-4.1823840	1.9882330
H	3.6358590	-1.7224310	1.7944000
N	-2.3571810	-0.8731450	0.4123340
C	4.5885090	0.6210020	-0.7245530
O	5.1523320	-0.5011010	-0.8198270
O	5.1063890	1.7635920	-0.9185100
N	2.4921930	1.8696060	-0.3200730
H	3.0101430	2.7228070	-0.4922970
C	-3.6855950	-1.1021810	-0.0944310
C	-4.7811490	-0.8103300	0.7211950
C	-3.8488420	-1.6109090	-1.3847420
C	-6.0678130	-1.0363390	0.2284250
H	-4.6334590	-0.4005600	1.7146690
C	-5.1422030	-1.8180480	-1.8695500
H	-2.9845120	-1.8571660	-1.9925610
C	-6.2501590	-1.5347930	-1.0654070
H	-6.9254500	-0.8112840	0.8539530
H	-5.2791690	-2.2130620	-2.8708550

H	-7.2528840	-1.7037900	-1.4447810				
Molecule **4i** (gas phase)				Molecule **4i** (PCM for CH_2Cl_2)			
**E =** -1815.88526871, **H (0K)** = -1815.475464, **H (298K)** = -1815.447224, **G (298K)** = -1815.539072 au.				**E =** -1815.92013867, **H (0K)** = -1815.509895, **H (298K)** = -1815.481845, **G (298K)** = -1815.571955 au			
Imaginary frequency = 0.				Imaginary frequency = 0.			
C	2.1527550	-0.3955870	1.3514850	C	1.7597370	-1.2357930	1.5006760
C	1.0783380	0.4372650	1.4718830	C	0.9173050	-0.1743570	1.6510240
N	0.2278000	0.1764620	0.4086880	N	0.2036130	-0.0442900	0.4683090
C	0.7828950	-0.7843760	-0.3400730	C	0.6120680	-1.0051150	-0.3741090
C	-1.0391230	0.7872060	0.1551360	C	-0.8054390	0.9309540	0.1806830
C	-2.2638160	0.0993460	-0.0486280	C	-2.1849480	0.6700000	-0.0334400
N	-3.2252720	1.0064890	-0.3061120	N	-2.8068910	1.8322540	-0.3131870
C	-2.6525510	2.2449430	-0.2435360	C	-1.8635850	2.8254760	-0.2587350
C	-1.2690920	2.1785810	0.0355080	C	-0.5779930	2.3166380	0.0446380
C	-2.5555440	-1.3416180	0.0111630	C	-2.9188250	-0.6068740	0.0093330
C	-3.5382690	-1.8940550	-0.8330070	C	-4.0431640	-0.7957010	-0.8178040
C	-3.8458070	-3.2531600	-0.7985430	C	-4.7725290	-1.9837220	-0.7945990
C	-3.1635550	-4.0878810	0.0885940	C	-4.3746490	-3.0089500	0.0647600
C	-2.1973030	-3.5724060	0.9515190	C	-3.2718630	-2.8556490	0.9033460
C	-1.9046320	-2.2076080	0.9112780	C	-2.5552980	-1.6573440	0.8733430
C	-0.2572080	3.2510280	0.1440770	C	0.7164910	3.0178450	0.1925330
C	0.9521220	3.1704320	-0.5719830	C	1.8467910	2.6028890	-0.5356110
C	1.9324040	4.1584640	-0.4503510	C	3.0802550	3.2409010	-0.3759190
C	1.7218470	5.2539420	0.3904050	C	3.2080520	4.3097080	0.5156210
C	0.5198190	5.3537400	1.0986190	C	2.0911680	4.7352720	1.2435590
C	-0.4576180	4.3657080	0.9783210	C	0.8607380	4.0953380	1.0854330
H	3.0406820	-0.4963540	1.9539270	H	2.4823550	-1.6635720	2.1761530
H	0.8412270	1.1938640	2.2015360	H	0.7560200	0.4919620	2.4823960
H	0.3431640	-1.1986560	-1.2329310	H	0.2371940	-1.1524840	-1.3742580
H	-4.0632090	-1.2371420	-1.5184050	H	-4.3431710	0.0024640	-1.4881840
H	-4.6047510	-3.6633810	-1.4562610	H	-5.6343280	-2.1127600	-1.4405850
H	-1.6990570	-4.2240640	1.6617520	H	-2.9829920	-3.6509880	1.5819780
H	-1.1898390	-1.8079580	1.6249480	H	-1.7231960	-1.5381210	1.5592750
H	1.1102180	2.3413150	-1.2570010	H	1.7548510	1.7878660	-1.2478230
H	2.8517040	4.0804660	-1.0250170	H	3.9374860	2.9069380	-0.9538730
H	2.4784390	6.0280420	0.4822510	H	4.1649440	4.8085670	0.6392960
H	0.3377420	6.2097170	1.7423330	H	2.1784280	5.5669780	1.9371060
H	-1.3951340	4.4627630	1.5134370	H	-0.0006790	4.4318650	1.6527720
N	1.9480980	-1.1601600	0.2155030	N	1.5501290	-1.7442430	0.2298770
C	2.8160310	-2.2516580	-0.2766470	C	2.2337000	-2.9198410	-0.3641240
H	2.6923310	-3.1092930	0.3911800	H	1.9731690	-3.7882690	0.2449380
H	2.4230250	-2.5349160	-1.2568740	H	1.7962140	-3.0580850	-1.3546310
C	-3.5165250	3.4141110	-0.5048850	C	-2.3071940	4.1837110	-0.5738110
O	-3.1675430	4.5803590	-0.4836010	O	-1.6194830	5.1970790	-0.6202010
O	-4.8020860	3.0753720	-0.7822240	O	-3.6406010	4.2638830	-0.8434290
H	-4.8464080	2.0978110	-0.7290270	H	-3.9872720	3.3477050	-0.7602440
Cl	-3.5354450	-5.8095880	0.1295650	Cl	-5.2864920	-4.5210640	0.0955400
C	4.2719250	-1.8495580	-0.3637340	C	3.7349120	-2.7431190	-0.4412510
C	5.2174580	-2.4467870	0.4781610	C	4.5699180	-3.4039940	0.4689470
C	4.6926220	-0.8876910	-1.2941890	C	4.3067920	-1.9249390	-1.4272900
C	6.5665710	-2.0877500	0.3957090	C	5.9576670	-3.2425760	0.4032110
H	4.9029200	-3.2005050	1.1960220	H	4.1374040	-4.0512510	1.2276030
C	6.0370230	-0.5256780	-1.3740920	C	5.6915850	-1.7617630	-1.4923990
H	3.9688800	-0.4192490	-1.9566620	H	3.6714500	-1.4173660	-2.1485290
C	6.9767960	-1.1255530	-0.5284750	C	6.5197600	-2.4195360	-0.5757450
H	7.2915030	-2.5598870	1.0517000	H	6.5950720	-3.7611030	1.1129680
H	6.3528110	0.2195510	-2.0977280	H	6.1243570	-1.1281020	-2.2606270
H	8.0235740	-0.8449020	-0.5940260	H	7.5970550	-2.2946430	-0.6294820

Molecule **4i** (PCM for DMSO)				Molecule **5i** (gas phase)			
**E =** -1815.92656095, **H (0K)** = -1815.516657, **H (298K)** = -1815.488485, **G (298K)** = -1815.579502 au.<br>Imaginary frequency = 0.				**E =** -1815.87896128, **H (0K)** = -1815.469331, **H (298K)** = -1815.440918, **G (298K)** = -1815.532889 au.<br>Imaginary frequency = 0.			
C	1.7337120	-1.2788370	1.4873780	C	1.5990880	-1.4005580	1.5783000
C	0.8904880	-0.2197190	1.6457060	C	0.8094710	-0.3052100	1.7037690
N	0.1986830	-0.0628870	0.4525500	N	0.1822500	-0.1381770	0.4650130
C	0.6218000	-1.0058970	-0.4035800	C	0.5530760	-1.0961760	-0.4539410
C	-0.8014580	0.9232340	0.1718030	C	-0.7481760	0.8949450	0.1840850
C	-2.1877820	0.6831850	-0.0289540	C	-2.1189980	0.7088730	-0.0364550
N	-2.7975460	1.8560350	-0.2911610	C	-1.6515900	2.9113170	-0.2448890
C	-1.8388860	2.8361190	-0.2399290	C	-0.4384050	2.2772770	0.0575420
C	-0.5577570	2.3056010	0.0447500	C	-2.9602970	-0.4932530	-0.0256780
C	-2.9398920	-0.5836680	0.0076470	C	-4.0697430	-0.5954130	-0.8837850
C	-4.0890200	-0.7371050	-0.7925690	C	-4.8982300	-1.7166630	-0.8630590
C	-4.8375410	-1.9132670	-0.7737940	C	-4.6125920	-2.7566830	0.0208660
C	-4.4334430	-2.9619820	0.0537780	C	-3.5131870	-2.6867910	0.8777510
C	-3.3050410	-2.8449100	0.8635940	C	-2.6949510	-1.5596180	0.8514910
C	-2.5693960	-1.6581170	0.8385480	C	0.9096620	2.8720950	0.2043490
C	0.7471900	2.9903770	0.1846710	C	1.9726530	2.4017970	-0.5848050
C	1.8468110	2.6108490	-0.6065150	C	3.2532010	2.9394850	-0.4426550
C	3.0884820	3.2364190	-0.4587150	C	3.4931210	3.9483870	0.4939060
C	3.2544790	4.2550900	0.4840130	C	2.4431480	4.4175230	1.2883780
C	2.1683370	4.6429750	1.2771060	C	1.1619330	3.8830510	1.1454820
C	0.9295750	4.0158810	1.1301600	H	2.2668050	-1.8703770	2.2840750
H	2.4398900	-1.7254090	2.1679770	H	0.6447150	0.3607990	2.5363660
H	0.7137230	0.4269140	2.4893120	H	-4.2774560	0.1925830	-1.6019840
H	0.2669580	-1.1310180	-1.4140650	H	-5.7471190	-1.7875630	-1.5340640
H	-4.3934190	0.0785680	-1.4394590	H	-3.3036830	-3.5021630	1.5613240
H	-5.7176430	-2.0148680	-1.3996690	H	-1.8483450	-1.5062080	1.5265110
H	-3.0083120	-3.6596510	1.5151230	H	1.7875530	1.6182420	-1.3131350
H	-1.7149310	-1.5701770	1.5008390	H	4.0634680	2.5697850	-1.0644800
H	1.7246270	1.8332790	-1.3550610	H	4.4902530	4.3646440	0.6052980
H	3.9223100	2.9309010	-1.0844990	H	2.6211920	5.1995020	2.0211670
H	4.2181180	4.7427750	0.5992390	H	0.3497980	4.2509170	1.7644470
H	2.2869690	5.4326590	2.0137810	N	1.4232980	-1.8593130	0.2729970
H	0.0923400	4.3197330	1.7504290	C	2.0924810	-3.0391720	-0.2784260
N	1.5472000	-1.7583660	0.2017540	H	1.8552720	-3.9071180	0.3469800
C	2.2381800	-2.9239370	-0.4028240	H	1.6369870	-3.1972750	-1.2590750
H	1.9622790	-3.8033970	0.1828430	C	-2.0732820	4.2707640	-0.5516700
H	1.8208270	-3.0390830	-1.4046290	O	-3.2336030	4.5623910	-0.8182670
C	-2.2645860	4.2006820	-0.5371250	O	-1.0845230	5.1933770	-0.5277490
O	-1.5631500	5.2066780	-0.5884110	N	-2.6303320	1.9429250	-0.3032770
O	-3.6009370	4.3065530	-0.7855600	H	-3.6006460	2.1748720	-0.4678910
H	-3.9623490	3.3954470	-0.7059940	H	-1.4957090	6.0413190	-0.7652720
Cl	-5.3702200	-4.4593890	0.0795560	Cl	-5.6484610	-4.1757660	0.0534350
C	3.7414060	-2.7510390	-0.4454950	C	3.5977420	-2.8869280	-0.3980710
C	4.5577650	-3.4583830	0.4465460	C	4.4592680	-3.7820050	0.2465050
C	4.3334370	-1.8911660	-1.3831050	C	4.1480220	-1.8610070	-1.1808910
C	5.9475780	-3.3036110	0.4096300	C	5.8468390	-3.6596170	0.1146340
H	4.1090470	-4.1350870	1.1690270	H	4.0449190	-4.5840360	0.8533190
C	5.7201690	-1.7340630	-1.4187780	C	5.5310590	-1.7348390	-1.3122570
H	3.7114240	-1.3465760	-2.0886120	H	3.4849680	-1.1623990	-1.6840320
C	6.5301020	-2.4396490	-0.5211050	C	6.3854920	-2.6342690	-0.6642440
H	6.5703030	-3.8581920	1.1050800	H	6.5018330	-4.3626700	0.6213150
H	6.1687940	-1.0677010	-2.1493920	H	5.9445640	-0.9375950	-1.9235120
H	7.6088650	-2.3191980	-0.5519780	H	7.4621280	-2.5353210	-0.7681460
Molecule **5i**				Molecule **5i**			

(PCM for CH_2Cl_2)				(PCM for DMSO)			
**E =** -1815.89667585, **H (0K)** = -1815.487357, **H (298K)** = -1815.458849, **G (298K)** = -1815.551321 au				**E =** -1815.90027179, **H (0K)** = -1815.490993, **H (298K)** = -1815.462482, **G (298K)** = -1815.555191 au			
Imaginary frequency = 0.				Imaginary frequency = 0.			
C	1.5334530	-1.4789360	1.5680220	C	1.5073380	-1.5170510	1.5377000
C	0.7594410	-0.3726930	1.6968760	C	0.7328700	-0.4129660	1.6833990
N	0.1581200	-0.1749410	0.4502340	N	0.1499510	-0.1826180	0.4337670
C	0.5319600	-1.1256670	-0.4738790	C	0.5363700	-1.1105920	-0.5076020
C	-0.7389950	0.8877980	0.1685230	C	-0.7430050	0.8875190	0.1653150
C	-2.1177080	0.7529610	-0.0479250	C	-2.1247660	0.7620790	-0.0374670
C	-1.5710100	2.9354760	-0.2547950	C	-1.5669700	2.9422510	-0.2378600
C	-0.3814740	2.2570790	0.0454520	C	-0.3780560	2.2550690	0.0463980
C	-3.0004200	-0.4201080	-0.0334700	C	-3.0113260	-0.4084810	-0.0249960
C	-4.1101950	-0.4886830	-0.8950480	C	-4.1268290	-0.4680980	-0.8801490
C	-4.9742390	-1.5832920	-0.8712370	C	-4.9911270	-1.5627110	-0.8612140
C	-4.7215160	-2.6264350	0.0190480	C	-4.7328470	-2.6142610	0.0176810
C	-3.6244830	-2.5908650	0.8807510	C	-3.6312370	-2.5871760	0.8738040
C	-2.7708390	-1.4893670	0.8510010	C	-2.7774350	-1.4854020	0.8493320
C	0.9847990	2.8082190	0.2087980	C	0.9933840	2.7950420	0.2021040
C	2.0177120	2.4028000	-0.6523900	C	2.0217700	2.3688090	-0.6549870
C	3.3130740	2.9015800	-0.4915470	C	3.3230680	2.8545360	-0.5003090
C	3.5962630	3.8045770	0.5375680	C	3.6165540	3.7667830	0.5178430
C	2.5754850	4.2075290	1.4048220	C	2.6004220	4.1909300	1.3808600
C	1.2799920	3.7125970	1.2420490	C	1.2993320	3.7080400	1.2249540
H	2.1697280	-1.9792420	2.2817060	H	2.1293730	-2.0387120	2.2486510
H	0.5805180	0.2757910	2.5402090	H	0.5401040	0.2124430	2.5409810
H	-4.2954720	0.3044760	-1.6129060	H	-4.3177730	0.3324170	-1.5880970
H	-5.8235780	-1.6269580	-1.5437810	H	-5.8449580	-1.5989820	-1.5284310
H	-3.4418920	-3.4071630	1.5707300	H	-3.4450020	-3.4089960	1.5561640
H	-1.9288310	-1.4593560	1.5331050	H	-1.9329990	-1.4613100	1.5285350
H	1.8010520	1.7018260	-1.4526450	H	1.7981170	1.6608000	-1.4470480
H	4.0994200	2.5843560	-1.1703380	H	4.1054120	2.5204260	-1.1755750
H	4.6038020	4.1900490	0.6639680	H	4.6281820	4.1429370	0.6392170
H	2.7875520	4.9055860	2.2096060	H	2.8205020	4.8958110	2.1774330
H	0.4911910	4.0266160	1.9188860	H	0.5150940	4.0375410	1.8995020
N	1.3765690	-1.9146960	0.2530920	N	1.3696910	-1.9190570	0.2100520
C	2.0517620	-3.0931650	-0.2991770	C	2.0530490	-3.0853050	-0.3613290
H	1.8124690	-3.9606770	0.3237430	H	1.7959230	-3.9680230	0.2319170
H	1.6066690	-3.2538990	-1.2838150	H	1.6308050	-3.2163600	-1.3601090
C	-1.9208880	4.3168780	-0.5488370	C	-1.9110080	4.3272680	-0.5216870
O	-3.0696650	4.6784430	-0.7934950	O	-3.0614100	4.6985250	-0.7451150
O	-0.8798580	5.1746560	-0.5429000	O	-0.8635460	5.1763870	-0.5337960
N	-2.5852430	2.0040300	-0.3116570	N	-2.5874660	2.0171310	-0.2884090
H	-3.5501060	2.2597090	-0.4760050	H	-3.5527700	2.2787580	-0.4412250
H	-1.2290630	6.0567320	-0.7585240	H	-1.2083090	6.0633250	-0.7371090
Cl	-5.8052040	-4.0148120	0.0553140	Cl	-5.8166750	-4.0033110	0.0469480
C	3.5579120	-2.9355760	-0.4082820	C	3.5615410	-2.9289050	-0.4300790
C	4.4145970	-3.8467710	0.2215670	C	4.4001600	-3.8106790	0.2631070
C	4.1143400	-1.8931330	-1.1654280	C	4.1391430	-1.9122260	-1.2067610
C	5.8035700	-3.7239650	0.0983980	C	5.7920900	-3.6828620	0.1845750
H	3.9957510	-4.6586930	0.8111250	H	3.9649240	-4.6030060	0.8669840
C	5.4991700	-1.7659950	-1.2875380	C	5.5268740	-1.7801960	-1.2848790
H	3.4581870	-1.1797060	-1.6567140	H	3.4980780	-1.2225460	-1.7494550
C	6.3488810	-2.6822350	-0.6555850	C	6.3582740	-2.6663010	-0.5884380
H	6.4545070	-4.4388330	0.5935190	H	6.4289050	-4.3746620	0.7283460
H	5.9165810	-0.9550110	-1.8775560	H	5.9607360	-0.9899040	-1.8908250
H	7.4261910	-2.5830020	-0.7515890	H	7.4377550	-2.5638460	-0.6504550
Molecule **6i** (gas phase)				Molecule **6i** (PCM for CH_2Cl_2)			

E = -1815.86790094, **H (0K)** = -1815.458000,
H (298K) = -1815.429777, **G (298K)** = -1815.520839 au.
Imaginary frequency = 0.

C	1.0768710	0.2396600	1.4942300
N	0.2306540	0.1002310	0.4064600
C	0.7050230	-0.8822430	-0.3696940
C	-0.9462430	0.8697550	0.1465800
C	-2.2199660	0.3163690	-0.0324930
C	-2.3488890	2.5789780	-0.2236830
C	-0.9997800	2.2950650	0.0235480
C	-2.7144090	-1.0627040	0.0051040
C	-3.7291190	-1.4819870	-0.8778440
C	-4.2195730	-2.7868330	-0.8500160
C	-3.6875960	-3.7000310	0.0613600
C	-2.6873830	-3.3141790	0.9550090
C	-2.2140920	-2.0026860	0.9278340
C	0.1489700	3.2224940	0.1107200
C	1.3765520	2.9060790	-0.5018440
C	2.4777700	3.7597150	-0.3992330
C	2.3705270	4.9546830	0.3159340
C	1.1503470	5.2903740	0.9113560
C	0.0497540	4.4386710	0.8111490
H	2.9283410	-0.8977180	1.9742620
H	0.9018280	0.9940300	2.2438340
H	0.2511030	-1.2137480	-1.2898740
H	-4.1311050	-0.7827320	-1.6049160
H	-5.0016190	-3.0940240	-1.5357870
H	-2.3038460	-4.0240330	1.6801760
H	-1.4752150	-1.6971110	1.6625050
H	1.4621490	2.0023510	-1.1000210
H	3.4103430	3.5002190	-0.8936200
H	3.2216950	5.6254500	0.3924910
H	1.0474550	6.2298140	1.4468930
H	-0.9085130	4.7280320	1.2251130
N	1.8146230	-1.3875040	0.1915650
C	2.5963830	-2.5313990	-0.3336890
H	2.3829150	-3.4000650	0.2957420
H	2.2010070	-2.7367410	-1.3320340
C	-3.2302870	3.8286820	-0.4426560
O	-2.6716800	4.9378820	-0.3381210
O	-4.4271780	3.5149910	-0.6856430
Cl	-4.2890800	-5.3507690	0.0933580
C	4.0822100	-2.2529090	-0.3748630
C	4.9477420	-2.9255440	0.4961260
C	4.6089960	-1.3276920	-1.2882080
C	6.3229760	-2.6749320	0.4603260
H	4.5503240	-3.6535750	1.1993200
C	5.9799760	-1.0742510	-1.3216500
H	3.9480680	-0.8034790	-1.9741580
C	6.8392590	-1.7476030	-0.4463680
H	6.9857510	-3.2042880	1.1379250
H	6.3787020	-0.3567600	-2.0321220
H	7.9066650	-1.5514850	-0.4757290
N	-3.0220010	1.3931900	-0.2705980
H	-4.0301320	1.4603500	-0.4187420

E = -1815.92353560, **H (0K)** = -1815.513030,
H (298K) = -1815.484750, **G (298K)** = -1815.576384 au
Imaginary frequency = 0.

C	1.7872870	-1.2112010	1.3862880
C	0.9144050	-0.1824920	1.5789650
N	0.1909940	-0.0343650	0.4037020
C	0.6266560	-0.9496460	-0.4768400
C	-0.8374090	0.9267780	0.1549940
C	-2.1891150	0.6206470	-0.0193890
C	-1.9014440	2.8664350	-0.2315560
C	-0.6357870	2.3355930	0.0270400
C	-2.9276080	-0.6480400	0.0087330
C	-4.0142910	-0.8598750	-0.8609260
C	-4.7375930	-2.0519410	-0.8348680
C	-4.3663620	-3.0529250	0.0630330
C	-3.2938460	-2.8746850	0.9372450
C	-2.5854600	-1.6736410	0.9097340
C	0.6638820	3.0347590	0.1599380
C	1.7484220	2.6949470	-0.6668940
C	2.9853080	3.3311020	-0.5244720
C	3.1579350	4.3187530	0.4489560
C	2.0845940	4.6664410	1.2768240
C	0.8504850	4.0299420	1.1343780
H	2.5250920	-1.6416530	2.0433830
H	0.7392760	0.4499430	2.4337200
H	0.2538540	-1.0706040	-1.4812490
H	-4.2920010	-0.0961360	-1.5807330
H	-5.5713900	-2.2018480	-1.5116520
H	-3.0245060	-3.6539220	1.6416200
H	-1.7771930	-1.5295800	1.6186110
H	1.6178150	1.9452660	-1.4423060
H	3.8084560	3.0607350	-1.1798530
H	4.1171240	4.8163220	0.5591650
H	2.2077600	5.4365090	2.0330550
H	0.0188050	4.3073730	1.7732120
N	1.5888930	-1.6780770	0.0973760
C	2.2933130	-2.8214050	-0.5365530
H	1.9638540	-3.7292600	-0.0263590
H	1.9370410	-2.8628400	-1.5679500
C	-2.4494150	4.2528700	-0.5094400
O	-1.6324030	5.2046160	-0.5800670
O	-3.7091760	4.2909120	-0.6525620
Cl	-5.2688570	-4.5653160	0.0972710
C	3.7984120	-2.6827190	-0.4732550
C	4.5440300	-3.5038100	0.3820340
C	4.4634200	-1.7429260	-1.2752900
C	5.9364230	-3.3842860	0.4422640
H	4.0382680	-4.2422680	0.9986730
C	5.8522530	-1.6204550	-1.2129630
H	3.8967530	-1.1077940	-1.9508780
C	6.5914390	-2.4408950	-0.3527440
H	6.5044910	-4.0274080	1.1074810
H	6.3579010	-0.8910490	-1.8383940
H	7.6722420	-2.3468850	-0.3075580
N	-2.7902290	1.8214400	-0.2620390
H	-3.7776550	2.0042990	-0.3992330

Molecule **6i**
(PCM for DMSO)

E = -1815.93338442, **H (0K)** = -1815.522977,
H (298K) = -1815.494592, **G (298K)** = -1815.587614

Molecule **4j**
(gas phase)

E = -1431.50154287, **H (0K)** = -1431.078544,
H (298K) = -1431.050322, **G (298K)** = -1431.139736

au.				au.			
Imaginary frequency = 0.				Imaginary frequency = 0.			
C	1.6689880	-1.3410870	1.4725040	C	0.4934940	2.5562580	1.3502620
C	0.8490540	-0.2666430	1.6428580	C	-0.5299250	1.6582690	1.4127540
N	0.1658270	-0.0812050	0.4482400	N	-0.3468450	0.7434880	0.3847950
C	0.5735860	-1.0206070	-0.4210270	C	0.7534170	1.0897060	-0.2915240
C	-0.7947130	0.9400520	0.1745810	C	-1.1469090	-0.4044810	0.1117630
C	-2.1612290	0.7271530	-0.0196200	C	-0.6552840	-1.7208810	-0.0891210
C	-1.7159240	2.9460760	-0.2403700	N	-1.6964130	-2.5288630	-0.3554200
C	-0.4956510	2.3293610	0.0401980	C	-2.8346130	-1.7692610	-0.3042120
C	-2.9841260	-0.4890200	0.0008480	C	-2.5580620	-0.4153460	-0.0206830
C	-4.0655690	-0.6319210	-0.8887090	C	0.7277000	-2.2275520	-0.0100700
C	-4.8682930	-1.7722100	-0.8704890	C	1.1772580	-3.2031740	-0.9245310
C	-4.5815450	-2.7894590	0.0399580	C	2.4676260	-3.7134140	-0.8638750
C	-3.5148060	-2.6800490	0.9322450	C	3.3627080	-3.2626820	0.1193870
C	-2.7262080	-1.5298850	0.9119490	C	2.9398690	-2.3009090	1.0443300
C	0.8538750	2.9294990	0.1760660	C	1.6321460	-1.8015830	0.9743490
C	1.8751470	2.5959500	-0.7295540	C	-3.4600650	0.7524110	0.0673500
C	3.1590460	3.1322330	-0.5891930	C	-3.1786830	1.9289070	-0.6533850
C	3.4423560	4.0083700	0.4624130	C	-4.0024910	3.0527570	-0.5542960
C	2.4334810	4.3444210	1.3722360	C	-5.1311570	3.0235130	0.2685560
C	1.1520370	3.8074820	1.2314900	C	-5.4301870	1.8573940	0.9809080
H	2.3612260	-1.8127650	2.1504160	C	-4.6064400	0.7357630	0.8825010
H	0.6830330	0.3724850	2.4943170	H	0.7343980	3.3993960	1.9764180
H	0.2217910	-1.1222120	-1.4352900	H	-1.3650240	1.5764610	2.0884770
H	-4.2761880	0.1434000	-1.6186700	H	1.1321640	0.5767580	-1.1595320
H	-5.6967600	-1.8703450	-1.5630900	H	0.4879020	-3.5615890	-1.6821720
H	-3.3088010	-3.4724560	1.6431410	H	2.8053110	-4.4674190	-1.5679940
H	-1.9200330	-1.4390970	1.6317740	H	3.5952980	-1.9585450	1.8370220
H	1.6596820	1.9258420	-1.5570860	H	1.3068240	-1.0971930	1.7356100
H	3.9337260	2.8678670	-1.3033230	H	-2.3240890	1.9500330	-1.3249060
H	4.4389030	4.4260580	0.5725820	H	-3.7716880	3.9433650	-1.1331480
H	2.6449550	5.0236190	2.1932660	H	-5.7785630	3.8927300	0.3427300
H	0.3726550	4.0700560	1.9399430	H	-6.3149130	1.8166890	1.6101570
N	1.4787120	-1.7986550	0.1789310	H	-4.8590120	-0.1716740	1.4186760
C	2.1502960	-2.9708760	-0.4372780	N	1.3013830	2.1816340	0.2840110
H	1.8432940	-3.8526670	0.1289840	C	-4.1188780	-2.4421760	-0.5809180
H	1.7457570	-3.0574860	-1.4470320	O	-5.2196050	-1.9209410	-0.5628650
C	-2.1504350	4.3653460	-0.5237350	O	-3.9770000	-3.7622230	-0.8677980
O	-1.2655210	5.2600770	-0.5622510	H	-3.0174360	-3.9539930	-0.8077420
O	-3.3989860	4.5063800	-0.7047860	C	2.5146220	2.8193740	-0.1357950
Cl	-5.5860000	-4.2373950	0.0653430	C	2.5630170	4.2132090	-0.2246460
C	3.6573600	-2.8330460	-0.4533040	C	3.6276160	2.0348580	-0.4522100
C	4.4422060	-3.5701530	0.4429230	C	3.7488700	4.8274520	-0.6306240
C	4.2840510	-1.9754350	-1.3701320	H	1.6819370	4.8062590	-0.0012810
C	5.8354830	-3.4463000	0.4311400	C	4.8013300	2.6618880	-0.8742570
H	3.9664570	-4.2459180	1.1487240	H	3.5821670	0.9547350	-0.3517300
C	5.6743400	-1.8492810	-1.3806130	C	4.8663340	4.0551980	-0.9599230
H	3.6860370	-1.4088630	-2.0790250	H	3.7918730	5.9095340	-0.7033340
C	6.4527860	-2.5839680	-0.4784540	H	5.6681160	2.0579120	-1.1231190
H	6.4337610	-4.0235570	1.1295620	H	5.7836540	4.5374320	-1.2824200
H	6.1501580	-1.1843150	-2.0950730	O	4.6122310	-3.8240940	0.0944720
H	7.5343260	-2.4874320	-0.4895410	C	5.5363300	-3.4663540	1.1119650
N	-2.6761930	1.9642370	-0.2785320	H	5.1542190	-3.7216270	2.1085830
H	-3.6479160	2.2052020	-0.4331810	H	6.4394840	-4.0444730	0.9120420
				H	5.7776430	-2.3954250	1.0791490
Molecule **4j** (PCM for CH_2Cl_2)				Molecule **4j** (PCM for DMSO)			
**E** = -1431.53563772, **H (0K)** = -1431.112751,				**E** = -1431.54218855, **H (0K)** = -1431.119402,			

**H (298K)** = -1431.084488, **G (298K)** = -1431.174707 au<br>Imaginary frequency = 0.				**H (298K)** = -1431.091124, **G (298K)** = -1431.181372 au<br>Imaginary frequency = 0.			
C	0.6996340	2.3091690	1.6010940	C	0.7147290	2.2884080	1.6205710
C	-0.2683800	1.3539040	1.6778610	C	-0.2469370	1.3273750	1.6993680
N	-0.2441830	0.6425750	0.4861040	N	-0.2398370	0.6352090	0.4956080
C	0.7152070	1.1578200	-0.2932100	C	0.7044590	1.1670270	-0.2917140
C	-1.0750900	-0.4736530	0.1471540	C	-1.0703560	-0.4800800	0.1527870
C	-0.6366410	-1.8043620	-0.0862350	C	-0.6305410	-1.8102320	-0.0836940
N	-1.7065010	-2.5613750	-0.3959620	N	-1.6986050	-2.5682290	-0.3971490
C	-2.8144410	-1.7510520	-0.3432700	C	-2.8082830	-1.7580740	-0.3452430
C	-2.4771430	-0.4197700	-0.0077830	C	-2.4711160	-0.4269380	-0.0049190
C	0.7263640	-2.3661300	-0.0298510	C	0.7340390	-2.3690890	-0.0303360
C	1.1025500	-3.3994240	-0.9138010	C	1.1123590	-3.3978040	-0.9187220
C	2.3732770	-3.9598390	-0.8748120	C	2.3846820	-3.9552840	-0.8823720
C	3.3218890	-3.5003600	0.0538130	C	3.3323090	-3.4968070	0.0479830
C	2.9712370	-2.4802650	0.9462820	C	2.9797150	-2.4798340	0.9435290
C	1.6841610	-1.9307150	0.8991140	C	1.6917020	-1.9329660	0.8986870
C	-3.3364180	0.7754840	0.1431870	C	-3.3324130	0.7667340	0.1493400
C	-3.0743550	1.9451650	-0.5938580	C	-3.0749290	1.9373100	-0.5879200
C	-3.8626270	3.0884970	-0.4343700	C	-3.8673980	3.0777550	-0.4267800
C	-4.9308020	3.0851820	0.4671590	C	-4.9346250	3.0702220	0.4761440
C	-5.2047640	1.9275940	1.2044040	C	-5.2025830	1.9121230	1.2151480
C	-4.4159130	0.7869480	1.0448310	C	-4.4091220	0.7745390	1.0546310
H	0.9911570	3.0793360	2.2959190	H	1.0162040	3.0474680	2.3232800
H	-0.9693370	1.1162740	2.4607450	H	-0.9319300	1.0740570	2.4913810
H	0.9815960	0.7979030	-1.2737150	H	0.9550410	0.8249470	-1.2826410
H	0.3801210	-3.7588930	-1.6394480	H	0.3923610	-3.7555030	-1.6476940
H	2.6531390	-4.7529180	-1.5615710	H	2.6661320	-4.7440010	-1.5735950
H	3.6736340	-2.1167620	1.6868280	H	3.6821970	-2.1141530	1.6827940
H	1.4265520	-1.1683390	1.6280700	H	1.4352160	-1.1697430	1.6269120
H	-2.2597620	1.9522430	-1.3128000	H	-2.2595580	1.9488670	-1.3056390
H	-3.6462580	3.9773900	-1.0205660	H	-3.6531150	3.9682780	-1.0110350
H	-5.5462710	3.9715960	0.5907870	H	-5.5519000	3.9550080	0.6020580
H	-6.0348170	1.9125610	1.9052740	H	-6.0288180	1.8957840	1.9204970
H	-4.6358990	-0.1075580	1.6185100	H	-4.6214380	-0.1189460	1.6330360
N	1.3103180	2.1700190	0.3611960	N	1.3044050	2.1715980	0.3682360
C	-4.1000870	-2.3553300	-0.6865530	C	-4.0880960	-2.3630770	-0.6968170
O	-5.1938450	-1.8025680	-0.7332350	O	-5.1862980	-1.8157010	-0.7513460
O	-4.0059710	-3.6829230	-0.9830880	O	-3.9906740	-3.6904780	-0.9963230
H	-3.0534870	-3.9101580	-0.8919280	H	-3.0375480	-3.9156630	-0.9021200
C	2.3915270	2.9728040	-0.1453000	C	2.3734210	2.9873370	-0.1444360
C	3.5017540	3.2079130	0.6692920	C	3.4987620	3.2104840	0.6525560
C	2.3080380	3.5019650	-1.4352020	C	2.2626520	3.5394980	-1.4226030
C	4.5460440	3.9936950	0.1779660	C	4.5317120	4.0071590	0.1545250
H	3.5562720	2.7727320	1.6617440	H	3.5739500	2.7583560	1.6358980
C	3.3667570	4.2735800	-1.9191280	C	3.3101280	4.3222270	-1.9134040
H	1.4270570	3.3296540	-2.0447070	H	1.3698850	3.3763120	-2.0171480
C	4.4823760	4.5232040	-1.1146910	C	4.4413410	4.5596570	-1.1269930
H	5.4128350	4.1807640	0.8034800	H	5.4102940	4.1849550	0.7661240
H	3.3100050	4.6884220	-2.9202290	H	3.2323410	4.7553320	-2.9053230
H	5.2991740	5.1294660	-1.4931300	H	5.2492890	5.1744770	-1.5106690
O	4.5475570	-4.1078900	0.0096210	O	4.5590200	-4.1015370	0.0024210
C	5.5513170	-3.6842160	0.9330020	C	5.5626870	-3.6777410	0.9282100
H	5.2371980	-3.8588130	1.9685160	H	5.2470880	-3.8550870	1.9625040
H	6.4311440	-4.2892970	0.7135950	H	6.4431270	-4.2814480	0.7079800
H	5.7937360	-2.6239300	0.7963150	H	5.8032660	-2.6171950	0.7933620
Molecule **5j**<br>(gas phase)<br>**E =** -1431.49817184, **H (0K)** = -1431.075159,<br>**H (298K)** = -1431.046770, **G (298K)** = -1431.136625				Molecule **5j**<br>(PCM for CH_2Cl_2)<br>**E =** -1431.51649480, **H (0K)** = -1431.093897,<br>**H (298K)** = -1431.065400, **G (298K)** = -1431.155874			

au.				au			
Imaginary frequency = 0.				Imaginary frequency = 0.			
C	0.6271850	2.2365470	1.6903080	C	0.6004780	2.2445220	1.6678550
C	-0.1812590	1.1531610	1.7704490	C	-0.1940510	1.1507400	1.7490600
N	-0.1790820	0.5716440	0.4983060	N	-0.1808350	0.5648590	0.4790050
C	0.5997650	1.2532900	-0.4009310	C	0.5937700	1.2547870	-0.4178360
C	-0.9186740	-0.5949600	0.1672340	C	-0.9087010	-0.6091460	0.1469430
C	-0.3732740	-1.8688530	-0.0443510	C	-0.3514230	-1.8789590	-0.0662830
C	-2.6156010	-1.9810920	-0.3419380	C	-2.5913370	-2.0115820	-0.3659540
C	-2.3276440	-0.6488640	-0.0154690	C	-2.3159110	-0.6776780	-0.0331370
C	1.0040270	-2.3701620	0.0032750	C	1.0321400	-2.3644200	-0.0116860
C	1.4097880	-3.4345500	-0.8284250	C	1.4639500	-3.4022960	-0.8639460
C	2.7030350	-3.9357770	-0.7794350	C	2.7641330	-3.8862300	-0.8062250
C	3.6416430	-3.3784400	0.1027550	C	3.6815560	-3.3396120	0.1062090
C	3.2612780	-2.3151760	0.9319830	C	3.2732150	-2.3038940	0.9582690
C	1.9552430	-1.8244440	0.8777430	C	1.9613970	-1.8295480	0.8941410
C	-3.2503560	0.5026760	0.1072650	C	-3.2549480	0.4591370	0.1127760
C	-3.0261060	1.6634990	-0.6524640	C	-3.1012250	1.6076980	-0.6820100
C	-3.8728420	2.7667140	-0.5280570	C	-3.9639600	2.6965910	-0.5319300
C	-4.9504370	2.7308670	0.3608500	C	-4.9877250	2.6570620	0.4193890
C	-5.1777440	1.5824970	1.1249030	C	-5.1442140	1.5206020	1.2198610
C	-4.3343320	0.4777310	0.9995890	C	-4.2841490	0.4310330	1.0685460
H	0.9226590	2.9431170	2.4487910	H	0.8862590	2.9604180	2.4216760
H	-0.7460410	0.7490570	2.5959350	H	-0.7482140	0.7366820	2.5766780
H	0.7145420	-3.8575940	-1.5482540	H	0.7848900	-3.8208410	-1.6010850
H	3.0136960	-4.7487800	-1.4273110	H	3.0917390	-4.6792470	-1.4706540
H	3.9634810	-1.8647950	1.6232100	H	3.9566590	-1.8656710	1.6752300
H	1.6783500	-1.0082680	1.5354960	H	1.6624150	-1.0387830	1.5732080
H	-2.1890280	1.6953900	-1.3431310	H	-2.3074790	1.6430470	-1.4218160
H	-3.6894670	3.6536630	-1.1278780	H	-3.8354400	3.5739720	-1.1592890
H	-5.6079210	3.5901910	0.4578230	H	-5.6573270	3.5041620	0.5369560
H	-6.0120200	1.5460850	1.8198670	H	-5.9347730	1.4824170	1.9638950
H	-4.5153510	-0.4118030	1.5945780	H	-4.4077740	-0.4471000	1.6947560
N	1.0923990	2.2807840	0.3707480	N	1.0718890	2.2889220	0.3507040
C	-3.8053060	-2.7334180	-0.7068620	C	-3.7849770	-2.7559130	-0.7293630
O	-3.7803640	-3.9282160	-0.9823760	O	-3.7721810	-3.9569910	-0.9933610
O	-4.9517700	-2.0140110	-0.7277410	O	-4.9185240	-2.0234100	-0.7673010
N	-1.4242400	-2.6769020	-0.3559450	N	-1.3931580	-2.6965730	-0.3810370
H	-1.3903780	-3.6715830	-0.5331820	H	-1.3380290	-3.6898430	-0.5631720
H	-5.6532760	-2.6280010	-1.0019070	H	-5.6403550	-2.6215590	-1.0269100
C	1.9700060	3.2825810	-0.1424170	C	1.9403710	3.3058100	-0.1530350
C	1.9770750	4.5692220	0.4083980	C	1.8110210	4.6251630	0.2965170
C	2.8256100	2.9713240	-1.2063160	C	2.9217530	2.9770230	-1.0957300
C	2.8509670	5.5363590	-0.0949420	C	2.6709970	5.6119030	-0.1934360
H	1.2907220	4.8271190	1.2081860	H	1.0346150	4.8867720	1.0080360
C	3.6846490	3.9480520	-1.7091780	C	3.7670170	3.9724850	-1.5892550
H	2.7958540	1.9731220	-1.6267470	H	3.0122880	1.9504990	-1.4311900
C	3.7076930	5.2319600	-1.1545730	C	3.6495030	5.2916540	-1.1385760
H	2.8483670	6.5328530	0.3370610	H	2.5640230	6.6338350	0.1578130
H	4.3446150	3.7004160	-2.5355690	H	4.5261680	3.7118820	-2.3207920
H	4.3826470	5.9866820	-1.5468130	H	4.3131430	6.0614080	-1.5205020
O	4.8876740	-3.9331600	0.0744240	O	4.9350730	-3.8752540	0.0859530
C	5.8951110	-3.3920380	0.9221940	C	5.9234700	-3.3422480	0.9722040
H	5.6237000	-3.4930850	1.9803500	H	5.6300350	-3.4777090	2.0190790
H	6.7947590	-3.9749830	0.7223580	H	6.8335030	-3.9078740	0.7731720
H	6.0852480	-2.3366260	0.6913830	H	6.1005400	-2.2797870	0.7716780
Molecule **5j** (PCM for DMSO) **E** = -1431.52023581, **H (0K)** = -1431.097755, **H (298K)** = -1431.069227, **G (298K)** = -1431.159941 au.				Molecule **6j** (gas phase) **E** = -1431.48350059, **H (0K)** = -1431.060597, **H (298K)** = -1431.032305, **G (298K)** = -1431.122053 au.			

Imaginary frequency = 0.				Imaginary frequency = 0.			
C	0.6112370	2.2369040	1.6704290	C	0.5033000	2.4870560	1.4467700
C	-0.1857190	1.1447230	1.7510050	C	-0.4903250	1.5558500	1.4997560
N	-0.1799760	0.5636020	0.4786780	N	-0.3206260	0.7072730	0.4164060
C	0.5933560	1.2551280	-0.4179750	C	0.7395050	1.1231040	-0.2840930
C	-0.9099750	-0.6091820	0.1462950	C	-1.1213570	-0.4326250	0.1041910
C	-0.3543330	-1.8799270	-0.0664850	C	-0.6005590	-1.7159320	-0.0946240
C	-2.5941900	-2.0108490	-0.3632910	C	-2.8637080	-1.7752990	-0.3279710
C	-2.3171240	-0.6766260	-0.0318750	C	-2.5475890	-0.4429030	-0.0434590
C	1.0301550	-2.3638880	-0.0149400	C	0.7665680	-2.2494710	-0.0304720
C	1.4675410	-3.3856830	-0.8836630	C	1.2216570	-3.1782570	-0.9911030
C	2.7696190	-3.8654840	-0.8300660	C	2.5045730	-3.7064300	-0.9323350
C	3.6821720	-3.3315050	0.0950270	C	3.3858700	-3.3158050	0.0886030
C	3.2677970	-2.3120350	0.9639640	C	2.9570320	-2.3944160	1.0530650
C	1.9544740	-1.8411240	0.9032890	C	1.6570570	-1.8806510	0.9902200
C	-3.2561710	0.4608500	0.1126760	C	-3.4396180	0.7331390	0.0457380
C	-3.1202580	1.5965550	-0.7035330	C	-3.0687260	1.9596410	-0.5384640
C	-3.9857940	2.6842990	-0.5587670	C	-3.8866600	3.0875730	-0.4347290
C	-4.9947480	2.6558630	0.4088160	C	-5.0997150	3.0093630	0.2535490
C	-5.1328870	1.5325690	1.2312550	C	-5.4894740	1.7911470	0.8196990
C	-4.2697700	0.4444110	1.0853270	C	-4.6736630	0.6637280	0.7182260
H	0.9023290	2.9499290	2.4249880	H	0.7443710	3.3045300	2.1060410
H	-0.7345500	0.7271080	2.5803500	H	-1.2990790	1.4139830	2.1976620
H	0.7911420	-3.7955850	-1.6279510	H	1.0934310	0.6741110	-1.1973930
H	3.1013130	-4.6468670	-1.5062230	H	0.5586340	-3.4822990	-1.7959840
H	3.9469190	-1.8851350	1.6916430	H	2.8473680	-4.4248080	-1.6698640
H	1.6498050	-1.0643170	1.5958390	H	3.6042640	-2.0964340	1.8696020
H	-2.3387940	1.6227660	-1.4567630	H	1.3238210	-1.2094310	1.7769110
H	-3.8711560	3.5517950	-1.2023170	H	-2.1510200	2.0236960	-1.1178570
H	-5.6665920	3.5016500	0.5225010	H	-3.5855020	4.0182500	-0.9087780
H	-5.9109000	1.5038740	1.9887280	H	-5.7431130	3.8814190	0.3306720
H	-4.3791270	-0.4232760	1.7285500	H	-6.4435750	1.7114480	1.3329620
N	1.0772670	2.2849900	0.3516040	H	-5.0041900	-0.2916380	1.1075260
C	-3.7907730	-2.7530920	-0.7211090	N	1.2750530	2.1980810	0.3276080
O	-3.7819500	-3.9554820	-0.9810620	C	-4.1336910	-2.6122980	-0.5894330
O	-4.9217070	-2.0175630	-0.7592830	O	-5.2281830	-2.0239650	-0.4848260
N	-1.3966520	-2.6969010	-0.3794200	O	-3.8540570	-3.8119100	-0.8597000
H	-1.3409730	-3.6908710	-0.5585610	N	-1.6936360	-2.4813580	-0.3682990
H	-5.6482680	-2.6136950	-1.0108520	H	-1.7791530	-3.4826970	-0.5456820
C	1.9469960	3.3023090	-0.1503790	C	2.4415210	2.9099050	-0.1128970
C	1.7985640	4.6257450	0.2809450	C	2.4126660	4.3058310	-0.1631310
C	2.9479060	2.9698590	-1.0709550	C	3.5813060	2.1930800	-0.4869200
C	2.6584420	5.6138510	-0.2065190	C	3.5501840	4.9921320	-0.5913590
H	1.0088990	4.8881810	0.9773750	H	1.5100530	4.8447610	0.1066350
C	3.7931900	3.9665880	-1.5627440	C	4.7058960	2.8920190	-0.9295900
H	3.0571440	1.9396500	-1.3894950	H	3.5947210	1.1098420	-0.4157700
C	3.6556450	5.2902560	-1.1311020	C	4.6941100	4.2884980	-0.9786490
H	2.5374300	6.6388850	0.1306190	H	3.5345550	6.0763930	-0.6355930
H	4.5682280	3.7036220	-2.2764910	H	5.5941850	2.3421780	-1.2236730
H	4.3192580	6.0609220	-1.5111150	H	5.5732040	4.8269990	-1.3179660
O	4.9371390	-3.8633490	0.0701980	O	4.6251730	-3.8881950	0.0573880
C	5.9175160	-3.3539230	0.9807680	C	5.5410390	-3.5923380	1.1049200
H	5.6121790	-3.5167750	2.0200260	H	5.1408700	-3.8921990	2.0813300
H	6.8290120	-3.9150540	0.7762960	H	6.4382830	-4.1726230	0.8875310
H	6.0971620	-2.2870270	0.8096350	H	5.7956870	-2.5247410	1.1257480
Molecule **6j**<br>(PCM for CH_2Cl_2)<br>**E =** -1431.53974248, **H (0K)** = -1431.116236,<br>**H (298K)** = -1431.087908, **G (298K)** = -1431.178329 au<br>Imaginary frequency = 0.				Molecule **6j**<br>(PCM for DMSO)<br>**E =** -1431.54979750, **H (0K)** = -1431.126354,<br>**H (298K)** = -1431.097952, **G (298K)** = -1431.188886 au<br>Imaginary frequency = 0.			

C	0.5013100	2.3389540	1.6136170	C	0.4751750	2.3304350	1.6319310
C	-0.3609760	1.2879520	1.6888110	C	-0.3548990	1.2547050	1.7149010
N	-0.2715810	0.5959970	0.4887110	N	-0.2681540	0.5749900	0.5069470
C	0.6167350	1.2203850	-0.2972160	C	0.5876350	1.2309820	-0.2900730
C	-1.0099170	-0.5751910	0.1348130	C	-0.9857550	-0.6070840	0.1481990
C	-0.4499160	-1.8351500	-0.0843990	C	-0.4067320	-1.8562300	-0.0830710
C	-2.7024310	-1.9522270	-0.3965330	C	-2.6545120	-2.0008060	-0.4164740
C	-2.4269190	-0.6282150	-0.0532520	C	-2.3993240	-0.6786260	-0.0518840
C	0.9320240	-2.3297230	-0.0197920	C	0.9821110	-2.3300300	-0.0153830
C	1.4025360	-3.2679440	-0.9626230	C	1.4699920	-3.2614390	-0.9558230
C	2.7005940	-3.7581620	-0.9022460	C	2.7761860	-3.7295510	-0.8918290
C	3.5779860	-3.3174920	0.1025510	C	3.6438610	-3.2721470	0.1141410
C	3.1311800	-2.3836210	1.0474780	C	3.1790150	-2.3448400	1.0567960
C	1.8190020	-1.9061270	0.9820890	C	1.8595630	-1.8897060	0.9878180
C	-3.3527790	0.5187590	0.0975240	C	-3.3430100	0.4523680	0.1145620
C	-3.1743030	1.6842440	-0.6678490	C	-3.2124170	1.6117340	-0.6691480
C	-4.0208050	2.7852550	-0.5066700	C	-4.0761980	2.6971250	-0.4921520
C	-5.0602600	2.7398560	0.4264150	C	-5.0834770	2.6419320	0.4753420
C	-5.2483290	1.5844710	1.1933580	C	-5.2211290	1.4935590	1.2635030
C	-4.4026340	0.4856780	1.0311980	C	-4.3573690	0.4108500	1.0864520
H	0.7521250	3.1015420	2.3323060	H	0.7179940	3.0934330	2.3529080
H	-1.0146920	0.9620090	2.4808460	H	-0.9814240	0.9033190	2.5178690
H	0.8708130	0.9314790	-1.3044890	H	0.8304280	0.9582340	-1.3045450
H	0.7491580	-3.6059220	-1.7616520	H	0.8255630	-3.6109260	-1.7571530
H	3.0565550	-4.4788430	-1.6314580	H	3.1456950	-4.4435820	-1.6209430
H	3.7794450	-2.0361170	1.8426600	H	3.8199250	-1.9825870	1.8511790
H	1.4831070	-1.2110470	1.7452670	H	1.5122350	-1.1973380	1.7479050
H	-2.3819560	1.7213670	-1.4103640	H	-2.4414800	1.6584500	-1.4332000
H	-3.8720200	3.6725500	-1.1156750	H	-3.9631250	3.5813140	-1.1130450
H	-5.7207010	3.5930480	0.5517960	H	-5.7553730	3.4839840	0.6140210
H	-6.0567860	1.5376880	1.9175200	H	-6.0005490	1.4418940	2.0185380
H	-4.5565680	-0.4100030	1.6237190	H	-4.4670290	-0.4770020	1.7010130
N	1.1110550	2.2774010	0.3665690	N	1.0621960	2.2963210	0.3729020
C	-3.9507010	-2.7261040	-0.7684410	C	-3.8887140	-2.7738860	-0.8164530
O	-5.0343410	-2.0936940	-0.8435800	O	-4.9759090	-2.1459560	-0.9169820
O	-3.7523400	-3.9621930	-0.9751340	O	-3.6918530	-4.0106420	-1.0264480
N	-1.5077150	-2.6316520	-0.4128890	N	-1.4494520	-2.6648860	-0.4303030
H	-1.4929350	-3.6258260	-0.6080670	H	-1.4099540	-3.6549130	-0.6395740
C	2.1035880	3.1911130	-0.1370510	C	2.0150010	3.2434960	-0.1450180
C	1.8658600	4.5648060	-0.0518650	C	1.7438260	4.6088220	-0.0315770
C	3.2798100	2.6853160	-0.6949840	C	3.1853730	2.7771070	-0.7475350
C	2.8328810	5.4477170	-0.5364160	C	2.6712980	5.5240890	-0.5333020
H	0.9374410	4.9366250	0.3689060	H	0.8201940	4.9496390	0.4241900
C	4.2317590	3.5802630	-1.1883580	C	4.0972530	3.7041300	-1.2573050
H	3.4570750	1.6154150	-0.7285870	H	3.3887390	1.7128420	-0.8032390
C	4.0119530	4.9583780	-1.1071910	C	3.8440590	5.0747340	-1.1485570
H	2.6567860	6.5167910	-0.4770860	H	2.4689340	6.5870800	-0.4520820
H	5.1487180	3.1970180	-1.6239980	H	5.0095480	3.3514930	-1.7272540
H	4.7577270	5.6495140	-1.4866970	H	4.5590000	5.7908930	-1.5409290
O	4.8325070	-3.8523210	0.0757990	O	4.9078920	-3.7842820	0.0911710
C	5.7686850	-3.4569910	1.0819210	C	5.8394800	-3.3575880	1.0908160
H	5.4103560	-3.7257390	2.0818180	H	5.4901470	-3.6234250	2.0944540
H	6.6850680	-4.0051110	0.8639610	H	6.7666590	-3.8880560	0.8751370
H	5.9667700	-2.3802050	1.0365480	H	6.0129660	-2.2775050	1.0327580
